# Acceptability of emergent *Aedes aegypti* vector control methods in Ponce, Puerto Rico: A qualitative assessment

**DOI:** 10.1371/journal.pgph.0002744

**Published:** 2024-03-06

**Authors:** Carmen L. Pérez-Guerra, Coral Rosado-Santiago, Sue Anette Ramos, Karla Michelle Marrero-Santos, Gladys González-Zeno, Susanna K. Partridge, Vanessa Rivera-Amill, Gabriela Paz-Bailey, Liliana Sánchez-González, Mary H. Hayden

**Affiliations:** 1 Division of Vector-Borne Diseases, Centers for Disease Control and Prevention, Dengue Branch, San Juan, Puerto Rico, United States of America; 2 Ponce Health Sciences University, Ponce Research Institute, Ponce, Puerto Rico, United States of America; 3 Division of Vector-Borne Diseases, Centers for Disease Control and Prevention, Fort Collins, Colorado, United States of America; 4 Lyda Hill Institute for Human Resilience, University of Colorado, Colorado Springs, Colorado, United States of America; Arizona State University, UNITED STATES

## Abstract

*Aedes aegypti* control has been fraught with challenges in Puerto Rico. The government has implemented commonly used vector control methods, but arboviral epidemics still occur. It is necessary to explore new *Ae*. *aegypti* control methods. This study aimed to understand the perceptions of community members in Ponce, Puerto Rico about emergent and traditional *Ae*. *aegypti* vector control methods and determine their acceptability and support for these methods. We identified the type of information needed to increase support for emergent vector control methods, and the preferred strategies to disseminate this information. Four group discussions were conducted with a total of 32 participants representing eight of the 14 clusters participating in the Communities Organized for the Prevention of Arboviruses (COPA), a project designed to mobilize communities in Ponce, Puerto Rico to prevent diseases transmitted by mosquitoes. Group discussions began with an overview of different methods used for controlling *Ae*. *aegypti* mosquitoes. These overviews facilitated participant understanding of the mosquito control methods presented. Use of source reduction, autocidal gravid ovitraps (AGO), and manual application of larvicide for arboviral mosquito control received support from almost all participants. Vector control methods that use more familiar techniques in Puerto Rico such as truck-mounted larvicide spraying (TMLS) and insecticide residual spraying received support from most participants. More than half of participants supported the use of emergent mosquito control methods including *Wolbachia* suppression, *Wolbachia* replacement, or genetically modified mosquitoes (GMM). Participants preferred to receive vector control information through house-to-house visits with the distribution of written materials, followed by dissemination of information through traditional (i.e., radio, television) and social media. The detailed information resulting from this study was used to develop messages for a communications campaign to garner future community support. Community acceptance and support are critical for the success of vector control programs using emergent mosquito control methods.

## Introduction

Dengue [DENV], Zika [ZIKV] and chikungunya [CHIKV] viruses present an ongoing public health threat to Puerto Rico and other countries in tropical and subtropical regions of the world where their vectors, *Aedes aegypti* and *Aedes albopictus* mosquitoes, are established. These diseases cause a substantial economic and social burden to individuals and healthcare systems worldwide [1–8] and have resulted in large epidemics in Puerto Rico [9–11].

Vector control as a mechanism to reduce arbovirus transmission has been fraught with challenges in Puerto Rico. Traditionally used *Ae*. *aegypti* vector control methods like truck-mounted ultra-low volume insecticide spraying (ULV), source reduction, individual indoor insecticide spraying, and educational campaigns have been insufficient to prevent arboviral transmission [12–16]. Also, resistance to many insecticides has been reported, adding to the vector control challenges on the island [14]. More recently, use of the autocidal gravid ovitraps (AGO) in Puerto Rico proved to be effective in the reduction of CHIKV transmission [17]. Further studies are needed to assess household acceptability of the required maintenance and the sustainability of AGO [17–19]. S1 Appendix contains more information on these vector control methods.

In addition to traditional vector control methods, emergent vector control methods have shown promise for controlling *Aedes* mosquitoes or reducing arbovirus disease transmission. These methods include use of mosquitoes with *Wolbachia*, (a naturally occurring bacteria that reduces transmission of viruses such as dengue), genetic modification of mosquitoes (GMM), and use of truck-mounted larvicide spraying (TMLS). With TMLS larvicides are sprayed, different from truck mounted ULV insecticide spraying where adulticides are sprayed. All have been implemented in different countries to control *Ae*. *aegypti* mosquito populations [20–29]. Strains of the bacterium *Wolbachia* have been shown to block replication and transmission of DENV, ZIKV, and CHIKV in *Ae*. *aegypti* mosquitoes [30]. Mosquito control programs implementing the *Wolbachia* suppression technique (male mosquitoes with *Wolbachia*) [28, 29] have been shown to reduce mosquito populations. *Wolbachia* replacement technique (male and female mosquitoes with *Wolbachia*) [20, 22] targeting *Aedes* mosquitoes have reduced dengue incidence. Releases of GMM (the *Ae*. *aegypti* RIDL strain, OX513A) have been shown to suppress wild *Aedes* mosquito populations [31], whereas TMLS has been effective in reducing *Ae*. *aegypti* indices [25–27, 32].

Community acceptance and support are critical for the success of vector control programs using emergent mosquito control methods [22–24, 33, 34]. Research assessing community acceptability to GMM [23], *Wolbachia* suppression, [20, 28, 29] and *Wolbachia* replacement [20] has shown that projects were successful [20, 24, 28, 29, 33, 35] where the community was actively engaged early in the process, providing awareness of the proposed technique characteristics, and ensuring its acceptability. Community acceptability studies for *Wolbachia* suppression [28, 29, 33, 36] and GMM [23, 37, 38] have been published in other countries, but not for community acceptability of TMLS. Limited information is available on the acceptability of control methods in Puerto Rico. As part of the Communities Organized for the Prevention of Arboviruses (COPA) project work, both qualitative and quantitative assessments were conducted, and the latter has been published [39]. COPA is a community-based project developed in collaboration with the Ponce Health Sciences University, the Puerto Rico Vector Control Unit, and the Centers for Disease Control and Prevention (CDC) Dengue Branch, implemented in Ponce, a municipality in southern Puerto Rico with high incidence of mosquito-borne diseases [9, 10, 16]. Qualitative methods using group discussions were used to explore participants’ opinions, their support for current and new vector control methods in the study communities and their preferred ways to receive information on the methods.

## Methods

Between April 21 and May 16, 2018, we recruited participants and conducted four group discussions (GD) in Spanish with a duration of one to two hours each. To generate conversations among participants, we developed a discussion guide and a slide set including descriptions and illustrations of the following eight vector control methods: source reduction, manual application of larvicides, TMLS, insecticide residual spraying inside houses (IRS), AGO, male and female mosquitoes with *Wolbachia* (*Wolbachia* replacement), male mosquitoes with *Wolbachia* (*Wolbachia* suppression), and GMM. Participants were asked about their familiarity and support for each vector control method, with questions to learn their reasons for support or opposition.

### Study site

The study was conducted in Ponce, Puerto Rico. Ponce is located on the southern coast of Puerto Rico and has a total population of approximately 135,000 residents. Participants were recruited in Ponce from six *barrios* (neighborhoods) with a total population of 51,573 residents [40].

### Sample selection

Using a snowball technique [41, 42] with residents, we identified community leaders with the assistance of our Ponce Health Sciences University collaborators. The initial community leaders then identified other leaders and residents who agreed to participate. Selection criteria included adults 21 ≥ years of age, residents and community leaders living in the COPA clusters. A total of 32 participants, including 10 leaders and 22 residents, took part in the GD, all ≥ 21 years of age.

### Data collection

Before starting each GD, a moderator assigned each participant a number to maintain anonymity, read a verbal consent form, and asked if participants agreed to allow audio recordings. A copy of the verbal consent form was given to participants, and the consent to participate was audio recorded. Two notetakers were assigned to each group session. The moderator read the slide set with each control method description (S1 Appendix), answered participants’ questions, and then continued the discussion based on the *a priori* developed discussion guide.

### Data analysis

The audio recordings were transcribed and merged with the notes from the two notetakers (S2 Appendix). We conducted a content analysis for which we developed eight categories based on the study’s objectives, the discussion guide, and the participants’ comments. Three analysts individually coded the comments under each category using MAXQDA 12 Plus software. Later, the analysts met to perform an intercoder analysis to discuss coding and which comments should be included under each category. These results were summarized in a presentation. Then, they regrouped the eight categories for each vector control method into five themes (Table 1). Participants’ comments were translated into English for publication purposes.

**Table 1:** Categories analyzed for each vector control method and resulting themes.

Vector control methods (VCM)	Categories analyzed for each VCM	Resulting themes from categories
• Source reduction spraying • Applying larvicide • Insecticide residual spraying • Truck-mounted larvicide • Autocidal gravid ovitraps • *Wolbachia* replacement • *Wolbachia* suppression • Genetically modified mosquitoes	• Knowledge of the VCM • Perceived benefits of the VCM • Perceived barriers of the VCM • Solutions to perceived barriers • Feasibility of implementation • Support for the VCM • Information needs of the VCM • Best strategies for dissemination and sources	• Awareness of vector control methods • Perceived Benefits/Advantages and Barriers/Disadvantages of Discussed Vector Control Methods • Solutions to Barriers and Feasibility of Implementation • Community support for the vector control methods • Participants’ Information Needs and Preferred Strategies for Dissemination

### Ethics statement

This study was reviewed and approved by the Ponce Medical School Foundation, Inc. Institutional Review Board, approval number 171110-VR. Participants provided verbal consent before group discussions which was audio recorded and witnessed by participants, moderators and notetakers. Moderators explained participation was voluntary and that participants could withdraw participation at any moment. Data was kept secure in password protected computers and locked cabinets. Only the principal researcher and analysts had access to the data. Complying with CDC guidelines, we keep raw data for six to ten years.

## Results

### Awareness of vector control methods

Participants knew about source reduction (the elimination of containers with water) because this method has been historically promoted by the local government as an individual action to control mosquitoes. Although participants did not know about TMLS, they associated it with truck-mounted ultra-low-volume (ULV) spraying of adulticides because the latter is commonly used by the central and municipal governments in PR. For TMLS, a participant said:

*“Like this in the truck? It has come this way, but they pass by very fast.”*

Most participants had not heard about manual larvicide application, the AGO or IRS, methods introduced during the Zika outbreak to treat the houses of pregnant women in selected municipalities. Some participants confused IRS with traditional insect extermination services commonly used in Puerto Rico. Almost no participants knew about emergent control methods such as GMM and TMLS. Two participants had heard about *Wolbachia* replacement and two had heard about *Wolbachia* suppression through television and from their participation in the COPA project. In one group discussion, a participant said:

*“I heard a report on television on a foreign channel. Yeah.”*

The vector control descriptions presented in the slide set and the question-and-answer session about the various methods encouraged GD participants to discuss the benefits and advantages, the barriers and disadvantages, and the feasibility of implementing each method. The following themes summarize the discussion.

### Perceived benefits/advantages and barriers/disadvantages of discussed vector control methods

#### Emergent vector control methods

*Benefits/advantages*. Except for *Wolbachia* replacement, participants perceived that the main benefit of almost all the vector control methods that were discussed was to reduce the number of mosquitoes in their communities. If mosquitoes were reduced, it would stop the transmission of viruses and reduce the overall burden of disease. For example, *Wolbachia* suppression was perceived as a good method to reduce mosquito numbers. That is, when male mosquitoes with *Wolbachia* mate with wild female mosquitoes, the resulting eggs will not hatch thus reducing the mosquito numbers in the treated areas. Also, participants noted that male mosquitoes do not bite. One participant said:

*“…[Wolbachia] is the best way to eliminate… mosquitoes…, It’s what we want.”*

*"At least you are giving me more hope; there will be no mosquitoes …*

*[it’s] ‘safe sex’. Because, as the [male] mosquito mates with the [female] mosquito…, the eggs won’t hatch…”*

For TMLS another participant said:

*“If you eliminate the larva, you eliminate the mosquito.”*

And for GMM a participant said:

*“[GMM, it] controls that they [mosquitoes] spread further.”*

Regarding *Wolbachia* methods, participants also noted that the use of the *Wolbachia* bacterium would not be harmful to other insects, would not cause diseases in humans, and could reduce virus or disease transmission. Participants noted the lack of adverse effects to the environment, humans, or animals because the method does not involve the use of chemicals. Participants said:

*"An advantage [of Wolbachia replacement] would be… [that] there is no risk of bees dying, or butterflies, or anything like that… But since that [Wolbachia] is being passed from generation to generation of mosquitoes, there will be mosquitoes but no risk of getting sick, or [no risk] of bees and other insects [dying]…"*

*“[Wolbachia replacement; not harmful to people, health or animals], they [the community] see it as beneficial.”*

*“It [Wolbachia replacement] is not a disease.”*

*“It [Wolbachia suppression] does not bring disease.”*

The absence of individual involvement for the method’s implementation was another advantage mentioned by participants when evaluating the emergent control methods. For example:

*“If they don’t have to do much, yes. If the activity [Wolbachia replacement]… they don’t need to do the activity, the community accepts everything. The laziness…”*

*"They don’t have to do anything [for TMLS]. The truck passes by, and that covers enough."*

*“… this type of activity [GMM] are activities where people are not going to have action on this.”*

Some participants associated TMLS with the usual truck-mounted ULV spraying of insecticides for adult mosquitoes and pointed out that this method has the advantage of killing larvae by reaching difficult-to-access areas such as backyards and hard-to-reach breeding sites, (i.e., gutters, septic systems) where mosquitoes could continue to lay eggs. They were aware that larvicides would not kill other insects and animals.

*“… if that [TMLS] comes by close to the area where they live and where standing water is at, and [larvicide] lands there, it will solve the problem there.”*

*“If it’s not insecticide, it will not be harmful to the environment. It will attack the mosquito…”*

*“We know that it’s not harmful to other insects…”*

*Barriers/disadvantages*. The main disadvantage highlighted by participants for emergent vector control methods were the lack of knowledge and information about the control methods, along with the lack of trust towards the government because they perceived officials do not provide timely information, especially for methods that involve laboratory manipulation of mosquitoes. For example, for *Wolbachia* suppression some participants said:

*“[Residents] are going to hold back… they don’t have the knowledge [on Wolbachia suppression]. You know, it’s something unfamiliar, nobody has any information about that…”*

At the time of the group discussions, mosquitoes for *Wolbachia* suppression and *Wolbachia* replacement were produced in a laboratory to inject the bacterium in them. Therefore, this is another disadvantage mentioned by participants.

*“Everything that comes from the laboratory will have consequences.”*

*“*… *[Wolbachia suppression] … what happens is that the mosquito will inject it to me*. *If the mosquito has it [Wolbachia]*, *it is as if they inject it to me*."

*“No. It’s a pilot project [Wolbachia replacement] … we are not [a] laboratory… No, we are not guinea pigs.”*

*"…if it had been implemented in the United States, [it] would have a little more.”*

For methods based on mosquito releases, most participants had concerns about the time it would take to produce the mosquitoes and to get the government’s approval and permits, the cost of its implementation to the government, and its sustainability. Lack of continuity and program sustainability were also noted by participants.

*"No [won’t support GMM], we are going back to the same thing… because if you stop [releasing the mosquitoes], so that they can mate, we go back to the same thing… there has to be consistency in these projects [referring to all methods], if there is no consistency, it doesn’t work.”*

*"Getting the mosquitoes… that’s distant, because you need to have authorization from the US; [local] government authorization, the big insecticide companies will oppose, …because that’s business, it generates $… That’s why it will be very difficult for the government to approve it.”*

*“I repeat to you, if it [Wolbachia suppression] is free, yes [I support it], if it isn’t free, no.”*

Participants agreed that GMM would be useful to reduce mosquitoes, but the perceived barriers were based on religious and cultural values and the lack of information about genetics. For instance, participants said:

*"[no] …Because they [GMM] are mutating… Like flu, which is still mutating, and we continue to spread new strains… Mutations are the consequence… [it] creates negative effects on our health… Mutations are the consequences of all things. "*

*-"Many [people] are not going to believe in that [GMM]. Elders… many are religious, and that is not from God, because of genetic [modification]."*

For TMLS, there was a concern that its smell could affect people with asthma. Others had a negative perception because they did not have sufficient information and distrusted its use. A few participants said:

*“I can’t assess [this method] because I don’t know what a larvicide is.”*

*“… if it [TMLS] hasn’t been used [in Puerto Rico] we don’t know the effects. There was evidence in Puerto Rico that they tested Agent Orange; they may say it’s for mosquitoes when it’s not."*

*“… the smell… would it affect people with respiratory problems…”*

Another participant doubted its effectiveness:

*“I don’t think it [TMLS] would reach everywhere and I don’t see its effectiveness. For example, these closed houses with a terrace behind that have containers with bromeliads and plants that hold water will not be reached… there are places where trucks won’t reach [gated communities], they must stay outside…”*

*Wolbachia* replacement was the least supported method because it would not reduce the number of mosquitoes in the communities. This was a disadvantage as residents noted that they would still feel the mosquito bites, *“mosquitoes will keep pestering*, *biting”*, even though these mosquitoes would not spread viruses.

#### Previously used vector control methods

*Benefits/advantages*. Participants liked previously used vector control methods such as source reduction and IRS with which they were familiar. Participants mentioned source reduction because it was a known vector control method and had been used in previous Puerto Rican educational campaigns. They perceived it was easy to eliminate containers, and therefore mosquitoes, with almost no involvement from the government.

*“When I go down the street, I also do it. I see a recipient full of water and I turn it over.”*

*“I understand that it is the fundamental activity to prevent the spread of the mosquito… if we engage our neighborhood… we won’t need the government, and I understand that we shouldn’t need spraying or fumigation."*

Many participants liked IRS because they associated it with the extermination services commonly used in Puerto Rico. Advantages that were identified by participants were its long-lasting effect on killing adult mosquitoes inside the houses and that it could be used along with larvicides to kill both the larva and the adult mosquito.

*“The benefit I see is that it kills the mosquito that gets into your house… the effect lasts a while.”*

*“If there is spraying [of larvicide], in addition to [killing] the larvae… an insecticide would have a greater impact.”*

*“[IRS] would kill mosquitoes that were not killed outside. After exterminating outside if there were larvae, those [mosquitoes] who stayed inside the house, then they would be exterminated.”*

Participants perceived that the manual application of larvicides was easy for the individual to undertake. Larvicide application was appealing because it was not harmful, and it would reduce the number of adult mosquitoes by reducing larvae. One participant noted:

*“The next [time there is the need to] store water, one can keep larvicide [on hand] and apply it [in containers with water], and we will feel safe.”*

The AGO were one of the methods preferred by participants. Almost all saw the benefit of the AGO because they do not involve chemicals or electric power; thus, they were perceived as friendly for the environment, animals, and humans. Participants thought AGO would be easy to use and liked that they could see the trapped mosquitoes. Therefore, they felt that AGO would reduce the number of mosquitoes and diseases they transmitted. One participant said:

*“It [AGO, doesn’t use] electricity, it’s something manual… It’s natural; just water and hay.”*

*Barriers/disadvantages*. For participants, source reduction was the least effective control method because households do not inspect their yards, and do not treat or eliminate containers frequently. A few participants said:

*“The problem is that… the [first] day, people get excited and do it. But that’s it. …The next day they forget that we must collect the garbage, …collect the tires; … [and] they start littering again…”*

*“Many [residents] do not care [about eliminating containers with water]; they have the problem [mosquito breeding], but they don’t want to contribute.”*

Some participants perceived IRS could be harmful to humans because it is a chemical and noted that it would require participation from residents over an extensive area to be effective. Another concern was that if is used repeatedly; mosquitoes could develop resistance.

*“It says there that if it’s used a lot, that mosquitoes would be resistant.”*

In response to the question regarding the use of IRS, participants indicated:

*“…it’s a disadvantage and I don’t support it [IRS]. …there may be a smell for a few hours… my wife will die [she has asthma]… If there are children [in the house],…they touch everything, they don’t wash their hands and put them in their mouth."*

*“…if everyone accepts, it could reduce [the number of mosquitoes], but there are always uncovered areas. …where nobody lives [abandoned houses] that won’t be sprayed. "*

*“The only disadvantage that I see is that [IRS] must be [done] by a trained professional, we do not have the access to do it.”*

Some participants thought that people would not see the need to use larvicide and would not use it because of its cost. Additionally, participants noted it would need to be used extensively to be effective. As some said:

*“It will work if you have larvicide. Once you run out of it, it will have a cost; you are not going to buy it. I’m not going to buy it.”*

*"People buy spray to kill mosquitoes because mosquitoes bite them. If the mosquito doesn’t bite them, they will not buy it. They won’t buy [larvicide] to kill a larva of a mosquito that has not bitten them; that will not work."*

*"I think it doesn’t reduce [mosquitoes] because… as long as one person does a good job eliminating [the larvae] and another person doesn’t, we go back to the same…"*

Others noted that AGO would need to be placed and maintained by most residents over an extensive area to be effective, and that their maintenance, as well as the traps themselves, would have a cost to users. In addition, participants believed that supplies were not available in retail stores. Participants were concerned about the cost of the AGO and the time required for maintenance:

**“You need many [AGO traps] to have an effect…*, *you will have to engage a lot of people*… *with spraying*, *it’s done at a given time*, *but you won’t interrupt the daily life of people; [it’s not feasible] even if they are for free*. "*

*"The maintenance, no one is going to be waiting for [the distribution of AGO supplies] to change [them]; maintenance is the problem."*

*“Well, the only downside is getting them [AGO]… it has a cost.”*

*"That [maintenance] would be an additional cost to the [AGO] trap…”*

### Recommendations to barriers and feasibility of implementation

Since lack of information was considered the main barrier to the adoption of control methods, participants agreed that providing information and educating community residents on vector control methods was crucial to garner support and increase community engagement. Participants suggested that it was essential to know the results of each method’s implementation in other countries and its effectiveness. Ensuring that methods did not have adverse effects on the environment, animals, or humans would also increase community support and improve the feasibility of implementing methods. Participants said:

*" My opinion: …if you will educate, you should have the appropriate elements and training for you to be able to hold a meeting [to provide information]… All can be done as long as it is in the right hands with proper information. "*

*“[Community leaders need to be] well trained [of methods], to master the subject, to be effective. Sometimes they [residents] ask questions and we [leaders] are not prepared to answer them.”*

*"…if it had been implemented in the (mainland) United States, we would have a little more credibility…"*

Other solutions to increase support for and feasibility of emergent interventions were associated with having municipal and state government collaboration and allocation of funds for implementation and sustainability, as well as having federal (US) approval for implementation. Participants defined what they meant by government collaboration for each discussed method. For source reduction, collaboration was defined as having the guidance of the municipal government to 1) create community support groups to conduct community garbage collection campaigns, 2) assist in conducting house visits with yard inspections, 3) educate communities on mosquito control and disease prevention; and 4) help the elderly and residents with disabilities in performing the vector control tasks. For methods such as IRS, *Wolbachia* suppression, *Wolbachia* replacement, and GMM, participants noted a need to allocate funds to ensure project sustainability. For all the methods, participants felt that for government collaboration to be successful, media should be used to keep residents informed about scheduled activities through promotional campaigns and advertisements.

Support for and feasibility of IRS, individual application of larvicides, and the use of AGO were associated with having services and supplies free of cost to residents. Also, for IRS and the AGO, the implementation would require the visible support of community leaders. Professional staff must be properly identified with the company or government logo for visits. Some participants would support these methods if:

*“If there is a company that would bring them [AGO] and [service] them every two months…”*

*“…if the municipality provided us the [AGO] traps for free.”*

### Community support for the vector control methods

After discussing the pros and cons of each method, participants were asked which method(s) they supported. All participants who supported source reduction said they had done it in the past and, in general, believed that community residents would support it. Also, all participants supported the use of AGO. But some believed that while residents would support the AGO, many would not commit to doing the maintenance (Table 2).

**Table 2:** Support for vector control methods among group discussions participants in Ponce, Puerto Rico– 2018.

Vector Control Method	# and % of participants who supported	# of % participants who were uncertain or neutral	Main perceived benefits	Main perceived concerns
Source reduction (n = 28)	28(100%)	0(0%)	• Easy to do for most residents	• Not everyone does it frequently or is able to do it
Autocidal gravid ovitraps (n = 27)	27(100%)	0(0%)	• Friendly to the environment, animals, and humans	• It has a cost • A lot of people would need to do it to be effective
Insecticide residual spraying (n = 27)	26(96%)	0(0%)	• Kills mosquitoes inside the house • Lasts a long time	• Could be harmful to humans • Requires participation over an extensive area • A need to pay a professional to do it
Larviciding (n = 28)	26(93%)	1(4%)	• Kills mosquito larvae (fewer adult mosquitoes)	• It has a cost • The need to use it extensively
Truck- mounted larvicide spraying (n = 27)	20(74%)	1(4%)	• Kills larvae in difficult-to-access areas	• Potential adverse effects to human health and the environment
*Wolbachia* suppression (n = 28)	18(64%)	2(7%)	• Male mosquitoes would not bite • Reduced mosquito numbers over time	• Lack of timely information about the method • The need to continue releases • High cost for the government
Genetically modified mosquitoes (n = 28)	18(64%)	1(4%)	• Less mosquitoes over time	• Lack of timely information about the method • High cost for the government • Not sustainable • It involves genetic modification
Wolbachia replacement(n = 27)	14(52%)	7(26%)	• Less arboviral diseases for humans • Safe for animals and the environment	• Lack of timely information about the method • Female mosquitoes would still bite • Would not reduce mosquito numbers • High cost for the government

Almost all (96%) participants would support IRS and believed the community would accept the service because they compared it with commonly used fumigation services. Likewise, individual application of larvicides to containers with water that could not be eliminated was supported by almost all (93%) participants who believed that the community would support its use if it were available at no cost or at low cost.

For emergent *Ae*. *aegypti* control methods to Puerto Rico, most (74%) participants would support TMLS in their communities and believed that residents would support the method, due to their prior knowledge of truck mounted ULV spraying of adulticides. More than half (64%) would support *Wolbachia* suppression and said that the community would as well. Similarly, more than half (64%) would support GMM but, contrary to *Wolbachia* suppression, they perceived that community support would be mixed because it involves genetic modification. *Wolbachia* replacement was the least supported method with about half (52%) of participants’ support; however, participants believed the community would support it because it would reduce disease transmission. (Table 2).

### Participants’ information needs and preferred strategies for dissemination

Participants noted that their information needs depended on the type of method being implemented. For example, for source reduction, residents would need to be educated about ways to treat and keep containers that could not be emptied free from larvae. For the AGO, residents would require training to correctly assemble and service the traps and information about their cost. For all methods, and especially for manual application of larvicide, TMLS, and IRS, residents would need information about the product, how it is used, its efficacy, impact on humans and the environment, and potential adverse events (i.e., its smell, amount used, and how to apply it). Particularly, for *Wolbachia* suppression, *Wolbachia* replacement, and GMM, residents would need information on *Wolbachia* and about the process of genetic modification, mosquito production, results of use in other countries, implementation logistics, permit procedures, US approval for implementation, effectiveness in disease reduction, government funding allocation, and project continuity and sustainability. (Table 3). For *Wolbachia* methods, participants said:

*“You have to see the pros and cons…the effectiveness, consistency.”*

*“Wolbachia, is it harmful to people and animals? Could it be harmful…?”*

*“That entails [shipping the mosquitoes, a functional laboratory, paying scientists], I imagine, a cost. And, how much?”*

**Table 3:** Categories of information reported as helpful to increase support for selected vector control methods during discussion groups, Ponce, Puerto Rico, 2018.

	Mosquito-borne diseases	Source reduction	Applying larvicide	Truck-Mounted larvicide spraying	Insecticide residual spraying	Autocidal gravid ovitraps	*Wolbachia* replacement	*Wolbachia* suppression	GMM
Information needs									
Arboviruses’ health effects	**X**								
Arbovirus transmission	**X**								
Product ingredients			**X**	**X**	**X**				
Adverse effect on health/ environment			**X**	**X**	**X**		**X**	**X**	**X**
Effectiveness					**X**	**X**	**X**		
Frequency of application				**X**					
Workshops		**X**	**X**			**X**			
Cost			**X**		**X**	**X**			
Long-lasting effect					**X**		**X**		
*Wolbachia*							**X**		
Implementation			**X**		**X**		**X**	**X**	**X**
Mosquito production							**X**	**X**	**X**
Results from previous studies				**X**			**X**	**X**	**X**
Funding								**X**	
Approval and licensing							**X**	**X**	**X**
Sustainability				**X**				**X**	**X**

Perceptions of preferred strategies to disseminate information depended on the age of residents. Older participants would prefer house visits, community meetings, and the distribution of written materials by project researchers working alongside community leaders. They also favored word of mouth with community leaders and other community spokespersons like religious leaders. Younger people would prefer to receive information via social media.

## Discussion

This study was one of the first steps to assess the acceptability of eight vector control methods, and to better understand information needs and preferred practices for dissemination of information within the COPA project in Ponce, Puerto Rico. After continued community education on the vector control methods and feedback obtained from different local stakeholders, the COPA project conducted additional surveys and phone interviews to assess the proposed implementation of *Wolbachia* suppression across the full cohort and found high acceptance (86%). The results provided insights into the development of successful key messages for the community to ensure the acceptability of future *Wolbachia* releases [39]. A similar approach was used to raise community awareness and answer concerns in Australia about the deployment of *Wolbachia* infected mosquitoes and resulted in high levels of community support [20]. It is important for vector control programs that want to implement *Wolbachia* technologies, to assess the perceptions of the community to later develop educational materials according to their needs, inform the community and achieve better public acceptance.

Most residents supported methods that are familiar to them like source reduction, IRS, and TMLS because they personally have carried them out or know the techniques used to apply them. For example, source reduction has been promoted historically in dengue educational campaigns [12, 13], and individuals commonly use insecticides inside their homes. The government also uses truck mounted insecticide spraying for adult mosquitoes, so it is familiar to residents [39]. Still, residents know that these methods require continuous application, community-wide participation, and commitment from the community to be effective. They also know that mosquitoes will return once the government stops spraying insecticide. At the time of our study, we did not find research regarding community acceptance for TMLS. Our results may help other vector control programs that are considering using TMLS understand community perceptions on its use.

Another important factor for community support was the methods’ safety for humans, animals, and the environment. Participants noted advantages of methods that did not affect bees or butterflies and saw disadvantages in those that affect human health. For example, although participants were not familiar with the AGO, larvicide application, *Wolbachia-*based methods, and GMM, they supported these vector control methods because they perceived them to be harmless to humans, animals, or the environment. Residents who supported GMM in Key West, Florida also preferred GMM over chemicals and spraying [38]. If the method (i.e., IRS, TMLS) is perceived to affect the respiratory system, participants who experience asthma either would not be supportive of the method or would need to be advised of precautions they should take to reduce risk of adverse effects.

For laboratory manipulated mosquitoes (e.g., mosquitoes with *Wolbachia*, GMM), some participants feared *Wolbachia* would be injected into humans through mosquito bites, or believed that instead of *Wolbachia*, laboratories would insert another organism or chemical in the mosquitoes. Other studies have found that concerns about safety to humans and the environment with the implementation of unfamiliar methods such as *Wolbachia* or GMM were common [21, 23, 33, 43]. This suggests the need to provide salient information about the safety of vector control methods. As done in *Eliminate Dengue* in Australia, presenting clear information about the procedures used to produce mosquitoes with *Wolbachia* and GMM would improve residents’ trust towards the technique as well [35].

Participants also indicated that the cost to individuals or the government would influence community support, determining the sustainability of the method over time [44]. People who live in Puerto Rico are aware of the current government economic deficit, and participants understood that for most of the presented methods, if the method stops being used continuously, mosquitoes will come back. Methods like the AGO and IRS would receive more support if the service were free of cost to residents. Similar studies found challenges for individuals in implementing vector control methods. One example was in a malaria prevention study in Uganda where individuals lacked resources to pay for vector control costs and had little free time to implement control methods at a household level (i.e., closing doors and windows early and removing standing water where mosquitoes lay eggs) [45]. A study in Cambodia also found that residents wanted to have continued vector control product access after the project was over [46]. From our literature search, we concluded that more studies needed to be carried out on the use of AGOs. Results from our study help understand perceived barriers on AGO maintenance and sustainability in large communities.

In addition, our study indicated that religious values may have an influence over the support of an emergent method. For example, people may think genetic modification is against divine or natural processes and not support GMM. In Brazil, communities’ religious values were taken into consideration prior to the use of GMM [47]. However, our study participants did not cite religious values as a reason for not supporting GMM. Our study participants supported GMM more than *Wolbachia* replacement because with the latter they were concerned about continued nuisance mosquito bites.

Additional information on how vector control methods are implemented and how effective these methods are in other countries, including the continental United States, was also important for participants’ support. This was particularly important to participants regarding emergent vector control methods, such as *Wolbachia* suppression, *Wolbachia* replacement, GMM, and TMLS. Lack of information (i.e., technique, product, and adverse effects) would generate distrust towards the method and the government. For example, during the 2016–2017 Zika outbreak in PR, public opposition and resistance halted the use of aircraft for aerial spraying with Naled (Dibrom), an organophosphate, due to a lack of timely information about its use prior to attempted implementation [48]. The public was most concerned about adverse effects on bees and frogs, as well as adverse health effects on humans. In addition, there were concerns related to the history of experimentation with chemicals like Agent Orange and hormonal oral contraception in PR [49–54]. Thus, for our study participants, knowledge of emergent vector control methods and their effectiveness and implementation in other countries [38] are necessary components for community acceptance in Puerto Rico. This information is vital when developing educational materials for emergent vector control methods in Puerto Rico and communities in other countries with similar information needs.

Messages and materials about the vector control methods were distributed to the community using the strategies suggested by the group discussion participants: house-to-house visits with distribution of newsletters, flyers, and dissemination of information in health fairs, school visits, and through traditional and social media. McNaughton (2012) argues: “citizens most affected by the use or trial of new disease control methods should be engaged early and given access to culturally appropriate, understandable and accessible information from which they can decide how they want the disease to be managed and whether to support a new initiative.” The COPA project carried out timely promotional campaigns and used consistent messaging, addressing community members questions, and keeping residents informed about scheduled activities. This information was vital for gaining buy-in and community trust. Involving the community and educating them about emergent methods, addressing their concerns, and ensuring the government defrays costs and provides resources will allow for more opportunities for community acceptance of emergent vector control methods [20, 24, 33, 35, 45].

This study has some limitations. As a qualitative study, the results are not generalizable to a global population; however, results are likely to have commonalities for other community members where the *Ae*. *aegypti* vector lives. In addition, participants heard the descriptions of these emergent control methods for the first time. If participants had received the slide set prior to the group discussions, results might have been different. To build a stronger consensus for the use of a novel vector control method, the COPA project educated the community based on the group discussions and continuously surveyed residents in COPA clusters.

## Conclusions

Information, knowledge, and understanding were crucial to obtaining support of vector control methods. The vector control methods commonly used in Puerto Rico (source reduction, IRS) or the use a familiar technique (TMLS), received the most support from participants. Methods perceived to not harm humans, animals, or the environment (AGO, larvicide application, *Wolbachia* suppression and GMM) also received support. The latter two received the support of more than half of the participants although this was the first time that they had heard about these vector control methods. Participants did note that they would need more information to support these methods fully. Regarding public health policies, vector control program leaders need to evaluate and consider if emergent vector control methods are sustainable beyond the implementation of pilot projects in Puerto Rico, and in other countries. They also need to measure if the reduction in the number of mosquitos counterbalances the emergent method’s implementation cost.

## Supporting information

S1 Appendix Vector control methods’ descriptions and illustrations used to guide discussions groups. COPA, 2017, Ponce, Puerto Rico.
(DOCX)

S2 Appendix Anonymized transcripts of group discussions (Spanish). (ZIP)

S3 Appendix Community outreach activities conducted as part of the COPA (communities organized to prevent arboviruses) project. Ponce, Puerto Rico, 2020–2021.
(DOCX)
