# Supplementary material for: Acceptability of emergent Aedes aegypti vector control methods in Ponce, Puerto Rico: A qualitative assessment
Source: PLOS Glob Public Health. 2024 Mar 6;4(3):e0002744. doi: 10.1371/journal.pgph.0002744 (PMC10917327; doi:10.1371/journal.pgph.0002744)
Supplement: S1 Appendix — COPA, 2017, Ponce, Puerto Rico. (DOCX) [file pgph.0002744.s001.docx]

**S1 Appendix. Vector Control Methods’ Descriptions and illustrations used to guide discussions groups. COPA, 2017, Ponce, Puerto Rico.**

| **Vector Control Methods** | **Descriptions** |
| --- | --- |
| **Source Reduction**  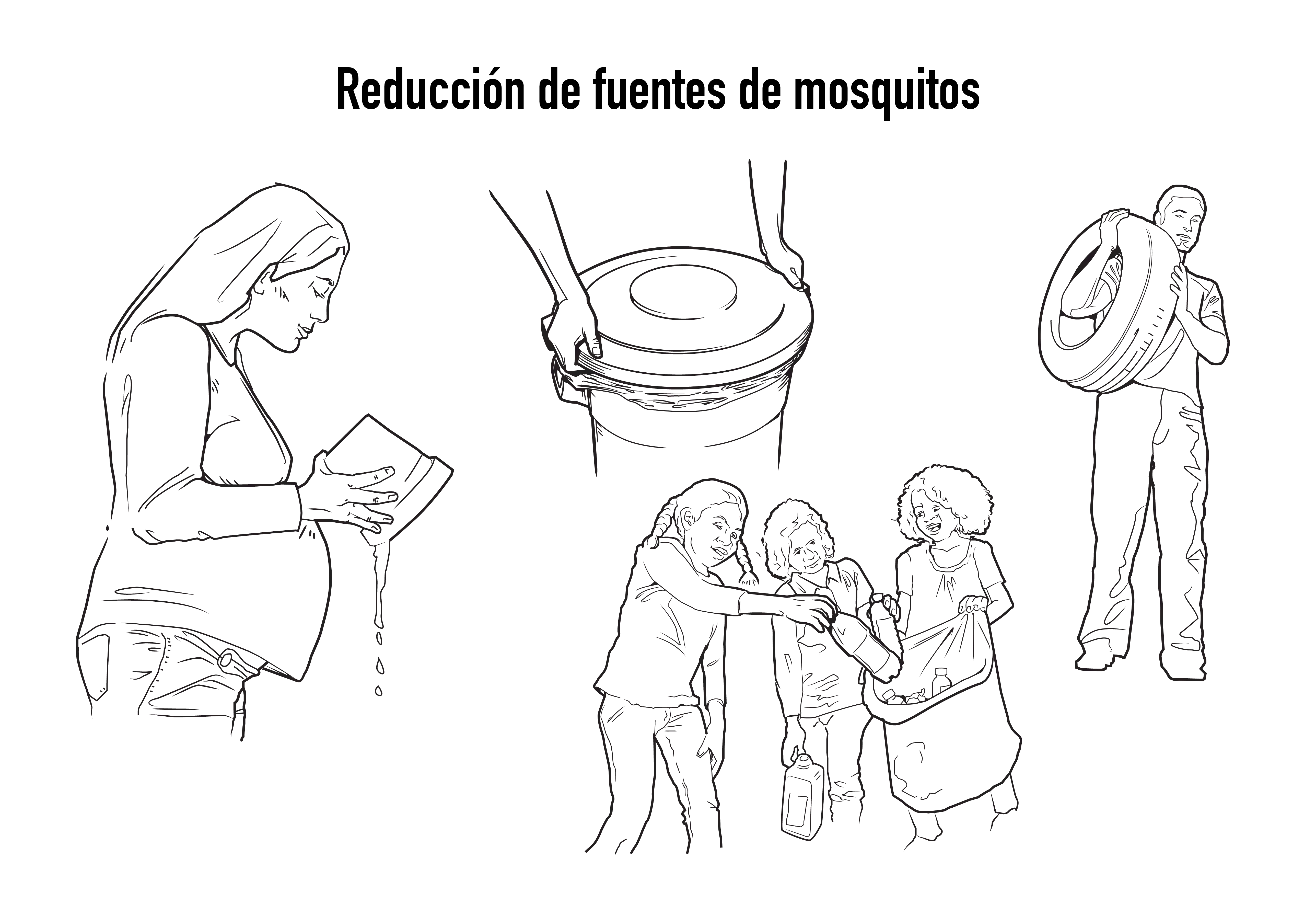 | - Source reduction is the elimination of places where mosquitoes lay their eggs. - The municipality and its community would work together to eliminate, empty, and collect the containers with accumulated water that could be breeding sites for mosquitoes in public areas. - Inside your home and yard, you would cover, empty, or dispose of containers that collect water such as flowerpots, cans, tires, and drains. |
| **Application of Larvicide**  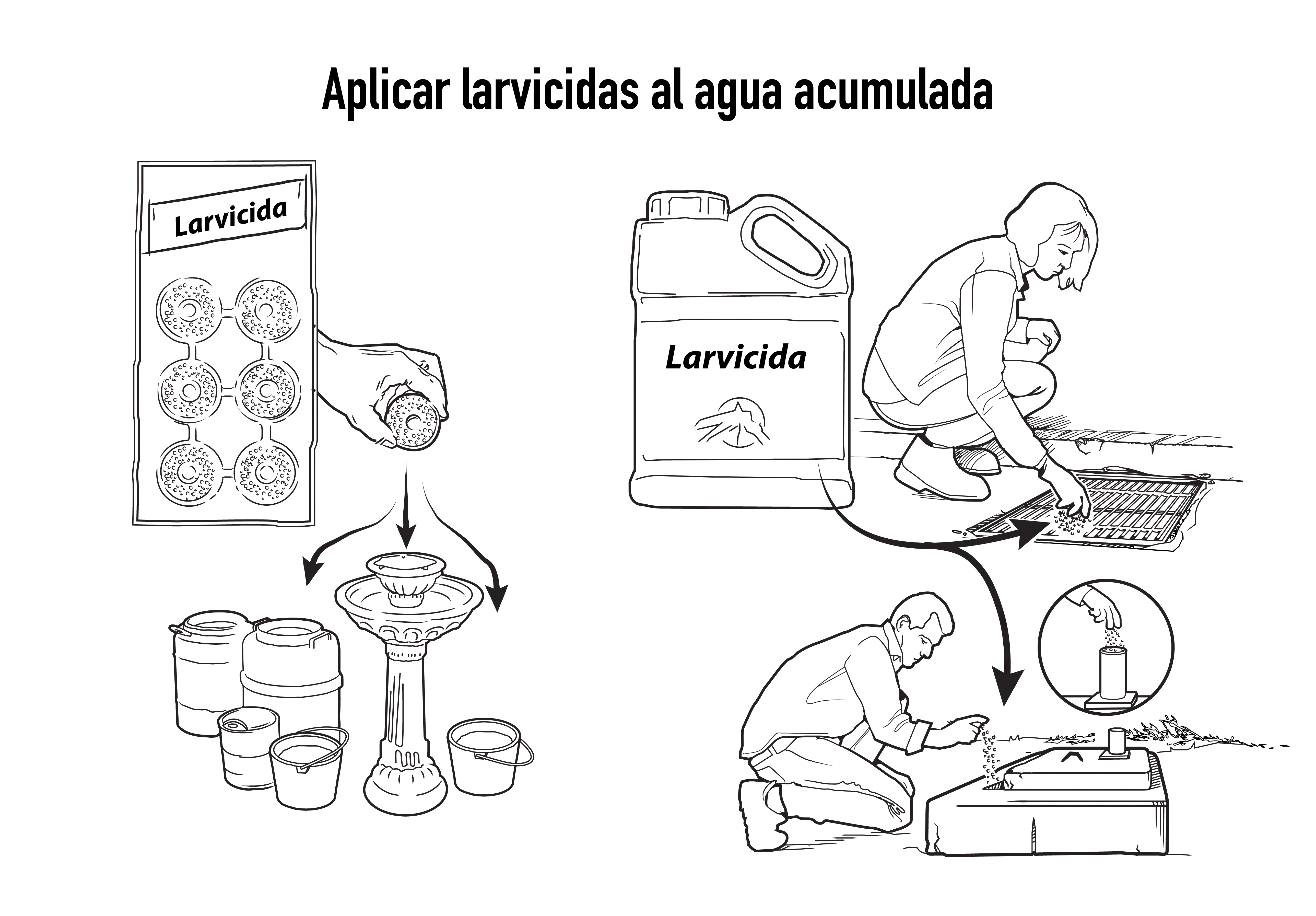 | - Larvicides are pesticides that are used to kill larvae before they develop into adult mosquitoes. - Larvicides can be applied in different ways: in granules, tablets, or liquid. - The application of larvicides can reduce the number of mosquitoes if applied correctly. Larvicides do not affect people, their pets, or the environment if the instructions on the label are followed. - Larvicides should not be used in drinking water for human or animal consumption. - It is required to apply the correct amount according to the directions on the label. - It is required to be replicated from time to time. - It does not reach hidden places where mosquitoes breed. |
| **Truck-mounted Larvicide Spraying**  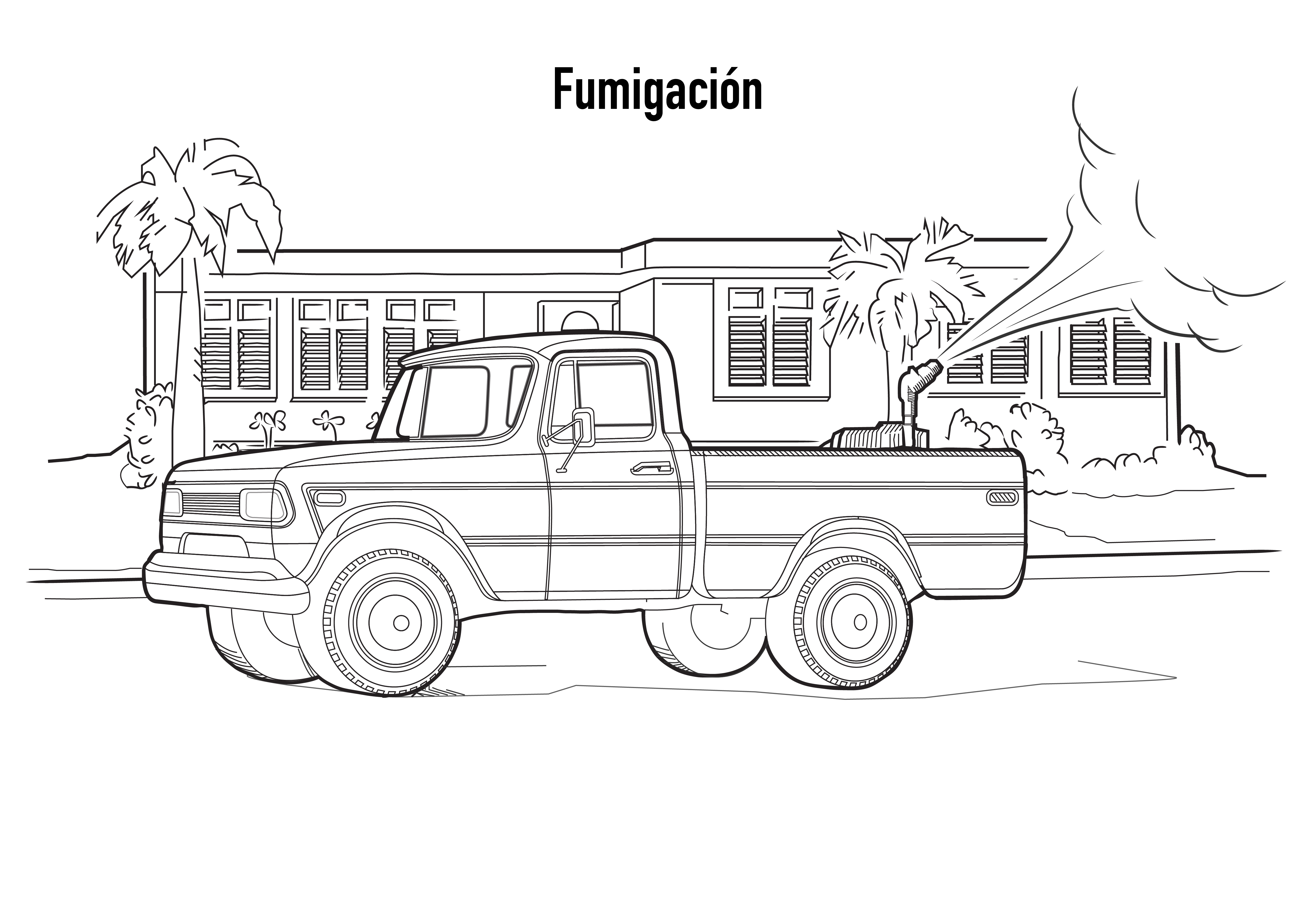 | - Larvicides can be applied in different ways, but many mosquito controls programs have found that applying larvicides from a truck can be effective in reaching most places where larvae are found. - The larvicide is sprayed from a truck on buildings and vegetation, properties, and land, etc. - Larvicides must be reapplied regularly. |
| **Indoor Residual Insecticide Spraying**  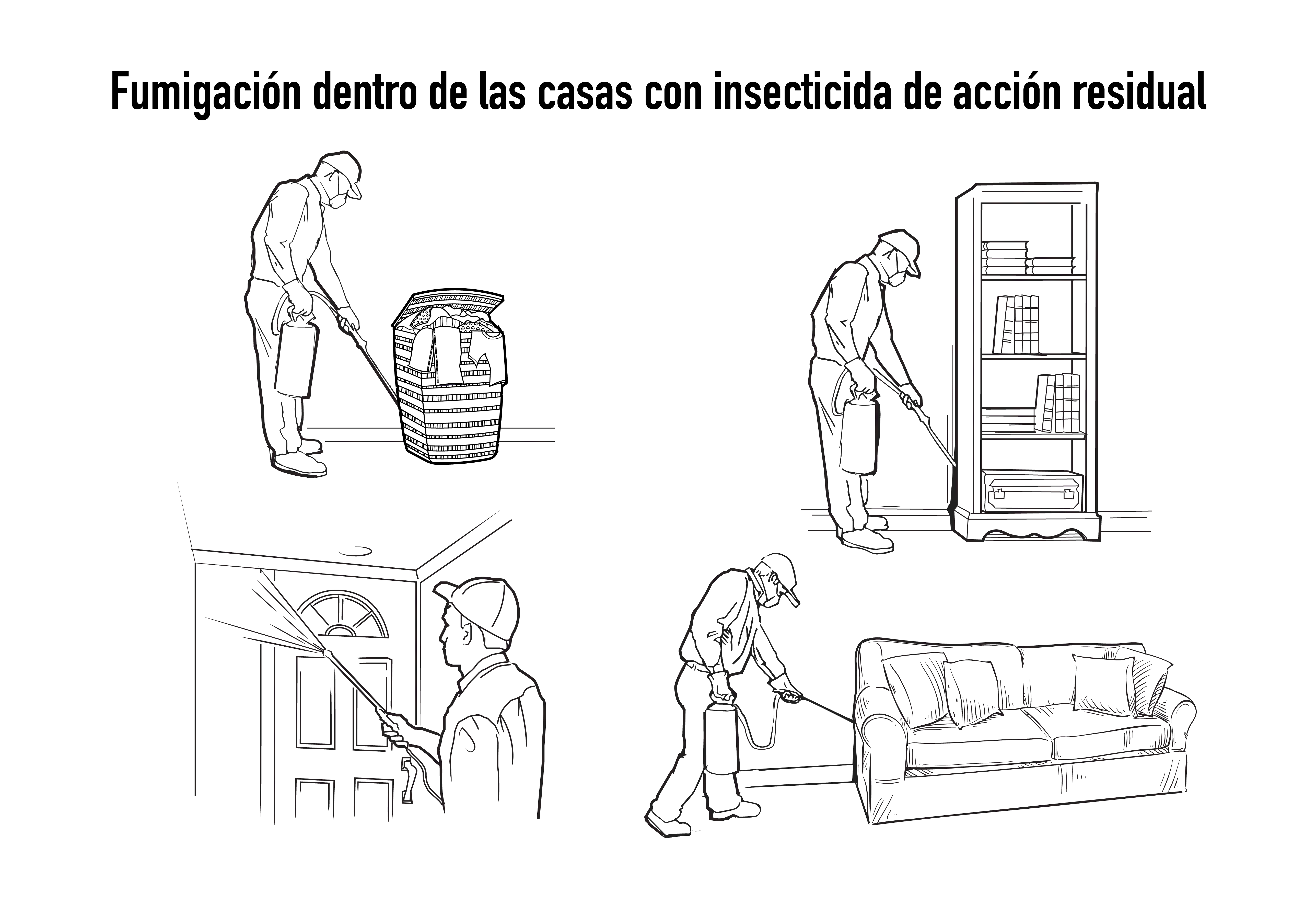 | - Indoor residual insecticide spraying is a mosquito control method where a trained professional treats the interior of your home with an insecticide. - It consists of spraying the walls and other surfaces of a house with an insecticide that continues to work for several months. - It kills mosquitoes that land on surfaces.   that have been sprayed with the insecticide.   - This type of fumigation has been used in many countries around the world including Puerto Rico and the United States. - It can be effective in reducing the number of mosquitoes if applied to a large number of houses in an area. - After application there may be an odor for a few hours, but it is unlikely to cause harm to people when done correctly. - Requires permission and availability of - resident to enter the house to fumigate. - Repeated use over time can make mosquitoes resistant to the insecticides. - This spraying should be repeated to keep mosquito populations low. |
| **AGO traps**  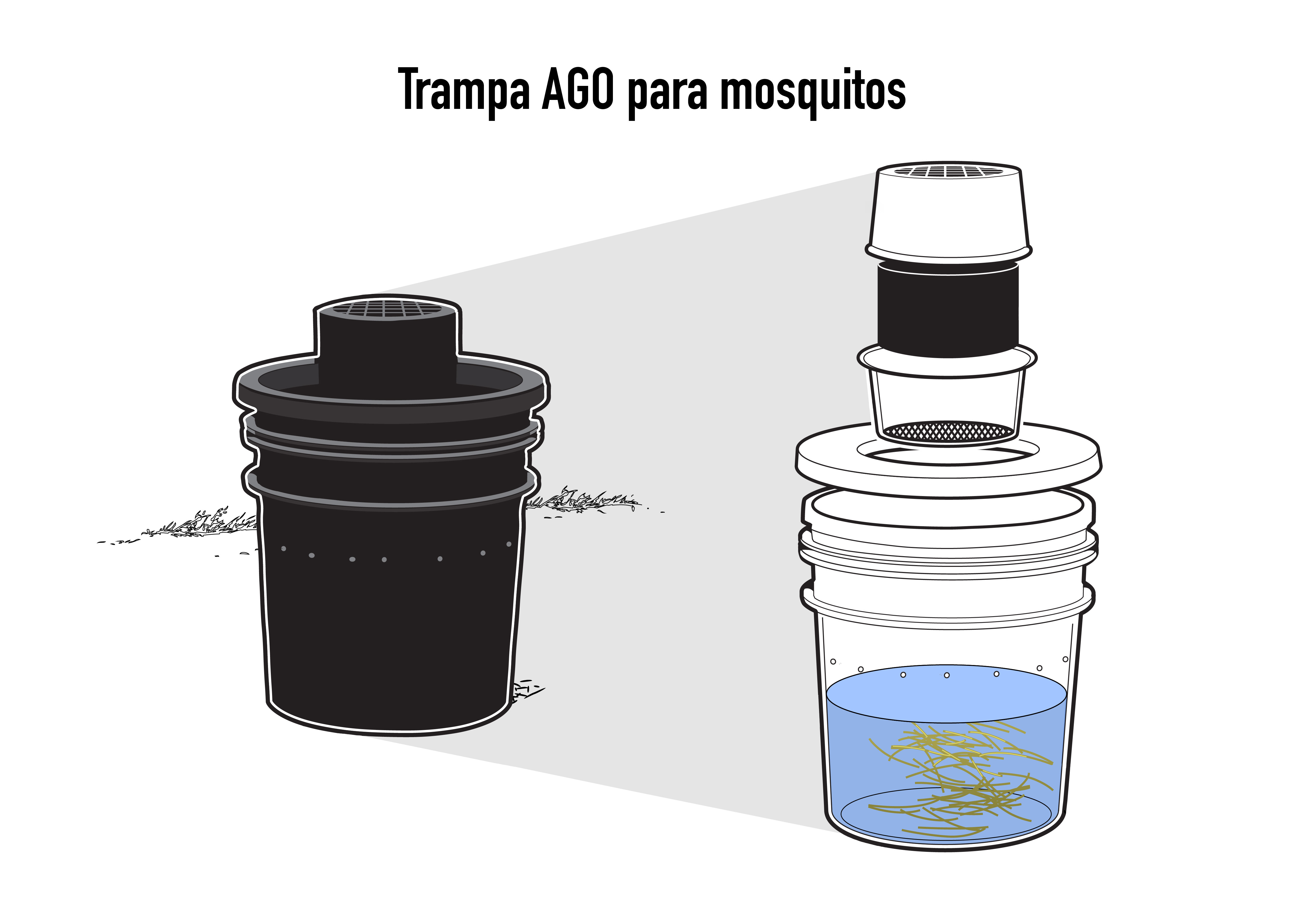 | - The AGO mosquito trap has been used in Puerto Rico to reduce the number of mosquitoes. - The trap attracts and captures female *Aedes aegypti* mosquitoes looking for containers to lay their eggs. - The trap consists of a 5-gallon black plastic bucket, a capture chamber, and a screen. - The bucket is half full of water and hay to attract female mosquitoes. - Inside the capture chamber there is a paper with a special glue that catches the mosquitoes when they enter to lay their eggs. - The trap contains organic material, so it can smell. - No specialized training is required to set the trap. - The trap requires maintenance every 2 months so that it does not become a breeding ground for mosquitoes. - The trap reduces mosquito numbers if properly maintained and used in 8 out of 10 households in the community. |
| ***Wolbachia* Replacement**  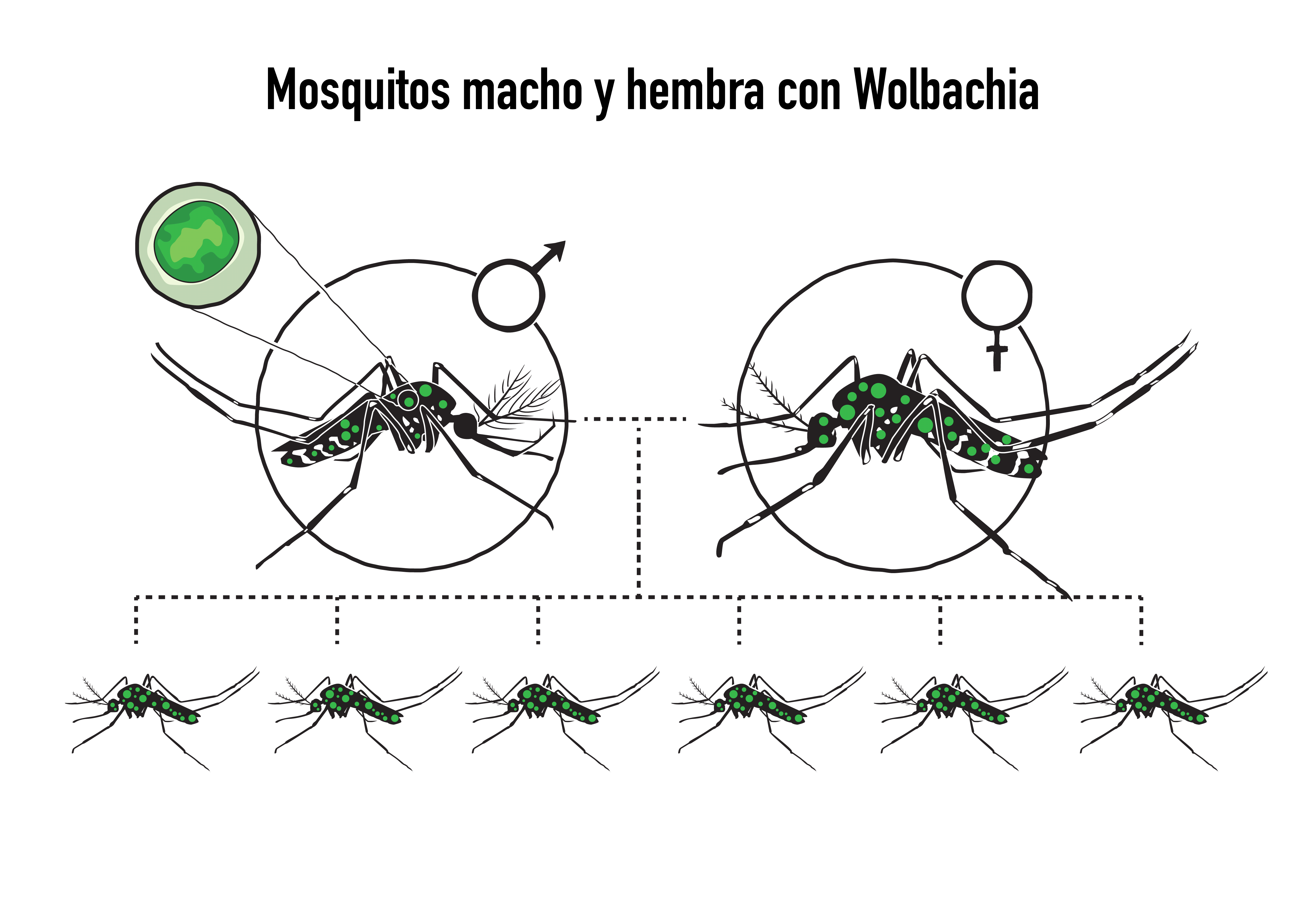 | - *Wolbachia* mosquitoes work in two different ways: - In the first, male and female *Aedes Aegypti* mosquitoes are released with *Wolbachia*. - When the female mosquito with *Wolbachia* breeds with a male mosquito with or without *Wolbachia*,   the bacterium is passed through the female mosquito generation to generation.   - Over time, the number of mosquitoes with *Wolbachia* increases and replaces the mosquitoes of the environment without the bacteria. - After releasing them several times, the mosquito population with *Wolbachia* will be maintained without releasing more of   these mosquitoes.   - Mosquitoes with *Wolbachia* are less capable of transmitting diseases. - There will still be mosquitoes in the community because the intention of this method is not reducing the number of mosquitoes, but to reduce the risk of epidemics. - However, mosquito bites will not be reduced. - This activity has been used in other countries such as Colombia and Brazil. - Currently, there are no defined rules for the use of male and female *Wolbachia* mosquitoes in the United States. |
| ***Wolbachia* Suppression**  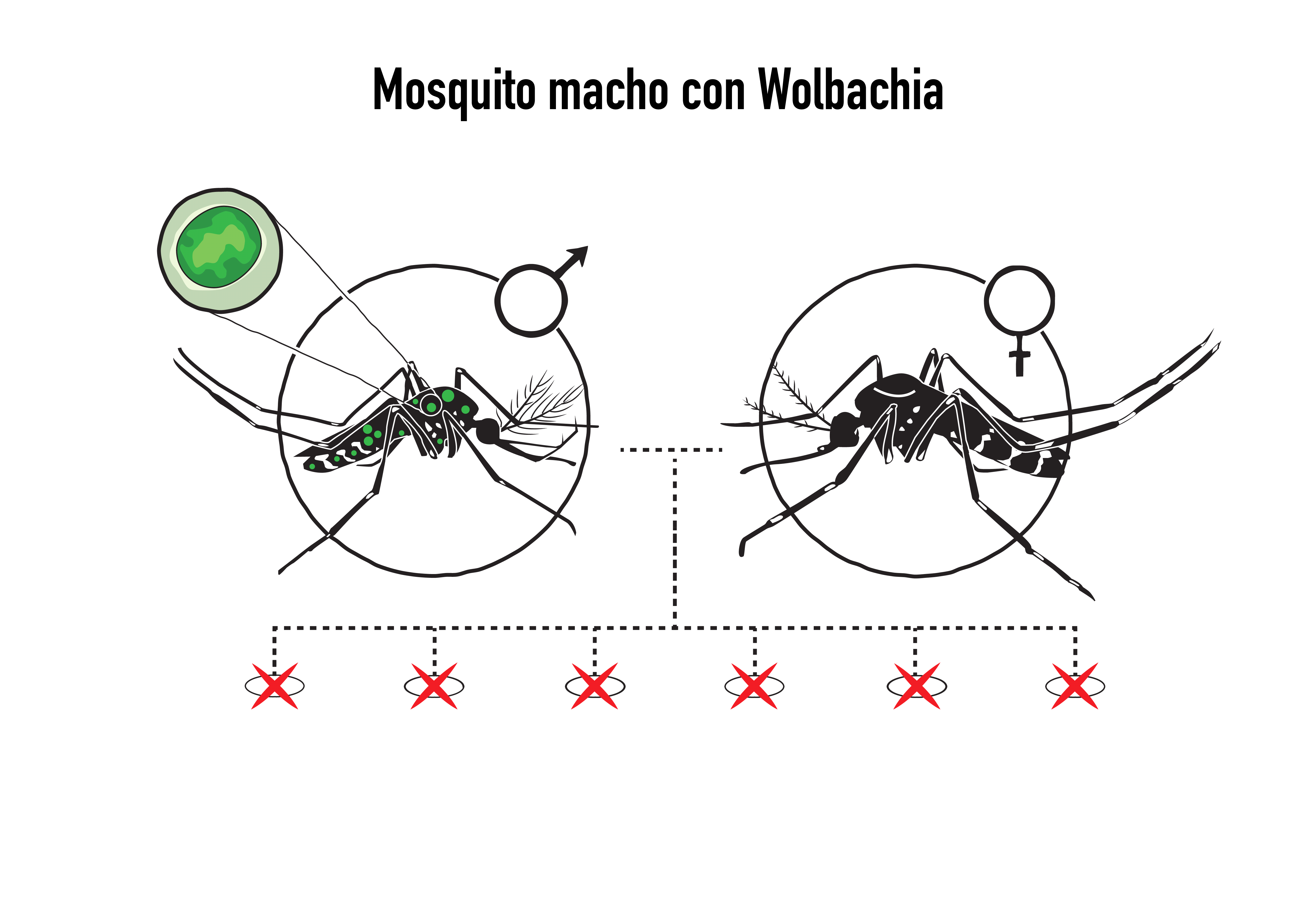 | - In the second way of using this activity, only male mosquitoes are released with *Wolbachia*. - Male mosquitoes are released into the environment. - They do not bite or transmit disease. - Male mosquitoes with *Wolbachia* mate with females without *Wolbachia*. - Females without *Wolbachia* lay their eggs, but these do not hatch. - Mosquitoes with *Wolbachia* should continuously be released in large quantities to keep mosquito populations low. - Once mosquitoes with *Wolbachia* stop being released in an area, the mosquito population will increase again. - Male mosquitoes with *Wolbachia* have been used in studies in California and in the Florida Keys and have been approved for evaluation in Miami, Florida. |
| **Genetically Modified Mosquitoes**  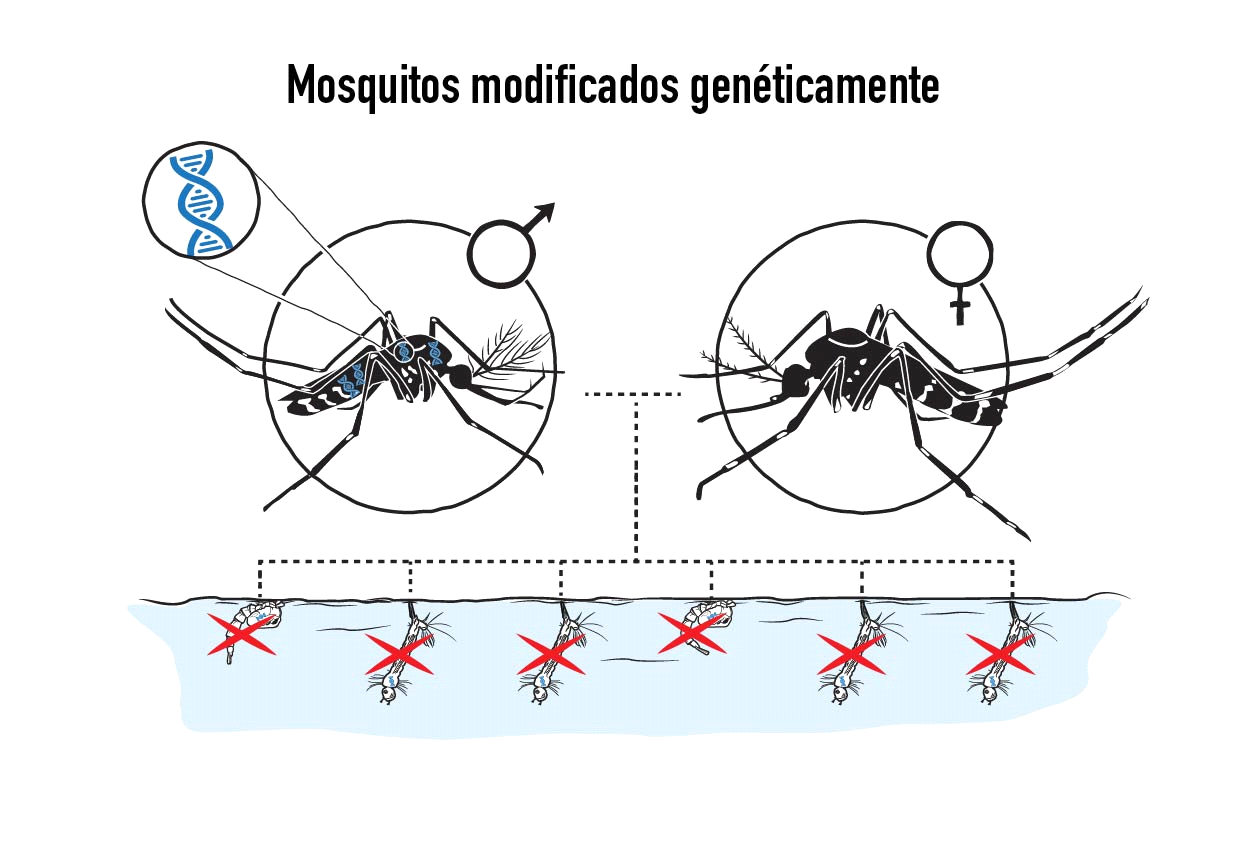 | - Male genetically modified mosquitoes are released, to join with female mosquitoes in the environment. - Genetically modified male mosquitoes reproduce with females in the environment, passing a gene to their offspring that prevents larvae and pupae from developing normally. Thus, they die before becoming adult mosquitoes. - Released male mosquitoes do not bite or transmit disease. - These mosquitoes must be released several times a week over time and in large quantities to keep low *Aedes aegypti* mosquito populations only. - Once the genetically modified mosquitoes *Aedes aegypti* cease to be released in an area, the mosquito population will increase again. - Genetically modified mosquitoes have been evaluated in different countries, including the Cayman Islands, Brazil, and Panama. - At present, there have been no studies of genetically modified mosquitoes in the United States. |
