# Supplementary material for: Acceptability of emergent Aedes aegypti vector control methods in Ponce, Puerto Rico: A qualitative assessment
Source: PLOS Glob Public Health. 2024 Mar 6;4(3):e0002744. doi: 10.1371/journal.pgph.0002744 (PMC10917327; doi:10.1371/journal.pgph.0002744)
Supplement: S2 Appendix — (ZIP) [file pgph.0002744.s002.zip › S2C_Appendix.docx]

**S2 Appendix. Anonymized Transcripts of Group Discussions (Spanish)**

**COPA Grupo de Discusión con Líderes y Residentes sobre Actividades de Control de Vectores en los Clústeres VG, PO, JDC y TU**

Fecha y hora- 25 de abril del 2018; 6pm

Lugar- Centro Comunal VG

Moderador- Carmen Pérez

Anotadores- Coral Rosado y José Robles

Transcriptor- Sue Ramos

Participante #1- TU (M) - Uno

Participante #2- TU (M) - Dos

Participante #3- TU (M) – Tres

Participante #4- VG (M) - Cuatro

Participante #5- PO (M) - Cinco

Participante #6- PO (M) - Seis

Participante #7- PO (H) - Siete

Participante #8- VG (M) - Ocho

Participante #9- VG (H) – Nueve

Participante #10- VG (M) - Diez

Participante #11- PO (H) - Once

Comentario- En este grupo de discusión los participantes, ni el moderador desarrollaron la dinámica de decir su número antes de hablar, y en un punto llegaron a ser once participantes. Traté lo mejor que pude de asignar un seudónimo a cada voz que escuchaba tratando de diferenciar con la posición de la grabadora y las notas de los anotadores, pero no siempre estoy tan segura si asigné el número correcto al participante correcto todo el tiempo. Otro factor que ha hecho difícil seguir quién decía qué, es el hecho de que, al llegar nuevos participantes, los participantes ya en el grupo se movieron de posición, haciéndome perder nuevamente la identificación de las voces por su posición. La información sobre a qué comunidad pertenecían, para los participantes 8,9,10 y 11 vino de las notas de los anotadores.

Introducción- Se comenzó leyendo el consentimiento informado con los participantes. Todos confirmaron ser mayores de 21 años. Se les preguntó a los participantes si tenían preguntas sobre lo leído. Todos afirmaron no tener preguntas. Y se les preguntó si deseaban participar de la discusión. Todos los presentes hasta el momento afirmaron participar. Se les asignó número a cada participante que estaba presente al momento de comenzar.

Moderadora

Les voy a asignar los números y me van a decir de la comunidad que son, para que él los pueda anotar. O sea… solamente… yo voy a decir número uno… ay bendito, si me deja. Número uno, número dos… ¿Número uno (*Uno*) de qué comunidad?

Uno

El TU.

Moderadora

¿Número dos (*Dos*)

Dos

El TU

Uno

Sector NV.

Dos

Sector NV.

Coral

¿Una y dos el TU?

Uno

El TU y ella el TU, pero sector NV.

Moderadora

¿Número tres (*Tres*)?

Tres

TU.

Moderadora

Número cuatro (*Cuatro*)… ¿De qué comunidad…?

Cuatro

VG.

Moderadora

VG. ¿El cinco? El cinco, ¿de qué comunidad…?

Cinco

PO.

Moderadora

El seis.

Seis

PO.

Moderadora

PO. ¿Y el siete?

Siete

PO.

Moderadora

PO también. Gracias y así, eso los va a ayudar a ellos, como no van a decir sus nombres, eso les va a ayudar a ellos a… cuando ustedes hablen, apuntar el número de quién lo dijo. Ok y entonces, habiendo dicho eso, ahora empezamos.

Parte 1- Conocimiento sobre enfermedades transmitidas por mosquitos

Pregunta 1- ¿Qué ha escuchado sobre el dengue, Zika y Chikunguña?

Moderadora

Ok, yo les voy a hacer una primera pregunta y después les vamos a enseñar una… pre… vamos a ir sobre unos dibujos y ustedes me van a ir contestando las mismas preguntas para cada dibujo. Pero antes de eso, les voy a hacer una pregunta. ¿Qué han escuchado ustedes, sobre el dengue, el Zika y el Chikunguña? ¿Cómo se transmiten y cuán peligrosas son?

Cuatro

Se transmite por el mosquito, este, pueden causar la muerte, aunque tienen condiciones similares. Unas tienen un *rash*, otras pueden tener un derrame, o sea, que son de sangre. Se pueden adquirir por la humedad que hay en los patios, las aguas empozadas, donde hay mucha hierba que no se corta.

Moderadora

Ok. Muy bien.

Seis

El Zika tengo entendido, que se transmite también sexual.

Moderadora

Sí. Sí. Eso es la diferencia, que también se puede transmitir sexual. Y tú eres el número seis. Ok. Ajá.

Tres

Cuando a mí me dio el Chikunguña, ese día, ja, antes… la noche antes, o sea, el día antes que amaneciera enferma, yo vi ese moquito que venía. Yo estaba abajo y yo estuve más de un mes, desde que empezaron las… cómo se dice, el hablar de estas cosas.

Uno

La promoción y eso…

Tres

Sí. Yo estuve con pantalón largo, tenis, manga larga, me ponía todos los *sprays* para los mosquitos. Y ese día antes, no me puse nada. Me bañé y me fui para el patio a deshierbar y después me siento a descansar, cuando veo ese mosquito, bien grande, y vino y para, y de que me tocó… que yo lo vi que se pegó, yo lo maté. Pero que no me limpié. Y al otro día, yo dije, ‘Ay, Dios…’, pero no fui y me lavé. Y al otro día amanecí que no podía caminar. El dolor en los talones era tan horrible y en la… en los huesos de las piernas, que yo no podía caminar. Y pasé así, un mes. Con fiebre, no me dio vómitos, pero me dio una fiebre… horrible, horrible y unos dolores en todo el cuerpo.

Moderadora

Ay bendito. Sí, ese es bien terrible a mi hija también le dio. ¿Alguien tiene algo más que decir?

Seis

El dengue tiene dos clasificaciones. Está el hemorrágico y el otro es… no sé, algo ahí que te baja las plaquetas.

Moderadora

Ajá. Ok. Ok. ¿Algo más?

Pregunta 1a- ¿Cómo se transmiten?

[se contestó más arriba]

Pregunta 1b- ¿Cuán peligrosas son?

[se contestó más arriba]

Parte 2- Reacciones a las actividades de control de vectores

Moderadora

Ok, bueno, pues entonces, para comenzar les voy a mostrar un video que explica el ciclo de vida del mosquito *Aedes aegypti*. Esta explicación le ayudará a entender mejor cómo funcionan las actividades para el control de mosquitos. Ok. Así que vamos para allá con el… Empezamos diciendo que el mosquito *Aedes aegypti* macho no pica, pero la hembra pica, y está picando muy bien ahí, verdad, a una persona para obtener sangre y producir sus huevos. El macho no pica, pero la hembra pica y chupa sangre para poder producir sus huevos. Mira cómo se le va llenando la barriguita de sangre según pica. ¿La ven? ¿La pueden ver desde ahí?

Seis

Sí.

Moderadora

Y entonces, el mosquito hembra pone sus huevos en las paredes de los envases con agua acumulada, dentro o fuera del hogar. Esos huevos pueden durar hasta ocho meses pegados a las paredes del envase. Cuando los huevos se sumergen en el agua, verdad, ahí van a nacer las larvas. ¿La ven? Que está naciendo. Está saliendo del cascarón del huevito, está naciendo la larva. Como todos los bebés, es bien bonita, pero después se pone peligrosa. Las larvas viven en el agua donde se alimentan y se convierten en pupa en aproximadamente cinco días. Aquí está la larva que ya creció, verdad, crecen cinco… en aproximadamente cinco días y eso se llama el sifón, y eso es para ella respirar oxígeno, ve. Y entonces luego, en aproximadamente cinco días se convierten en pupas. Ya en pupa, a los dos o tres días se convierten en mosquitos adultos. Míralo como está saliendo de la pupa. ¿Ven cómo va saliendo de la pupa? Muchacho sal ya. [risas] Ahí salió, necesitaba ese empujón. Se toma de siete a diez días desde que un huevo de mosquito se convierte en un mosquito adulto. Desde que el huevo se convierte en mosquito adulto, todo ese ciclo que vimos tarda de siete a diez días. Por eso es por lo que les recomendamos que vaya semanalmente a su patio a virar todos los envases y limpiar todos los envases que tenga con agua, porque de esa forma rompemos… si vamos semanalmente, rompemos el ciclo de vida del mosquito. Ok. Ahora, se están desarrollando muchas formas de reducir el número de mosquitos en el ambiente. Algunas acciones o métodos pueden ser realizadas por los residentes y las comunidades, y otras pueden ser realizadas por profesionales de control de mosquitos del municipio. Ahora les mostraré unos dibujos que describen las actividades que se podrían realizar y cómo podrán ayudar. Les voy a hacer una serie de preguntas para obtener su opinión sobre esta actividad o método. Ok, vamos por la primera.

Actividad #1 – Reducción de fuentes de mosquitos

Moderadora

La primera sería reducción de fuentes de mosquitos. La reducción de fuentes es la eliminación de lugares donde los mosquitos ponen sus huevos. El municipio y su comunidad trabajarían juntos para eliminar, vaciar y recoger los envases con agua acumulada que pueden ser criaderos de mosquitos en áreas públicas. Dentro de su hogar y patio, usted cubriría, vaciaría o eliminaría los envases que acumulan agua como los tiestos, latas, gomas y desagües.

Pregunta 2- ¿Es esta una actividad nueva para usted o es algo que ya había escuchado antes? Si la escuchó, ¿dónde la escuchó?

Moderadora

Les pregunto. ¿Habían escuchado ustedes antes sobre esta actividad? Sobre este método.

Seis

Sí.

Uno

Sí, ese es el más común.

Dos

Sí…

Moderadora

Ese es el más común. ¿Verdad? Ok.

Uno

Que no lo hacemos es otra cosa.

Moderadora

O… exacto, que no se haga es otra cosa. ¿Dónde lo escuchó? ¿Dónde lo habían escuchado ustedes?

Tres

En la televisión.

Cuatro

Radio.

Moderadora

En la televisión. ¿Dónde más?

Cuatro

Periódico.

Moderadora

¿En internet?

Cuatro En la radio.

Seis

Periódico, noticias. En las escuelas.

Moderadora

En las escuelas, o sea, que esto se ha corrido por todos los medios, verdad. Ok.

Pregunta 3- ¿Cree que esta actividad reduciría el número de mosquitos en su comunidad? ¿Por qué?

Moderadora

Y entonces, ¿Cree usted que esta actividad reduciría el número de mosquitos en su comunidad?

Tres

Sí.

Uno

No.

Cuatro

Ayuda, pero no lo reduce.

Moderadora

Ayuda, pero no lo reduce. Aquí mi compañera está diciendo que no. ¿Por qué no?

Uno

Porque nada más no es…. Este, por virar los envases. Estamos hablando de la hierba, los mosquitos se quedan en la hierba. Todo lo que sea general. O sea, una cosa es que tú limpies tu casa y que el vecino no limpie la de él, ya tú limpias tu casa, pero los mosquitos del vecino pasan para tu casa a picarte. Tú sabes, que, eso no es… no es… no.

Dos

Hay que tener control.

Moderadora

Y entonces, usted estaba diciendo ¿qué?

Cuatro

Ayuda, pero no lo resuelve.

Moderadora

Ayuda porque no…. Pero no lo resuelve. ¿Por qué?

Cuatro

Ayuda, porque es como ella dice, tú mantienes tu área más o menos en condición, pero si el vecino o si hay este pasto tan a la derriba como uno dice…

Uno

Es difícil.

Cuatro

… entonces, además, este… a veces uno no encuentra o no tiene conocimiento, vamos a decirle, no que, si no encuentra, no tiene conocimiento de qué agencias me pueden ayudar en situaciones así difíciles que tú tengas un solar vacío, baldío al lado que nadie se haga cargo de limpiarlo, en tu vecino que son de los que recogen y echan a un lado… entonces, si no tienes conocimiento, pues, entonces tú tienes que luchar sola.

Moderadora

¿Y los que dijeron que sí? ¿Por qué ustedes creen que esto sí ayudaría a reducir el número de mosquitos en su comunidad?

Tres

Bueno si… perdón, si nosotros también lo hacemos, nosotros tenemos que llevar esa… ese conocimiento a los demás, para que entre todos se pueda hacer algo.

Moderadora

O sea, que es una cosa, según lo que yo estoy entendiendo que usted dice, que es algo que cada persona tiene que hacer en su casa, pero también lo tiene que hacer la comunidad…

Cuatro

Exacto.

Seis

Exacto…

Moderadora

…verdad, en los lugares quizás públicos.

Uno

Es como una cadena.

Moderadora

Es una cadena.

Uno

Eso es como un eslabón, tú sabes, yo aprendo, tú aprendes, yo aprendo. Lo que pasa es que el ser humano es bien complicadito. Porque, entonces, hay muchas personas que sí lo hacen, pero hay muchas personas como que no, no les importa. *Hello*…

Cinco

Que no lo hacen, que conviven… hasta conviven con el mosquito.

Seis

Exacto.

Cinco

Piensan que es parte de la familia.

Uno

Son sus mascotas. Sus mascotas.

Cinco

Exacto.

Tres

Hermana, este, perdón, este mi vecina y yo tenemos el problema…

Dos

Con la vecina…

Tres

…con la vecina de atrás. Yo le estuve hablando, ‘mira, ¿cuándo vas a limpiar eso ahí?’ Y, ¿saben qué me contestó? Ahora, esta vez, porque eso ya es como un disco rayado para ella. Lo que me contestó fue, ‘no, yo lo voy a dejar así hasta el próximo huracán’.

Moderadora

Ah cara.

Tres

Porque ella, la hierba ya no está tapando a nosotras también. ¿De qué vale que nosotras limpiemos y ella atrás no limpia?

Moderadora

Y ella no limpia… claro.

Uno

Y tampoco se deja ayudar, que ese es el otro problema.

Tres

Y no se deja ayudar, tú sabes…

Uno

Que está la persona también que no limpia, pero tampoco se deja ayudar.

Pregunta 3a- ¿Qué beneficios o ventajas tiene esta actividad para usted?

Moderadora

Entonces, ¿qué ventajas o desventajas tiene esta actividad para ustedes?

Tres

Nos trae conocimiento.

Uno

Es cansona.

Moderadora

O sea, el conocimiento… esta actividad no, este método, este método.

Uno

Es cansón.

Moderadora

Es cansón. Esa es la desventaja.

Uno

Nada más de levantar uno, una goma de esas que se llena de agua eso es un stress.

Dos

Un balde lleno de agua…

Uno

Un balde de agua, las piscinas que las dejan llenas, tú sabes, es bien cuesta arriba. Es cansón…

Dos

Eso atrae mosquitos.

Moderadora

¿Y beneficios, tiene alguno?

Uno

Bueno me imagino que el beneficio es a limpieza del lugar.

Seis

La reducción… bueno, me imagino que la reducción del mosquito.

Uno

Y la reducción del mosquito.

Moderadora

La reducción del mosquito.

Pregunta 3b- ¿Qué desventajas o dificultades le ve a esta actividad? ¿De qué forma se podrían solucionar esas dificultades?

Moderadora

¿Cómo se podría solucionar, si ustedes le ven alguna solución, a que… a esas dificultades? Que usted me dice que alguna gente lo hace y otras no. ¿Cómo se podría bregar con eso?

Uno

Hipnotizándolos, será. [risas]

Dos

A veces se les llama la atención, y no hacen caso.

Cuatro

Pero bueno, yo iría alguna agencia…

Seis

Bueno, si la comunidad tiene una buena relación, un buen plan de trabajo, esta semana te toca a ti, la otra semana le toca al otro.

Moderadora

Ah, ok, que sería establecer…

Seis

Exacto, un fin de semana uno, un fin de semana otro…

Cuatro

Exacto.

Seis

… y así sucesivamente.

Moderadora

Establecer un plan de trabajo de la comunidad. Y usted decía, si tuvieran una buena relación…

Cuatro

Pero, o sea, un buen plan de trabajo de la comunidad, pero tiene que haber como un supervisor de afuera que haga entender que es nece… por qué se está haciendo y que es necesario hacerlo para mantener la salud en ese lugar. Porque como dice la señora, ese mismo caso, yo tengo. Lo mismo en VG al lado de un camino vecinal, mis vecinos, disfuncionales total. Para comunicarle, tiene que ser por cartita y con eso y con todo… Entonces tú limpias y entonces, eso tiene desde la punta hasta el final, bolsas de basura de cosas que sacan de la casa todos los días sacan… limpian los techos, los bolsos que recogen de las hojas las dejan ahí hasta que el sol las seca, las tuesta, se abren, recorren por todo…. Entonces, como son personas que, hablando en el rasgo popular como yo digo, aquí yo planto bandera y como grito y digo, todo el mundo se retiene. Entonces, cuando tú eres la única persona que les llama… no que les llame la atención, pero que les haces entender que eso está mal pues ya no hay más nada…

Uno

Estás mal. Estás mal.

Cuatro

Mal mira, tú sabes, y es un problema, o sea, es un problema fuerte porque es como yo digo, tú limpias y pues, de nada te vale, porque sales para el patio y tienes que meterte para adentro porque el mosquito te quiere comer. Y es el otro lugar. Entonces, tienen animales, tampoco limpian los patios. Pero, entonces, si tú llevas la situación a esta agencia, esta te pasa aquella, la otra te pasa a la otra, y eso es como una bola en el paño de billar y nadie te resuelve.

Uno

Se pasan a la batuta.

Cuatro

¿Tú sabes?

Uno

O no me corresponde a mí como agencia, le corresponde a fulano. ‘No, no es a mí es al otro fulano’, y yo ‘ah bueno…’

Cuatro

Sí, nada.

Uno

Y ya [no se entiende] a los tres, porque ya como que he pasado a varios y no me han resuelto el problema.

Cuatro

Exacto. Porque si hubiera una persona… un grupo aquí, tú te dirigieras y esa persona como que hiciera, como la fuerza, como yo digo, ‘no, esto vamos a hacerlo, porque usted quiere que esto sea así’, pero… plantamos bandera y se acabó.

Pregunta 4- ¿Cuán posible es realizar esta actividad en su comunidad para reducir el número de mosquitos? ¿Por qué?

Moderadora

¿Cuán posible es realizar esta actividad, este método, en su comunidad para reducir el número de mosquitos? ¿Es posible realizar un tipo de actividad como este, que la gente se reúna y hagan como una limpieza…?

Uno

Es posible. El problema aquí está, que quizás ese día pues la gente coge fiebre y lo hace, pero ahí se acaba todo. Tú sabes, ya al otro día, pues, ya se olvidaron de que había que recoger la basurita, había que recoger la goma, entonces, empiezan otra vez a tirar. Volvemos a lo mismo. A tirar basura de nuevo… que ya se supone que, si ya tú aprendiste a hacerlo, mira, síguelo haciéndolo, pero llega un momento en que no… lo hacen ese día, la fiebre, como que se une todo el mundo, y a lo mejor hay un come lata, hacemos un bochinche… que se yo qué, y todo el mundo, pues, ese día se bota, pero ya los otros días después o pasa un mes y se acabó. Hay que volver a hacer otra comidita.

Pregunta 5- ¿Apoya usted esta actividad en su comunidad? ¿Por qué? (*Preguntar a cada participante del grupo de discusión*)

Moderadora

Ah, ok. [risas] ¿Apoyaría usted esta actividad en su comunidad? ¿Usted la apoyaría?

Seis

Claro

Moderadora

Si el seis es sí.

Uno

Claro.

Moderadora

¿Y tú la apoyarías?

Cinco

Sí.

Moderadora

¿Tú la apoyarías?

Cuatro

Claro.

Moderadora

¿Y usted la apoyaría?

Tres

Claro.

Moderadora

¿Y usted la apoyaría?

Dos

Sí.

Moderadora

¿Y usted?

Siete

[no se escucha en el audio]*

*Anotador tiene en sus notas que todos los participantes apoyan esta actividad.

Pregunta 5a- ¿Piensa que su comunidad apoyaría esta actividad? Sí, No, ¿Por qué?

Moderadora

¿Piensa que su comunidad apoyaría esta actividad?

Siete

No vinieron hoy… [risas] Está difícil.

Moderadora

Difícil. O sea, ese sería el por qué.

Siete

Ajá.

Moderadora

No, porque si no están aquí hoy…

Uno

Es porque no le interesa.

Tres

No hay interés.

Siete

Ese es el primer paso…

Uno

Eso es como en la escuela con los piojos. Siempre había cinco nenas más con piojos, y eran los mismos. O sea, que la mamá… rápido una conferencia, porque la [no se entiende la palabra], porque los nenes y aquello… cinco papás, los mismos, o sea, esos eran los únicos papás que los nenes tenían piojos. Los demás no tienen. Eso es todo lo que te voy a decir, los malos responsables, el resto por vergüenza. Por… ‘ay, es que no quiero que sepan que ellos…’, yo decía, ‘mira, eso no es necesariamente tiene que ser limpieza. Porque tú le peinas la cabeza a tu hija todos los días y lamentablemente eso ya es un germen, eso ya está regado y va a seguir con piojitos. Pero si tú no vas a orientarte, a buscar los métodos, que a veces te regalan champucitos, te regalan estas cosas y otras cosas, pues entonces, no hay chance’. No hay chance. Entonces, empiezan las peleas.

Moderadora

Ay Dios mío y rápido me pica. [risas]

Uno

Sí porque no, porque volvemos a lo mismo, el problema aquí está en nosotros, nosotros los seres humanos, que no transamos, no, no… o nos da bochorno. O sea, no sé, hay otras cosas que se supone que se hacen y se supone que dan bochorno, pero las cosas como son que se yo, como que es bochorno, ay, que no me atrevo, ay que si pasa la gente que la nena tiene piojos… y yo, ‘en serio loca…’ [risas] No es fácil.

Pregunta 6- ¿Qué otra información necesitaría para entender mejor esta actividad?

Moderadora

Ok, y entonces. ¿Qué otra información necesitaría la gente para entender mejor este método? Si alguno…

Uno

Lo más que… lo más cerca es la literatura y la gente la coge y la bota…

Tres

Sí. Se la das y cuando salen la dejan ahí.

Pregunta 7- ¿Considerarían realizar ustedes mismos esta actividad?

Moderadora

Y entonces, ¿considerarían ustedes realizar… ustedes mismos, en sus casas, esta… este método?

Tres

Yo lo hago.

Uno

Yo lo hago.

Seis

Sí…

Moderadora

¿Lo hace?

Tres

Yo lo he tratado muchas veces en mi casa.

Moderadora

Lo hace.

Uno

Y cuando voy por la calle también lo hago. Veo un pote lleno de agua y lo viro.

Moderadora

Ok. ¿Y la número cinco, lo hace? ¿Sí lo hace?

Cinco

¿Relacionado a qué, disculpe?

Moderadora

A que si tú consideraras a hacer esto en tu casa.

Cinco

Sí. Claro. Sí.

Pregunta 8- ¿Qué les haría difícil realizar esta actividad?

Moderadora

Ok. ¿Qué te haría a ti difícil, o qué les haría a ustedes difícil realizar esta actividad? Tú sabes qué, este… José, yo los pondría aquí para que pudieran ver los *slides*, porque de ahí no los van a ver.

***** Primera mención que indica que han llegado nuevos participantes. Según las notas de los anotadores fueron participantes 8 y 9. Luego de acomodar a los nuevos participantes se siguió con la dinámica*****

Moderadora

Qué bueno que vinieron más. Me alegra mucho que hayan venido. Se puede sentar ahí. Mire, ella se va a mover para acá para que se pueda sentar en la mesa. Se pueda sentar en la mesa. Y entonces, ¿qué les haría difícil para hacer este método? Seguir este método. ¿Hay algo que les haría difícil hacer este método?

Uno

Depende las condiciones. Que no estés en buena… en salud buena.

Moderadora

Ah, importante, que la persona no esté en buena condición de salud, para bajar al patio y hacer las cosas.

Uno

Sí. Como tenemos personas ancianas, personas mayores.

Dos

Claro, uno tiene que ayudarlos.

Tres

Yo tengo que pagar para que me limpien la parte de al frente…

Uno

Dime mi amor… que pues lamentablemente no pueden hacer esa limpieza… ¿qué fue mi amor? [creo que habla con la niña]

Moderadora

O sea, que usted tiene que pagar para que le limpien, porque usted no lo puede hacer.

Dos

Ay yo no, yo limpio, yo soy la que…. El patio. Pero cada vez, vuelve y crece de nuevo.

Moderadora

Exactamente. Ok. ¿Y por acá? ¿Hay algo… alguna cosa que se les haría difícil hacer esto? No. Ok.

Pregunta 9- ¿Hay algo que podría ayudarles a realizar esta actividad de manera más fácil?

Moderadora

Algo que podría ayudarles a realizar este método. Para que se les haga más fácil.

Tres

Hablar, con los demás.

Dos

Con los vecinos a ver.

Moderadora

Hablar con los vecinos a ver si ellos desean unirse a la causa.

Dos

Unirse. Bueno, nosotros verdad, como vecinos.

Pregunta 9a- ¿Necesitarían más información?

Moderadora

¿Usted cree que los vecinos necesitarían más información? O ustedes necesitarían más información…

Dos

Aján.

Tres

Sí.

Moderadora

Ok. ¿Sobre qué?

Tres

Eso mismo que está hablando con nosotros. Que puedan… vamos a suponer, ir casa por casa, y notificarle a todo el mundo.

Moderadora

Ok. O sea, que habría que hacer… habría que decirle a la gente, ir casa por casa y orientar a la gente.

Dos

Literatura.

Uno

Como hacen con los censos.

Cuatro

Mira, como hacer una pequeña inspección en los patios, de los diferentes hogares, y si ven algo que está más o menos… pues orientarlos, respecto a esa parte.

Cinco

Ellos tienen…. Yo creo que ellos tienen información.

Siete

Deben de multarlos. Los que tengan gomas….

Cinco

Exacto.

Siete

Los zafacones hay que hacerles rotos.

Moderadora

Ok. ¿Y por acá?

Siete

Hay que multarlos cuando vean una goma, pues multarlos. Los zafacones tengan rotos abajo.

Moderadora

Ok.

Cinco

Es todo lo que les hace falta. Porque yo creo que tienen bastante información.

Siete

Sí, son cosas del mosquito…

Pregunta 9b- ¿Necesitarían más adiestramiento?

Moderadora

¿Ustedes creen que necesitarían más adiestramiento para hacer eso?

Siete

No.

Cuatro

Más acción…

Cinco

¿Esto de reducción de fuentes de mosquitos? No creo que un adiestramiento fuerte se necesite.

Actividad #2 – Aplicar larvicidas al agua acumulada

Moderadora

Ok. Pues vamos para la próxima. Aplicar larvicida al agua acumulada. Los larvicidas son pesticidas, son unos insecticidas, verdad, que se usan para matar las larvas. Ustedes ya saben qué es la larva verdad, porque lo vieron. Antes de que se conviertan en mosquitos adultos. Las larvas… Los larvicidas se pueden aplicar en diferentes maneras, en gránulos, o granos, en tabletas, en granos o en líquido. La aplicación de larvicidas puede reducir la cantidad de mosquitos si se aplica correctamente. Los larvicidas no afectan a las personas, ni a sus mascotas, ni el ambiente, si se siguen las instrucciones en la etiqueta. Los larvicidas no deben usarse en agua potable para consumo humano o animal. Requiere aplicar la cantidad correcta según las instrucciones de la etiqueta. Requiere reaplicarse cada cierto tiempo. Y no alcanza lugares que estén ocultos donde los mosquitos se reproducen. Esto es lo que yo te decía que hay que hacerlo en dos *bullets*. [parece que habla con Coral] ¿Ok? Y entonces, ¿tienen alguna pregunta de lo que dice ahí? ¿Entendieron lo que dice? Si uno tiene preguntas, me pregunta. Si no tiene… si no lo tiene claro, me pregunta.

Uno

¿Esos larvicidas es al alcance de uno? O sea, de que uno va y lo compra, en una tienda X.

Moderadora

Sí. Sí, en las ferreterías, hay una ferretería muy famosa, de una cadena de una megatienda, los puede conseguir. En la ferretería por ahí. Usualmente también lo consigue, me han dicho que lo consiguen en los…

Cuatro

Agro centros…

Moderadora

Agro centros. Eso mismo, los agro centros.

Pregunta 2- ¿Es esta una actividad nueva para usted o es algo que ya había escuchado antes? Si la escuchó, ¿dónde la escuchó?

Moderadora

Entonces, ustedes… ¿es esta una actividad nueva para ustedes o es algo que habían escuchado antes?

Tres

Yo no… yo lo había escuchado…

Dos

Lo había visto…

Moderadora

¿Lo había escuchado? ¿Dónde?

Tres

Fueron… este… fueron visitando el barrio.

Uno

El municipio.

Tres

Ajá.

Moderadora

En el municipio.

Uno

El municipio llevó…

Tres

Y nos llegaron a regalar unas pastillitas así, para que le echáramos al agua que tenemos…

Moderadora

¿Cómo aquéllas que están allí?

Tres

Exacto.

Dos

Sí, esas.

Moderadora

Ok.

Tres

Sí, y ellos nos explicaron.

Moderadora

Sí. ¿Y por acá?

Siete

No.

Seis

No.

Moderadora

¿No lo habían escuchado antes?

Seis

No.

Moderadora

Ok. ¿Por allá, lo había escuchado? ¿Larvicida? Sí. ¿Dónde?

Nueve

Sí, buenas. Mi nombre es [participante dice su nombre], presidente de aquí de VG. Ese fue mi primer trabajo en mi vida.

Moderadora

Ah, usted es el fa… vamos a quitar el nombre de aquí, porque estamos grabando, ¿usted permite que grabemos?

Nueve

Sí. Claro.

Moderadora

Ok. Pero, quitamos sus nombres verdad, porque es confidencial lo que diga, pero me habían hablado de usted. Yo trabajé en el Departamento de Salud. Catorce años. Mira para allá.

Nueve

Eso fue unos cuantos añitos. Eso fue cuando salí de… empezando la universidad, empecé a trabajar allí en el Departamento de Salud.

Moderadora

¿Y en sus tiempos se usaban…? Ajá.

Nueve

Cuando eso era… era… corría con fondos, este… federales. Completo. Y aquí el supervisor lo… del área era el señor Pereira. Ése era el… Y pues, tuve la oportunidad de trabajar y… vamos a hablarlo así, de combatir el mosquito. Conozco de la larva, conozco, pues… los últimos detalles de ese asunto, no los conozco, pero sí cómo se produce, cómo se reproduce, el caso… el daño que causa. La manera en que se utilizaba antes, para eliminar. Y yo creo que en Puerto Rico se eliminó bastante. Hace tiempo.

Moderadora

Sí señor.

Nueve

Estamos hablando ya de los años 70. Sí. Y era una preocupación bien grande porque verdaderamente, el mosquito, que era como se le conocía antes *Aedes aegypti*, era totalmente peligroso. Peligroso de… en el sentido de que hubo hasta muerte causadas por ellos. Y naturalmente, la falta de información para ese tiempo, que la escolaridad, era menos que la de ahora, pues eso ayudaba a la reproducción por la falta de… no interés, si no de conocimiento, porque las personas le tenían miedo. Aunque íbamos a visitar, porque nosotros trabajábamos en grupo, era un vehículo federal, y eso causaba un poquito de malestar y miedo a las personas, ve. Miedo en el sentido de que se creían, porque hubo muchos trabajadores [no entendí la palabra en la grabación], porque se creían que eran agentes federales, para ese tiempo. Luego, hubo una pausa bastante grande, bastante larga. Desde que se eliminaron las oficinas, para este tiempo. Falta de comunicación al pueblo, porque el *Aedes,* que era como nosotros le decíamos, era más peligroso de lo que la gente se podía imaginar.

Moderadora

Aján. Todavía.

Nueve

Y todavía sigue siéndolo. Y eso es una cosa que se produce en donde quiera. Y yo les voy a contar a ustedes una anécdota que me sucedió a mí esta semana en mi casa. En mi casa, eh… ella, que es mi esposa, dijo, ‘[nombre del participante]…’, yo tengo cajas [de dientes] y la de… la parte de abajo no la uso por incomodidad. Y yo la tengo dentro de un envase con agua limpia. El cambio un día sí, un día no. ¿Qué me dijo?, ‘[nombre del participante], en… ahí con los dientes hay algo… ‘, y yo ‘pero, como que con…’, pues como era un envase que tenía agua, pues, exactamente, tenía larvas.

Moderadora

Sí. [risas] Sí.

Nueve

Estamos hablando de un envase de aproximadamente tres pulgadas de ancho y como dos pulgadas de profundidad. Había. En el baño. En el baño, había. Inmediatamente…

Moderadora

Sí, que, en el tiempo suyo, se utilizaba el abate. Y entonces, el larvicida que se usaba, muy probablemente. Y entonces, solamente lo podían usar los inspectores de…

Nueve

El malatión.

Moderadora

…de control de vectores.

Nueve

Malatión era el líquido que se utilizaba para fumigar las casas.

Moderadora

Malatión. Sí.

Nueve

Era un sistema bien caro, era costoso. Había unos inspectores que se dedicaban única y exclusivamente a la identificación de la larva, dónde encontrarla. Eso es un problema de… ese mosquito tiene una cosa bien peculiar, que es de agua limpia. En agua oscura, no se da. En agua oscura se da la mosca y otras cosas más, y… ¿y qué era lo que se hacía? Pues aquí estaba este bloque, nos salía positivo, positivo es que había larvas, ve, porque el mosquito, tú no lo puedes identificar hasta que no… hasta que no muere. Pero tiene que localizarlo. Pero la larva es más fácil, porque es de agua limpia y bien especialmente donde hay sombra. Él se reproduce en las sombras con más facilidad. El sol le afecta, porque el agua, que es el área donde vive, este… se calienta por el sol y no es fácil. Pero en las aguas claras se da. Hay que tener mucho cuidado con las matas de agua y otras…

Ocho

Bromelias.

Moderadora

Bueno, ahora hemos aprendido Don [nombre del participante], porque se han hecho más estudios, que hasta en agua sucia.

Nueve

Ah, eso desconocía.

Moderadora

Sí, en agua de pozo muro, en agua… no fango, obviamente, no en fango, aunque uno… yo he visto larvas en fango, pero ahora se ven también porque necesitan material orgánico, necesitan hojas y necesitan la comida que hay en el envase con… que tiene desperdicio, para la larva comer. Ahora lo hacemos así.

Pregunta 3- ¿Cree que esta actividad reduciría el número de mosquitos en su comunidad? ¿Por qué?

[no se hizo]

Pregunta 3a- ¿Qué beneficios o ventajas tiene esta actividad para usted?

Moderadora

Bueno y entonces. ¿Qué beneficio usted le ve a esta… a este método del larvicida? ¿Qué beneficios ustedes le ven?

Tres

Bueno, que, si eso limpia el agua para que el mosquito no llegue, nos beneficia. Porque esa agua si la usamos, consumo para nuestras plantas, para lavarnos o para X razones, va a ser como desinfectarla.

Moderadora

Y si… ¿Y qué otro beneficio ustedes le ven?

Cuatro

Controla el desarrollo de la larva.

Moderadora

¿Cómo?

Cuatro

Que controla el desarrollo…

Moderadora

Controla el desarrollo de la… ¿de qué?

Cuatro

De la larva.

Moderadora

De la larva. ¿Y Don [nombre del participante] que la usó? Ay perdón, dije el nombre. [risas]

Nueve

No, no, está bien.

Moderadora

Ajá y el número ocho [realmente nueve] que lo usó.

Nueve

Nueve…

Moderadora

¿Qué beneficio usted le ve?

Nueve

Bueno, recuerde que esto es un beneficio de mejorar la salud, no hay ningún otro.

Pregunta 3b- ¿Qué desventajas o dificultades le ve a esta actividad? ¿De qué forma se podrían solucionar esas dificultades?

Moderadora

Exacto. Y desventaja. ¿Cuál sería una desventaja? ¿Cuál sería una desventaja?

Uno

Que uno sin querer se la eche al agua de perro.

Moderadora

Exactamente. Que uno se lo eche al agua de la mascota. Porque fíjese ahora… antes, cuando él trabajaba, lo que se usaba era abate, que era un químico, verdad. Ahora, el que se usa es un… es orgánico. Y aunque, este… el envase puede decir que se puede echar en agua potable, nosotros no lo recomendamos, verdad. ¿Por qué no lo recomendamos? Porque somos puertorriqueños. ¿Qué hace el puertorriqueño? Ah, espérate… ¿esta es tuya? Esta es mía. Le voy a echar una… la etiqueta dice que le eche una tapita, verdad. Ah, pero si le echo más, sirve más. Y entonces, ya no se puede asegurar que la cantidad no sea dañina a la salud, cuando usted le echó más de lo que se supone que le eche, verdad. Y como los puertorriqueños somos así, que no leemos, pues entonces, no recomendamos ni que se le eche al agua potable, ni al agua con que usted se va a bañar, ni nada de eso. Si no que, si usted está guardando agua, digamos, por el huracán, para bajar los baños, para echarle a las matas o para mapear y cosas así, ah bueno, pues ahí sí. Ahí lo pueden echar.

Pregunta 4- ¿Cuán posible es realizar esta actividad en su comunidad para reducir el número de mosquitos? ¿Por qué?

Moderadora

Ok. Y entonces, ¿es posible se pueda realizar esta… este método en su comunidad?

Tres

Sí.

Moderadora

Para reducir el número de mosquitos. ¿Ustedes lo ven como algo posible que se pueda hacer?

Cuatro

Claro que sí.

Seis

Sí, es positivo, efectivo.

Cinco

Sí.

Uno

Siempre y cuando se compre.

Moderadora

¿Siempre y cuando se compre?

Dos

Que se compre.

Moderadora

O sea, ¿que eso podría ser otra desventaja entonces?

Dos

Sí.

Tres

Que la gente no…

Uno

Sí, al ser humano le gusta todo gratis.

Moderadora

O sea, que a las personas les gusta lo gratis.

Uno

Sí, casi todos. Dice, pero yo te voy a comprar esto, ah si me lo traes está bien, pero no…

Cuatro

Cómprame a mí primero…

Nueve

Es que es caro.

Pregunta 5- ¿Apoya usted esta actividad en su comunidad? ¿Por qué? (*Preguntar a cada participante del grupo de discusión*)

Moderadora

Ok. Y entonces, ¿usted apoyaría este método en su comunidad?

Uno

Sí.

Dos

Claro que sí.

Cuatro

Sí.

Seis

Sí.

Moderadora

¿Por qué?

Tres

Porque no queremos mosquitos.

Dos

Por los mosquitos….

Seis

Exacto. Bueno, de alguna manera hay que controlarlos.

Pregunta 5a- ¿Piensa que su comunidad apoyaría esta actividad? Sí, No, ¿Por qué?

Moderadora

Ok. Ok. Y entonces, ¿piensan ustedes que su comunidad apoyaría este método, el uso de este método?

Seis

Sí. Yo creo que sí…

Moderadora

¿Sí? ¿Hay duda? Ya hay duda. ¿Por qué?

Uno

No necesariamente.

Cuatro

Yo creo que lo apoyarían, si es que tú se los suple el material para hacerlo.

Moderadora

Ah, si se lo suplimos.

Cuatro

Pero si hay que comprarlo, dependiendo…

Moderadora

Ok. ¿Por qué? ¿Por la misma razón?

Uno

Si lo suples. Si se lo suples encantados. Pero si no se los suples… Entonces si la negra no tiene dinero…

Dos

Si no el vecino se lo compra. [risas]

Pregunta 6- ¿Qué otra información necesitaría para entender mejor esta actividad?

Moderadora

Ok. ¿Qué información necesitarían… necesitaría la comunidad para entender mejor esta actividad? O necesitarían ustedes.

Uno

Promoción. Promoción, literatura.

Moderadora

Promoción, literatura…

Uno

Personalmente hablarle a la gente.

Moderadora

¿Qué más? ¿Hay algo en específico, alguna información en específica que ustedes necesitarían? Para entender mejor este método. ¿No? Bueno.

Pregunta 7- ¿Considerarían realizar ustedes mismos esta actividad?

Moderadora

Ustedes mismos, ustedes en sus casas, ¿considerarían usar este método?

Cuatro

Sí.

Moderadora

¿Sí?

Uno

Sí.

Seis

Sí.

Dos

Bueno, sí.

Moderadora

Ok.

Pregunta 8- ¿Qué les haría difícil realizar esta actividad?

Moderadora

¿Qué les haría difícil realizar este método?

Tres

¿Qué se haría qué?

Moderadora

¿Qué… qué le haría a usted difícil… qué le haría a usted difícil realizar este método, hacer este método en su casa?

Siete

No. Eso no es difícil.

Tres

No creo que me vaya a costar nada…

Seis

Claro que no.

Moderadora

¿No cree que le vaya a hacer difícil? Ok.

Pregunta 9- ¿Hay algo que podría ayudarles a realizar esta actividad de manera más fácil?

Moderadora

¿Hay algo que podría ayudarles a realizar esta actividad de manera más fácil? ¿No?

Pregunta 9a- ¿Necesitarían más información?

Moderadora

¿Necesitarían más información ustedes o la comunidad?

Dos

Claro.

Moderadora

¿En términos de qué?

Dos

Pues sobre el larvicida ese… sobre el agua acumulada esa, las gomas, el patio, todo eso.

Pregunta 9b- ¿Necesitarían más adiestramiento?

Moderadora

Ajá. Ok. ¿Y necesitarían más adiestramiento para usar este método?

Dos

Aján.

Uno

Sí.

Seis

Quizás para…

Siete

Dónde conseguirlo.

Seis

Quizás para muchos no, pero quizás para personas mayores ya quizás de la tercera edad, quizás se les haga un poco más complicado porque muchos simplemente conocen lo que es el *Clorox*, un poquito de *Clorox* al agua. Hay quienes no conocen cómo se utilizan ese tipo de… de material…

Moderadora

Ok.

Seis

…químico para controlar la propagación del mosquito. En este caso te puedo poner, por ejemplo, un vecino mayor de 80, supervisarlo, porque el que no pueda, por la edad, mover alguna paila de agua que se llenó por la lluvia, pues entonces, nosotros como vecinos también estar pendiente a eso…

Moderadora

Ah, muy bien.

Seis

…quizás, este… facilitándole los químicos para que entonces esa persona pueda… nosotros ayudarlo, e independientemente, en algún momento dado esa persona pueda hacerlo.

Moderadora

Ok. Ok. Bueno… Buenas, ¿Cómo está?

Diez

Bien, bien.

*****Llega un participante nuevo, participante número diez (*Diez*)*****

Actividad #3 – Rociar larvicida desde un camión

Moderadora

Pues vamos para la próxima que es… se acuerdan de que, yo les había dicho que venía… el larvicida venia en grano, en tableta y en líquido. Vamos a ver cómo se utilizaría en líquido. En líquido es hacer… o rociar larvicida desde un camión. Se le parece a algo, verdad que sí.

Ocho

Sí… [no se entiende el resto por niños hablando]

Tres

Sí, cuando fumigaban.

Moderadora

Los larvicidas se pueden aplicar de diferentes maneras. Pero muchos programas de control de mosquitos han encontrado, que aplicar el larvicida… que aplicar el larvicida desde un camión puede ser efectivo para alcanzar la mayoría de los lugares donde se encuentran las larvas. El larvicida se rocía desde un camión sobre edificios, la vegetación, y las propiedades, y también los terrenos, etc. Los larvicidas tienen que reaplicarse, o sea, reusarse regularmente. Dios mío, como están estos pajaritos, ¿qué es esto?

Uno

Es que están hablando de que van a fumigar los compañeros…

Cuatro

Sí…

Nueve

Eso es polilla.

Moderadora

No, que va, esa es la polilla. Y no es la mía. [risas]

Pregunta 2- ¿Es esta una actividad nueva para usted o es algo que ya había escuchado antes? Si la escuchó, ¿dónde la escuchó?

Moderadora

Ok. Bueno. ¿Habían escuchado antes de rociar larvicida desde un camión? Como la fumigación.

Tres

Sí.

Cuatro

Sí.

Seis

Sí.

Moderadora

¿Lo habían escuchado?

Dos

Sí, ellos pasaban por la casa…

Uno

Antes…

Tres

Antes se hacía.

Moderadora

Sí, pero una cosa es rociar insecticida, y aquí el señor sabe, que es rociar larvicida. Porque el insecticida, como nos decía, nos contaba él, el número nueve antes, se usaba el malatión, verdad, que es el líquido que mata los mosquitos adultos. Pero esto es rociar larvicida líquido para matar las larvas. No lo habían escuchado antes, ¿verdad? Habían oído del método, pero no con larvicida.

Uno

No, no con la…

Tres

No.

Pregunta 3- ¿Cree que esta actividad reduciría el número de mosquitos en su comunidad? ¿Por qué?

[No se hizo la pregunta]

Pregunta 3a- ¿Qué beneficios o ventajas tiene esta actividad para usted?

Moderadora

¿Qué beneficios o ventajas ustedes le ven a este método?

Uno

Bueno, si mata los huevos y las larvas, me imagino que está matándolos. Porque te está, te está, ¿cómo es? Te está ocupando más área.

Moderadora

O sea, que cubre más área…

Uno

Cubre más área.

Moderadora

Ok. ¿Qué más? ¿Qué otro beneficio o ventaja le ven?

Tres

No hace, no hace ningún efecto a… como la fatiga, asma.

Moderadora

O sea, que, entonces, sería eso, información que habría que buscar, verdad, darle a la gente. Si tiene algún efecto nocivo hacia la salud como este… matar… causar asma o algún tipo de condición respiratoria. Verdad, o alergia. Eso sería una desventaja, y que además habría que solucionarlo buscando información. Ok. ¿Algún otro beneficio?

Diez

Tenemos que ver hasta dónde llegaría, este… a eliminar las larvas.

Moderadora

¿En qué sentido?

Diez

O sea, por ejemplo, en las casas. Hasta qué lugar, cuando rocíen, podría… hasta dónde podría llegar dentro de…

Nueve

El alcance.

Diez

En los patios. El alcance que tendría… en los patios.

Moderadora

Ok. Ok. O sea, que no solamente que pase el camión por la calle, sino dentro de sus predios…

Uno

La parcela, la parcela.

Moderadora

…dentro de su patio, si verdaderamente llegase.

Uno

En la parcela.

Moderadora

Muy bien. O sea, que eso sería como otra dificultad. Otra…

Nueve

Desventaja.

Moderadora

…desventaja.

Pregunta 3b- ¿Qué desventajas o dificultades le ve a esta actividad? ¿De qué forma se podrían solucionar esas dificultades?

[Se contestó en otra parte]

Pregunta 4- ¿Cuán posible es realizar esta actividad en su comunidad para reducir el número de mosquitos? ¿Por qué?

Moderadora

Ok. ¿Sería posible realizar este método en su comunidad? Si se hiciera.

Tres

Yo pienso que sí.

Seis

Han pasado…

Uno

Si la guagua está afuera, no voy a decir, porque eso es cuesta baja, cuesta arriba…[risas] El camión tiene que estar muy…

Moderadora

Yo no sé si ustedes se han percatado, pero aquí hay una diferencia de usar larvicida en grano a usar larvicida desde un camión. ¿Cuál sería la diferencia?

Seis

El efecto directo.

Tres

Uno es *spray* líquido y el otro es grano.

Uno

Que va directamente a… el grano, me imagino que va directamente a…

Seis

¿El efecto directo que quizás pueda tener?

Moderadora

Sí, pero hay una diferencia que nadie me ha dicho.

Seis

¿Cuál?

Moderadora

¿Quién haría esto?

Seis

Una agencia privada, una agencia, el municipio.

Moderadora

Exactamente. Es el municipio quien haría esto. Esto no lo puede hacer una persona.

Uno

No sí, eso sí. Por eso digo, debe tener un camión bien bueno.

Pregunta 5- ¿Apoya usted esta actividad en su comunidad? ¿Por qué? (*Preguntar a cada participante del grupo de discusión*)

Moderadora

Exactamente. Si el mu… si… digamos que el gobierno o el municipio decidiera que puede hacer esto, ¿usted apoyaría el uso de este método en su comunidad?

Tres

Sí.

Dos

Claro.

Uno

Volvemos a lo mismo. Si no nos afecta porque lamentablemente hay personas que a veces… no escar… que tienen el problema, sino que hacen… no… cómo puedo explicar eso… eh, siempre es un no. No porque mi hijo es asmático. No porque no, porque no, y bueno todo es no. Y yo, bueno, pero mijito, vamos a buscar alternativas. Porque si tú no quieres que vengan mosquitos, pues yo sé que tú no quieres eso, pues vamos a buscar alternativas, buscar mascarillas, que avisen cuándo van a hacerlo, para que las personas se preparen. Este los asmáticos verdad, que se pongan sus mascarillas, cosa de que en lo mucho, que lo pequeño sea bastante bueno.

*Anotadora tiene en sus notas que un participante diferente al #3, #2 y #1, (no identificado con #) dijo: Mjm.

Pregunta 5a- ¿Piensa que su comunidad apoyaría esta actividad? Sí, No, ¿Por qué?

Moderadora

Claro. Y entonces. ¿Piensan ustedes que su comunidad apoyaría esta actividad?

Tres

Sí.

Moderadora

¿Sí la apoyarían? ¿Por qué?

Siete

Esa mas que todas.

Tres

Porque es beneficiosa.

Dos

Es beneficio para uno.

Moderadora

¿Porque es beneficioso y por qué?

Siete

Esa mas que todas.

Moderadora

¿Más que todas? ¿Por qué tú dirías que esa más que…?

Siete

No tienen que hacer nada. Pasa el… la guagua, y eso cubre bastante. Por lo menos con el olor se siente hasta un terreno.

Diez

Como eso se ha hecho antes también, ya la gente tiene conocimiento.

[se escucha a la participante cinco decirle a la hija, que se está ahogando, salir del salón]

Moderadora

O sea, que como la gente ha visto ese mismo… forma de regar insecticida la gente lo va a apoyar.

Diez

Claro.

Moderadora

Y como no lo tienen que hacer ellos.

Pregunta 6- ¿Qué otra información necesitaría para entender mejor esta actividad?

Moderadora

Ok. ¿Qué información necesitarían para entender mejor este método? Ya yo entiendo que necesitarían información sobre si hace daño o no hace daño a la salud. Sobre si tuviera un alcance, hasta cuan… hasta cuan alcanzaría dentro del patio. ¿Qué otra información debería tener? Entendería que necesitaría.

Uno

Los pros y los contras.

Moderadora

Los pros y los contras. Ok.

Actividad #4 – Fumigación dentro de las casas con insecticida de acción residual

Moderadora

Bueno, pues vamos para la próxima. Oye, estas cositas son tremendas. Fumigación dentro de las casas con insecticida de acción residual. La fumigación dentro de las casas con insecticida de acción residual es un método de control de mosquitos donde un profesional adiestrado… esto me está acá matando… donde un profesional adiestrado trata el interior de su hogar con insecticida. Consiste en rociar las paredes y otras superficies de la casa con un insecticida que continúa funcionando varios meses. Mata a los mosquitos que se posen en superficies que han sido rociadas con el insecticida. Este tipo de fumigación se ha usado en muchos países del mundo incluyendo Puerto Rico y Estados Unidos, ahora sé por qué estaba prendido. Bendito sea Dios. Entonces, puede ser efectivo en reducir la cantidad de mosquitos si se aplica en una gran cantidad de casas en un área. Después de aplicarlo, puede que haya olor por unas horas, pero es poco probable que cause daño a las personas cuando se hace correctamente. Requiere del permiso de la persona, verdad, del dueño de la casa y de la disponibilidad para entrar a la casa a fumigar. El uso repetido, a través del tiempo, o sea, que, si se usa mucho a través del tiempo, puede hacer que los mosquitos sean resistentes a los insecticidas. Y entonces, esta fumigación debe repetirse para mantener bajas las poblaciones de mosquitos. ¿Entendieron?

Tres

Sí…

Cuatro

Sí.

Moderadora

Este es un insecticida que se llama de larga duración, porque dura varios meses. Van a tu casa, fumigan, y fumigan todo. Las paredes de arriba a abajo fumigan todo. Esto no es si fumigan estos mosquitos…estas polillas. Entonces, mata los mosquitos que se posen, los mosquitos se tienen que posar en la pared o donde se eche, en el piso, o en el *hamper*, como ustedes ven aquí, este… si el mosquito se posa ahí, pues entonces, el mosquito muere, verdad. Y este… necesitaría aplicarse en muchas casas. En muchas casas en un área grande. Entonces, después de aplicarlo puede ser de que quede olor. Que es que… puede ser que quede olor, por algunas horas. Pero es poco probable que cause daño. Se requiere que la persona de su permiso para que se haga y el uso… el mucho uso constante puede hacer que los mosquitos se vuelvan resistentes. Y hay que estarlo haciendo constantemente, verdad. Para que la población de mosquitos… cada varios meses, verdad, hay que volverlo hacer para que los mosquitos se mantengan… este las poblaciones de mosquitos se reduzcan. Dios mío, que es esto.

Diez

Hay que apagar las luces…

Moderadora

Probablemente es por la luz. ¡Mira para allá!

Pregunta 2- ¿Es esta una actividad nueva para usted o es algo que ya había escuchado antes? Si la escuchó, ¿dónde la escuchó?

Moderadora

Bueno, ¿habían escuchado ustedes sobre este método antes?

Diez

Aján.

Cinco

Yo esto no lo había escuchado.

Tres

No.

Uno

Bueno, sí, hay profesionales que se dedican a eso.

Moderadora

Ah porque son exterminadores. Usted está hablando de exterminadores, verdad. Pero esto no es como la exterminación.

Uno

No, me imagino que no.

Cinco

Bueno, yo, en mi caso, yo no lo había escuchado.

Moderadora

Porque la exterminación es en el piso. Pero esto, ¿dónde lo ponen?

Cinco

En las paredes, en las puertas…

Cuatro

En las paredes…

Moderadora

En las paredes, en los bordes, en todo. Cogen la casa completa. Verdad. Y entonces, pero se parece mucho al de exterminación.

Uno

Sí, se parece mucho.

Pregunta 3- ¿Cree que esta actividad reduciría el número de mosquitos en su comunidad? ¿Por qué?

Moderadora

¿Cree usted que esta actividad, que este método, perdón, reduciría el número de mosquitos en su comunidad?

Cuatro

Ayudaría.

Tres

Sí.

Pregunta 3a- ¿Qué beneficios o ventajas tiene esta actividad para usted?

Moderadora

¿Por qué? ¿Qué beneficios tiene?

Uno

Porque va directamente dentro del hogar.

Diez

Porque eso mataría a los mosquitos que no se mataron afuera. Luego de exterminar afuera, pues los que se quedaron dentro de la casa, si había larvas y eso, pues se exterminaría…

Moderadora

Y como dice ella, verdad, eso está complementando con lo que dice número uno, que es, que entonces, mata los mosquitos que están dentro de la casa.

Dos

Los que están dentro de las casas.

Moderadora

Ok. Y entonces, ¿habrá algún otro beneficio, ventaja de este método?

Cuatro

Como es de más larga duración… como tiene… es de más larga duración…

Moderadora

Tiene…

Cuatro

Exacto.

Moderadora

Tiene más larga duración, verdad.

Uno

Es individual… casa por casa. Dentro del hogar.

Tres

Una pregunta.

Moderadora

Sí, claro.

Tres

Este… ¿qué efecto hace… a suponer, con las abejas y las mariposas?

Moderadora

Ok. Quiere se decir entonces, que se necesitaría saber si el producto que se utilice es dañino a otros insectos. Como las abejas, mariposas… ¿usted dijo mariposas? Ok. A las abejas y las mariposas. O sea, que eso sería una información que usted quisiera saber.

Tres

Aján.

Pregunta 3b- ¿Qué desventajas o dificultades le ve a esta actividad? ¿De qué forma se podrían solucionar esas dificultades?

Moderadora

Ok. Y ahora, otras desventajas o difi… y eso también si lo tiene, pues podría ser una desventaja. Ok. ¿Qué otras desventajas ustedes le ven? Si alguna.

Uno

Que es un poquito más… porque mucha gente que no dejan entrar extraños a mi casa. Entonces, hace un poquito más difícil con las personas, dejar entrar al profesional que llegue [no entiendo el audio] y todas esas cosas, porque la gente no tiene esa confianza.

Moderadora

Exactamente. O sea, que también habría personas que se quejen o que no dejarían entrar a la persona, ¿por qué? Porque no dejan entrar a las personas a su casa. Entonces, sería… ¿alguna persona ve otra desventaja de esta actividad? De este método.

Uno

No.

Cinco

Ahí dice de que si se utiliza mucho puede que los mosquitos sean resistentes, “so”…

Moderadora

Ok. O sea, que eso podría ser una desventaja.

Cinco

Exacto.

Pregunta 4- ¿Cuán posible es realizar esta actividad en su comunidad para reducir el número de mosquitos? ¿Por qué?

Moderadora

Ok. Y entonces, ¿es posible que esta… este método se pueda hacer en su comunidad? ¿Ustedes lo ven como algo posible?

Cuatro

Después que haya una buena orientación… se les diga…

Cinco

Es cuestión de una buena coordinación, porque muchas personas trabajan. Es más, qué día se podría hacer.

Diez

Este, con permiso, este también, sería, bueno… una buena orientación porque así las personas, pues están conscientes de que eso se puede hacer y lo beneficioso que podría ser.

Moderadora

Y qué… ¿Qué debería incluirse en la orientación?

Diez

Bueno, yo creo que *flyers*.

Siete

*Flyers*, radio.

Diez

Sí, este también, radio. También internet.

Moderadora

Ok. Pero ese es el método. Yo le pregunto, qué tipo de información debería compartirse.

Diez

Ah ok. Pues, se les llevaría la información de to… eh… de lo que conlleva y cómo se aplicaría, cómo se haría ese método. Y los beneficios que salgan de eso…

Uno

Y cuánto por ciento hay de seguridad de que va a matar esos mosquitos.

Moderadora

Ok. Ah. Qué por ciento hay de que mate los mosquitos…

Uno

De que si es efectivo.

Pregunta 5- ¿Apoya usted esta actividad en su comunidad? * ¿Por qué? (*Preguntar a cada participante del grupo de discusión*)

Moderadora

Ok. Y entonces, ¿usted apoyaría esta actividad en su comunidad?

Diez

Claro que sí.

Moderadora

¿Y usted apoyaría esta actividad en su comunidad número siete? ¿Usted apoyaría esta actividad en su comunidad?

Siete

Sí.

Moderadora

Y número seis, ¿apoyaría esta actividad en su comunidad?

Seis

Sí.

Moderadora

¿Y número cinco?

Cinco

Sí, sí.

Moderadora

¿Número cuatro, número nueve?

Cuatro

También.

Nueve

Sí.

Moderadora

Sí. ¿Número ocho?

Ocho

Sí.

Moderadora

Sí. ¿Y usted?

Tres

Sí.

Moderadora

¿Y usted?

Dos

Sí.

Moderadora

¿Usted?

Uno

Sí.

Seis

Lo importante es la coordinación.

*Anotadores presenciales tienen en sus notas que los participantes apoyan la actividad.

Pregunta 5a- ¿Piensa que su comunidad apoyaría esta actividad? Sí, No, ¿Por qué?

Moderadora

¿Piensan que en su comunidad apoyarían este método?

Tres

Yo pienso que sí.

Moderadora

¿Piensan que su comunidad apoyaría este método?

Tres

Sí. Porque sería para su beneficio.

Cuatro

Sí. Si están bien orientados.

Moderadora

Sí.

Uno

Si vamos a decir en por ciento, por lo menos como el 80% dice que sí.

Moderadora

¿Por lo menos, tú crees, que el 80% dice que sí?

Pregunta 6- ¿Qué otra información necesitaría para entender mejor esta actividad?

Moderadora

Y entonces, ya más o menos cubrimos la pregunta de qué tendría que incluirse, verdad. Que sería el por ciento de efectividad, que sería… ¿qué fue lo que usted me dijo horita?

Diez

Pues, de que las personas estuvieran conscientes de… este… de lo que se les va a aplicar en…

Moderadora

Exacto. El producto…

Diez

El producto.

Moderadora

…que utilizaría.

Tres

¿Qué pasaría… qué pasaría, a suponer, donde no vive gente?

Moderadora

Ah… muy bien. Eso sería otra pregunta, si aún con tantas casas abandonadas que hay, continuaría siendo efectivo, verdad. Aun con el número de casas que hay. Y cómo entonces, se trabajarían esas casas que están abandonadas.

Actividad #5 – Trampa AGO para mosquitos

Moderadora

Ok. Bueno, pues vamos para la próxima. Ahora es que se está poniendo buena la cosa. Trampas AGO para mosquitos. Ya escuchamos del señor aquí que sabe… quiero escuchar un poquito las opiniones. La trampa para mosquitos AGO, ya ha sido utilizada en Puerto Rico para reducir el número de mosquitos. Se ha utilizado en el municipio de Caguas, de Salinas, la utilizamos y la vamos a utilizar ahora en Guayama. La trampa atrae y captura los mosquitos hembra *Aedes aegypti* que buscan envases para poner sus huevos. La trampa consiste en una paila negra de cinco galones. Lo ven, que es una paila plástica con 5 galones. De cinco galones, una cámara de captura, que es esto, la cámara de captura es esto, la paila, la cámara de captura… este… y un escrín. Miren el escrín aquí. La paila está llena hasta la mitad con agua y heno, y esa agua… esa mezcla de agua y heno atrae a los mosquitos, verdad, que tiene un olor que atrae los mosquitos. A las mosquitas hembras. Dentro de la trampa hay un papel… dentro de la cámara de captura, hay un papel con pega especial que atrapa a los mosquitos. Verdad, que es este papel. Que atrapa a los mosquitos cuando entran a poner sus huevos. La trampa… la trampa contiene material orgánico, que es el heno, que… así que puede oler. Verdad, como les había dicho. No requiere de entrenamiento especializado para armar la trampa. La trampa requiere mantenimiento cada dos meses, para que no se convierta en un criadero de mosquitos. Y la trampa reduce la cantidad de mosquitos si se mantiene adecuadamente y se usa en ocho de cada diez hogares dentro de la comunidad. O sea, que para que la trampa funciona, debe ponerse en ocho de cada diez casas. Y tienen que ponerse tres trampas en cada casa.

Pregunta 2- ¿Es esta una actividad nueva para usted o es algo que ya había escuchado antes? Si la escuchó, ¿dónde la escuchó?

Moderadora

¿Habían escuchado estas trampas anteriormente?

Siete

No.

Cinco

No.

Seis

No.

Tres

No.

Uno

Esa no, esa es la primera vez.

Moderadora

No. ¿Dónde lo había escuchado?

Diez

La orientación que ustedes me dieron en mi casa.

Moderadora

Exactamente. Exactamente.

Uno

Hubo una reunión de líderes comunitarios y entonces, y ahí… la primera vez que la…

Moderadora

Ajá, claro. Tú también, verdad, en la reunión de líderes.

Pregunta 3- ¿Cree que esta actividad reduciría el número de mosquitos en su comunidad? ¿Por qué?

Moderadora

¿Creen que esta actividad… cree que este método, esta trampa, reduciría el número de mosquitos en su comunidad?

Uno

Sí.

Moderadora

Sí. ¿Por qué?

Uno

Porque es más factible para llegar a los hogares. La trampita esa. Como que es más…

Moderadora

Es más factible. Y usted, ¿qué cree? El número nueve. ¿Crees que esa trampa funcionaría para reducir el número de mosquitos en la comunidad?

Nueve

Funciona, porque después que se pongan en los sitios adecuados y áreas señaladas para eso tiene que ser efectivo. Cualquier movimiento que se haga en contra del mosquito va a ser positivo.

Pregunta 3a- ¿Qué beneficios o ventajas tiene esta actividad para usted?

Moderadora

Ok. Y entonces, ¿qué beneficios o ventajas ustedes le ven a esta trampa?

Tres

Es beneficiosa, pero también tiene que estar uno consciente de… tener bien consciente de cada cuanto tiempo hay que cambiarle.

Moderadora

Exactamente. Hay que estar consciente de cada cuanto tiempo hay que cambiarle, verdad, que es de cada dos meses. Hay que cambiarle… darle mantenimiento, verdad. Ok. ¿Qué más beneficios le ven o ventajas?

Diez

Yo le veo la ventaja de que lo que se va a usar es heno que es una… natural, que no lleva, o sea que no le va a hacer daño a nadie. Porque es una cosa natural.

Moderadora

O sea, que es una cosa natural. Porque el heno es algo natural.

Diez

Natural…

Moderadora

Ok. Y entonces, ¿algún otro beneficio que le vean o ventaja que le vean?

Pregunta 3b- ¿Qué desventajas o dificultades le ve a esta actividad? ¿De qué forma se podrían solucionar esas dificultades?

Moderadora

Ok. ¿Qué desventajas o dificultades le ve a este método?

Uno

Pues, volvemos a lo mismo, de estar pendientes al tiempo porque si no, pues se hace un criadero de mosquitos.

Tres

Y también, por los niños.

Moderadora

Ah, ¿por qué por los niños?

Tres

Bueno porque ellos pueden tocarlo…

Cinco

La curiosidad.

Tres

Exacto. Tumbarlo, o meter la manita, o chuparse los deditos con esas cosas…

Moderadora

O sea, que entonces, se tendría que dar esta información sobre si lo que hay dentro, verdad, que es el heno y agua, si pudiese ser perjudicial para un niño.

Diez

No es el heno como tal, es el envase…

Moderadora

El envase.

Diez

El efecto que pueda tener el envase cuando lo puedan coger.

Moderadora

Ok.

Uno

Que no abra… que tenga un seguro bueno, porque el niño puede abrirlo y… ahí se descocotó.

Moderadora

Ok. Ok. Y entonces, ¿cómo se podrían solucionar esas desventajas?

Uno

La seguridad de la trampa. O sea, ver la seguridad que tiene la trampa.

Moderadora

Ok. ¿Y entonces? Y entonces, ¿Qué más? ¿Qué más? ¿Cómo se podrían solucionar esas dificultades?

Diez

Llevando una y mostrándosela a las personas y decirles pues, orientarla sobre cómo alguien tendría que…

Tres

Sabiendo también dónde se consiguen.

Moderadora

Ah, dónde se consiguen. Si están asequibles, verdad.

Uno

Si están accesibles. Si son gratis, porque esa es la primera pregunta. ¡Hello! [risas]

Moderadora

Si es gratis.

Uno

Yo sigo insistiendo, y discúlpame, sigo insistiendo.

Diez

Sobre todo.

Moderadora

No, claro. El costo, verdad que sí.

Uno

Si hay un costo…

Moderadora

Y entonces…

Siete

Si va a ser carita.

Moderadora

Ajá, dime.

Siete

Debe ser bastante cara. Debe ser bastante cara.

Diez

Pero si lo provee el municipio…

Moderadora

Se solucio… ¿Cómo se solucionaría?

Diez

Pues que nos proveyeran gratis las trampitas…

Moderadora

Las trampas y, ¿qué más?

Diez

O por lo menos un precio… si no las van a vender a un precio…

Uno

Un precio razonable.

Moderadora

Los suplidos verdad, los suplidos del cambio que son la pega y el heno, verdad.

Uno

Exacto.

Siete

Mira, una paila vacía de un litro vale seis pesos, más IVU, una paila vacía. Y si tiene todas esas cosas… [conversa con otro participante]

Pregunta 4- ¿Cuán posible es realizar esta actividad en su comunidad para reducir el número de mosquitos? ¿Por qué?

Moderadora

Y entonces, ¿ustedes creen que es posible que esta actividad se pueda realizar en su comunidad?

Uno

Sí.

Tres

Sí.

Diez

Sí.

Siete

En mi casa yo las pongo…

Dos

Aján.

Moderadora

¿Sí? ¿Por qué?

Diez

Yo creo, con el permiso, después de una buena orientación para los residentes… que se digan los pros y los contras.

Seis

Bueno, yo creo que todos queremos… este… verdad, minimizar la propagación de mosquitos y las enfermedades, “so” yo entiendo que sí. No debería haber ningún tipo de problema, porque no sería solamente para el beneficio de una persona, sino de…

Diez

La comunidad.

Seis

…la comunidad completa.

Pregunta 5- ¿Apoya usted esta actividad en su comunidad? ¿Por qué? (*Preguntar a cada participante del grupo de discusión*)

Moderadora

Ok. Y entonces, ¿apoyarían ustedes, esta actividad?

Diez

Yo sí la…

Moderadora

¿La apoyaría número siete?

Siete

[no se escucha en el audio]*

Cinco

Sí.

Moderadora

¿Número seis?

Seis

Sí. Sí.

Moderadora

¿La apoyaría?

Ocho

Sí.

Moderadora

¿La apoyaría?

Cuatro

Sí.

Dos

100%.

Tres

Sí.

Moderadora

La apoyaría. Ok.

Uno

[Participante asiente con la cabeza. Anotadores presenciales tienen en sus notas que todos apoyaron la actividad.]

Nueve

[Participante asiente con la cabeza. Anotadores presenciales tienen en sus notas que todos apoyaron la actividad.]

Moderadora

¿Por qué la apoyaría?

Dos

Por nuestra salud.

Cinco

Bueno, ahí indica que reduce la cantidad de mosquitos hasta un 50… o sea, más todavía también, “so”.

Pregunta 5a- ¿Piensa que su comunidad apoyaría esta actividad? Sí, No, ¿Por qué?

Moderadora

Exacto. Y en su comunidad, ¿la comunidad apoyaría esta actividad?

Seis

Sí.

Moderadora

¿Por qué lo apoyaría?

Dos

Por nuestra salud.

Cuatro

Por la salud.

Siete

Porque hay muchos mosquitos.

Pregunta 6- ¿Qué otra información necesitaría para entender mejor esta actividad?

Moderadora

Ok. Y entonces, ¿qué información necesitarían, la comunidad y ustedes, para entender mejor esta actividad?

Cinco

Se puede ofrecer un ejemplo. O sea, por lo menos, llegar un día, vean cómo se hace el proceso… una demostración para que ellos entiendan.

Moderadora

¿VG ya tuvo, verdad, la actividad de *Townhall Meeting*? Porque en las actividades que se están haciendo por las comunidades la llevan, y la explican. Bueno, ok, pues vamos para la próxima. Don… número nueve, aun yo estoy muy intrigada con su opinión.

Nueve

Vamos.

Moderadora

¿Le gusta esta…?

Nueve

La información está completa.

Moderadora

Sí, pero ¿le gusta el método?

Nueve

Sí.

Moderadora

El método de la trampa.

Nueve

Eso es un producto naturalmente que, mayormente, para que se haga más fácil, una orientación de cómo se construye, el envase.

Moderadora

Ah ok. Que usted quisiera información sobre cómo se construye.

Nueve

Sí, porque… porque aquí todos miramos el costo efectivo. Vale tanto, vale tanto, no lo puedo hacer porque vale tanto. No puedo…

Moderadora

Pero, esto no sería para que la gente lo hiciera. Esto ya viene hecho.

Nueve

Por eso, viene hecho, porque eso es así, pero… pero, es costoso.

Moderadora

Ok.

Nueve

Y es más fácil… es más fácil orientarle cómo se construye, ser bien efectivo que esperar que el gobierno, o quien sea, lo facilite.

Moderadora

Ok. Ok. ¿O sea, que si el gobierno las diera?

Nueve

Mucho más fácil.

Pregunta 7- ¿Considerarían realizar ustedes mismos esta actividad?

[No se hizo la pregunta]

Pregunta 8- ¿Qué les haría difícil realizar esta actividad?

[No se hizo la pregunta]

Pregunta 9- ¿Hay algo que podría ayudarles a realizar esta actividad de manera más fácil?

[No se hizo la pregunta]

Pregunta 9a- ¿Necesitarían más información?

[Se contestó en otro lado]

Pregunta 9b- ¿Necesitarían más adiestramiento?

[No se hizo la pregunta]

Actividad #6 – Mosquitos macho y hembra con Wolbachia

Moderadora

Ok. Vamos para la próxima. Mosquitos con *Wolbachia*. Ahora vamos a hablar sobre los mosquitos estériles. *Wolbachia* es una bacteria que vive en muchos insectos incluyendo algunas especies de mosquitos que pican a las personas. Es una bacteria natural. Se encuentra en el ambiente, algunos insectos la tienen, incluyendo mosquitos de otras especies. En Puerto Rico hay más de cuarenta especies de mosquitos, el *Aedes aegypti* es una. Pero hay muchas, muchas más especies de mosquitos. Como el *Culex,* como el *Anofeles,* como [nombre de una especie muy complicada de entender, jejeje], como muchísimas otras especies de mosquitos*…* Ahora, el mismo mosquito que transmite… hasta ahora, enfermedades en Puerto Rico es el *Aedes aegypti,* ven. Pero la *Wolbachia* no se encuentra en los mosquitos *Aedes aegypti* que transmiten el dengue, el Zika y el Chikunguña en Puerto Rico. La *Wolbachia* se introduce a los mosquitos *Aedes aegypti* en un laboratorio. Al presente, los estudios muestran que el uso de mosquitos infectados con *Wolbachia* es seguro para los animales… las personas, los animales y el ambiente. Los científicos creen que cuando nacen mosquitos con *Wolbachia* pueden ser menos capaces de transmitir enfermedades a las personas. ¿Entendieron esta información? ¿Tienen alguna pregunta?

Seis

¿Son genéticamente alterados? ¿O no?

Moderadora

Se hace… se les pone la *Wolbachia* en un laboratorio, de los genéticamente modificados vamos a hablar de esos. Es el último. Pero la *Wolbachia* se le pone en un laboratorio al mosquito. Don… número nueve sabe que en el… en los laboratorios pueden criar los huevos, verdad. Cuando ponen las trampas de mosquitos, ponen las trampas en las casas, pues la que… hay un tipo de trampa en que la mosquita hembra pone sus huevos ahí, en un papel. Ese papel se puede llevar… verdad, se lleva al laboratorio, verdad, y esos huevitos se sumergen en el agua y nacen las larvas. En Puerto Rico se hace eso, pero no se pone la *Wolbachia* porque hay que tener unos permisos especiales de la FDA y todo eso. Además, que no tenemos fábricas de eso aquí. Pero, en donde lo hacen, verdad, que ya mismo vamos a ver en qué países se están haciendo, pues ahí, esos mosquitos que ya están… que se germinan los huevos, los huevitos se sumergen en el agua y nacen las larvas, a esos mosquitos se les inyecta la *Wolbachia*. Y entonces, la próxima generación de mosquitos ya nace con *Wolbachia*. Y eso es lo que voy a explicar. ¿Hay alguna otra pregunta de esta información?

Tres

*Wolbachia*, ¿es la bacteria?

Dos

La bacteria.

Moderadora

¿Qué no entendiste? Que veo que hiciste [algún gesto]. ¿Por qué hiciste [gesto]?

Siete

Eso de un laboratorio, inyectarle eso a los mosquitos... [risas]

Cinco

Ya veo por dónde va. [más risas]

Siete

Sí.

Moderadora

Ok. Y entonces… Alguien por aquí dijo otra cosa, no se…

Tres

Yo…

Moderadora

Número tres.

Tres

Que *Wolbachia*, ¿qué es aparte de ser una bacteria?

Moderadora

Una bacteria. No es ninguna otra cosa.

Tres

Pero ¿por qué le ponen esa bacteria?

Moderadora

Porque esa bacteria hace que los mosquitos no puedan transmitir virus.

Siete

Sí, por ahí mismo meten… se muera todo el mundo, le meten una… [le contesta algo a alguien] Yo estoy aquí pero no digo nada [conversa con otra persona, creo que un anotador]

Moderadora

Es importante que escuchen eso. Ella dice que, ¿qué es la *Wolbachia* además de una bacteria? Es una bacteria, no es otra cosa. Y que, ¿por qué se les inyectan a los mosquitos?

Siete

Pues, porque nacen con la bacteria y no cogen el… la otra bacteria….

Moderadora

Porque la *Wolbachia* hace que la… que ningún virus pueda estar en el mosquito.

Siete

Solamente tienen ese… está en el ambiente ya…

Uno

Esa bacteria es más fuerte que lo que… tú sabes, es fuerte para amarrar los… el mosquito. Para que no…el virus…

Moderadora

Tu cuerpo tiene bacterias.

Cinco

Todo el cuerpo…exacto, todo el cuerpo tiene bacterias.

Moderadora

Si tu cuerpo no tuviera bacterias, tú no podrías tener al… no podrías hacer el proceso de digestión. Tu cuerpo tiene bacterias desde la boca hasta… por ahí para abajo. Y las de acá abajo son diferentes a las de allí. Que las de allí, que las de dentro del estómago. Dentro del estómago también tenemos bacterias porque si no, no podemos descomponer la comida para que el cuerpo la pueda usar. La parte de nuestro cuerpo que más bacteria tiene es la boca. Por eso es que, hay que lavarse los dientes, tres veces al día. Porque si esas bacterias se proliferan usted va a tener grandes problemas con sus dientes, con su lengua y con su digestión. ¿Verdad que sí? Pues así mismo es esta bacteria. Esta bacteria… esta es una bacteria que la hay en el ambiente natural y que la tienen otros insectos, pero este *Aedes aegypti* no la tiene y entonces, se le pone. Para que evite que los virus que coja los mosquitos se lo pasen a otra persona. Así es el asunto. Entonces, *Wolbachia* trabaja de dos maneras, pero usted me tiene que decir lo que siente en su corazón. [risas] Usted me lo tiene que decir porque usted aquí porque su opinión cuenta.

Siete

No, no tengo ninguna duda ahora.

Moderadora

Su opinión es importante y cuenta. diferentes. Y el caballero que llegó quiero su opinión también.

*****Llega el último participante número 11*****

Moderadora

Ok. Bueno, pues la *Wolbachia*… los mosquitos con *Wolbachia* funcionan de dos maneras. Esas dos maneras es… la primera. Se liberan mosquitos *Aedes aegypti* macho y hembra con *Wolbachia*. Míralo aquí, el macho con *Wolbachia* y la hembra con *Wolbachia*. Se liberan al ambiente, se sueltan. Cuando el mosquito hembra con *Wolbachia* se reproduce con un mosquito macho o… con o sin *Wolbachia* la bacteria se pasa a través de la hembra a sus crías, de generación en generación. ¿Lo ven? Se unen ellos, cuando paren, cuando salgan los huevitos, salen los mosquitos con *Wolbachia*. Y entonces, con el tiempo la cantidad de mosquitos con *Wolbachia* aumenta y reemplaza a los mosquitos del ambiente sin la bacteria. Después de liberarlos varias veces la población de mosquitos con *Wolbachia* se mantendrá sin tener que liberar más de estos mosquitos. Porque como siguen de generación en generación pasándola, pues va a llegar un momento en que ya no hay que tirar más los mosquitos con *Wolbachia* porque ya van a haber. Los mosquitos con *Wolbachia* son menos capaces de transmitir enfermedades. Aun habrá mosquitos en la comunidad, porque van a estar los que tienen *Wolbachia*, ya que la intención de este método no es reducir el número de mosquitos sino reducir el riesgo de epidemias. Sin embargo, no se reducirá las picadas de mosquitos. O sea, va a haber siempre mosquitos lo único es que no van a poder transmitir enfermedades. Va a haber menos riesgo de que transmitan enfermedades. Esta actividad se ha usado en otros países como Colombia y Brasil. Actualmente no hay reglas definidas para el uso de los machos y hembras con *Wolbachia* en los Estados Unidos. O sea, no hay reglas en los Estados Unidos que regulen tirar machos y hembras, los dos a la vez, en los Estados Unidos. Por lo tanto, se ha usado en Colombia y en Brasil solamente.

Pregunta 2- ¿Es esta una actividad nueva para usted o es algo que ya había escuchado antes? Si la escuchó, ¿dónde la escuchó?

Moderadora

¿Habían escuchado ustedes antes sobre esto?

Uno

No.

Dos

No.

Tres

No.

Moderadora

¿Jamás?

Ocho

Primera vez.

Seis

No. Primera vez. Ni en mi ciencia de Biología. [risas]

Pregunta 3- ¿Cree que esta actividad reduciría el número de mosquitos en su comunidad? ¿Por qué?

Moderadora

¿Creen que esta actividad reduciría… este método, reduciría el número de mosquitos en su comunidad?

Seis

Ay, yo no sé…

Siete

El número de mosquitos no.

Moderadora

El número de mosquitos no.

Siete

No.

Moderadora

¿Por qué?

Uno

Las enfermedades, ¿no?

Cinco

No porque el va a seguir… el mosquito va a seguir reproduciéndose.

Moderadora

¿Qué es lo que puede reducir?

Uno

Las enfermedades.

Dos

Las enfermedades…

Siete

Pero es la mejor, yo diría, de todas de las que ha dicho. Es el mejor método yo creo.

Uno

Las picadas. Las picadas siguen doliendo.

Moderadora

¿Este es el mejor método? ¿Tú crees?

Cinco

Ay no. No.

Uno

No, porque las picadas siguen doliéndome. [risas] Digo, siguen doliendo las picadas.

Siete

Mosquitos siempre van a haber. Que te piquen, pero sin dengue. [risas]

Uno

Bueno, esa es buena también.

Moderadora

O sea, que el beneficio es… la desventaja es, según tú, que van a haber mosquitos como quiera y te van a picar y las picadas duelen. La ventaja para él es que te… van a haber mosquitos, pero ahora no van a transmitir la enfermedad.

Uno

Las enfermedades no van…

Moderadora

No van a transmitir el dengue.

Diez

Otra ventaja que habría, que tendríamos, sería lo que dijo la señora, no hay riesgo de que las abejas mueran, ni que las mariposas, ni nada de eso. Sino que entonces habría, y se mantendría el equilibrio….

Uno

El balance.

Moderadora

Muy buena observación.

Diez

Porque…Como eso va pasando, como dice ahí, de generación en generación de mosquitos, habría mosquitos, pero no hay el riesgo de esos que tienen entonces…

Uno

De enfermedades, hay más control de enfermedades entonces.

Diez

…las abejas y otros insectos que pueden ayudar en la naturaleza.

Moderadora

Ok. Dígame.

Tres

Cómo uno va a saber cuál tiene….

Uno

La bacteria.

Tres

Exacto. Cuál tiene las enfermedades o cuál va a tener la *Wolbachia*.

Uno

Déjate picar. [nombre del participante], déjate picar. [risas]

Siete

Como ahora mismo…

Moderadora

O sea, que eso sería una desventaja.

Tres

Porque ustedes no van a recoger todos los mosquitos. Para todos…

Uno

Tienen que hacerle como a las aves. Que las aves tú las marcas con un numerito bajo el ala… [risas] Sí. Si tú tienes aves en tu casa para que el ave… sepas dónde están tus aves, pues viene un metalito con un número un control de tu ave y tú le pones…

Siete

¿Te vas a poner a mirar el mosquito?

Diez

No, pero lo que pasa es… que a medida que…

Moderadora

Pues no está tan difícil la cosa porque fíjate que hay estudios, y esto es aparte verdad, hay estudios donde se les… a los mosquitos que van naciendo se les pone un tipo de líquido, verdad, que los pinta, para uno saber hasta cuán lejos viajan.

Tres

Sí, eso sí lo he…

Moderadora

Para uno saber cuánto es el *range* que ellos pueden viajar de un lugar a otro. Cuánto se pueden desplazar. Esos estudios se han hecho.

Tres

Eso se ha hecho en ballenas… en muchas cosas.

Seis

¿Y qué probabilidades entonces hay de que realmente ese mosquito no te transmita nada?

Moderadora

Ah esa es otra… o sea, una información que hay que saber es, cuán… qué probabilidades, cuánto es el por ciento de que esos mosquitos verdaderamente…

Seis

De que eso en efecto sea válido. Que sea efectivo para nosotros.

Uno

Por eso, que sea seguro.

Moderadora

Esa es una información que tú necesitarías tener.

Uno

Bueno, para la duda, ponte repelente, verdad.

Dos

El repelente.

Diez

Yo creo que sería investigar… que sería bueno investigar qué resultados dio en esos países.

Moderadora

Que resultados dio…

Siete

¿En Estados Unidos no se ha hecho todavía?

Diez

No… Lo que pasa es que…

Moderadora

Macho y hembra, no. Vamos a ver en el próximo. Porque acuérdate que yo te dije que eran dos maneras en que se usaban los *Wolbachia*. Esto es, liberando, verdad, soltando, machos y hembras a la vez con *Wolbachia*. Este método no se ha utilizado. Solamente en Colombia y en Brasil.

Diez

Habría que ver los resultados que ellos obtuvieron, si fue bien o no.

Pregunta 3a- ¿Qué beneficios o ventajas tiene esta actividad para usted?

[No se hizo la pregunta]

Pregunta 3b- ¿Qué desventajas o dificultades le ve a esta actividad? ¿De qué forma se podrían solucionar esas dificultades?

[No se hizo la pregunta]

Pregunta 4- ¿Cuán posible es realizar esta actividad en su comunidad para reducir el número de mosquitos? ¿Por qué?

Moderadora

Ok ¿Cuán posible es que se pueda realizar esta actividad, este método en su comunidad? [risas]

Diez

¡Ay virgen!

Moderadora

¿Por qué se ríen? Número seis, número cinco, díganme, ¿cuál es la risa?

Seis

Es que estos métodos científicos como que tenemos una distancia y categoría.

Moderadora

¿Por qué?

Seis

No sé. Es que la ciencia pone algo…

Siete

Porque así mismito como inyectan el *Wolbachia*, también pueden…en un laboratorio pueden inyectar…

Cinco

Inyectan cualquier otra cosa.

Seis

No, porque yo voy a hacer, ‘ve, libera mil mosquitos en mi comunidad’. No creo verdad. No como que no… no le encuentro como que lógico. O sea, libéralos a ver cómo resulta todo… cómo sería efectivo ese método.

Moderadora

Ok. Pero, tú me estás trayendo ahí dos cosas. A ver qué… tú pensarías que si lo van a hacer en Puerto Rico es a ver si cómo funciona.

Seis

No ya… si lo sueltan, ya que.

Siete

No si lo han hecho en otros….

Moderadora

Que sería… ¿y si lo han probado en otros sitios y resulta que sirve?

Tres

¿Qué van a hacer esos mosquitos?

Siete

El mosquito va a tener la bacteria ya.

Seis

Sí, pero es que, ¿cómo podemos implementar eso directamente a mi comunidad…?

Siete

Eso es con tiempo.

Moderadora

O sea, que tú deberías tener información…

Uno

Eso es cuesta arriba. Ese es cuesta arriba, ese sí que es cuesta arriba.

Seis

Cómo se llevaría a cabo el proceso.

Siete

Eso tiene que ser por... por…

Seis

Para entonces, yo poder entender y poder decir si para mí sería efectivo o no sería efectivo y estar de acuerdo o no.

Moderadora

Ok. Muy bien.

Uno

Y eso es a largo plazo.

Moderadora

Alguien por aquí dijo algo.

Tres

Yo. Yo digo que, el mosquito que tiene… que ya le han inyectado. ¿Qué van a hacer esos mosquitos? ¿Acabar con los que ya están infectados o seguir picando igual que los demás?

Uno

No, pero siguen picando. Siguen picando.

Tres

Por eso, esos van a picar y nosotros vamos a seguir matando.

Uno

La diferencia es que no va a haber la enfermedad. Pero va a seguir picando.

Moderadora

O sea, que hay menos posibilidades de que se transmitan las enfermedades. O sea que…

Tres

Sí, pero, también van a estar sueltos los que están…

Uno

Por eso, que tú no sabes quién es quién.

Tres

Por eso, uno no sabe, como quiera los va a matar.

Uno

Sí eso es el proceso.

Diez

Con permiso…

Siete

Sí, pero eso es un proceso.

Diez

Eso sería un proceso.

Siete

Eso lo recogen, me imagino que lo sueltan un…

Uno

Eso como que no sé, no me convence.

Tres

No.

Moderadora

¿No te convence? Ok.

Tres

No.

Diez

Yo creo que no.

Siete

A mí sí.

Seis

Yo estoy igual que ella. A mí no me convence.

Uno

Bueno, por lo menos, no sé. No sé, pero le estoy buscando la vuelta, busco el mosquito por algún lado y todavía no me convence.

Siete

Es como abejas, como si te picara una abeja y no te doliera.

Diez

Lo que pasa es que a medida que va pasando el tiempo…

Pregunta 5- ¿Apoya usted esta actividad en su comunidad? ¿Por qué? (*Preguntar a cada participante del grupo de discusión*)

Moderadora

Ok, ¿apoyaría usted… vamos acá, apoyaría usted esta… este método en su comunidad?

Diez

Bueno, tendría que llevar basta información para que la gente tenga credibilidad.

Moderadora

¿Y usted, lo apoyaría en su comunidad? ¿Sí?

[Anotador presencial tiene que el participante #7 apoya la actividad, y participantes #4 y #6 no apoyan la actividad.]

Moderadora

¿Y en su comunidad?

[Anotador presencial tiene que el participante #7 apoya la actividad, y participantes #4 y #6 no apoyan la actividad.]

Uno

Ah y el problema de esto…

Moderadora

¿Y tú?

Cinco

Número cinco, no.

Moderadora

Ok. ¿Y usted?

[Anotador presencial tiene que el participante #7 apoya la actividad, y participantes #4 y #6 no apoyan la actividad.]

Moderadora

¿Y usted número nueve? ¿Sí?

Nueve

Toda información es buena.

Tres

Yo me voy neutral.

Moderadora

¿Sí?

Nueve

Sí.

Moderadora

¿Y usted?

Tres

Neutral.

Moderadora

Neutral. ¿Número dos?

Dos

Indecisa.

Moderadora

Indecisa.

Uno

El problema de esto es que, ok, si sueltas el mosquito en mi comunidad, tampoco estoy segura de que el mosquito se quede aquí. A lo mejor se va para el vecino, para la comunidad vecina. Entonces, yo me quedo guindando. O sea, como quiera, no. No.

Tres

Ya que tenemos mosquitos y nos van a echar más.

Moderadora

El número diez.

Seis

Once.

Moderadora

Once.

Once

Tengo muy poca información.

Moderadora

Tiene poca información, no puede decidir todavía. Ok. Bueno. O sea, que se necesitaría tener más información de todo lo que hemos dicho. De qué es la *Wolbachia*, el proceso, todo eso.

Pregunta 5a- ¿Piensa que su comunidad apoyaría esta actividad? Sí, No, ¿Por qué?

Moderadora

Ok. Y entonces, ¿piensa que su comunidad apoyaría este método?

Diez

No creo.

Moderadora

¿No cree?

Siete

Yo no creo.

Moderadora

¿No creen que su comunidad lo apoyaría?

Dos

No.

Siete

No porque no tiene… le falta información. Falta mucha información, yo creo que no.

Moderadora

Porque falta mucha información.

Uno

Sí, es cuesta arriba. Es cuesta arriba.

Seis

Sí, es cuesta arriba. Buscar los por cientos, cuán efectivo…

Once

Suena a mucha película… [risas]

Uno

Eso como que es cuesta arriba.

Moderadora

Y entonces…

Tres

Nosotros estamos evitando que nos piquen los mosquitos, y nos van a echar más mosquitos… No tiene chiste.

Diez

Tal vez si se usara en Estados Unidos, verdad, tal vez tendríamos un poquito más de credibilidad.

Seis

Esa es la palabra, credibilidad.

Pregunta 6- ¿Qué otra información necesitaría para entender mejor esta actividad?

Moderadora

Ok. Muy bien. Pues yo no les voy a preguntar qué otra información necesitarían para entender mejor este método porque ya lo han dicho.

Actividad #7 – Mosquito macho con Wolbachia

Moderadora

Nos quedan dos más y acabamos. Dos más. Uno más, el que viene ahora y otro. Ahora viene la otra forma de que se puede llevar a cabo la *Wolbachia*. En la segunda forma solo se liberan mosquitos macho con *Wolbachia*, estos machos. Se liberan machos al ambiente, que no pican ni transmiten enfermedades. Los mosquitos macho con *Wolbachia* se unen a las hembras sin *Wolbachia* que hay en el ambiente. Las hembras sin *Wolbachia* van a poner sus huevos, pero estos no van a nacer.

Uno

Ese es bueno. Eso es sexo seguro. [risas]

Moderadora

Y entonces, los mosquitos con *Wolbachia* deben liberarse continuamente, en grandes cantidades para mantener baja las poblaciones de mosquitos en la comunidad. Una vez que los mosquitos con *Wolbachia* dejan de ser liberados en un área, la población de mosquitos va a aumentar de nuevo. O sea, la población de mosquitos normal va a aumentar de nuevo. Porque ya no están los mosquitos con *Wolbachia* que cuando se casan con las hembras, ¿qué pasa con los huevos? No nacen, verdad. Y entonces, los mosquitos macho con *Wolbachia* se han utilizado en California, en los Cayos de la Florida. Y han sido aprobados para su evaluación en Miami, Florida.

Uno

Eso está más cerca. O sea que el pasaje vale más caro. Porque es más cerca. [risas] Colombia está más lejos, ahora está más cerca.

Moderadora

Lo primero, la primera técnica, que es el… primer método, que es que se liberan machos y hembras, eso no se ha usado en Estados Unidos, ni hay reglas para usarlo, pero éste, que solamente sean los machos, sí.

Cinco

Ok.

Tres

Por la temperatura…

Pregunta 2- ¿Es esta una actividad nueva para usted o es algo que ya había escuchado antes? Si la escuchó, ¿dónde la escuchó?

Moderadora

Ok. ¿Habían escuchado sobre esta actividad?

Cinco

No.

Siete

De la actividad no.

Dos

No

Uno

No.

Tres

No.

Moderadora

¿No?

Pregunta 3- ¿Cree que esta actividad reduciría el número de mosquitos en su comunidad? ¿Por qué?

Moderadora

¿Cree que este método pueda reducir el número de mosquitos en su comunidad?

Nueve

Sí.

Dos

Si así… si es 100% sí.

Moderadora

El número dos dice sí.

Dos

Si es 100% sí.

Uno

Que ya por ahí me estas dando, este… un poquito más de esperanza.

Moderadora

Ah, de que no va a haber mosquitos.

Uno

De que no va a haber mosquitos porque al eliminarse… bueno, como dicen acá, sexo seguro. [risas] Porque a la vez que el mosquito coge a la tipa y ella pone los huevitos, para ningún lado va.

Moderadora

Sí. Sexo seguro.

Uno

Esa es buena.

Moderadora

Ok. Muy bien.

Pregunta 3a- ¿Qué beneficios o ventajas tiene esta actividad para usted?

Moderadora

Ok. ¿Qué beneficios o ventajas tiene este método para ustedes? Beneficios que tiene este método. ¿Tiene algún beneficio para ustedes?

Tres

Que no nos enfermemos.

Dos

No ponen huevitos…

Seis

Bueno el huevo no nace. El huevo, no nace…

Moderadora

El huevo no nace.

Uno

Pues hay menos mosquitos.

Moderadora

Exactamente. Si el huevo no nace hay menos mosquitos. ¿Algún otro beneficio? ¿Qué dice número cinco por allí?

Uno

Porque esos son los que traen la enfermedad, porque estamos hablando… estamos hablando de unos que no hacen nada, pero pican. Y hacen la misma… daño, porque los que son alérgicos a…

Moderadora

Yo quiero, yo quiero oír qué dice cinco.

Cinco

Pero, aunque no nazca el huevito, la que transmite es la hembra, verdad.

Moderadora

Pero no nacen sus huevos así que no puede haber más hembras.

Cinco

Vamos a verificar el mosquito macho con *Wolbachia*.

Uno

Sí, porque se queda la chica nada más… pero los hijos no van a nacer.

Pregunta 3b- ¿Qué desventajas o dificultades le ve a esta actividad? ¿De qué forma se podrían solucionar esas dificultades?

Moderadora

Ok. Y entonces, ¿qué desventajas o dificultades le ven a esta actividad?

Uno

Muy complicado.

Moderadora

Es complicado.

Uno

Sí, aunque quiera… [no se entiende]… es complicado.

Moderadora

¿Por qué es complicado?

Uno

Pues, porque primero debes tener los machos.

Moderadora

Pero eso también…

Uno

Que los machos, este… tienen que cogerlos, atraparlos, para entonces inyectarlos, para entonces… es un proceso eso.

Moderadora

Acuérdate que dijimos que… y los… estos mosquitos se cogen los huevitos y los huevitos se germinan en laboratorio.

Uno

Ok. Que ya saben la seguridad de que es macho. Ah pues…

Moderadora

Los machos tienen más bigote. Las nenas tienen antenitas, pero tienen menos bigote en las antenitas. Los machos, él lo sabe [refiriéndose al nueve] en las antenitas tienen mucho bigote. Y así se conocen. Aparte de… con otras cosas más, pero esa es la más evidente.

Uno

Sí porque imagínate. Más mosquitos y virarlos patas arriba…

Diez

Pero, este, con permiso, eso sería un proceso a…

Uno

A largo plazo.

Tres

A largo tiempo.

Diez

…a largo plazo. Porque en lo que los tiran y entonces se reproducen no sería… [no se entiende]

Moderadora

O sea, que usted ve eso como una desventaja.

Tres

Sí.

Uno

Sí.

Diez

Sí. Sería a largo plazo.

Uno

En el ínterin puede haber muchas enfermedades todavía. En lo que…

Pregunta 4- ¿Cuán posible es realizar esta actividad en su comunidad para reducir el número de mosquitos? ¿Por qué?

Moderadora

¿Ustedes creen que es posible realizar esta actividad comunidad para reducir el número de mosquitos? Este método en su comunidad, ¿usted cree que es posible? Seis ya…

Seis

Es más convincente que el anterior.

Moderadora

Ok. La cinco todavía lo está pensando.

Uno

No hay que pensar también de dinerito, porque cuánto va a costar la inversión, de traerlos aquí, todo eso para acá, que el laboratorio, científico, allá, pagar a todo el mundo, eso es un lío [no se entiende] Eso conlleva, me imagino que un costo. Y, ¿Cuánto?

Dos

Números.

Moderadora

¿Un costo para quién?

Uno

Para el gobierno.

Tres

Para el gobierno.

Seis

Para el gobierno, el alcalde, el municipio. Nosotros.

Uno

El municipio.

Moderadora

Exactamente.

Uno

Porque en algún momento dado te van a tener que cobrar, por un lado, a nosotros mismos. Pagar esa situación.

Pregunta 5- ¿Apoya usted esta actividad en su comunidad? ¿Por qué? (*Preguntar a cada participante del grupo de discusión*)

Moderadora

Y entonces, le pregunto. ¿Apoyaría usted esta actividad?

Diez

Bueno, yo la apoyaría siempre y cuando se le llevara una buena orientación al pueblo y que ellos estuvieran de acuerdo.

Moderadora

Número once, ¿apoyaría esta actividad ya que habló horita?

Seis

El once…

Once

No. No la apoyaría.

Moderadora

¿Por qué? ¿Por qué?

Once

No me convence.

Moderadora

¿Ni la otra? La otra… ¿ni la otra?

Once

No, la otra tampoco.

Moderadora

Ok. Número siete.

Siete

No está… no estoy seguro…

Cinco

Es la misma que la de… ser macho [no se entiende].

Siete

Sí… sí, sí, sí. Es la misma.

Moderadora

La apoyaría.

Cinco

Que si apoyó la otra… pero no, yo…

Seis

Esta la apoyo, la anterior no.

Moderadora

La otra no. Ok.

Cinco

Yo todavía no.

Moderadora

Todavía no. ¿Y usted, la apoyaría?

Cuatro

No.

Moderadora

¿Y usted la apoyaría?

Ocho

Yo creo que no, porque ella dijo que no.

Moderadora

¿Y el número nueve lo apoyaría?

Nueve

Sí.

Moderadora

¿Y usted la apoyaría? ¿No?

Tres

[No se escucha en la grabación.]*

Moderadora

¿Y usted la apoyaría?

Dos

[No se escucha en la grabación.]*

Moderadora

¿Y usted, la apoyaría?

Uno

Bueno, siempre y cuando tengan los chakachuquis, el *wiki wiki* [dinero] como dicen, para poder ponerse…

Moderadora

Yo quiero saber, por qué la número cuatro no lo apoyaría que lleva un ratito ahí callada.

Cuatro

Pues lo como lo que pasa es que no tenemos tanta información, ni unas estadísticas verídicas que digan que eso es así…

Cinco

Validez.

Uno

Exacto.

Cuatro

…pues entonces, ¿cómo se lo voy a llevar a mi comunidad? Si yo misma no me lo puedo explicar.

Moderadora

Ok. O sea, que debería tener más información.

Cuatro

Más información.

Moderadora

Información de estadísticas…

Cinco

Estadísticas, validez, credibilidad, porcentaje, o sea…

Uno

Si y todo lo que sea bueno…

Cuatro

Los resultados positivos en el área donde ya lo han aplicado. Para entonces, de ahí partir si es beneficioso para mi área.

***Anotadores presenciales tienen en sus notas que participantes #2 y #3 dijeron que no apoyarían la actividad.**

Pregunta 5a- ¿Piensa que su comunidad apoyaría esta actividad? Sí, No, ¿Por qué?

Moderadora

Ok. ¿Piensa que su comunidad apoyaría esta actividad?

Uno

Vuelvo a repetirte, si es gratis, sí, si no es gratis, no.

Cinco

Hasta que yo no me la crea…

Diez

Hay que llevarles más información.

Moderadora

Más información. ¿Y usted, piensa que la comunidad apoyaría?

Cinco

No.

Once

Todos se van a cohibir.

Moderadora

¿Por qué?

Once

Porque como están presentando la información… no tienen el conocimiento. Para dejar lo desconocido, nadie tiene ese dato… [no se escucha] A mí es como si me dijeran a mí que me inyectara eso yo. [risas] No me lo voy a poner. Es lo mismo, lo único es que me lo va a inyectar un mosquito después.

Moderadora

Ah, ok. Porque mira la lógica de él. La lógica de él es que el piensa que cuando el mosquito lo pique a él, le va a inyectar la *Wolbachia*. ¿Esa es la lógica?

Siete

¿Y si me Wolbachio? [risas]

Uno

Él tiene que saber si la *Wolbachia* es perjudicial a la salud del ser humano.

Moderadora

Ok. Muy bien.

Uno

Volvemos a lo mismo. Es información.

Moderadora

Entonces, por acá. ¿Lo apoyaría su comunidad?

Cinco

No. Yo no.

Moderadora

¿Usted cree que la apoyaría?

Cinco

No.

Moderadora

¿Y por este lado? ¿Lo apoyaría su comunidad?

Tres

No, yo pienso que…

Dos

Es que no hay mucha información.

Tres

En realidad, no sé si tenga… cómo es, credibilidad lo que digo…

Moderadora

Vamos a escucharnos, vamos a escucharnos.

Tres

No sé si tenga credibilidad lo que digo, pero yo pienso que… eso es un trabajo de más, cuando tenemos el *spray* que usted dijo primero que los acaba. Se me hace que es mejor el *spray* que los acabe y los…

Moderadora

Ok. ¿Cuál era la conversación que tenían por aquí? Ahora díganmela a mí. Que estaban hablando por aquí. El número siete y la número diez. Por aquí. Díganme.

Siete

No ella dice que hay que apoyarlo primero, para que entonces surja la información y toda la cosa.

Uno

Sí, a largo plazo. Eso es a largo plazo.

Moderadora

Ok. Bueno, pues vamos a la próxima.

Uno

Lamentablemente, aquí, por lo menos aquí en Puerto Rico es bien difícil la promoción. Porque ahora mismo, yo vine de una educación sencilla, yo estoy en la feria de salud que eso ponen muchas cosas. Están todos los de [no se entiende], está defensa civil… [no se entiende]. Cuando yo llego a defensa civil, que está el mapa de Ponce, de aquí del municipio de Ponce, de Ponce, fui allí y veo que mi parque, del que yo tengo la llave, dice que es zona de asamblea. Yo llamé al supervisor, quién es el supervisor de aquí, porque quiero saber, por qué esa [no se entiende] mi parque es reunión de asamblea de tsunami donde yo no tengo conocimiento. Donde en el parque pusi… yo me di cuenta, porque en el parque, cuando yo llegué al parque, me pusieron un letrero que dice, zona de asamblea de tsunami. Hello, en qué momento a mí me dijeron que mi parque, de mi comunidad, que yo le dé consciencia a mi gente, a mis comunidades que ese sitio va a ser reunión de asamblea. Nunca [no se entiende]. Ese es el problema aquí, que esto no se… o sea, si hay una persona que por usted se da la comunicación, aunque sea cualquier persona. Porque está… yo vengo y se lo digo a ésta, y ella se lo dice a [nombre del participante tres], [nombre del participante tres] se lo dice a la otra, y así sucesivamente. Pero cómo motivo de razón, yo vine a enterarme en una feria de salud, que mi parque de mi comunidad es sitio de asamblea de tsunami. Donde yo no tenía conocimiento. Donde la gente no sabe… por, en vez de poner reunión, casi siempre la asamblea es como para clubes grandes, tú sabes, como la Liga que hacen asamblea y qué sé yo qué… eh, nuestra gente entiende reunión no es asamblea. Y lo primero que me preguntaron fue, ‘[nombre de participante uno], ¿qué es eso de asamblea? Que en el parque hay un letrero con una cosa azul así…’ y yo, ‘pues, porque eso quiere decir que cuando tú te encuentras en este sitio, tú coges para este sitio’. ‘¿Y por qué no lo dicen?’ Se dieron cuenta ese día…

Moderadora

¿Y qué conlleva el no saber?

Uno

Falta de comunicación. Del municipio hacia nosotros.

Moderadora

Ok. O sea que… ¿y qué tiene que ver esto con esta información?

Uno

Por eso que estamos hablando. Porque si no llevas la información correcta a la comunidad, no… nadie se va a enterar, nadie se entera. Nadie se va a enterar. Por lo menos yo vine a enterarme aquí ahora, de que le ponen una inyección al macho, entonces a la hembra… aquí.

Diez

La primera vez.

Uno

Ustedes están conscientes de que nosotros estamos preguntando estas cosas, porque estas son las cosas que se están haciendo a nivel mundial. El gobierno de Puerto Rico tendría que aprobar estos…

Uno

Exacto.

Moderadora

¿Entienden? Y por eso, nosotros estamos, precisamente, como la comunidad no sabe, por eso estamos haciendo estos grupos de discusión para saber su opinión. Para conocer su opinión. Y se van a estar haciendo más reuniones con todos estos métodos. Explicando todos estos métodos a las comunidades. Pero si el gobierno de Puerto Rico no apoya o no… ellos son los que tienen que decidir sí o no, porque nosotros no podemos. Son, es el gobierno y la gente del gobierno son las que tienen… y los presidentes son los que tienen que decidir. Ahora, tienen que decidir, como ella dice, informados. No sin informar a las personas.

Uno

Por eso es que, estamos como estamos. Por la falta de comunicación.

Moderadora

Por eso es por lo que estamos haciendo esto, para que la gente tenga información. Para que la gente tenga esa información.

Pregunta 6- ¿Qué otra información necesitaría para entender mejor esta actividad?

[Se contestó en otra sección]

Actividad #8 – Mosquitos modificados genéticamente

Moderadora

Y entonces, la última. Y llegamos a los que tú dijiste. Mosquitos genéticamente modificados. Se llevan mosquitos macho para que se unan con mosquitos hembra del ambiente. Los mosquitos macho modificados genéticamente se producen… se reproducen con las hembras del ambiente y pasan un gen a sus crías que impide que las larvas y las pupas se desarrollen normalmente. Así, estas mueren antes de convertirse en mosquitos adultos. O sea, al mosquito macho le ponen un gen. En un laboratorio le ponen un gen. Y los liberan y esos mosquitos macho cuando se casan con las hembras y ponen los huevos, el gen se va a ir al huevo. Se va a ir con el huevo. Y cuando estos mosquitos…eh, estos huevitos se rompen, estas van a tener los huevos, y ¿qué les va a pasar? No van a nacer, se van a morir. Porque tienen ese gen para que mueran. ¿Entendieron? Los mosquitos macho generados no pican ni transmiten enfermedades. Estos mosquitos se deben liberar varias veces a la semana a lo largo del tiempo, en grandes cantidades para mantener bajas las poblaciones de mosquitos *Aedes aegypti* solamente. Una vez los mosquitos modificados genéticamente *Aedes aegypti*, dejan de ser liberados en un área, la población de mosquitos aumentará de nuevo. Los mosquitos modificados genéticamente han sido evaluados en diferentes países incluyendo las Islas Caimán, Brasil y Panamá. Al presente no se han hecho estudios de los mosquitos modificados genéticamente en los Estados Unidos.

Uno

Perdóname, pero no hay ningún indicio, no hay ningún papel, no hay ninguna evidencia que dice que esto es viable.

Pregunta 2- ¿Es esta una actividad nueva para usted o es algo que ya había escuchado antes? Si la escuchó, ¿dónde la escuchó?

Moderadora

¿Lo habían escuchado anteriormente? ¿No? Nadie lo había escuchado.

Seis

Son los *Aedes aegypti*, no Zika, ni el otro, solamente ese.

Moderadora

No, este… es que… vamos a ir por partes. Zika no es un mosquito.

Seis

Ah, verdad.

Moderadora

El dengue no es un mosquito. Es un virus. El Chikunguña es un virus. No es un mosquito.

Seis

Gracias por la aclaración.

Moderadora

Los virus son virus y los mosquitos son insectos. Son dos cosas diferentes. Un virus no puede mutar a otro virus. Mutan en sí mismos para mejorar o para empeorar. Pero no mutan… el Chikunguña, nunca va a ser Zika, ni va a ser dengue. Ni un mosquito puede convertirse en un virus, va a seguir siendo un mosquito. Y va a seguir siendo el mosquito de su especie, que es *Aedes aegypti*, ven. Y hay *Aedes aegypti,* hay *Aedes albopictus,* hay *Aedes mediovittatus*, hay *Aedes* de los miles *Aedes.* Pero el que tenemos en Puerto Rico es el *Aedes aegypti* que transmite las enfermedades del Chikunguña, Zika y dengue. Y que en el pasado y en el presente también transmite fiebre amarilla y mayaro y otras enfermedades.

Uno

El ambiente que esté ese… él se apoya de acuerdo con la convivencia de ellos.

Moderadora

Ellos adquieren el… ellos no nacen enfermos. Ellos pican a una persona que ya tiene el virus y cuando pican a esas personas ellos… ellos adquieren el virus, se infectan ellos. Y cuando pican a otra persona le transmiten el virus.

Uno

Entonces, [no se entiende] vino enferma, la picó el mosquito y nos chavamos.

Moderadora

Exactamente, así es que se riegan las epidemias. Yo fui a Brasil o fui a Colombia, allá había Zika y me traje el virus conmigo. Como aquí, no había Zika antes, la gente no tiene inmunidad. Pero hay los mosquitos que lo transmiten. Así que cuando yo me siente en el balcón de mi casa infectado con el virus y venga el mosquito y me pique va a seguir picando a todo el mundo que hay en mi casa. Y esos que hay en mi casa, cuando vayan al trabajo, a la escuela, a la oficina del médico, van allí, se van a sentar allí infectados y los mosquitos los van a picar y se los van a llevar a otra gente. Y así es la cadena de cómo ocurren las infecciones y las epidemias.

Uno

Por algún lugar tiene que empezar, eso tiene que ser alguien que vino infectado de allá de otros lares. Lamentablemente.

Moderadora

Así es que ocurre. Y antes, no ocurrían tan seguido porque antes no estábamos tan avanzados verdad, y no era lo mismo si uno estaba en Brasil llegar a Puerto Rico, como hoy en día, que en doce horas ya tú estás en Puerto Rico. Antes, cuando uno iba a Brasil, a Colombia, a África, se tardaba dos y tres días y quizás una semana, y si uno iba en barco, peor todavía, verdad. Estaba meses. Pero ahora los aviones son así [gesto con los dedos]. En un día tú te moviste de Bra… de Puerto Rico a Brasil, en menos de doce horas. Y regresas para atrás en las próximas doce. Y así es que ocurren las epidemias ahora tan rápido. Porque la gente se mueve rápido. La gente [no entiendo la palabra] y se mueve. Ok, habiendo aclarado eso, sigo yo.

Pregunta 3- ¿Cree que esta actividad reduciría el número de mosquitos en su comunidad? ¿Por qué?

[No se hizo la pregunta.]

Pregunta 3a- ¿Qué beneficios o ventajas tiene esta actividad para usted?

Moderadora

¿Qué beneficios o ventajas le ven a esta actividad?

Uno

A largo plazo, el beneficio sería fantástico a largo plazo.

Moderadora

A largo plazo hay un beneficio de, ¿qué?

Uno

Hay un beneficio. De menos enfermedades…

Cinco

Y el controlar.

Uno

Del control del mosquito.

Cinco

Controlar de que se propaguen más.

Moderadora

De que se propaguen más los mosquitos…

Cinco

Todos van a morir, van a morir todos.

Uno

Van a estar, pero no van a estar, así como esas manadas así grandísimas. Porque aquí en Puerto Rico, acuérdate que en Puerto Rico… el problema de Puerto Rico es que donde quiera hay un lago, donde quiera hay un charco, donde quiera... tú sabes, porque tú puedes controlar los de tu casa y quizás el del vecino, pero tú no puedes controlar como estamos viviendo, El TU, el mangle. El mangle cuando llueve se hacen charcos y esos charcos… Yo me acuerdo de que, cuando pequeña, yo iba con mi papá a pescar y cuando una vez veníamos, yo venía primero, venía sentada, yo iba a correr, porque eso era una cosa… los mosquitos estos verdes, esos verdes. Esos tipos me cayeron encima, yo salí corriendo, todo el mundo muerto de la risa allá pero cuando… era como una cortina, como si hubieras entrado a un cuarto lleno de mosquitos. Cuando yo entré a esa cortina…. Bueno, eso fue una cosa, que yo salí corriendo y todo el mundo riéndose, pero cuando entraron allí, me reía yo, porque ya yo estaba al otro lado, ve. Por los charcos que se hacen y tú no tienes control de eso. Porque eso es en el mangle, eso es monte, eso ahí no hay nada.

Moderadora

Bueno, ¿hay alguien que le ve algún beneficio o… u otra ventaja más?

Pregunta 3b- ¿Qué desventajas o dificultades le ve a esta actividad? ¿De qué forma se podrían solucionar esas dificultades?

Moderadora

¿Qué desventajas entonces, o dificultades le ven a este método?

Uno

Hacer el mosquito. Porque esto lo están haciendo…[no se entiende lo último]

Moderadora

Ajá, ¿eso sería una desventaja?

Uno

Sí.

Moderadora

¿Qué otra desventaja le ven? Están cansados verdad.

Diez

El tiempo que transcurriría en…de que eso ocurriera.

Moderadora

Ok. ¿Nadie más le ve más desventajas?

Uno

No pues un montón. Si venimos a ver… es cuesta arriba, tú sabes, es cuesta arriba.

Pregunta 4- ¿Cuán posible es realizar esta actividad en su comunidad para reducir el número de mosquitos? ¿Por qué?

Moderadora

¿Cuán posible es que se pueda realizar esta actividad en su comunidad para reducir el número de mosquitos?

Siete

Es posible.

Moderadora

¿Es posible?

Siete

Sí…

Uno

Bueno, siempre y cuando hagan… siempre y cuando lo hagan y lo suelten. El mosquito pica… vamos a poner que [no se escucha bien] para proteger esos mosquitos. O sea, porque en otros sitios hay mosquitos así, tú sabes cómo en nuestro... Por lo menos en el TU hay muchos mosquitos por la situación de que es área de costera. Que hay muchos mosquitos, pero hay sitios que son… no hay tantos.

Moderadora

Y bueno, cinco y cuatro. ¿Qué estaban hablando por allí?

Cinco

No, no, era para que no llegara aquí….

Moderadora

Bueno, este… ajá.

Tres

Este… ay, déjame ver si no se me olvidó. [risas] Que, yo pienso que en la orientación que se dice, verdad, de orientar al pueblo a… lo único que yo veo es, lo que yo veo es, que cuando tú digas… ¿qué pasa? [risas] que vas a soltar más mosquitos de los que ya tenemos, te van a decir que no.

Moderadora

Ok. Ok.

Uno

Sí, esa es una desventaja.

Tres

Que nadie quiere mosquitos.

Moderadora

Ok. ¿Y cómo se podría solucionar eso?

Tres

Bueno, pues dándoles la información…

Uno

Y lo crean.

Tres

Y que lo crean. Exacto…

Uno

Porque el problema de esto es que, con el internet ahora… antes había una guerra por allá por el [nombre inventado que no entiendo] y tú no te enterabas, porque no había… pues ahora tú te enteras de todo. Al enterarte de todo, ahora pues la gente como que está muy receptiva [creo que quiso decir, no está muy receptiva] a esa información. Porque dicen, ‘Ah Dios, esos mosquitos, a lo mejor es algo que están poniendo en esos mosquitos y nosotros somos los conejillos de India... ¿Ve?

Tres

Exacto.

Uno

…Somos conejillos de India. Tú sabes, eso es para probarlo y si nos descocotamos, nos descocotamos. Entonces, la gente dice, ‘para qué traen ese mosquito para…’ Porque no es seguro. Porque tiene que haber un por ciento genial, para que te digan, ‘mira, no, hay un 90% que al tirar el mosquito macho éste, sentado acá, va… es a largo plazo, pero va reduciendo la situación’. O sea, hay que estar bien…

Moderadora

Ok. ¿Y qué pasó con la información de internet?

Cinco

No toda la información es verídica.

Diez

No todo se puede creer.

Tres

No todos tienen…

Dos

Internet.

Once

Una pregunta. ¿Por qué antes de citarnos aquí no nos dieron la información para que la gente estuviera más informada de lo que íbamos hablar y las soluciones más efectivas?

Moderadora

¿Qué tipo de información… como qué tipo de información?

Once

La que estas leyendo, por ejemplo, esa.

Moderadora

Ah, porque precisamente, esto es para comenzar un diálogo. Para comenzar un diálogo. Yo me imagino que luego cuando se hagan más actividades de índole informativa pues entonces, se les va a dar entonces, material educativo… Ahora mismo si ustedes quieren… si ustedes quisieran obtener más información podrían ir al… ¿cómo es que se llama el site?

Coral

*Mosquito World Program*.

Moderadora

Al Mosquito World Program, que en internet… ¿y cómo se llama en español? Porque ellos tienen una página para gente en español. *Mosquito World Program*. Como Mundo… eh, Programa del Mundo del Mosquito, pero en inglés. *Mosquito World Program*. Y ustedes pueden buscar más información sobre *Wolbachia*. Si van a Oxytec pueden entonces, también buscar información sobre los mosquitos genéticamente modificados. Oxytec. Pero, para… no le damos la información antes porque esta es una primera aproximación. No todo el mundo que coge literatura científica la puede entender. Y entonces, tenemos que explicar. Con esta misma confusión del Zika, del dengue, del Chikunguña, de que si el mosquito Zika… esto mismo, porque no todo el mundo tiene la capacidad de entender el nivel científico, verdad, de la información. Entonces, hay que llevárselo a las personas de una manera más accesible. A mí se me hace todavía no fácil.

Pregunta 5- ¿Apoya usted esta actividad en su comunidad? ¿Por qué? (*Preguntar a cada participante del grupo de discusión*)

Moderadora

Ok. Y entonces. ¿Apoyaría usted esta actividad en su comunidad?

Diez

Sí, la apoyaría.

Moderadora

¿La apoyaría?

Once

No, me falta información.

Moderadora

¿Por qué la apoyaría usted?

Diez

Yo la apoyaría porque al llevar… al decirle a las personas de que se podría erradicar el mosquito, yo creo que las personas se… necesitarían más información y se orientarían más…

Moderadora

¿Por qué no la apoyaría?

Once

Por lo mismo, por falta de información.

Moderadora

Falta de información. ¿La apoyaría?

Siete

[No se escucha en la grabación*. Contestó luego.]

Moderadora

¿Sí? Deja que él diga… [risas] Él no es tu cabeza. Deja que él diga.

Cinco

Yo digo, éste apoya todo.

Moderadora

Pues déjalo, porque él quiere apoyar.

Siete

Es que todo lo que sea para reducir… hay que apoyarlo. [risas]

Moderadora

Déjalo que lo apoye. ¿Tú lo apoyarías?

Seis

No.

Moderadora

No. ¿Por qué?

Seis

Precisamente por eso mismo, por la falta de información.

Cinco

Por la falta de información.

Seis

Estadísticas… Los pros y los contras. No todo… nos han dicho que sí, que los han utilizado en Brasil, en Colombia. Pero, dónde están los datos estadísticos.

Moderadora

¿Lo apoyaría usted, número cuatro?

Cuatro

No.

Moderadora

Ya tú me dijiste, cinco, que no. ¿Y usted, el número nueve, lo apoyaría? El número nueve.

Nueve

Sí.

Moderadora

Y la número ocho, ¿lo apoyaría?

Ocho

Sí.

Moderadora

¿Y la número tres?

Tres

Yo estaba pensando… sí, sí lo apoyaría, pero, desde ahora, después que tomamos esta información deberíamos que, si para un futuro queremos que el barrio, el pueblo o lo que sea, nos apoyen, es desde ahora ir dando esta información.

Moderadora

Eso es lo que queremos. Empezamos por aquí, seguimos… Pero, aun así, esto no quiere decir que estas cosas van a pasar. Nosotros estamos dando la información. Nuestro rol es darle la información…

Tres

Y nosotros seguirlas.

Moderadora

…ver sus opiniones y educarlos. Ahora de aquí, con esta información nosotros vemos, ‘mira, ellos necesitan estadísticas, ellos necesitan saber qué ha pasado con estos estudios, si hay resultados ya, cuántos resultados hubo. Toda esa información, y la próxima vez que nos reunamos, según vaya saliendo la información se la vamos dando a ustedes. Eso es… para precisamente, para eso es.

Tres

Para que nosotros llevemos… llevemos la voz a los demás.

Seis

Exacto.

Moderadora

Exactamente. Y entonces, ¿usted, apoyaría esta actividad o no?

Dos

Bueno, por falta de orientación e información…

Moderadora

¿No?

Dos

No.

Moderadora

Ok. ¿Y usted, la apoyaría?

Uno

Bueno, eh… sí y no.

Moderadora

¿Sí, por qué? Y, ¿no, por qué?

Uno

Sí, porque si es así… como… porque se oye bonito y se ve viable hacer el mosquito y hacer eso, pero no, porque las personas que yo les voy a explicar, yo debo tener un [choca sus manos] para entonces yo decirles, ‘mira, esto es por esto, esto es por aquello, y esto es por esto’.

Moderadora

O sea, que necesitaría tener más información. Ok.

Pregunta 5a- ¿Piensa que su comunidad apoyaría esta actividad? Sí, No, ¿Por qué?

Moderadora

Y entonces, ¿apoyarían sus comunidades esta actividad?

Uno

Esta actividad, sí.

Moderadora

¿Apoyaría esta actividad su comunidad?

Diez

Bueno, como te digo. Siempre y cuando se les lleve una buena orientación… bueno, nosotros estamos aquí para eso, yo creo que nosotros estamos aquí para eso, para… lo que nosotros aprendamos acá, se lo podamos transmitir a las otras personas. Y posiblemente, verdad, si nos creen, pues…

Siete

Eso iba a decir yo…

Moderadora

Sí, ustedes por aquí.

Siete

Yo me imagino yo hablando de eso en el trabajo, ‘tú estás loco’.

Moderadora

¿Cómo fue?

Siete

Mucha gente va a decir que uno se está inventando esa información.

Moderadora

Que se está inventando esa información.

Diez

Pero tú tienes que decirle que tú te orientaste con una persona que tiene conocimiento de lo que están hablando.

*Anotadores presenciales tienen en sus notas que el participante #7 apoyaría la actividad.

Moderadora

Pregunta 5 - Y tú. ¿Tú crees que tu comunidad apoyaría esta actividad, este método?

Seis

Ay, no sabría decirte, pero a mi entender…

Siete

No.

Seis

No. Porque todos son… de la tercera… ya están llegando a la tercer… si no están, ya están llegando a la tercera edad.

Moderadora

¿Y qué tiene que ver?

Once

Los de la tercera edad son más reacios que las personas…

Siete

Ella no es de la tercera edad y no lo está apoyando.

Seis

No, no, no, pero no importa…

Diez

Yo soy de la tercera edad y yo…

Moderadora

Después te voy a contestar algo. [risas]

Cuatro

Es que como no hay una documentación, una información genuina, como uno dice, para tú presentársela, pues yo voy y se los digo, ‘pero ¿quién te dijo eso?’, ‘muchacha no te dejes llevar por eso, que eso es por llenar un requisito’, y la verdad…

Moderadora

Ok. ¿Y por aquí, cree que su comunidad apoyaría esta actividad?

Uno

Sí.

Moderadora

¿Sí?

Uno

Es como te digo, es cuestión de hablarlo y echar para adelante.

Moderadora

Por acá, por acá, cuéntamelo, cuéntamelo, no lo hable allá. Cuéntamelo a mí. Háblalo para acá.

Siete

Este… se me olvidó… era lo de la tercera edad, las personas de la tercera edad. Muchas ni van a creer eso, porque son bien religiosas y eso no es de Dios. Están genéticamente…

Moderadora

¿Por qué eso no es de Dios?

Siete

Pues porque por la genética.

Seis

Es alterado.

Uno

Porque es un laboratorio.

Siete

Exacto. Nada más por saber qué es eso [hace ruidos de personas refunfuñando].

Seis

Van a empezar a crear, ya quizás tienen otras ideas.

Cinco

Esos son los Iluminati. [risas]

Pregunta 6- ¿Qué otra información necesitaría para entender mejor esta actividad?

[Se contestó en otra sección]

**Parte 3- Cierre de sesión**

Moderadora

Bueno, pues esta fue la última pregunta. Gracias por estar aquí, ya podemos cerrar las…

Coral

Faltan las que están…

Moderadora

Ah, sí, se me olvidó, la última, la última… ah, y se me quedaron dos más. Ok. De todas, de todas, las actividades que les he presentado, todos los métodos. ¿Cuál les gustó más? ¿Cuál usted apoyaría más?

Diez

El de fumigación este… la… con…

Uno

Larvicidas.

Diez

…con la guagua con la que van a fumigar.

Moderadora

Con larvicida.

Diez

Y lo de la dentro de las casas.

Moderadora

Y la de acción residual.

Diez

La de acción residual.

Moderadora

Ok. Ah tú no lo vistes… [refiriéndose a que no estuvo presente sino hasta el final]

Once

No, yo no lo ví.

Moderadora

¿Y tú?

Siete

Yo me voy con lo de la guagua.

Moderadora

Los de la guagua, ¿lo de larvicida?

Siete

Si lo del *spray*. Si porque…

Once

Eso es algo que lo hemos visto ya y no se han muerto.

Siete

Y esto nunca va a pasar, y mucho menos en Puerto Rico. [creo que se refiere a los mosquitos]

Moderadora

¿Tú, cuál apoyarías más?

Seis

La tradicional que sería la eliminación de agua de los envases y también apoyaría la de la fumigación.

Moderadora

La fumigación.

Cuatro

La paila.

Moderadora

La paila.

Ocho

[No se escucha en la grabación. Posiblemente afirmó con algún gesto, no se escuchó que se opusiera]

Moderadora

Las pailas.

Nueve

Fumigación.

Moderadora

Fumigación con larvicida.

Tres

Fumigación.

Moderadora

Fumigación con larvicida. O sea, la que es por… de camión.

Tres

La de larvicida.

Dos

La de camión.

Moderadora

¿La de camión? Ok. ¿Y usted?

Dos

La de camión.

Moderadora

La de camión. ¿Y tú?

Uno

La de camión, la de la casa, la de los drones, y la de sacar toda la porquería de la casa.

Moderadora

Ok. La primeras cinco, digamos.

Pregunta 10- ¿En quién de su comunidad confiarían ustedes para hablar sobre estas actividades?

Moderadora

Bueno, ahora yo les preguntaría. ¿quién en su comunidad…en quién de su comunidad confiarían ustedes para hablar sobre estos métodos?

Tres

El Consejo.

Siete

Yo en una vecina. [risas]

Moderadora

¿Un vecino?

Siete

Un vecino.

Moderadora

¿La vecina?

Siete

No quiso venir.

Moderadora

No, pero quién confiarías tú, que hablara… ¿quién tú piensas que la gente de tu comunidad confiaría para hablar de esto?

Siete

Oh, ok. Ok.

Moderadora

Sí, porque la vecina… [risas]

Siete

No, no, no, no…

Cinco

A ella.

Siete

Yo no voy a decir nada. No, no, sí. Entendí la pregunta mal, fue.

Moderadora

Sí, está bien. Pero bueno, ¿en quién confiarían? ¿En quién confiarían ustedes de que hablara esto en su comunidad?

Diez

Ah, de qué personas…

Siete

Sí, que dieran la orientación.

Moderadora

¿En quién confiarían?

Diez

Bueno, personas que tengan credibilidad y el que sepan del tema.

Moderadora

¿Pero, qué personas tienen credibilidad? Las personas que trabajan en esto. Dice ella. ¿Y ustedes?

Once

Yo, que demuestre que tiene el conocimiento.

Diez

Por eso, ella tiene conocimiento. Eso es lo que está diciendo.

Once

Sí porque tiene la *expertise* y está como a cargo de lo que es el proyecto, porque si uno se lo dice a nuestros vecinos, van a quedar como que…

Moderadora

Ok. ¿Y ustedes?

Seis

Exacto, lo mismo, la gente pertinente, porque la realidad… al que sea le van a ocurrir dudas, que quizás nosotros por no tener el mayor conocimiento no podamos responder. Y para responder con una ignorancia o algo que quizás nosotros no sabemos, pues no, personas que esté preparada…

Moderadora

¿Y usted?

Ocho

Lo mismo alguien que tiene que estar preparada.

Moderadora

Una persona que esté preparada.

Cuatro

Una persona que sepa del tema.

Nueve

Una persona preparada.

Moderadora

Una persona preparada. Ok. ¿Si se lo dice el pastor de la iglesia? ¿Confiarían?

Tres

Sí, por qué no.

Diez

Bueno, si ha cogido la orientación…

Seis

Exacto, si tiene el “expertise”.

Diez

…pues también, pero si no…

Pregunta 11- ¿Cuál sería la mejor manera de hablar a su comunidad sobre estas actividades?

Moderadora

Ok. Y entonces, ¿Cuál sería la mejor manera de hablar en su comunidad sobre estos métodos? ¿Cuál sería la mejor forma?

Uno

Reunión.

Seis

Convocando una reunión.

Moderadora

Reuniones.

Seis

Una reunión en la que, así, se les presente los pros y los contras.

Uno

Una reunión extraordinaria.

Moderadora

Ahorita estaban hablando… este… la número diez estaba hablando de formas de pasar la información.

Diez

Bueno este…

Siete

Radio.

Diez

Radio, televisión, este, redes sociales, aunque hay redes sociales que no tienen mucha credibilidad, pero después que uno le cite los lugares donde ellos puedan entrar… [no se escucha] y recibir más información.

Moderadora

Y él ahorita mencionó como información escrita, ¿me distes a entender?

Once

Sí.

Moderadora

Como que por qué no habíamos dado algún tipo de información antes, para discutir.

Seis

No está de más, pero y cuando surja la duda, ¿a quién se recurre en ese momento? Porque la duda te surge mientras tú vas leyendo. ¿Entiendes?

Moderadora

Pero yo creo que lo que él dijo era, que, el que diéramos la información para que la gente tuviera la oportunidad de leerla y entonces luego…

Cuatro

Cuando vienen aquí, ya tienen conocimiento.

Once

Sí, que la reunión fuera más fluida. Ya que tienen más conocimiento de los temas a tratar…

Moderadora

O sea, que es una combinación de la tuya, más esa parte de que hay información previa para leer para que la gente venga preparada para preguntar.

Uno

Ya vengan con la curiosidad. Con la curiosidad de venir al sitio…

Moderadora

¿Y por aquí?

Tres

Tener una línea telefónica donde se les pueda contestar las preguntas que las personas necesitan.

Moderadora

Una línea caliente. Una línea caliente. Ok. Bueno, pues muchas gracias por haber estado con nosotros y darnos este tiempo, que es muy valioso verdad, porque venir del trabajo a una reunión, está difícil…

*****Fin del audio*****
