# Supplementary material for: Acceptability of emergent Aedes aegypti vector control methods in Ponce, Puerto Rico: A qualitative assessment
Source: PLOS Glob Public Health. 2024 Mar 6;4(3):e0002744. doi: 10.1371/journal.pgph.0002744 (PMC10917327; doi:10.1371/journal.pgph.0002744)
Supplement: S2 Appendix — (ZIP) [file pgph.0002744.s002.zip › S2D_Appendix.docx]

**S2 Appendix. Anonymized Transcripts of Group Discussions (Spanish)**

**COPA Grupo de Discusión con Líderes y Residentes sobre Actividades de Control de Vectores en el Clúster PL**

Fecha y hora- 16 de mayo del 2018; 7pm

Lugar- Centro Comunal AM

Moderador- Carmen Pérez

Anotadores- Coral Rosado, Karla Marrero y Sue Ramos

Transcriptor- Sue Ramos

Participante #1- SL (M) - Verde

Participante #2- SL (M) - Naranja

Participante #3- SL (H) – Violeta

Participante #4- AM(H) - Azul

Participante #5- SA (M) - Gris

Participante #6- VT (M) - Rojo

Participante #7- VT (H) - Blanco

Introducción- Se comenzó leyendo la introducción de la guía de preguntas.

Gris

Una pregunta, o sea, ¿que la información uno no se la puede pasar a otra persona, si no viene a los talleres?

Moderadora

No, yo no puedo hablar de lo que aquí- de las opiniones de ustedes a otras personas de su comunidad. Yo les puedo hacer las preguntas, estas mismas preguntas, a otras comunidades, pero lo que ustedes digan aquí, yo no se lo puedo decir a ellos. ¿Verdad? Ahora, ustedes sí. Ustedes pueden hablar con quién les dé la gana sobre lo que hicimos aquí.

Violeta

En otras palabras, las conversaciones no pueden salir de aquí.

Moderadora

Para nosotros. Pero ustedes sí, usted puede hablar de lo que quiera de este grupo. Pero nosotros no podemos, verdad.

Se continuó leyendo, y los participantes accedieron a ser grabados. Se les preguntó a los participantes si tenían preguntas sobre lo leído. Se pasó a leer el consentimiento informado con los participantes. Todos confirmaron ser mayores de 21 años. Se les preguntó a los participantes si tenían preguntas sobre lo leído. Todos afirmaron no tener preguntas. Y se les preguntó si deseaban participar de la discusión. Todos los presentes afirmaron participar. Se les asignó número a cada participante que estaba presente al momento de comenzar.

Moderadora

Ahora sí que vamos a empezar.

Sue

Moderadora… comunidad…

Moderadora

Ah, tú le das el número y ella que te diga su comunidad. ¿Cuál es su comunidad, número uno (*Verde*)?

Verde

SL.

Moderadora

SL. ¿Y usted, número dos (*Naranja*)?

Naranja

También.

Moderadora

SL.

Naranja

Y él es SL.

Violeta

SL.

Moderadora

SL, el número tres (*Violeta*). ¿Y el cua…?

Azul

AM.

Moderadora

¿Y el cinco (*Gris*)?

Gris

SA.

Moderadora

SA.

Rojo

VT.

Blanco

VT.

Moderadora

Y VT el seis (*Rojo*) y el siete (*Blanco*). Eso se lo ponen aquí [el tag con el número].

Azul

Vistas del Mar no ha llegado y SL [personas que había invitado, pero no fueron].

Sue

Moderadora, ¿puedo decir algo un momentito?

Moderadora

Sí.

Sue

Eso es para que cuando digan algo, digan su número primero y entonces dicen lo que van a decir, para poder identificarlos.

Moderadora

Exacto.

Naranja

Esta es para los mosquitos, el *Aedes aegypti* debe estar por ahí.

Moderadora

*Aedes aegypti*, ahorita voy a tener que ponerme de esto ahorita.

Parte 1- Conocimiento sobre enfermedades transmitidas por mosquitos

Pregunta 1- ¿Qué ha escuchado sobre el dengue, Zika y Chikunguña?

Moderadora

Ok, pues ya que todos sabemos lo que vamos a hacer. Vamos a empezar. Ahora, les voy a asignar el número, que ya se los asigné. Y cada uno tiene su número con su comunidad. Y les voy a preguntar, la primera. Conocimiento sobre enfermedades transmitidas por mosquitos. ¿Qué han escuchado ustedes, sobre el dengue, el Zika y el Chikunguña? ¿Cómo se transmiten y cuán peligrosas son?

Azul

¿Cómo va a ser la respuesta? Individual o en orden, o como el que quiera levantar la mano…

Moderadora

No, el que quiera empezar.

Blanco

Ah, el que quiera.

Azul

El que quiera levantar la mano. [risas]

Gris

Tienes que decir el cuatro (*Azul*).

Azul

Mira… Yo te iba a decir… por lo menos, de eso es lo que hemos escuchado de que son transmitidas por mosquito. De que todas sean peligro de muerte, desconozco. Yo sé que de muchas de ellas lo dicen, y si se complica la condición, puede que todas. Lo que más siempre me ha estado curioso es que todas sean transmitidas por el mismo mosquito. Todas esas enfermedades, es lo que siempre me ha estado más curioso.

Moderadora

Ok. ¿Qué más? ¿Qué más me pueden decir?

Blanco

Que estas…Muchos de los problemas que suceden es que estas enfermedades que se transmiten muchas de las personas que reciben estas picadas de estos animalitos, pues, tienen otras enfermedades y se le complica más la cosa a la persona esa. Tienen una complicación.

Moderadora

Ok. Exactamente. O sea, que, para usted, eso es un problema, verdad, porque entonces ahí es que se puede poner la cosa como más peligrosa.

Blanco

Más peligrosa. Más peluda.

Gris

Yo creo, que es que uno tiene que mantener el patio bien limpio y fuera de agua, que no se contaminen para que los mosquitos no sigan ahí. De ahí es donde se salen las enfermedades.

Moderadora

Ok. Exacto. ¿Qué más? ¿Estamos bien hasta ahí?

Verde

Yo creo que también de las plantas, que uno tiene muchas plantas, el agua se acumula ahí eso también crean mosquitos, y uno debe tener cuidado ahí también.

Moderadora

Ok. Rafa, ¿tú podrías ser tan amable de darle una botellita de agua a cada persona? Gracias. Pueden coger galletitas.

Verde

Mira, ella cogió galletitas.

Naranja

Yo creo que de todas el más peligroso es también cuando da el dengue porque el hemorrágico es bien malo.

Moderadora

Exacto.

Naranja

Eso causa muerte. [se reparten galletas y agua]

Moderadora

Ok. Y entonces, estaba diciendo la señora que… este… ¿puede repetir?

Naranja

Sí que… este… De todos esos mosquitos, las enfermedades que transmite, el más malo yo creo que es el dengue hemorrágico. Que ese es el que causa muerte, el hemorrágico.

Moderadora

Ok. Muy bien.

Blanco

También otra cosa que nos preocupa en este caso. Que las autoridades del gobierno, necesitamos la ayuda de ellos. Muy grande. Porque hay cosas que nosotros podemos prevenir, pero necesitamos la ayuda también del gobierno. Usar cierto equipo para poder…

Moderadora

Y de eso es lo que vamos a hablar, pero la número seis (*Rojo*) quiere decir… de eso es que vamos a hablar.

Rojo

También es mantener los patios fuera de las hierbas y eso; porque mientras más suben, la hierba, como pasa en donde nosotros vivimos, ahí se cobija el mosquito.

Moderadora

Claro.

Violeta

Es mantener el área limpia.

Moderadora

Muy bien. Exactamente.

Violeta

Y todas tenemos exactamente todo correcto. Para que no haya ninguna clase de enfermedad, ni vengan los mosquitos, ni agua sucia.

Moderadora

Exactamente.

Pregunta 1a- ¿Cómo se transmiten?

[se contestó más arriba]

Pregunta 1b- ¿Cuán peligrosas son?

[se contestó más arriba]

Parte 2- Reacciones a las actividades de control de vectores

Moderadora

Entonces, muy bien, todas esas respuestas han sido muy buenas. Ahora, para comenzar les voy a mostrar un video que explica el ciclo de vida del mosquito *Aedes aegypti*. Esta explicación les ayudará a entender mejor cómo funcionan las actividades para el control de mosquitos. Y vamos a empezar aquí.

Verde

Ay que lindo.

Moderadora

Van a ver esto, esto está divino.

Verde

Está bello.

Moderadora

Bueno. Ahí está, dice, el mosquito *Aedes aegypti* macho no pica, pero la hembra pica a una persona para obtener sangre y producir sus huevos. Mira cómo está picando ahí ese brazo.

Azul

Está chupando.

Moderadora

Mira cómo se le va llenando la barriguita de sangre, cómo va picando. El mosquito hembra pone sus huevos en las paredes de cualquier envase con agua acumulada, dentro o fuera del hogar. Esos huevos pueden durar hasta ocho meses pegados en las paredes del envase. Cuando los huevos se sumergen en el agua nacen las larvas. Mira la larva que bonita. [risas] Mira que lindo.

Violeta

¿Bonita?

Moderadora

Y entonces, las larvas viven en el agua donde se alimentan y se convierten en pupa en aproximadamente cinco días. Estas son las larvas, mira, tienen un sifón donde respiran. Suben y ese sifón las ayuda a respirar. A coger oxígeno, ven. Y entonces, de dos a tres días… se convierten en pupas y de dos a tres días, las pupas se convierten en mosquitos adultos. Estas son las pupas. ¿Ven cómo va saliendo el mosquito? De la pupa. Se toma su tiempo, verdad, porque le cuesta trabajo salir.

Verde

Sí, pero no tardan en picar a uno.

Moderadora

Míralo como sale. Muchacho sal ya. Ahí salió, ve, las patitas las tenía pilladas. [risas] Entonces, se toma de siete a diez días desde que el huevo de mosquito se convierte en un mosquito adulto. Ven, vieron el ciclo. Cómo pasa. El ciclo del mosquito. Y ahí está la mano picada, bendito, debe tener una roncha.

Naranja

Una víctima. [risas]

Moderadora

Se están desarrollando muchas formas de reducir el número de mosquitos en el ambiente. Algunas acciones, pueden ser realizadas por los residentes y las comunidades, y otras pueden ser realizadas por profesionales de control de mosquitos o el gobierno. Ahora les mostraré unos dibujos que describen las actividades que se podrían realizar y cómo podrían ayudar. Luego, les voy a hacer una serie de preguntas para obtener su opinión sobre estas actividades.

Actividad #1 – Reducción de fuentes de mosquitos

Moderadora

Esa es la primera. Leo. Esta se llama… esta… este método de reducir los mosquitos se llama, reducción de fuentes de mosquitos. Y dice, la reducción de fuentes es la eliminación, déjame irme para acá, la eliminación de lugares donde los mosquitos ponen sus huevos. El municipio y su comunidad trabajarían juntos para eliminar, vaciar y recoger los envases con agua acumulada que puedan ser criaderos de mosquitos en áreas públicas. Dentro de su hogar y patio, usted cubriría, vaciaría o eliminaría los envases que acumulan agua como los tiestos, las latas, y gomas y desagües. ¿Qué ustedes ven ahí? En ese dibujo.

Verde

Una mujer embarazada…

Blanco

Una goma… embarazada…

Naranja

Botando el agua.

Verde

Botando el agua que se acumuló.

Moderadora

Botando el agua. ¿Qué más ven?

Verde

Los niños están…

Gris

Un hombre llevando las gomas.

Moderadora

Un hombre eliminando la goma. ¿Qué más?

Verde

¿Esos son niños?

Moderadora

Sí. Personas de la comunidad. Sí, niños.

Verde

Sí, están recogiendo…

Azul

Eliminando envases. Eliminando envases.

Moderadora

¿Y qué más? Falta una.

Blanco

Tapando el zafacón.

Verde

Tapando el zafacón.

Violeta

Claro.

Moderadora

Tapando el zafacón. Verdad, que son las actividades que dicen aquí, verdad. Eliminaría los envases que acumulan agua, como los tiestos, las latas, gomas y desagües. Ok. Ahora les pregunto. ¿Entendieron esa explicación? ¿Alguien tiene alguna duda de esa explicación que leí? ¿No? Ok.

Pregunta 2- ¿Es esta una actividad nueva para usted o es algo que ya había escuchado antes? Si la escuchó, ¿dónde la escuchó?

Moderadora

¿Esta… este método, es nuevo para ustedes o es algo que ya habían escuchado antes?

Blanco

Ese método es viejo.

Naranja

Ya lo habíamos escuchado y lo hacemos también en la casa.

Verde

Ya lo había escuchado.

Moderadora

Todos lo hacen en sus casas. Es viejo ese método. ¿Dónde lo habían escuchado?

Blanco

Por la televisión, por radio.

Verde

Ajá. La radio.

Azul

En anuncios.

Naranja

Orientaciones que dan, ya sea por televisión o en la radio que lo dicen también...

Verde

A veces hasta en los anuncios.

Naranja

…evitar que se acumulen, el mosquito.

Moderadora

Ok. Y ella dice que lo dicen en los anuncios.

Verde

A veces los anuncios también te lo dicen.

Gris

Las charlas que dan también.

Moderadora

En las charlas.

Gris

Aquí mismo dieron una charla de eso.

Moderadora

Charla. ¿Quién la dio?

Gris

Ay, yo no me acuerdo… pero era de la universidad.

Moderadora

¿De la universidad la dio? Ok. De la Escuela de Medicina.

Naranja

De la escuela de medicina, también aquí dieron una.

Verde

Aquí dieron una también. Sí.

Azul

Y en los grupos focales que se hicieron con CDC también se habló eso.

Moderadora

Ok. Los grupos focales que se hicieron con nosotros.

Verde

Aquí se hizo una que yo vine.

Naranja

Uno fue un doctor, y habló aquí en este centro comunal también….

Verde

Y había un muchacho que estaba diciendo cómo hacer una trampa o algo…

Gris

Las trampas, sí.

Verde

…para los mosquitos.

Pregunta 3- ¿Cree que esta actividad reduciría el número de mosquitos en su comunidad? ¿Por qué?

Moderadora

Ah ok. Ok. Muy bien. ¿Cree usted que esta actividad reduciría el número de mosquitos en su comunidad?

Verde

Yo creo que sí.

Naranja

Sí. Si todos cooperan…

Verde

Si todos cooperan…

Gris

Si todo el mundo coopera, sí.

Moderadora

¿Por qué?

Gris

Porque de qué vale que yo limpie mi casa, mi patio y eso, si el vecino tiene basura, tiene escombros y todo. De nada vale que… Tenemos que estar todos por igual. Ayudarnos todos.

Moderadora

Ok. Y los que dijeron que sí, que funcionaría. ¿Por qué funcionaría?

Naranja

Porque así evitaríamos que se nos acumulen los mosquitos. Si mantenemos, como dice, las áreas limpias que no se acumule agua. Mantener envases limpios, y todo eso, pues eso ayudaría.

Moderadora

¿Usted quería decir algo? ¿No? Está bien.

Blanco

Funcionaría en cierto aspecto, por la siguiente razón. Yo puedo hacer eso en mi casa, pero la vecina que tengo al lado no lo hace. ¿Qué sucede? Yo los elimino, pero el vecino que no lo hace, vuelan de casa de ese vecino para mi casa. Tendría que ser en cadena, que el vecino y el otro y el otro, lo puedan hacer. Porque si lo hago yo y no lo hacen los vecinos, pues...

Moderadora

Pero ahí dice que la comunidad se podría unir para hacerlo.

Naranja

Por eso, por eso te digo, si todos cooperan…

Verde

Si todo cooperan.

Naranja

…y todos lo hacen se podría evitar.

Verde

Si es como el señor dice, ¿de qué vale que yo limpie? Si aquellos… entonces los mosquitos de allá se van a venir a mí... No vale la pena.

Violeta

No vale la pena, porque entonces la acumulación está en el otro lado del hogar. Entonces esos mosquitos vuelan al sitio donde uno está.

Azul

Mi teoría siempre ha sido, y partiendo de la premisa, que es lo que se menciona ahí, que… vamos a eliminar todos los mosquitos, o sea, vamos a eliminar todas las formas que hay o que suceden para que los mosquitos se reproduzcan, y es eliminando envases, en todas esas cosas donde los mosquitos se meten porque hay agua acumulada. Y si partimos de la premisa, de que la idea es que se eliminen todo, no que yo la elimine y el vecino no, sino que busquen una estrategia para que lo elimine todo el mundo. Pues yo entiendo que, si eliminamos todas las posibilidades de que ese mosquito tenga un *nursery* para poner sus huevos, no va a haber huevo que poner. ¿Por qué? Porque no tienen dónde ponerlo. Partiendo de la premisa de que se haga una estrategia para que todo el mundo, no yo, sino, todo el mundo elimine los envases y que no haya envases. Y te lo digo yo que vivo en el sector, que todos los que estamos aquí, es dónde más envases hay, porque en las parcelas es donde más porquería se guarda. Y yo vivo en este sector. Es donde más se acumula, es aquí en las parcelas, donde más se acumula todo eso. Ahora, hay que partir de la premisa que la idea y estrategia es eliminar todos esos envases de todas esas personas que no lo quieren hacer, pues hay que buscar una forma de hacerlo.

Moderadora

Y, ¿se uniría la comunidad para hacer como una limpieza?

Azul

Yo entiendo que se podría hacer, pero, yo te voy a decir una cosa. Yo estoy en la junta de aquí de AM, y yo una vez llamé al municipio para pedirle una campaña de limpieza donde yo quería que se empezara con una orientación, que vinieran, porque había unos muchachos de la universidad que estaban en ese ambiente y querían participar para orientar a las personas en ese aspecto, qué había que hacer y por qué era necesario que se eliminaran los envases. Y el municipio me dijo que no tenía tiempo para eso, que no podían hacerlo. Que no tenían personal, que ya lo habían hecho…

Moderadora

Ok. ¿Qué beneficios o…? Ajá, usted quería decir algo.

Rojo

En cuanto a lo que dice [apellido del participante cuatro (*Azul*)], yo lo entiendo también como lo que está pasando en la comunidad de nosotros. En la comunidad de nosotros hay un…

Blanco

El río.

Gris

Canal.

Azul

Eso es un charco allá.

Rojo

Ya esa agua no corre al menos que haya mucha lluvia.

Azul

Está estancada ya. O sea, que es estancada.

Rojo

Hay mucho escombro, hay mucho coliforme, bueno, muchísimas cosas. Entonces, eso se mantiene siempre así. Uno va al municipio, no le hacen caso a uno. Entonces cuando llega la temporada de los mosquitos, nosotros tenemos que encerrarnos rapidito.

Blanco

Y ya están llegando.

Rojo

Tú sales y los mosquitos eso es uno aquí, aquí y acá. Empiezas a sacudirte.

Moderadora

¿La gente tira basura ahí?

Rojo

También. Hay gente que cuando necesitan…

Gris

Limpiar el patio.

Rojo

Sí. Van para allá.

Blanco

En bolsitas de noche. Que nadie los ve.

Rojo

Entonces, ahí tiran… este…

Blanco

De todo.

Rojo

…este… ¿cómo se llama? De esto de…jugos, o de lo que sea, entonces cuando llueve eso coge agua y ahí se va formando la…

Blanco

También tenemos un pequeño problema que es este, que usted invita a la comunidad para una actividad como esta, y mire los que venimos, los que estamos… los que nos gusta asistir a esto somos los que… y usted hace una red de reuniones venimos más que los mismos. Y usted invita a la gente como me pasa a mí en la urbanización donde yo vivo, y la gente no… hablando vulgarmente hablando, no le importa nada, ve. Porque a ellos no le ha pasado eso. Pero no vienen. Y ese es el problema que tenemos, como [apellido del participante cuatro (*Azul*)] tiene también que él invita la gente y no vienen a las reuniones.

Verde

Pasa en todas las comunidades.

Naranja

En todos los sitios, porque a mí me pasa igual.

Blanco

En todo, sí. Ese, es aquel de puertorriqueños, hablando vulgarmente.

Verde

Si decimos que van a regalar algo, ahí van.

Violeta

Sí, muela.

Verde

Muela.

Blanco

Mucha gente se ocupa y vienen a las reuniones, pero mucha gente, pues…

Verde

Les da lo mismo.

Blanco

Y como ahora están dando la novela esta…

Verde

Las novelas…

Naranja

Total, todas esas novelas turcas…

Verde

Están repitiendo *Fatmagul* (novela). No se las quieren perder.

Pregunta 3a- ¿Qué beneficios o ventajas tiene esta actividad para usted?

Moderadora

Entonces, ¿qué beneficios o ventajas tiene esta actividad para ustedes?

Violeta

Nos aclara los pensamientos…

Moderadora

No, este método, este método.

Azul

Ese método, pues que elimina…

Violeta

Ese método, pues que aclara unos pensamientos que están oscuros y se ponen claros. Y así vemos cual es la realidad de hoy en día lo que puede suceder.

Moderadora

Ok. ¿Y número cuatro (*Azul*)?

Azul

Nada, yo lo que pienso es que esto lo que nos ayuda es a eliminar lo que queremos, los mosquitos. Porque si eliminamos los sitios donde ellos van a poner los huevos, no hay dónde poner huevos, son menos mosquitos.

Pregunta 3b- ¿Qué desventajas o dificultades le ve a esta actividad? ¿De qué forma se podrían solucionar esas dificultades?

Moderadora

Ok. Y, ¿qué desventajas o dificultades le ve a esta actividad? Que ya lo hemos hablado un poquito.

Azul

Lo que han dicho todos, que mucha gente no le importa, eso es parte… la gente tiene el problema, pero no quieren aportar.

Gris

El mismo municipio tampoco ayuda a resolver el problema.

Moderadora

Y, ¿de qué forma se podría solucionar esa dificultad? ¿De qué forma se podría solucionar?

Naranja

Es que por más que tu hables con las personas de que cooperen haciendo eso, algunos te van a decir que sí, ‘sí lo voy a hacer’, pero después no lo hacen. Y ahí está la dificultad de que tu tratas de convencer a la gente, ‘vamos a tener que hacer esto para que no haya mosquito, trata de no tener esa agua acumulada’. Pero a la hora de la verdad, cuando tú vas, pues sí, quizás tiene un balde ahí de hace tiempo con agua, o las matas, como dice mi hermana. Pues entonces, no cooperan. Si no hay cooperación… Vamos a seguir en las mismas.

Verde

Ahora mismo, donde nosotros vivimos, en la parte de atrás cuando llueve mucho, a la parte de atrás se acumula agua. Que se lo hemos dicho ese problema…

Naranja

Ajá. Tenemos ese problema que se nos está acumulando agua en el terreno de atrás. Y eso, la otra vez mi esposo fue para ver… no sé para qué él iba para allá, y se puso hasta unas botas y me dijo, ‘mira, hasta aquí me llega el agua’. Hasta la rodilla.

Pregunta 4- ¿Cuán posible es realizar esta actividad en su comunidad para reducir el número de mosquitos? ¿Por qué?

Moderadora

Ok. ¿Cuán posible sería realizar una acti… un método, este método, una actividad en… como esta en su comunidad para reducir el número de mosquitos? Si les dijéramos, ‘vamos a hacer una campaña de limpieza en su comunidad’, ¿cuán posible sería hacerlo?

Naranja

Si fue [nombre del participante cuatro (*Azul*)] y no lo ayudaron mucho, el municipio…

Azul

Bueno, yo pienso que si las agencias que nos pueden ayudar se envuelven lo podemos hacer. Pero a mí me interesa más que una campaña de limpieza, una campaña acompañada de orientación. Porque ir a recoger la basura y te la llevas… yo entiendo, y fue lo que yo le pedí a ellos, yo quiero que me den, por ejemplo, una semana antes, yo quiero entrar en la etapa de ir casa por casa explicando a las personas de qué es lo que vamos a hacer y orientarlos y después se hace la campaña. Que debe ser un proceso de campaña, pero de orientación. Pero, nosotros quizás tenemos recursos humanos, no son muchos, pero el otro recurso no lo tenemos.

Gris

Yo estoy de acuerdo con [nombre del participante cuatro (*Azul*)], porque el problema de eso es que uno puede hacer una campaña de limpieza, suponer, un sábado, la gente no tiene esa conciencia y vuelven en la otra semana y vuelven y sacan basura, pues esta uno todo el tiempo con lo mismo, lo mismo, lo mismo, lo mismo.

Moderadora

O sea que para usted sería que en vez de que… que puede pasar que no todo el mundo saque todo lo que tiene o que saquen y sigan sacando.

Gris

No lo saquen y después vuelvan otra vez. Claro. Porque eso sucede muchas veces.

Azul

Y no sacando, acumulando en los patios también. Nosotros somos así. Somos parceleros.

Moderadora

Y ahora le pregunto… [risas]

Azul

Pues, es que es la realidad.

Moderadora

Es cierto.

Azul

Es que es así.

Moderadora

Parcelas. Claro.

Pregunta 5- ¿Apoya usted esta actividad en su comunidad? ¿Por qué? (*Preguntar a cada participante del grupo de discusión*)

Moderadora

Ahora le pregunto a cada uno individual. ¿Apoyaría usted una actividad como esta en su comunidad?

Verde

Claro que sí.

Moderadora

¿Usted la apoyaría?

Naranja

Sí.

Moderadora

¿Y usted lo apoyaría?

Violeta

Bueno, de hecho, tienen que apoyar.

Azul

Yo la quiero.

Moderadora

Usted la quiere. ¿Y usted?

Gris

Sí.

Moderadora

También. ¿Usted?

Rojo

Sí.

Moderadora

¿Y usted?

Blanco

[Anotadora tiene en sus notas que participante #7 (Blanco) apoya la actividad.]

Moderadora

Ok.

Rojo

Nosotros hace poco… hace… ¿cómo cuánto hace que nosotros…? nosotros hicimos una para limpiar…

Blanco

El río.

Rojo

El caño. Mientras ellos trabajaban nosotros le dábamos el agua, todo, todo, todo.

Moderadora

Muy bien.

Pregunta 5a- ¿Piensa que su comunidad apoyaría esta actividad? Sí, No, ¿Por qué?

Moderadora

Y entonces. ¿Piensa usted que su comunidad apoyaría esta actividad?

Verde

Yo creo que mitad, y mitad. Algunos sí. Algunos no.

Moderadora

¿Por qué?

Verde

No sé, tal vez no, como dice la señora, a lo mejor no le interesa estas cosas. A lo mismo.

Naranja

Yo también, yo creo que si… Para mí, yo creo que la mayoría sí lo apoyaría, sería un poco mínimo los que no estarían… Personas mayores que a veces no pueden ir hasta la reunión o no tienen tal vez la misma fuerza de eliminar las cosas en sus propios patios. Porque hay… yo tengo personas que son viejitas y no pueden ni siquiera alzar un balde.

Moderadora

Ok ¿Y ustedes? ¿Piensan que sus comunidades apoyarían esta actividad?

Azul

Yo creo que sí.

Violeta

Bueno, eso es dependiendo.

Moderadora

Dependiendo.

Azul

Pero bien, bien organizada.

Violeta

Hay unos que les gustan y otros que no les guste.

Naranja

Que si no les gusta la limpieza…

Moderadora

Usted dice que bien organizada.

Azul

Sí. Bien organizada porque yo siempre, por lo menos cuando… por ejemplo, si el municipio me dice, ‘mira, vamos a hacer una feria de salud’, en la cancha, por ejemplo, me lo tienen que decir una semana antes para anunciarlo. No me vengas el mismo día a decírmelo o el día antes, porque tú no vas a mover la gente de un día para otro.

Moderadora

La gente no está esperando sentados en sus casas, verdad.

Azul

Si se hace bien organizado, yo creo que sí.

Pregunta 6- ¿Qué otra información necesitaría para entender mejor esta actividad?

Moderadora

Ok, y entonces. ¿Qué otra información necesitaría para entender mejor esta actividad? O sea, para apoyar o entender mejor este método. ¿Qué información necesitarían?

Gris

Bueno, si fuera que se va a hacer una campaña, pues, este… hacer un *flyer* y darlo a la comunidad para que se enteren lo que hay.

Naranja

Promociones.

Moderadora

Ok. Que se necesitaría un *flyer*, él dijo que también quería orientación.

Gris

Sí. Pues, también.

Violeta

Correcto.

Moderadora

Ok. Orientación. ¿Cómo qué?

Azul

Y tener claro cuál va a ser la participación y el apoyo de las agencias pertinentes. Porque si no sabemos cuál va a ser el apoyo, nos tiramos y después…

Pregunta 7- ¿Considerarían realizar ustedes mismos esta actividad?

Moderadora

Yo creo que ustedes ya me han contestado que ustedes considerarían hacer esta actividad y que ustedes lo están haciendo. Todos aquí me dijeron que están recogiendo semanalmente, ¿verdad? Eso fue lo que escuché ahorita que todos dijeron.

Pregunta 8- ¿Qué les haría difícil realizar esta actividad?

Moderadora

¿Qué les haría difícil realizar esta actividad?

Rojo

¿Difícil?

Moderadora

Ajá. ¿Qué les haría difícil?

Rojo

Para mí nada, porque nosotros siempre estamos… ¿cómo se llama? Para ayudar también. Que, si nos ocupan, nosotros estamos ahí. Porque nosotros somos dos solos, no trabajamos.

Blanco

No digas eso. [risas]

Moderadora

No trabajan afuera, asalariados. Verdad, asalariados.

Rojo

Yo trabajo sí. En mi casa.

Moderadora

No trabajan asalariado. [risas] Ok, ¿alguien le haría esta actividad… se le haría difícil? ¿Qué le haría difícil de esta actividad? No.

Pregunta 9- ¿Hay algo que podría ayudarles a realizar esta actividad de manera más fácil?

Moderadora

Ok. ¿Hay algo que podría ayudarles a realizar esta actividad de manera más fácil?

Naranja

Mas fácil que lo que esta explicado ahí, yo encuentro que no.

Azul

Lo que lo haría más fácil es el apoyo de las agencias.

Moderadora

El apoyo. Lo que lo haría más fácil es el apoyo.

Verde

Aquí lo difícil es tratar de convencerlos. Y lo más fácil es que ya…

Violeta

O que vengan a las reuniones.

Pregunta 9a- ¿Necesitarían más información?

Moderadora

¿Se necesitaría más información? Usted dijo que querría orientación.

Azul

Orientación a las personas, a la comunidad. Ahora, traerlas aquí, imposible. Eso hay que ir casa por casa.

Moderadora

¿Casa por casa sería una manera?

Azul

De verdad, yo creo que es la única.

Violeta

Creo que es la única manera, fíjate.

Gris

O puede ser altoparlantes.

Azul

Y en eso, para mí, es lo que haría un poco más difícil es el tiempo. Porque ir casa por casa pues no es cuestión de un día.

Moderadora

Ay sí…

Azul

Ya eso sería… Y no lo hacen cuatro o cinco personas, ni doce, ni quince.

Moderadora

Aján.

Pregunta 9b- ¿Necesitarían más adiestramiento?

[No se hizo la pregunta]

Actividad #2 – Aplicar larvicidas al agua acumulada

Moderadora

Bueno. Pues vamos a la próxima. Vamos a la próxima porque el tiempo va corriendo. Aplicar larvicida al agua acumulada. Los larvicidas son pesticidas que se usan para matar las larvas antes de que se conviertan en mosquitos adultos. Todos ustedes vieron en el video la larva, verdad. Los larvicidas se pueden aplicar de diferentes maneras, en gránulos, en tabletas, o líquido. La aplicación de larvicidas puede reducir la cantidad de mosquitos si se aplica correctamente. Los larvicidas no afectan a las personas, ni a sus mascotas, ni el ambiente, si se siguen las instrucciones de la etiqueta. Los larvicidas no deben usarse en el agua potable para consumo humano o animal. Se requiere aplicar la cantidad correcta según las instrucciones de la etiqueta. Se requiere reaplicarse cada cierto tiempo. Y no alcanza lugares que estén ocultos donde los mosquitos se reproducen. Aquí está la foto del larvicida en tabletas, verdad, que parecen unas donitas y dónde se pueden echar. Y también está el larvicida en grano que, el señor lo está echando, ¿dónde?

Naranja

¿Qué es eso, como una jardinera?

Moderadora

Esto es un pozo muro. Un pozo muro, y ¿esto es qué?

Azul

Una alcantarilla.

Verde

Como una canasta.

Moderadora

Una alcantarilla.

Verde

Mira, que desde acá vi una canasta.

Moderadora

Sí.

Pregunta 2- ¿Es esta una actividad nueva para usted o es algo que ya había escuchado antes? Si la escuchó, ¿dónde la escuchó?

Moderadora

Y ahora le pregunto, ¿es este método uno nuevo para ustedes?

Naranja

Para mí, sí.

Violeta

Para mí sí.

Moderadora

Bueno, ¿entendieron lo que les leí?

Violeta

Sí.

Naranja

Sí.

Moderadora

¿Tienen alguna pregunta de lo que les leí?

Violeta

Bueno, yo no tengo dudas.

Moderadora

¿Y usted? ¿Todos estamos bien? ¿Entendieron?

Rojo

Sí…

Moderadora

Ok. ¿Es esta actividad nueva para ustedes? ¿Este método es nuevo? Me dijeron que sí. ¿Verdad?

Naranja

Bueno, ahora que lo veo, pero ya tú me lo habías dicho en la charla que tuvimos allá.

Azul

Nuevo en el sentido de que estas usando la palabra larvicidas, pero que se usen insecticida y estos químicos para matar mosquitos eso siempre se ha hecho. Lo hacia el gobierno.

Moderadora

Pero hay una diferencia entre los insecticidas y los larvicidas. ¿Qué específicamente del ciclo de vida del mosquito matan los insecticidas?

Azul

El adulto, será.

Moderadora

El mosquito adulto. ¿Y el larvicida qué mata?

Azul

Por eso, la larva. Por eso, eso es lo que es diferente, matarlos antes de que nazcan. Es un aborto.

Moderadora

Eso la cosa, eso es. Algo parecido. Y entonces, algunos dijeron que escucharon de esta actividad.

Azul

Yo no. Por lo menos…

Moderadora

¿No había escuchado de…?

Azul

Eliminar larvicidas así, no había escuchado mucho de eso.

Moderadora

¿Usted había escuchado?

Gris

Sí.

Moderadora

¿En dónde?

Naranja

Yo lo había oído, pero fue cuando tú me distes la…

Gris

Cuando dieron la charla. La otra charla.

Naranja

…el cuestionario. Ahí fue que yo aprendí muchas cosas.

Moderadora

¿Cuándo dieron la otra charla?

Gris

Sí.

Moderadora

Ah, ¿cuándo ustedes vinieron ese día?

Gris

No, ellos no estaban.

Azul

Sería la Escuela de Medicina.

Gris

Sí, de la Escuela de Medicina vino un doctor.

Moderadora

Ok… Dr.…

Gris

Un profesor, un profesor.

Moderadora

Ok. Y ustedes, ¿habían escuchado?

Rojo

No…

Moderadora

No. Ok.

Pregunta 3- ¿Cree que esta actividad reduciría el número de mosquitos en su comunidad? ¿Por qué?

Moderadora

¿Cree que esta actividad reduciría… este método, perdón, reduciría el número de mosquitos en su comunidad?

Gris

Aján.

Verde

Aja.

Moderadora

Sí. ¿Por qué? ¿Cree usted que esto reduciría el número de mosquitos en su comunidad?

Blanco

Yo creo que no lo reduce.

Moderadora

¿Por qué no?

Blanco

Por que como le expliqué anteriormente. Mientras una persona haga un trabajo bueno eliminándolos, y otra persona no lo haga, estamos en lo mismo.

Moderadora

Pero esto es para echárselo al agua. Las personas ya no tienen que botar los envases.

Azul

Lo que pasa es…

Naranja

Cuando se lo eche…

Azul

Lo que pasa es que si tú tienes un área donde hay mil mosquitos, mil larvas, y metes larvicidas y mueren 500, estas reduciendo. Está reduciendo…

Blanco

En la casa de uno, en la del vecino no.

Azul

Ahora, lo que por lo menos yo personalmente, no estoy muy de acuerdo con ese sistema, porque yo creo que no es matar al larvicida [larvas], es eliminar el contenedor de agua que no debe estar ahí para que no haya larvas. Punto.

Blanco

Exacto.

Moderadora

Yo… ok, entonces, fíjese que cosa más interesante. Usted dice que… eh… como quiera la gente lo tendría que hacer. Y que no todo el mundo lo va a hacer. ¿Eso fue lo que yo entendí que él dijo?

Blanco

Sí exactamente…

Azul

O sea que, aunque le den la oportunidad de hacerlo, no lo harían. Pero es que es eliminar el envase y ya, que no haya envases con agua que no debe haber. Que no haya larvas. Por lo menos es lo que yo pienso.

Moderadora

Sí, no. Estamos muy bien.

Verde

Para eso estamos aquí, ¿no?

Moderadora

Estamos muy bien. [risas]

Gris

Yo creo que antes el gobierno lo que hacía era, que pasaba las guaguas por… y entonces, fumigaban. Fumigaban todas las comunidades. Yo creo que eso lo deben de hacer para eliminar.

Moderadora

¿Por qué? ¿Por qué eso? ¿Y no hacer esto? No hacer larvicida. Y no echarle larvicida en la casa…

Gris

Porque yo creo que eso cubre más los sitios.

Rojo

Sí, pero eso yo entiendo el tirar el humo ese, para los mosquitos, eso se va rápidamente porque el viento se lo lleva todas esas cosas, pero ¿los que están dentro de nuestras casas?

Moderadora

Ajá.

Azul

No, yo pienso, también… o sea, no podemos dejar que nazca el mosquito, para después matarlo. Si lo podemos eliminar como larvas, es mejor eliminarlo. No esperar a que esté adulto para entonces fumigar, porque…Vamos a evitar que nazca y punto, y si no nace, no tenemos que fumigarlo. Yo creo que es más efectivo.

Verde

O sea, ¿Lo que quieres decir es q este sistema no funcionaría?

Naranja

Para él.

Verde

¿Para usted?

Azul

No es que no funcione. Si tu coges un envase con larvas, las vas a matar. Pero yo lo que pienso es que hay que evitar usar larvicida y la única forma que lo puedes eliminar es que no haya agua estancada. Ahora, hay un problema que es con la… que, en eso yo entiendo, no sé si vamos a llegar a ese punto, pero es lo que yo iba a mencionar, y es que yo pienso que las agencias de gobierno en eso pueden ayudarnos, no sé si ustedes aplican como agencia de gobierno que pueden bregar, entonces, en eso es que pueden ayudarnos. Y no sé si ustedes aplican como agencia del gobierno que pueden bregar en eso, y es que en las áreas públicas como en el caso del charco, que eso es un charco ahí, porque eso era río antes, y es agua estancada… y en sitios como el área de ella que se acumula agua, y en muchas parcelas aquí que se acumula agua, pues esas áreas hay que bregar con eso. Porque ahí tú no puedes ir a virar un envase, eso es que está el agua estancada. Y en esos casos, pues sí, haría falta que eso… que haya una operación agresiva para eliminar esas áreas que son públicas.

Pregunta 3a- ¿Qué beneficios o ventajas tiene esta actividad para usted?

Moderadora

O sea, que entonces, un beneficio o desventaja de esta actividad para usted sería, ¿cuál?

Azul

Desventaja en este sentido es que vamos a mantener los envases, o sea, no nos vamos a preocupar porque la gente deje los envases. Y si, por ejemplo, viene un envase… y la gente se acostumbra a dejar los envases donde quiera, deja los envases con agua, que se acumule agua y si en alguno de ellos se va el efecto del larvicida, puedes estar seguro que van a nacer más mosquitos.

Moderadora

Sí, pero yo le estoy preguntando ahora… eso, esa es la próxima pregunta y gracias por contestármela. ¿Cuál es el beneficio o ventaja que usted le ve a esto?

Azul

El beneficio es eliminar la larva- el mosquito antes de que nazca. No dejarlo nacer. Que no nazca punto.

Naranja

Yo estoy de acuerdo con él.

Violeta

Estoy de acuerdo con él.

Blanco

El beneficio es que es un método fácil de las personas hacerlo, la familia... Es más fácil eso.

Moderadora

Es más fácil que, ¿qué? ¿Con relación a qué?

Blanco

Bueno, es más fácil que llamar al municipio para que use el sistema… otro sistema. Sino que la hace la persona propia.

Azul

Va a funcionar mientras tengas larvicida. Una vez se te acabe y vayas a buscarlo y te cueste tres pesos no lo vas a comprar. Yo no lo voy a comprar. [risas]

Moderadora

Ah, que el costo también. El costo también sería una desventaja.

Azul

Mientras lo tengas y te los faciliten lo vas a usar. El día que se acabe…

Blanco

Exacto.

Verde

Pero es que antes fumigaban…

Azul

Le echo Vell [no sé si se escribe así, pero creo que se refiere al jabón de lavar platos]

Verde

… pero ya no fumigan.

Violeta

Ya no fumigan.

Verde

Ya no fumigan.

Violeta

Tú sabes, que en eso sí tiene razón.

Azul

Lo que pasa…

Moderadora

Vamos a llegar ahí, a otro tipo de fumigación vamos a llegar dentro de poco.

Azul

Ah, ¿vas a hablar de fumigación? ¿Ahora?

Moderadora

Sí. Ya mismo.

Azul

Sí, porque yo…

Moderadora

Y entonces, ¿Cuán posible…?

Azul

…he escuchado problemas de eso…

Moderadora

Pero no es la fumigación… vamos a llegar ahí.

Pregunta 3b- ¿Qué desventajas o dificultades le ve a esta actividad? ¿De qué forma se podrían solucionar esas dificultades?

[Se contestó en otra sección]

Pregunta 4- ¿Cuán posible es realizar esta actividad en su comunidad para reducir el número de mosquitos? ¿Por qué?

Moderadora

¿Cuán posible es realizar esta actividad en su comunidad para reducir el número de mosquitos?

Azul

Para mi es difícil, ahí sí que es difícil.

Moderadora

¿Por qué?

Azul

Porque tú le puedes decir a una persona, ‘mira te voy a dar este líquido para que tú le eches a todos los envases que tú tienes por ahí y los veas’, y no lo hacen, no lo van a hacer.

Violeta

No lo hacen.

Moderadora

¿No lo hacen?

Azul

No lo hacen, y, es más, a la hora que se le acabe… Es más, hay mucha gente que se lo das, lo cogen y lo venden. [risas] ‘Mira, te vendo esto…’

Violeta

Es la parte donde se olvida.

Azul

Yo creo que…

Moderadora

Se le puede olvidar a la gente hacerlo.

Azul

Ahí no habría…para mí yo no veo un buen control en el uso de ese producto. De ese método. Por lo menos, yo pienso.

Pregunta 5- ¿Apoya usted esta actividad en su comunidad? ¿Por qué? (*Preguntar a cada participante del grupo de discusión*)

Moderadora

Pero, y si se hiciera, ¿apoyaría usted este método en su comunidad?

Verde

¿Cuál método?

Moderadora

Éste. De echar larvicidas. ¿Lo apoyaría? Si se decidiera hacer, ¿lo apoyaría?

Verde

Ay, no sé.

Moderadora

¿No sabe?

Verde

Eso depende también de las personas, si las personas están dispuestas a comprarlo el...

Naranja

Ah, la larva…

Verde

El… el larvicida.

Azul

Vamos a partir de la premisa de que nos lo van a regalar. [risas]

Verde

¿Nos lo van a regalar?

Rojo

Si es para la salud…

Verde

¿Quiénes lo van a regalar?

Naranja

Si me lo regalan más todavía.

Violeta

Más fácil.

Verde

Si me lo regalan… la usan.

Moderadora

¿Apoyaría usted este método en su comunidad?

Naranja

Sí.

Moderadora

¿Lo apoyaría usted?

Violeta

Lo apoyaría.

Moderadora

¿Lo apoyaría usted?

Azul

Yo lo apoyaría…

Naranja

Eso me imagino que no es tan caro… eso viene en un paquetito así…

Azul

…en las áreas públicas, en áreas que no sean… pero en los hogares, no.

Verde

Dentro…No, en los hogares, no.

Azul

En los hogares, en los patios. En especialmente de los parceleros, nosotros, yo entiendo que, educar a la gente para que no tengan envases. Para dar un ejemplo, yo fui a donde un señor aquí cuando María que se le cayó un árbol, y cuando yo me metí a ese patio, ese señor tenía como siete, ocho pailas por todo el patio, y todas con agua. Yo estuve un rato vaciándole esas pailas. Las pailas no las bote porque son de él. Voy a ahora y deben estar llenas de agua.

Moderadora

¿Y usted, lo apoyaría?

Gris

Sí.

Moderadora

¿Y usted, lo apoyaría?

Rojo

También.

Moderadora

¿Y usted, lo apoyaría?

Blanco

[Anotadora tiene en sus notas que el participante #7 (Blanco) apoya la actividad.]

Pregunta 5a- ¿Piensa que su comunidad apoyaría esta actividad? Sí, No, ¿Por qué?

Moderadora

¿Cree usted que su comunidad apoyaría esta actividad?

Verde

Ahí es donde está el punto. Algunos quizás lo aprueben y algunos no.

Naranja

Yo creo que sí, eso quizás no… es para el beneficio de todos ellos y eso no debe ser tan caro. Pero si se lo regalaran, creo que entonces cooperarían más. Que les digan, ‘Pues te la vamos a traer para que lo prueben’.

Verde

Algunos lo harían y otros no.

Moderadora

Te pueden dar la pruebita, pero ¿y después?

Naranja

Sí, pues… Vamos a supones como a veces tú das… unos *samples*. Tú vas a los sitios a veces, ‘una muestrita de esto para ver si te gusta’, entonces pues, tú le das una muestra, ‘mira, esto es bueno, te vamos a dar una muestra, pero la próxima la compra tú’.

Azul

No…

Naranja

Bueno, pero es que no todo el tiempo se le va a dar todo.

Azul

Yo no te voy a preguntar si ustedes nos van a cubrir eso, no te lo voy a preguntar [risas]. Pero, la gente compra *spray* este de matar mosquitos porque el mosquito lo pica, mientras el mosquito no lo pique, no lo compran. Yo tampoco. Si el mosquito el mosquito no está, no van a comprar para matar una larva de un mosquito que no lo ha picado.

Verde

Por eso te digo…

Azul

La gente no lo va a ver… esa gente no lo va a… yo entiendo, yo lo que pienso es que la gente no va… eso no va a funcionar. Para mí, no funciona.

Moderadora

¿Y ustedes piensan que su comunidad apoyaría este método?

Gris

[No se escucha en la grabación.]

Blanco

Yo le voy a hacer franco. Yo, de acuerdo con…

Rojo

Bueno, nosotros lo hacemos.

Blanco

No…

Rojo

Lo hacemos…

Moderadora

Ustedes lo harían. Usted cree que su comunidad lo apoyaría. Este método de larvicida.

Blanco

Le voy a decir la franca verdad. A la experiencia que yo tengo ya en la comunidad donde yo vivo hace 22 años, la gente es apática para todas estas cosas. Nosotros, y ahí está [nombre de participante cuatro (*Azul*), de testigo, y está _______ [nombre de otra persona, no participante] aquí. Nosotros siempre estamos en la comunidad, buscando, ayudando a la gente. Y echando para adelante, todo lo que sea…

Moderadora

También se me van los tres…

***Participantes uno (*Verde*), dos (*Naranja*) y tres (*Violeta*) se retiran del grupo de discusión por otros compromisos***

Moderadora

Bueno, pero seguimos entonces. Seguimos. Este…

Blanco

Yo estaba hablando.

Moderadora

Ajá. Usted estaba hablando.

Blanco

Perdón.

Moderadora

Sí, dígame.

Blanco

Entonces, ya yo tengo la experiencia con mi comunidad. Mi comunidad es unas personas que… yo cuando hacía actividades, iba casa por casa… hay 175 unidad de vivienda, yo me levantaba a las 5:30 de la mañana para ir… a las personas que se iban a trabajar, darles una invitación, una invitación. Y yo calculé que hay 300 personas ahí. Incluyendo jóvenes y todas las personas. Ok. Cuando hacíamos la actividad, íbamos diez personas. O sea, que a las personas allí, no les interesa nada. Le voy a decir francamente. Nosotros no, porque nosotros siempre hemos estados en actividades y apoyando todo lo que sea bueno para la comunidad. Pero, ese es el problema que tenemos. La gente es apática para... ahora, en la Navidad, yo hacía una actividad, comida, fiesta y todo y se me llenaba…

Moderadora

La casa.

Blanco

El cubo de agua. El centro comunal se llenaba y se tenía que buscar sillas para afuera para la gente. Y eso es lo que nos pasa. Yo la… yo estoy de acuerdo como [apellido del participante cuatro (*Azul*)], ir a todas estas cosas, pero ese es el problema que enfrentamos con los vecinos.

Pregunta 6- ¿Qué otra información necesitaría para entender mejor esta actividad?

[No se hizo la pregunta]

Pregunta 7- ¿Considerarían realizar ustedes mismos esta actividad?

Moderadora

Y si… usted, tuviera la oportunidad de utilizar este método en su casa, ¿usted lo haría?

Blanco

Seguramente que sí.

Moderadora

¿Y usted lo haría?

Rojo

[*Anotadora presencial tiene en sus notas que todos los participantes considerarían realizar la actividad.]

Moderadora

¿Y usted?

Gris

[*Anotadora presencial tiene en sus notas que todos los participantes considerarían realizar la actividad.]

Moderadora

¿Y usted?

Azul

Sí, seguro que sí.

Rojo

Yo lo hago.

Pregunta 8- ¿Qué les haría difícil realizar esta actividad?

Moderadora

¿Qué le haría difícil realizar este método?

Blanco

A mi ninguna porque...

Azul

Obtener la disponibilidad del líquido, del producto.

Rojo

Después que sea para la salud nosotros lo compramos listos para que no los piquen.

Azul

Sí, que haya pro… que el producto se consiga y que no sea muy caro.

Pregunta 9- ¿Hay algo que podría ayudarles a realizar esta actividad de manera más fácil?

Moderadora

Ok. ¿Hay algo que podría ayudarles a realizar este método de manera más fácil?

Azul

No sé, no creo.

Pregunta 9a- ¿Necesitarían más información?

Moderadora

¿Qué información necesitaría?

Azul

La forma de diluirlo, la cantidad que se va a usar.

Moderadora

Ah, muy bien.

Blanco

El uso que se le va a dar.

Azul

Como la preparación.

Pregunta 9b- ¿Necesitarían más adiestramiento?

Moderadora

Ok. ¿Y necesitarían adiestramiento para eso?

Azul

No creo.

Gris

No, eso viene en la etiqueta.

Azul

Si vienen instrucciones claras.

Blanco

Leer bien la etiqueta.

Actividad #3 – Rociar larvicida desde un camión

Moderadora

Ok. Bueno, pues vamos al próximo que nos quedan bastante. Rociar larvicida desde un camión. Rociar larvicida desde un camión.

Azul

Ah no, eso no es.

Moderadora

Se acuerdan de que, yo les había dicho que venían granulados, que venía en tabletas y que venía en líquido. Pues, esta es la preparación líquida. Los larvicidas se pueden aplicar de diferentes maneras. Pero muchos programas de control de mosquitos han encontrado, que aplicar el larvicida desde un camión puede ser efectivo para alcanzar la mayoría de los lugares donde se encuentran las larvas. El larvicida se rocía desde un camión sobre edificios, la vegetación, y las propiedades, y terrenos, y etc. Los larvicidas tienen que re-aplicarse regularmente. ¿Entendieron esa explicación? Ahí está el camión, verdad. Rociando el larvicida líquido por las calles.

Pregunta 2- ¿Es esta una actividad nueva para usted o es algo que ya había escuchado antes? Si la escuchó, ¿dónde la escuchó?

Moderadora

Esta actividad, ¿es algo nuevo para ustedes?

Blanco

No.

Azul

Eso sí, para mí…

Rojo

No. Eso es…

Azul

Eso es larvicida, eso no es para matar mosquitos. Eso es para matar la larva también.

Rojo

Ah.

Moderadora

Es para matar la larva.

Azul

Es el mismo méto… el mismo producto, pero tirándolo por el aire. Porque es larvicida. Ese yo no e veo…

Moderadora

Es nuevo para ustedes.

Pregunta 3- ¿Cree que esta actividad reduciría el número de mosquitos en su comunidad? ¿Por qué?

Moderadora

Y entonces, ¿cree que esta actividad reduciría el número de mosquitos en su comunidad?

Blanco

Si eso se riega en los sitios específicos, sí.

Azul

Si llega. Si llega al sitio…

Blanco

Si llega al sitio.

Azul

Yo creo que todo lo que se haga para eliminar, siempre va a eliminar algo, siempre. Porque, en algún momento, eso si lo hace, va a llegar a algún sitio donde haya agua acumulada. Pero no creo que vaya a llegar a todos lados. La ventaja es que si es un camión que lo va a hacer, a lo mejor lo costea el gobierno o la agencia que los designa. Pero…

Moderadora

O sea, que eso sería una ventaja.

Azul

Es la ventaja que yo le veo. Pero, la efectividad no la veo porque, por ejemplo, estas casas que son cerradas con terraza atrás, que tienen envases con bromelias y matas que acumulan agua, no les va a llegar a ellas. ¿Y si no les llega? Y podría llegarle, pero hay sitios donde los camiones no llegan que tienen que quedarse lejos.

Moderadora

O sea, que eso sería como una desventaja. Usted piensa que no va a llegar a todos los sitios. Aunque sea hecho desde un camión.

Azul

Yo lo veo menos eficiente que directamente tirarlo.

Pregunta 3a- ¿Qué beneficios o ventajas tiene esta actividad para usted?

Moderadora

Ok. Y usted, ¿qué piensa? ¿Cuáles serían los beneficios o ventajas que tiene este método?

Blanco

Bueno, los beneficios que puede tener son, como te lo dije anteriormente, si esa acción se hace donde esté el problema. Por ejemplo, donde yo vivo. Nosotros tenemos lo que era el río portugués, eso es un caño ahí. Esa agua está estancada. Ahí, ese sitio, si ese camión funciona ahí, pues está muy bien. Y yo creo que es beneficioso si se tira ahí.

Pregunta 3b- ¿Qué desventajas o dificultades le ve a esta actividad? ¿De qué forma se podrían solucionar esas dificultades?

Moderadora

Ok. Y entonces, ¿qué desventaja usted le ve? ¿Le ve alguna desventaja?

Rojo

Una pregunta. Cuando eso empiezan a salir, para que los mosquitos… este… los herbicidas…

Blanco

La larva.

Rojo

…pueda, ajá. Entonces, esas personas que huelen ese… ¿le podría hacer daño? A personas que también padecen de asma y todas esas cosas.

Moderadora

Rafa, apúntame esa pregunta. Apúntame esa pregunta. O sea, que eso también, para usted podría ser una desventaja.

Azul

O sea, porque se desconoce si cuando se inhala cause algún tipo de efecto….

Moderadora

Podría causar daño, asma…

Azul

… a las personas. Especialmente al que tenga…

Moderadora

…alguna condición respiratoria.

Azul

…especialmente a las personas que tengan problemas respiratorios. Pero no sé.

Moderadora

Ok. O sea que, entonces, sería importante si yo le pregunto, ¿qué información necesitaría para entender mejor este método? Esa sería una pregunta que usted tendría. Si este método es nocivo para las personas, verdad.

Rojo

Exacto.

Azul

Sí. Si el producto causa algún tipo de…

Moderadora

De cosa de…

Azul

…condición.

Moderadora

…daños respiratorios. Ok.

Pregunta 4- ¿Cuán posible es realizar esta actividad en su comunidad para reducir el número de mosquitos? ¿Por qué?

Moderadora

¿Cuán posible sería realizar esta actividad en su comunidad para reducir el número de mosquitos? Usted que…

Azul

Aquí es fácil. Hacerlo es fácil... y como te dije, yo siempre entiendo que todo lo que se haga va a reducir. Pero de que sea en mayor grado que haciéndolo directamente con el larvicida que tú dijiste, creo que es menos eficiente. Pero, sí, siempre la va… para mí, que… Porque dijo él que cuando fumigan, cuando fumigan, que dice que lo va a mencionar, este, no mata todos los mosquitos.

Moderadora

Ese es. Ese es. Lo que pasa es que este es otro tipo de fumigación.

Azul

Ah porque…

Moderadora

Ya nosotros sabemos que el gen… no incluimos la fumigación porque ya sabemos que la gente, tradicionalmente lo sabe. Algunos lo quieren y otros no.

Azul

Ah bueno. Si no, eso iba a decir ahorita, que mucha gente… creo que habían dicho que, que hay problemas con eso.

Moderadora

Sí. Tradicionalmente lo saben, verdad. La gente sabe porque lo hemos vivido toda la vida, así que por eso no lo incluimos porque ya sabemos las opiniones de la gente.

Azul

Sí.

Moderadora

Estamos quizás hablando de cosas que la gente, pues tiene…

Gris

Conocimiento…

Moderadora

Que… aun no nos ha dado su opinión al respecto. Y por eso es que estamos haciendo esto. Ok. Ajá. Dígame.

Azul

Yo entiendo que es menos eficiente que ir directamente a la fuente, pero, de que va a tener algún tipo de ayuda… porque si eso pasa cerca, al área donde ellos viven que está esa agua estancada y cae de eso, va a resolver el problema allí.

Pregunta 5- ¿Apoya usted esta actividad en su comunidad? ¿Por qué? (*Preguntar a cada participante del grupo de discusión*)

Moderadora

Ok. ¿Y usted, apoyaría esto si se hace en su comunidad?

Blanco

Claro. Seguro.

Azul

Yo creo que ahí se beneficia.

Moderadora

¿Usted lo apoyaría?

Rojo

[*Anotadora presencial tiene en sus notas que participante #6 (Rojo) apoyaría la actividad.]

Moderadora

¿Usted lo apoyaría?

Gris

Claro.

Moderadora

¿Y usted, lo apoyaría?

Azul

Apoyarí… yo lo apoyaría, pero, ok, vuelvo y te digo, porque todo lo que se haga va a reducir en algún grado. Ahora, si me preguntas si es efectivo o no, yo entiendo que no es 100% efectivo.

Moderadora

Ok. ¿Y qué información…?

Azul

Perdona, no es 100% efectivo porque eso a lo que va a matar es la larva. Si fuera a matar al mosquito es otra cosa. Pero entonces si… eso tiene que llegar a la larva, y si no llega a los estanques donde está la larva, no las va a matar. Y si fumigan ahora y el efecto de eso dura un mes, y vienen a los dos meses, no se pierde mucho.

Moderadora

Ah ok. Que también tiene que ser consistente…

Azul

Consistente con el periodo que mantiene la efectividad el producto.

Gris

Y que también, el líquido debe ser puro para que entonces pueda matar la larva. Porque a veces, pues, echan esa cosa, y ni huele. [risas]

Azul

Lo que pasa es que estábamos acostumbrados cuando lo echaban, si era bien fuerte el olor, estaba matando a los mosquitos, ‘ah, eso no huele a nada, eso no… los mosquitos’.

Moderadora

Antes se usaba malatión, verdad… cuando nosotros nos criábamos, que aquí ninguno es jovencito, verdad, yo me incluyo. Cuando nosotros nos criábamos, se usaba el malatión y el malatión cogía a uno y lo acababa.

Azul

Hasta la garganta te chavaba, mano.

Moderadora

Sí. Pues ahora se usan otros productos que son… que no…

Azul

Son efectivos en lo que hacen, pero…

Moderadora

Que son efectivos, pero no causan…

Gris

Daño…

Azul

Como la garganta de uno no se le afecta, ‘mira, eso no está haciendo nada’.

Moderadora

Exacto, usted tiene… [risas]

Azul

Ese café está aguado.

Pregunta 5a- ¿Piensa que su comunidad apoyaría esta actividad? Sí, No, ¿Por qué?

Moderadora

Y entonces. ¿Piensa que su comunidad apoyaría esta actividad?

Azul

Pienso que sí.

Moderadora

¿Sí? ¿La apoyaría?

Azul

Sí. Creo que sí.

Moderadora

¿Por qué?

Azul

Porque se ve la acción. Y la gente… a la vez que la gente vea algo echando- fumigando, ven que se está haciendo algo. Y la gente todo lo que vean…

Blanco

Se interesan.

Azul

Sí, se interesan y, pues, se alegran, y lo apoyan, seguro que sí.

Pregunta 6- ¿Qué otra información necesitaría para entender mejor esta actividad?

Moderadora

Ok. ¿Y qué otra información necesitaría las personas para entender mejor este método? Ya usted dijo del olor, si causa algún tipo de condición respiratoria, algún tipo de daño. ¿Qué otra información necesitaría saber las personas?

Azul

No sé. Yo pienso el tiempo de efectividad del producto.

Moderadora

El tiempo de efectividad del producto. ¿Qué más? ¿Hay alguna otra cosa?

Gris

Cuanto tiempo lo harían.

Moderadora

Ah, por cuánto tiempo lo harían…

Azul

Con qué frecuencia.

Gris

Sí, con qué frecuencia. Si es un mes, dos meses o cada... el tiempo.

Actividad #4 – Fumigación dentro de las casas con insecticida de acción residual

Moderadora

Ok. Bueno, pues vamos para la próxima que también tiene que ver con fumigación. Dice, fumigación dentro de las casas con insecticida de acción residual. La fumigación dentro de las casas con insecticida de acción residual es un método de control de mosquitos donde un profesional adiestrado trata el interior de su hogar con un insecticida. Consiste en rociar las paredes y otras superficies de la casa con un insecticida que continúa funcionando varios meses. Mata los mosquitos que se posen en superficies que han sido rociadas con el insecticida. Este tipo de fumigación se ha usado en muchos países del mundo incluyendo Puerto Rico y los Estados Unidos. Puede ser efectivo en reducir la cantidad de mosquitos si se aplica a una gran cantidad de casas en un área. Después de aplicarlo, puede que haya olor por unas horas, pero es poco probable que cause daño a las personas cuando se hace correctamente. Requiere del permiso y de la disponibilidad del residente para entrar a la casa a fumigar. El uso repetido, a través del tiempo puede hacer que los mosquitos sean resistentes a los insecticidas. Esta fumigación debe repetirse para mantener bajas las poblaciones de mosquitos.

Pregunta 2- ¿Es esta una actividad nueva para usted o es algo que ya había escuchado antes? Si la escuchó, ¿dónde la escuchó?

Moderadora

Les pregunto. ¿Esta actividad es algo nuevo para ustedes?

Azul

Para mí no es nueva, porque cuando éramos pequeño eso lo hacía el gobierno.

Moderadora

¿Iban a su casa y les fumigaban las paredes y todo dentro de la casa?

Azul

Yo me acuerdo, cogían un papelito lo llenaban y lo pegaban en la pared de la sala, de la cocina así.

Moderadora

¿Ah sí?

Azul

Eso estaba ahí todo el mes. Cuando fumigaban las casas.

Moderadora

¿Ah sí?

Azul

El sistema era que te fumigaban la casa y el patio y entonces, orientaban a las personas. Decían, ‘usted no puede tener estos envases…’. Ese sistema se usó mucho. Eso es lo que llamaban la malaria que había aquí…

Moderadora

Ah, claro.

Azul

Sí. Pues ese era el sistema que usaban ellos.

Moderadora

Es que yo soy un poquito más de Menudo para acá. [risas]

Azul

Lo que pasa es que, yo no sé de dónde tú eres, pero a lo mejor en donde tú vivías no era tan necesario hacerlo.

Moderadora

Carolina. Imagínese. Esa es otra zona de mosquitos terrible.

Azul

Pero aquí en Ponce, aquí eso… en la Playa siempre.

Moderadora

Sí. Y cuando había malaria.

Azul

Había ese problema antes, porque había mucho mangle. Aquí siempre se fumigó, cuando yo era pequeño, me acuerdo.

Rojo

En Villa del Moderadora, también.

Moderadora

Así que lo hacían. Lo hacían.

Azul

Sí. Por lo menos yo lo conozco, el sistema.

Pregunta 3- ¿Cree que esta actividad reduciría el número de mosquitos en su comunidad? ¿Por qué?

Moderadora

¿Cree que esta actividad reduciría el número de mosquitos en su comunidad? ¿Y por qué?

Rojo

Perdón. ¿Cómo es?

Moderadora

¿Cree que este método reduciría el número de mosquitos en su comunidad?

Azul

Yo creo que sí.

Moderadora

¿Cree que sí?

Azul

Yo creo que todo lo que se haga para eliminar los elimina. Ahora, yo no quie… no me gusta ese sistema.

Moderadora

OK. Ahora vamos a intrigar.

Azul

Esa es la otra pregunta…

Moderadora

Vamos a llegar ahí. ¿Ustedes también creen que esta actividad, este método reduciría el número de mosquitos en su comunidad?

Rojo

Sí. Yo creo que sí.

Moderadora

¿Sí? Ok.

Pregunta 3a- ¿Qué beneficios o ventajas tiene esta actividad para usted?

Moderadora

¿Por qué? ¿Cuáles son los beneficios y las ventajas de esta actividad?

Azul

Ah bueno, verdad, sí, paso por paso.

Moderadora

Paso por paso. Paso por paso. [risas]

Gris

Beneficio, porque mata a todos los animales; y si es constantemente.

Moderadora

Ok. O sea que se haga… si se hace constantemente como indica, verdad, pues sí tiene un beneficio.

Gris

Tiene beneficio…

Moderadora

¿Cuál sería otro beneficio?

Azul

Pues, el beneficio que yo le veo es que mata al mosquito que se mete a tu casa. Al que se mete a tu casa, los va a matar. Si aquel no quiere que le fumiguen, o no le fumiguen… si tú lo tienes en tu casa… porque te garantiza que te dura un tiempito el efecto. Es una ventaja que yo le veo.

Moderadora

¿Qué otro beneficio le ven?

Blanco

Yo estoy de acuerdo con él. Eso es un beneficio lo que está diciendo. Yo estoy de acuerdo, sí, también. Yo le veo esos beneficios.

Pregunta 3b- ¿Qué desventajas o dificultades le ve a esta actividad? ¿De qué forma se podrían solucionar esas dificultades?

Moderadora

Y entonces, ¿qué desventajas o dificultades le ve a esta actividad?

Blanco

Yo no le veo ninguna.

Rojo

Ninguna.

Blanco

Desventaja, ninguna.

Moderadora

¿No le ven?

Azul

Yo le veo desventaja y no lo apoyo.

Moderadora

¿Cuál es la desventaja? Eso… esa es la que quiero. La que quiero. ¿Cuál es la desventaja que usted le ve?

Azul

Espérate, te voy a decir una cosa. “Después de aplicarlo puede que haya olor por unas horas”; eso es una desventaja. Si tú vas a mi casa y haces eso mi esposa se muera en esas dos horas, se va a morir. En mi casa no puedo fumigar nada, nada con olor y nada que ella esté. Porque ella sufre de alergias… unas alergias que es horrible. Y eso es, en la semana, tres y cuatro veces en la semana, siempre está así. Hoy estaba así. Y no puedo fumigar en nada. Yo cuando en casa… yo fumigo mucho en casa porque en el sitio que yo vivo hay mucho ciempiés. Y yo fumigo porque… de hecho, los otros días me picó uno chiquitito así. Es que aparecen, aparecen. Y yo fumigo cuando ella no está por las orillas. Y cuando aparecen, aparecen bobos y los puedo matar. Y eso pues, es una desventaja para las personas. Y si hay nenes pequeños pues me imagino que… Y el otro que para mí definitivamente no voy a permitir, es que van a permitir que entren personas a tu casa; que tú no sabes quién es, esa persona puede ser empleado del gobierno, pero como están las cosas ahora…aparecen un día, ‘no, yo vengo a fumigar’. Vienen a fumigarte la casa, a fumigarte las gavetas y todo lo que ven. Yo, permitir personas a que entren así a una casa, yo no lo permitiría en mi casa. A parte de que tú en tu casa tienes cosas en el cuarto, él no va a fumigar por todos lados, porque no va a mover todas las cosas y va a empezar a tirar por todos lados. Ese sistema yo lo veo un poco… por lo menos a mí no me gusta.

Rojo

La pregunta mía es, que, si se hace eso dentro de la casa y hay niños pequeños que, los niños se pegan en todo.

Moderadora

Tocan todo.

Rojo

Ajá. Tocan todo, no se lavan las manitos, se meten las manitos en la boca y todas esas cosas… ¿haría daño?

Moderadora

O sea, que eso sería una información que también debería tener. Qué va a pasar con los niños que tocan todo, verdad, si eso hace daño o no hace daño. Rafa, apúntame.

Azul

Por eso, porque tú dices que es poco probable que cause daño a las personas cuando se hace correctamente; pero correctamente, pues echando en la pared, pero como dice ella, si alguien toca… yo supongo que no hace daño, porque si lo permiten daño no le va a hacer, pero si es nene enfermo, que tenga las defensas bajas.

Rojo

Los niños también se ponen en el piso a jugar y todas esas cosas.

Azul

Personas mayores con defensas bajas. Habría que ver.

Moderadora

¿Y usted le ve alguna desventaja?

Gris

¿Desventaja?

Moderadora

Ajá.

Gris

Pues, las que dice ella.

Moderadora

Ok. Y entonces, ¿de qué forma podrían solucionarse esas desventajas? Uno sería dándoles información, verdad, sobre ese particular.

Gris

Orientando.

Moderadora

Orientando, exacto. Y, ¿cómo podríamos trabajar el asunto de la entrada a la casa?

Azul

Eso lo veo un poco difícil.

Blanco

Esa es la difícil.

Azul

Porque cuando estamos hablando de este sistema, ¿es un sistema que lo establecería el gobierno? O sea, ¿que el que va a hacer eso es un profesional que el gobierno lo contrate, o somos nosotros?

Moderadora

Es un profesional, no lo puede hacer la persona.

Azul

Por eso, pero lo…

Moderadora

Es un profesional que tiene… adiestrado y licenciado.

Azul

Adiestrado. Pero no contratado por nosotros.

Moderadora

No.

Azul

Por eso, que nosotros no lo vamos a conocer, a la persona.

Moderadora

Ok. O sea, que si fuera unas personas, para usted…

Azul

Si es una compañía que yo conozco pues yo llamo a tal compañía y vienen y me fumigan ya yo sé que la conozco. O conozco a una persona que sabe fumigar, yo la pongo a que fumigue que es certificada, pero va a venir una persona que no sabe quién es.

Moderadora

O sea, que para usted sería, que no lo tiene que hacer necesariamente el gobierno, sino que podría ser una persona que usted confíe, que sea un servicio que pueda dar privado.

Azul

Una persona de confianza que sea un profesional.

Moderadora

Ok. Ok. Muy bien. No, eso está muy bien.

Pregunta 4- ¿Cuán posible es realizar esta actividad en su comunidad para reducir el número de mosquitos? ¿Por qué?

Moderadora

¿Cuán posible sería realizar esta actividad en su comunidad para reducir el número de mosquitos? ¿Usted cree que es posible hacerlo? Que vayan casa por casa fumigando las paredes.

Azul

Por lo menos en mi sector. Y recuerde que yo nací, y criado en las parcelas… yo no vivo aquí, pero toda mi vida he estado aquí, aquí sería más fácil que en otros sitios, que en los otros sectores.

Moderadora

¿Por qué?

Azul

Porque la gente en esta comunidad son personas bien abiertas, que tú les dices, ‘mira…’, y te permiten entrar, entran te fumigan… ya cuando son urbanizaciones la gente es un poquito más reservada.

Moderadora

Ah, eso es importante.

Azul

Y ese es el asunto de la Playa, que tiene diferentes sectores, y hay urbanizaciones, hay lo que son las parcelas.

Moderadora

¿Y para usted, que vive en urbanización?

Blanco

Difícil.

Moderadora

¿Difícil que dejen entrar a las casas? ¿Aunque sea el gobierno, aunque sea una agencia privada?

Blanco

Sea quien sea, es más difícil.

Azul

Es más difícil. Una urbanización…

Moderadora

¿Por qué?

Blanco

Porque la gente no le gusta cooperar en ese sentido.

Gris

La desconfianza.

Azul

Tienden a ser más reservados. Ya cuando están en urbanización es un poquito más de nivel.

Moderadora

¿Usted es de urbanización?

Gris

Sí. Vivo en SA.

Moderadora

Y, entonces, dan desconfianza, de la gente que vive en urbanización. Ok. Y, ¿qué hay alguna forma de solucionar ese problema?

Azul

Con orientación posiblemente, pero hay que entrar en esa etapa.

Blanco

Orientación.

Moderadora

Por ejemplo, si se…

Gris

La única, perdón. De la única manera, y no creo que se podría, es ir con la persona [líder de comunidad], y decirle, este, ‘mire, esta persona viene para fumigar su casa, ¿usted lo permite? Esto y lo otro’.

Moderadora

Cosa que el líder de comunidad vaya con la persona…

Gris

Exacto.

Moderadora

Ah ok.

Gris

Esa sería un…

Moderadora

Y si la persona tuviera un uniforme y una…

Blanco

Identificación.

Azul

No sé, quizás si se establece el sistema por el gobierno, la gente sabe que van a venir. A lo mejor la gente poco a poco se abre y lo va permitiendo.

Gris

Irlo anunciándolo yo creo que por la televisión por la radio. Que la gente ya esté preparada para cuando llegue esa persona, pues ya uno sabe, ‘ah, ustedes son…’.

Azul

Que yo me acuerdo, todavía en la mente yo tengo claro cuando yo era pequeño, que esas personas entraban a la casa y mis papas los saludaban, y empezaban a fumigar sin preguntar y se iban. Llenaban el papel y pégalo. Le metían harina de trigo y [no se entiende la palabra]…

Moderadora

Harina de trigo. [risas]

Azul

No sé lo que usaban, pero era blanco y lo pegaban.

Pregunta 5- ¿Apoya usted esta actividad en su comunidad? ¿Por qué? (*Preguntar a cada participante del grupo de discusión*)

Moderadora

Ok. Piensa… ¿usted apoyaría esta actividad en su comunidad si se diera?

Blanco

Seguro que sí.

Moderadora

¿Usted?

Rojo

Claro.

Moderadora

¿Y usted?

Gris

Sí.

Moderadora

¿Y usted?

Azul

Yo, aunque estoy en contra de eso, si la gente lo acepta, todo lo que sea en beneficio para eliminar mosquitos yo estoy de acuerdo.

Pregunta 5a- ¿Piensa que su comunidad apoyaría esta actividad? Sí, No, ¿Por qué?

Moderadora

¿Y piensa usted que en su comunidad apoyaría esta actividad?

Blanco

Ahí, yo…Yo le voy a decir una cosa, ahí estoy en duda en cuanto a eso a la gente de la comunidad mía. Por lo que estábamos diciendo, la gente de urbanización, siempre usted sabe que viven encerrados. Y no son muy conforme con que la gente vaya a las casas.

Pregunta 6- ¿Qué otra información necesitaría para entender mejor esta actividad?

Moderadora

Ok. Bueno. Ya me dijeron que la información que necesitarían. Si le hace daño a los niños si…

Gris

Anunciarlo antes de…

Moderadora

….el olor, que había que anunciarlo y orientar a las personas. Ok.

Actividad #5 – Trampa AGO para mosquitos

Moderadora

Vamos para la próxima. Trampas AGO para mosquitos. Las trampas para mosquitos AGO, ya han sido utilizadas en Puerto Rico para reducir el número de mosquitos. La trampa atrae y captura los mosquitos hembra *Aedes aegypti* que buscan envases para poner sus huevos. La trampa consiste en una paila negra de cinco galones. Que es esta. Una cámara de captura, que es esta, y un escrín. La paila está llena hasta la mitad con agua y heno ¿Ven el heno? Para atraer los mosquitos. Dentro de la cámara de captura, que es esto, hay un papel, que es este, con pega especial que atrapa a los mosquitos cuando entran a poner sus huevos. El mosquito entra por aquí y se queda ahí pegado. Y como esto tiene un escrín, pues no puede bajar al agua, verdad. Y entonces, la trampa contiene material orgánico, que es este, así que puede oler. No requiere, no se requiere de entrenamiento especializado para armar la trampa. La trampa requiere mantenimiento cada dos meses, para que no se convierta en criadero de mosquitos. Y la trampa reduce la cantidad de mosquitos si se mantiene adecuadamente y se usa en ocho de cada diez hogares en su comunidad. Y lo que no dice ahí, también lo incluyo, hay que tener tres trampas por casa. Dos atrás, una al frente. Para que cubra bien.

Azul

Sabes qué…

Moderadora

Entonces, les pregunto… vamos por parte. [risas]

Azul

No. No. Está bien te digo ahorita.

Moderadora

Ok.

Azul

Con relación a ese sistema.

Pregunta 2- ¿Es esta una actividad nueva para usted o es algo que ya había escuchado antes? Si la escuchó, ¿dónde la escuchó?

Moderadora

¿Esta actividad es nueva para ustedes ya la habían escuchado antes?

Blanco

Es nueva.

Moderadora

Nueva. ¿Nueva?

Rojo

[No se escucha en la grabación, pero parece haber afirmado con algún gesto.]

Moderadora

¿Nueva?

Gris

[No se escucha en la grabación, pero parece haber afirmado con algún gesto.]

Moderadora

Para usted no es nueva.

Azul

¿Le puedo decir por qué?

Moderadora

¿Por qué?

Azul

Porque la Escuela de Medicina, implantó en la Playa, de esas trampas, en muchos hogares ahí. Ahora mismo, en las parcelas hay como…un montón de casas que tienen esas trampas. Esas mismas trampas. Ahora…

Moderadora

Ellos lo usan como un sistema de vigilancia, no como un sistema de control. Esa trampa…. Dilo.

Rafael

No, pero él está hablando de la parte de la Escuela.

Moderadora

Ah.

Azul

No, ellos… sí, perdona, lo que pasa, que ellos trajeron un sistema del que nos hablaron, tres… uno para coger estos mosquitos para estudiarlos. Hablaron de la trampa para cogerlos. Y de hecho, se supone que vienen a cambiar, esos huevitos que ellos cojan ahí, que se muera la hembra que los puso, los van a usar para estudiar. Pero eso los recoge porque eso es exactamente lo que ellos pusieron. Ahora, ellos pusieron uno por hogar, y están regados. No es uno aquí, uno al lado, eso es…

Sue

Porque es vigilancia….

Moderadora

O sea, ¿Qué son ustedes?

Sue

Sí, es ellos…

Rafael

Yo creo que sí.

Moderadora

Sí, ¿verdad? Sí, son ellos.

Azul

Yo me imagino, pues, yo me imagino que aparte de que lo están haciendo para hacer un estudio. Si se implanta para el propósito aquello, pues es perfecto.

Moderadora

Ok. Le explico. A lo mejor Rafa aquí me puede ayudar. Esta paila se usa de dos formas. Como usted dijo. Antes de hacer un estudio aquí, nosotros tenemos que tener un conteo, verdad, de cuántos son los mosquitos más o menos, un promedio de los mosquitos que hay en la comunidad. ¿Por qué tenemos que saber eso? Porque si luego se implanta alguna de estas actividades en su comunidad, nosotros queremos saber, cómo esa actividad bajó el número de mosquitos, verdad. Así que tenemos que coger primero, se tienen que coger primero, más o menos un aproximado…. Nosotros lo sabemos por el más o menos la aproximación de mosquitos que hay. Por eso, es que se despegan los mosquitos, verdad.

Azul

¿Tú eres CDC?

Moderadora

Se despegan y se cuentan.

Rafael

Yo soy de la Unidad de Control de Mosquitos.

Azul

Ah, por eso, sí, que están más [no entiendo la palabra] que la Escuela de Medicina.

Moderadora

Exactamente.

Azul

Pero, te pregunto, porque la Escuela de Medicina primero implantó el sistema de recolectar. Era con las gomas. Que ellos se llevaban los huevitos y qué sé yo, y venían y cambiaban y filtraban el agua, después de eso, inventaron esto.

Moderadora

Pero ese, es otro proyecto.

Azul

Por eso, sí.

Moderadora

Era de la Escuela de Medicina igual, pero era con el Dr. Orengo.

Azul

Exacto.

Moderadora

Este es otro proyecto. Y entonces, esa misma trampa se usa así por eso se puede usar regada, no tiene que haber una en cada casa. Pero cuando se usa para controlar el número de mosquitos que están en sus casas, entonces hay que tener las tres trampas en cada casa.

Azul

Pues por eso. Yo te voy a decir y te lo digo a ti ahora, yo he venido a las reuniones y cuando nos presentaron eso, yo una de las cosas que iba a plantear, y me quedé callado, porque dije, ‘vamos a dejarle, darle tiempo al tiempo’, es que yo entiendo que está bien que hagan estudios para saber la cantidad de mosquitos, saber las enfermedades, pero yo quiero ver acción para eliminar que es lo que estoy viendo ahora. Que es lo que me interesa.

Moderadora

Exacto.

Azul

Por eso yo di tiempo al tiempo, pero si tú me decías eso, cuando yo vi que él habló de eso, primero dijeron, ‘no para eliminar’, después él habló de llevárselo y sacar conteo y qué sé yo, pues yo… lo tenía así, pero pensé que era como que una trampa, ese.

Pregunta 3- ¿Cree que esta actividad reduciría el número de mosquitos en su comunidad? ¿Por qué?

Moderadora

O sea que entonces, volviendo a la, verdad, luego de la explicación que le di. ¿Cree que esta actividad reduciría el número de mosquitos en su hogar? Usted dice que eso sería fantástico.

Azul

Ese es fabuloso.

Moderadora

¿Y los demás?

Blanco

Sí, sí.

Azul

¿Tú sabes por qué? Porque eso tú lo pones, y la gente puede tener los envases, y ese mosquito cuando salga va a buscar y si ese método… si ese, tiene un sistema donde atrae más al mosquito, que es el sistema del heno, y el sistema de que es negro, va a haber más mosquitos metiéndose ahí que en una mata que tenga agua.

Moderadora

Exactamente.

Gris

Una pregunta. Ese sistema, ¿es que ustedes lo llevan a la comunidad o uno tiene que hacerlo?

Moderadora

Otra pregunta para anotar para darle información.

Azul

Es que eso… bueno, no sé si uno tenga que comprarlo, pero eso…

Moderadora

Ahorita hablamos de eso. Pero eso sería una de las informaciones que la persona debería tener. ¿Qué información? Si es algo que el munici… el municipio no, el gobierno va a dar o si es algo que el residente tiene que obtener. ¿Verdad?

Azul

Yo creo que sí, eso hacen una propuesta y se… nos lo dan.

Moderadora

Ok. Y entonces, ya mismo… esa, la contestamos al final.

Pregunta 3a- ¿Qué beneficios o ventajas tiene esta actividad para usted?

Moderadora

¿Qué beneficios o ventajas tiene esta actividad para ustedes? Beneficios.

Azul

Que no cuesta. Digo, si no tenemos que comprar el balde. [risas]

Moderadora

Si no cuesta, verdad.

Azul

Exacto. Si no hay que comprarlo pues ya es económica. Es efectiva...

Moderadora

Ok. Y para ustedes. ¿Qué ventajas y beneficios tiene?

Rojo

La educación que nos estás dando hasta ahora.

Moderadora

¿Ah?

Rojo

La buena educación que está dando hasta ahora.

Moderadora

No, pero el método.

Azul

El método de usar la paila.

Moderadora

El método de la paila. ¿Qué beneficios o ventajas tiene?

Gris

Pues que elimina los mosquitos.

Rojo

Elimina los mosquitos.

Gris

Va eliminando más mosquitos. Y entonces habría menos enfermedades.

Moderadora

Exacto. ¿Qué más? Específicamente este método.

Blanco

Es una ventaja porque salen de un sitio en específico de una casa. Y esa casa, pues, por lo menos tiene un 99% de librarse de ciertas enfermedades que traen los mosquitos. Porque a la vez que tengamos esas trampas se soluciona un poco el problema de…

Azul

Elimina la preocupación de los químicos que se puedan usar.

Moderadora

Ajá, elimina la preocupación de los químicos.

Blanco

Exacto.

Moderadora

Ok. ¿Y desventajas…? Ajá.

Azul

Puede funcionar sin la preocupación de que la gente tenga envases por ahí. Porque vuelvo y te digo, yo entiendo que el sistema que tiene eso atrae más a los mosquitos, que cualquier otro envase que haya en la casa.

Pregunta 3b- ¿Qué desventajas o dificultades le ve a esta actividad? ¿De qué forma se podrían solucionar esas dificultades?

Moderadora

Ok. Y entonces, ¿Qué desventajas o dificultades le ve a este método? ¿Le ve alguna? Dificultad o desventaja.

Blanco

Bueno, la única desventaja, como dijo [apellido del participante cuatro (Azul)], es obtenerlas.

Moderadora

Ah, que tenga un costo.

Azul

Que tenga un costo.

Blanco

Que tenga un costo…

Azul

No sé, y en hogares… me imagino que habrá hogares que se les haga un poco incómodo tenerlas. Porque esa paila es una paila y ocupa espacio, y si son tres pues imagínate.

Pregunta 4- ¿Cuán posible es realizar esta actividad en su comunidad para reducir el número de mosquitos? ¿Por qué?

Moderadora

Ok, ¿cuán posible sería realizar esta actividad en su comunidad para reducir el número de mosquitos?

Azul

En el caso de nosotros, como somos parcelas, es más viable porque las parcelas tienen mucho terreno.

Moderadora

¿Y en la urbanización? ¿Sería viable hacer esta actividad?

Blanco

No. No.

Moderadora

¿No?

Blanco

Es un poquito difícil.

Moderadora

¿Por qué?

Blanco

Porque para bregar con la gente que vive en urbanización, y convencerlas de estas cosas, es cuesta arriba.

Moderadora

¿Por qué? ¿Por qué?

Blanco

La gen… pues, porque la gente son apática a estar contribuyendo por estas cosas. Usted sabe que, en las urbanizaciones, la persona que vive aquí y la personas que se conocen, pero usted no conoce el que está en la tercera casa o en la cuarta casa. Y ese es el problema que tenemos aquí en las urbanizaciones. Aquí, [apellido del participante cuatro (*Azul*)], conoce a todo el mundo por aquí. Pero ahora mismo, en la urbanización donde yo vivo me conoce mucha gente por las actividades que yo he hecho. Pero, mucha de la gente yo los veo y no los conozco. Hay veces que pasan en los carros y me hacen, ‘Ay, adiós’, y yo, ‘¿y quién será ese?’ [risas] Y entonces, para llevar ese programa a la gente es un poquito difícil.

Azul

Tú sabes que las urbanizaciones, tienen poco terreno al frente y la gente generalmente pone los jardines bien bonitos. Y tu decirle a una persona, ‘mira debes tener una paila ahí en ese jardín…’, muchos lo van a hacer, pero no todo el mundo va a tener una paila al frente. En la parte de atrás, posible. A lo mejor te puedan tener una o dos, pero ya al frente es más…

Moderadora

¿Y la número cinco (*Gris*) que vive en urbanización? ¿Cree que no se podría realizar esta actividad en su comunidad? O, ¿sí?

Gris

Bueno, yo creo que sí.

Moderadora

Cree que sí.

Gris

Yo creo que sí. Pero…

Moderadora

¿Por qué?

Gris

…irlos orientando.

Moderadora

Hay que irlos orientando.

Gris

Ajá. Para que entonces ellos estén preparados.

Moderadora

¿Qué tipo de orientación sería? ¿Qué necesitaría la gente saber?

Gris

Cómo se va a utilizar el sistema, qué cada tiempo tienen que removerlo. O si es que lo van a buscar o…

Moderadora

Ok. Dónde se busca.

Gris

El sitio donde los van a poner. Como dice [nombre del participante cuatro (*Azul*)], si es al frente, son dos atrás o donde se vaya a poner.

Pregunta 5- ¿Apoya usted esta actividad en su comunidad? ¿Por qué? (*Preguntar a cada participante del grupo de discusión*)

Moderadora

Ok. Y entonces, ¿apoyaría usted esta actividad?

Azul

Yo lo apoyaría. Yo creo que lo más importante es que las personas entiendan que es efectivo. Y yo entiendo que sí. Yo entiendo que sí.

Moderadora

¿Y usted?

Gris

[*Anotadora tiene en sus notas que participante #5 (Gris) apoyaría la actividad.]

Rojo

Sí. Yo creo que sí.

Moderadora

¿Y usted? ¿Apoyaría esa actividad?

Blanco

Exacto. Sí.

Pregunta 5a- ¿Piensa que su comunidad apoyaría esta actividad? Sí, No, ¿Por qué?

Moderadora

¿Cree que la comunidad lo apoyaría?

Gris

Sí.

Rojo

Después que se les diga a ellos cómo es que se hace y para qué.

Gris

Que se orienten.

Rojo

Que entiendan el beneficio que ella da.

Moderadora

Ok. ¿Y usted me iba a decir algo?

Blanco

No.

Moderadora

Ok. ¿Y alguien me iba a decir algo? ¿Estamos bien? Ok. Pues vamos para la próxima. Ok. Mosquitos con Wolba…

Sue

Moderadora. Faltaron preguntas de esa.

Moderadora

¿Cuál?

Sue

Las de las 7 a la 9.

Moderadora

Es que estas son… ah, porque esta es una actividad… ah, porque esta actividad, perdón, sí. Esta actividad sería….

Pregunta 6- ¿Qué otra información necesitaría para entender mejor esta actividad?

[Se contestó en otra sección]

Pregunta 7- ¿Considerarían realizar ustedes mismos esta actividad?

Moderadora

¿Consideraría usted, hacer esta actividad, usted mismo en su casa? ¿Usted consideraría que es posible para ustedes poner la trampa, armar la trampa y después cada dos meses darle mantenimiento?

Azul

Yo lo haría.

Blanco

En casa no hay problema.

Azul

Seguro que sí.

Moderadora

Lo haría.

Pregunta 8- ¿Qué les haría difícil realizar esta actividad?

Moderadora

¿Qué les haría difícil a ustedes realizar esta activi… este método?

Blanco

¿En la casa?

Moderadora

En la casa

Blanco

Ninguna. Obtenerla con el sistema de…

Moderadora

Ah, si tiene un precio, cómo obtenerla. Lo mismo que dice él.

Azul

Que me digan que vale 50 pesos… no… y creo que es mejor, vamos a seguir fumigando. [risas]

Pregunta 9- ¿Hay algo que podría ayudarles a realizar esta actividad de manera más fácil?

Moderadora

¿Hay algo que podría ayudarles a ustedes a realizar esta actividad de manera más fácil? Este método, ¿de manera más fácil?

Azul

Pues, que nos ayuden si tuviera algún costo.

Moderadora

Que ayuden, si tuviera algún costo.

Pregunta 9a- ¿Necesitarían más información?

Moderadora

¿Qué información se necesitaría? Ya más o menos ella dijo, verdad. Que tendría la gente que saber.

Pregunta 9b- ¿Necesitarían más adiestramiento?

Moderadora

¿Necesitarían ustedes adiestramiento para armar la trampa y mantener… darle mantenimiento?

Azul

Yo creo que eso es sencillo.

Gris

Bueno, hay que… se supone que se explique a ver cómo es que funciona

Azul

Para todo hay que funciona la primera vez hay que ver si las instrucciones son claras si no pues...

Gris

Exacto.

Actividad #6 – Mosquitos macho y hembra con Wolbachia

Moderadora

Ok. Pues ahora sí pasamos a los Wolbachia, verdad que sí. Mosquitos con Wolbachia. Wolbachia… se oye feo, verdad. WOL-BACHIA, pero es fácil, es una bacteria que vive en muchos insectos incluyendo algunas especies de mosquitos que pican a las personas. Pero la Wolbachia no se encuentra en los mosquitos *Aedes aegypti* que transmiten dengue, Zika y Chikunguña en Puerto Rico. La Wolbachia se introduce a los mosquitos *Aedes aegypti* en un laboratorio. Al presente, los estudios muestran que el uso de mosquitos infectados con Wolbachia es seguro para las personas, los animales y el ambiente. Los científicos creen que cuando hacen mosquitos con Wolbachia pueden ser menos capaces de transmitir enfermedades a las personas. ¿Entendieron lo que les leí? ¿Tienen alguna pregunta? Hasta ahora.

Azul

Ni me preocupa.

Moderadora

Pero ¿tiene alguna pregunta de lo que les leí? ¿Tienen preguntas? ¿Entendieron? Es una bacteria que se encuentra en el medio ambiente, verdad, que la tienen algunos insectos. Pero, el *Aedes aegypti* no lo tiene, hay que ponérselo en un laboratorio. Y esa bacteria hace que los mosquitos creen… pasen menos enfermedades a las personas. Es una bacteria que no le causa daño ni a las personas, ni a los animales, ni al ambiente, porque ya es una bacteria que está en el ambiente. Ok.

Azul

¿Y tienen resultados de esa…?

Moderadora

Habiendo dicho… Resultados. Información, resultados de lo que se h a encontrado, verdad. Ahora les leo. A base de lo que les leí, ahora les voy a explicar este otro método. Mosquitos macho y hembras con Wolbachia. Los mosquitos con Wolbachia funcionan de dos maneras diferentes. La primera manera, se liberan mosquitos *Aedes aegypti* machos y hembra con Wolbachia. Cuando el mosquito hembra con Wolbachia se reproduce, verdad, se casa, con un mosquito macho con o sin Wolbachia la bacteria se pasa a través de la hembra en sus crías, de generación en generación. Con el tiempo la cantidad de mosquitos con Wolbachia aumenta y reemplaza a los mosquitos del ambiente sin bacteria. O sea, se reemplazan los mosquitos del ambiente que no tienen la bacteria. Después de liberarlos varias veces la población de mosquitos con Wolbachia se mantendrá sin tener que liberar más de estos mosquitos. Los mosquitos con Wolbachia son menos capaces de transmitir enfermedades. Aun habrá mosquitos en la comunidad ya que la intención de este método no es reducir el número de mosquitos sino reducir el riesgo de enfermedades y de epidemias, perdón. Sin embargo, no se reducirán las picadas de mosquitos. Esta actividad se ha usado en otros países como Colombia y Brasil. Actualmente no hay reglas definidas para el uso de mosquitos macho y hembras, o sea, para esta primera forma de usarse, con Wolbachia en los Estados Unidos.

Pregunta 2- ¿Es esta una actividad nueva para usted o es algo que ya había escuchado antes? Si la escuchó, ¿dónde la escuchó?

Moderadora

¿Habían escuchado ustedes hablar de esta actividad anteriormente?

Gris

No.

Moderadora

¿No?

Blanco

Yo no.

Azul

No.

Pregunta 3- ¿Cree que esta actividad reduciría el número de mosquitos en su comunidad? ¿Por qué?

Moderadora

Ok. ¿Creen que esta actividad reduciría el número de mosquitos en su comunidad?

Azul

Ahí dice que no lo reduce. El mismo sistema no lo reduce.

Moderadora

¿Cree usted que reduciría el número de enfermedades?

Blanco

Bueno. Así sí.

Azul

Si está probado ya y científicamente está probado y hay resultados, no hay forma de no aceptarlo.

Blanco

El mosquito se va a quedar, lo único que no va a transmitir las enfermedades.

Gris

Las enfermedades…

Moderadora

Exacto, entendieron el punto. Qué bueno que entendieron lo que leí, me alegro.

Pregunta 3a- ¿Qué beneficios o ventajas tiene esta actividad para usted?

Moderadora

¿Qué beneficios y ventajas tiene esta actividad para ustedes?

Azul

No más preocupación por los contenedores con agua, con los criaderos, con las pailas.

Gris

Que no es una enfermedad como tal.

Azul

Porque lo que sería es acostumbrarse a las picadas y ya.

Moderadora

Y entonces, usted dice, él dice que no hay que estar preocupándose por los criaderos ni nada de eso, verdad.

Azul

No hay que preocuparse mucho ya.

Moderadora

Y ella dice…. ¿qué? Eso me interesó. ¿Qué usted dijo?

Gris

Se me olvidó…

Moderadora

Que no enferma.

Gris

Sí, que no…

Azul

Se controlan las enfermedades.

Moderadora

Sí, pero, usted dijo algo muy particular.

Gris

Se me olvidó…

Moderadora

¿Alguien lo tomó?

Sue

En la grabación se oirá…

Moderadora

Sí, pero es que yo quería discutir lo que ella dijo. Yo quería que ella abundara.

Coral

Fue muy bajito.

Moderadora

Yo entendí, que lo que ella dijo era, que como que la bacteria no enferma.

Gris

Sí, como que no es una enfermedad como tal.

Moderadora

La bacteria no es una enfermedad como tal. Exacto. Muy bien, muy bien. Y eso para usted es un beneficio.

Gris

Claro.

Moderadora

Ok. ¿Qué otros beneficios ustedes le ven por acá, si alguno?

Gris

Lo mismo….

Moderadora

¿Lo mismo?

Blanco

Lo mismo.

Gris

No causa daño.

Moderadora

No causa enfermedades.

Azul

Se reduce el riesgo de las enfermedades que causa el mosquito regular.

Pregunta 3b- ¿Qué desventajas o dificultades le ve a esta actividad? ¿De qué forma se podrían solucionar esas dificultades?

Moderadora

Ok. ¿Qué desventajas o dificultades le ve usted a esta actividad? Desventajas o dificultades.

Azul

Que siempre va a haber muchos mosquitos. Si se hace ese sistema, muchos mosquitos. Y la gente siempre va a vivir con la preocupación. Siempre van a decir que tienen esta preocupación de…porque no todo el mundo va a estar consciente de eso [la bacteria].

Gris

Y explicarles a las personas que es esa enfermedad. Que no hace daño.

Moderadora

Yo creo que hay que explicarles, qué es la bacteria. Porque la bacteria no es una enfermedad.

Azul

Lo que pasa es que yo voy… me pongo a explicar a una persona de eso y me va a decir que cuándo me gradué de científico.

Moderadora

O sea que, no lo podría hacer… la explicación no la podría dar un líder de comunidad.

Azul

Por eso, que uno lo puede hacer, pero la gente puede ponerlo en duda. Porque la gente no va a aceptar que yo les hable de algo que es científico.

Moderadora

Y entonces, ¿quién podría hablar de ese tema?

Azul

No sé, yo creo que ahí, para mí sería más un tipo de publicidad. Publicidad por los medios, que es donde es bien efectivo.

Moderadora

Ok. De eso vamos a hablar las últimas preguntas, pero me alegro que lo haya traído ahora.

Azul

Porque no hay otra forma para mí.

Pregunta 4- ¿Cuán posible es realizar esta actividad en su comunidad para reducir el número de mosquitos? ¿Por qué?

Moderadora

Ok ¿Cuán posible es realizar esta actividad en su comunidad?

Azul

¿Pero, qué tendríamos que hacer nosotros?

Moderadora

Esto es un método que no lo puede hacer una persona.

Azul

Pues por eso.

Moderadora

Esto el gobierno tiene que aprobarlo y lo tendría que hacer. Pero ¿hay alguna…?

Azul

O sea, yo te puedo decir que por lo menos yo, en mi comunidad, lo aceptaría. Lo aceptaría, tráiganlo y ni me lo digan, tírenlo por ahí. Que cuando empiece a bajar la incidencia no se va a dar cuenta. Pero no podemos hacer nada porque nosotros no podemos adquirir esos mosquitos y soltarlos.

Moderadora

No, eso es algo que no pueden hacer.

Azul

Eso es una actividad controlada. Yo creo que eso es la gente que tiene conocimiento y sabe bregar, saben lo que está haciendo.

Moderadora

¿Y ustedes creen que la… que ustedes apoyarían esta actividad en su comunidad? ¿Usted lo apoyaría? ¿Usted lo apoyaría?

Blanco

Lo apoyaría en mi comunidad, sí.

Rojo

Claro, porque podemos vivir más sano.

Blanco

Más cómodamente.

Azul

Yo todo lo que sea para mejorar y…

Moderadora

Y, usted, ¿apoyaría esta actividad?

Gris

Sí, claro.

Moderadora

¿Y la gente en la comunidad, apoyaría esta actividad?

Azul

Si se logran convencer de que lo que se está haciendo es para evitar que esos mosquitos transmitan, las enfermedades que matan, como mencionamos ahorita, o que enferman, yo creo que la gente va a aceptar. Pero es cuestión de convencerlos.

Gris

Llevar la orientación.

Azul

Es cuestión de convencerlos de que la gente esté convencida.

Gris

O llevar la orientación.

Moderadora

Hay que orientar a la gente…

Azul

Y eso no lo podemos hacer nosotros.

Moderadora

Y no lo puede hacer ustedes. ¿Qué tipo de información la gente necesitaría tener?

Rojo

Lo mismo. Que vinieran aquí y vieran todo lo que ustedes están haciendo hoy… para que aprendan.

Azul

Sí, pero la gente no va a venir.

Moderadora

¿Y cómo sería una forma de hacérselo llegar? Usted dijo, por…

Azul

Debe ser por publicidad.

Gris

Publicidad y dándoles *flyers*.

Moderadora

¿Dándoles *flyers*?

Gris

Y anunciándolo por la radio y la televisión para que la gente tome conciencia.

Blanco

Mayormente, si lo ponen por televisión es más efectivo.

Moderadora

Es más efectivo, ¿Por qué?

Blanco

Porque la gente no oye la radio y siempre ve es la televisión. Porque la televisión, mire, le voy a decir, hay juegos de pelota, hay novelas, hay películas, y la gente, hoy en día, inclusive a mí, no vemos la radio, siempre estamos pendientes a la televisión. Y la televisión es más efectiva.

Pregunta 5- ¿Apoya usted esta actividad en su comunidad? ¿Por qué? (*Preguntar a cada participante del grupo de discusión*)

Moderadora

Ok, pues ya concluí con esas preguntas, verdad.

Pregunta 5a- ¿Piensa que su comunidad apoyaría esta actividad? Sí, No, ¿Por qué?

[Se contestó en otra sección]

Pregunta 6- ¿Qué otra información necesitaría para entender mejor esta actividad?

[Se contestó en otra sección]

Actividad #7 – Mosquito macho con Wolbachia

Moderadora

Pues vamos a la próxima. Yo les dije que había dos maneras de usar los mosquitos con Wolbachia. ¿Verdad? Les dije que la primera se liberaba, verdad, se soltaban machos y hembras. Ahora en la segunda forma de usar esta actividad, solo se liberan mosquitos macho con Wolbachia. Se liberan mosquitos macho al ambiente, que no pican ni transmiten enfermedades. Se acuerdan de que cuando discutimos el ciclo de vida, yo les dije que los mosquitos machos no pican. As que pican son las hembras.

Blanco

Las hembras….

Moderadora

Ok. Los mosquitos macho con Wolbachia se unen a las, con las hembras sin Wolbachia, verdad, que hay en el ambiente. Las hembras sin Wolbachia ponen sus huevos, pero estos no nacen. ¿Verdad? Aquí lo ven. Está el mosquito macho que se une con la mosquita hembra del ambiente, ella pone sus huevos, pero los huevos no nacen. En la otra, que no les expliqué la… ya nos queda bien poquito. En la otra que yo les mostré, ambos mosquitos, el macho y la hembra, tienen Wolbachia, y cuando se producen con mosquitos, cuando se casan con mosquitos macho del ambiente, van a tener mosquitos, por eso los mosquitos no se acaban, pero esos mosquitos van a nacer con Wolbachia y no van a traer… tienen menos posibilidades de transmitir enfermedades. En estos, el macho se casa con la hembra del ambiente, la hembra va a poner los huevos, pero los huevos no van a nacer. No van a poder nacer. ¿Por qué? Porque la combinación de este… la bacteria con el de sin bacteria va a crear eso. Y entonces, los mosquitos con Wolbachia deben liberarse continuamente, en grandes cantidades para mantener baja las poblaciones de mosquitos. Una vez que los mosquitos con Wolbachia se dejan de ser liberados en un área, la población de mosquitos del ambiente vuelve a aumentar. Los mosquitos macho con Wolbachia se han utilizado en estudios en California, en los Cayos de la Florida. Y han sido aprobados para evaluación en Miami, Florida. Esa es otra diferencia que tienen con los machos y hembras, verdad. Cuando se liberaban los dos. Aquellos se habían hecho en otros países del exterior, pero no tenían… no se habían aprobado reglas en los Estados Unidos. Los mosquitos macho, sí tienen aprobación para usarse en los Estados Unidos. Verdad que sí. Y se han utilizado. Y entonces, estos mosquitos sí van a bajar las cantidades de mosquitos. En el otro iba a haber siempre mosquitos que iban a picar, en esta bajan las cantidades de mosquitos. Pero, a diferencia de aquellos, cuando se tiran machos y hembras, ¿qué pasa? Se tiran por un tiempo y después se pueden dejar de tirar. Porque reemplazan las poblaciones de mosquitos del ambiente. Estos, se tienen que estar liberando constantemente porque si se dejan de liberar, entonces, sube de nuevo la población de mosquitos.

Azul

Y si sube la población de mosquito, sube sin la bacteria o…

Moderadora

Sí.

Azul

Una vez tú los liberaste y se van eliminando, los que nacen, ¿nacen nuevecitos?

Moderadora

Mientras se estén liberando van a casarse con las hembras del ambiente y los huevos no van a nacer. Una vez se dejan de liberar los mosquitos macho…

Azul

Sí, porque la hembra no tiene.

Moderadora

La hembra se va a casar con los mosquitos macho del ambiente y van a volver a nacer más mosquitos.

Azul

Sí porque los machos que están no son que nacieron de ese mosquito, sino que ya estaban.

Moderadora

Exacto.

Azul

Y cuando te hablan de eso, ¿qué cantidad se produce de esos mosquitos para liberar? ¿Eso se produce… es fácil de sacar muchos? O eso es uno a uno…

Moderadora

Ponte esa otra pregunta ahí. Eso es una pregunta, verdad, que la información se le debería de dar a la gente es una pregunta…

Azul

Porque si tú dices, ‘mira, se reproducen mil por minuto…’, o qué sé yo. Uno a uno, ponerlo ahí…

Moderadora

Pues eso se debería… esa es una información que tendríamos que dar. Después lo puedo contestar. ¿Ok?

Blanco

Esa bacteria, ¿cómo se produce?

Moderadora

Esta en el ambiente.

Azul

En otros insectos…

Gris

No hace daño….

Blanco

O sea, ¿No puede ser creada en un laboratorio?

Moderadora

Ah ok. No porque usualmente esa bacteria se hace… está en el ambiente y se recoge del ambiente. Ahora, eso es una información que usted diría que habría que darle a la gente. Si esa bacteria se va a recoger del ambiente o si se va a reproducir en un laboratorio.

Blanco

Exacto. Si se produce en algún laboratorio sería más efectivo.

Moderadora

Ah, a usted le gusta la idea de que se cree la bacteria en laboratorio.

Blanco

Seguro. Si se puede crear porque hay muchos adelantos últimamente y si se pudiera crear, y traerla a los sitios donde haya mosquitos, esa bacteria. Y se riega la bacteria, porque la bacteria no es nociva pues se puede regar.

Moderadora

Sí, pero, la bacteria, no todos los animales, no todos los insectos la van a coger. Tienen que ponérsela.

Blanco

No, no. Estamos hablando del mosquito.

Moderadora

Ok. Ok.

Azul

La ventaja es que, si se puede lo que él dice, en un laboratorio, pues compensa la falta de la bacteria en animales que no lo tengan disponible. Garantiza que hayan más…

Pregunta 2- ¿Es esta una actividad nueva para usted o es algo que ya había escuchado antes? Si la escuchó, ¿dónde la escuchó?

Moderadora

Ok. Esta actividad no la habían escuchado antes. Este método.

Azul

No. No.

Moderadora

No lo habían escuchado antes.

Pregunta 3- ¿Cree que esta actividad reduciría el número de mosquitos en su comunidad? ¿Por qué?

Moderadora

Y, ¿cree que este método reduciría el número de mosquitos en la comunidad?

Azul

Mientras se esté produciendo el mosquito, sí.

Moderadora

Mientras se estén liberando mosquitos con Wolbachia machos…

Azul

Si no, pues, volvemos a lo mismo, entonces, a poner las trampas, sacar los envases.

Pregunta 3a- ¿Qué beneficios o ventajas tiene esta actividad para usted?

Moderadora

Y entonces, ¿qué beneficios o ventajas tiene esta actividad para ustedes? ¿Usted, le ve algún beneficio a esta actividad?

Rojo

Pues nos ha servido mucho.

Moderadora

No, pero a este método. A este método. A tirar mosquitos macho con Wolbachia. ¿Usted le ve alguna ventaja?

Gris

Sí, porque no trae enfermedad.

Moderadora

No trae enfermedad…

Gris

No trae, porque es una bacteria.

Azul

Y llega… para mí llega a todos los mosquitos que están vivos, que no se pudieron eliminar. Porque si esos mosquitos interactúan con todas las mosquitas que están…mosquitas no, los zancudos, los *Aedes…* si se cruzan con todas las que están vivas, la ventaja es que va a ir directamente a todos. No es un método que puede eliminar a algunos y a otros no, ahí los va a coger todos. Ahora, ¿hasta cuándo va a seguirse produciendo para que no pase más? Porque el día que se acabe, nos quedamos sin Wolbachia y volvemos a lo mismo.

Pregunta 3b- ¿Qué desventajas o dificultades le ve a esta actividad? ¿De qué forma se podrían solucionar esas dificultades?

Moderadora

Ok. Y entonces…

Azul

La desventaja, perdona…

Moderadora

Ajá, la desventaja.

Azul

La desventaja que yo veo es eso, que depende de la capacidad que haya para producir la… para introducir la bacteria en eso…

Gris

El tiempo.

Blanco

Exacto.

Moderadora

O sea, que la desventaja sería, y todos están de acuerdo, en que… sería en que, cuánto tiempo se haría esto y la capacidad de poder hacer…

Azul

De hacerlo. Y la disponibilidad.

Moderadora

…de introducir la bacteria y tenerla, verdad, en el ambiente circulando.

Pregunta 4- ¿Cuán posible es realizar esta actividad en su comunidad para reducir el número de mosquitos? ¿Por qué?

Moderadora

Ok. ¿Cuán posible es realizar esta actividad en su comunidad para reducir el número de mosquitos? ¿Usted ve que es posible hacer esto en su comunidad?

Azul

Sí, yo creo que sí.

Blanco

Sí. En el área donde yo vivo sí. Porque hacer eso es bien factible porque ahí donde yo vivo, pues, como está el caño del río. Y ahí olvídese que eso es…

Azul

Yo creo que sí, porque si tu traes mil mosquitos de esos, con esa bacteria va a haber como tres mil mosquitas esperando para casarse. Así que va a haber demás. [risas] Porque tenemos muchas.

Moderadora

Ay que chévere, eso está chévere.

Pregunta 5- ¿Apoya usted esta actividad en su comunidad? ¿Por qué? (*Preguntar a cada participante del grupo de discusión*)

Moderadora

Y entonces, ¿apoyaría usted esta actividad en su comunidad?

Blanco

Por qué no…

Moderadora

¿Usted lo apoyaría?

Rojo

[*Anotadora presencial tiene en sus notas que todos apoyarían la actividad.]

Moderadora

¿Usted lo apoyaría?

Gris

[*Anotadora presencial tiene en sus notas que todos apoyarían la actividad.]

Moderadora

Y usted lo apoyaría.

Azul

Sí…

Pregunta 5a- ¿Piensa que su comunidad apoyaría esta actividad? Sí, No, ¿Por qué?

Moderadora

¿Cree que su comunidad apoyaría esta actividad en su comunidad?

Azul

Yo creo que sí.

Blanco

Sí.

Moderadora

¿Lo apoyaría?

Gris

Claro que sí.

Blanco

Sí.

Pregunta 6- ¿Qué otra información necesitaría para entender mejor esta actividad?

Moderadora

¿Qué información necesitarían tener?

Azul

Como eso es información científica es cuestión de decirle a la gente, ‘nada, van a ver lo efectos, lo van a ver…’.

Moderadora

Usted dijo ahorita que… cuando estábamos explicando anteriormente, que se necesitaría saber la efectividad.

Azul

Eso es lo que más me interesaría saber a mí. Si eso en realidad… si hay ya información, si tienen data ya de que ha sido efectivo, de que es efectivo, de que en verdad reduce o elimina los mosquitos. En ese caso si en verdad no nace más ninguno.

Actividad #8 – Mosquitos modificados genéticamente

Moderadora

Ok. Vamos para la última entonces. Esta es la última verdad. No tenemos más nada… exacto, esta es la última. Mosquitos modificados genéticamente. Se liberan mosquitos macho para que se unan con mosquitos hembra del ambiente. Los mosquitos macho modificados genéticamente se reproducen, se casan, con las hembras del ambiente y pasan un gen a su cría que impide que las larvas y pupas se desarrollen normalmente. Así, estas mueren antes de convertirse en mosquitos adultos. Los mosquitos macho liberados no pican ni transmiten enfermedades. Estos mosquitos se liberan varias veces a la semana a lo largo del tiempo, en grandes cantidades para mantener bajas las poblaciones de mosquitos *Aedes aegypti* solamente. Una vez los mosquitos modificados genéticamente *Aedes aegypti*, dejan de ser liberados en un área, la población de mosquitos aumentará de nuevo. Los mosquitos modificados genéticamente han sido evaluados en países incluyendo las Islas Caimán, Brasil y Panamá. Al presente no se han hecho estudios de los mosquitos modificados genéticamente en los Estados Unidos. Aquí usted ve que se le introduce, verdad, se le introduce al mosquito un gen. ¿Verdad? Y entonces, ese mosquito que es macho se casa con una hembra del ambiente, la hembra va a parir los mosquitos y las larvas van a salir y van a salir las pupas también, pero, ¿qué les va a pasar? Se van a morir. Se van a morir, no van a poder llegar a ser adultos. Verdad, porque tienen ese gen que hace que se mueran. ¿Alguna cosa aquí que no entendieron? ¿Entendieron?

Pregunta 2- ¿Es esta una actividad nueva para usted o es algo que ya había escuchado antes? Si la escuchó, ¿dónde la escuchó?

Moderadora

Ok. ¿Habían oído hablar ustedes de esta actividad? Mosquitos genéticamente modificados.

Gris

¿Qué es eso, que traen mosquitos, es?

Moderadora

No. Lo que pasa es… le explico. No, está muy bien que pregunte.

Gris

Sí, porque me está como curioso, de modificar.

Moderadora

Sí, porque lo que pasa es que todo el mundo, verdad, todo, humano, todas las especies de los animales del mundo tienen un código genético, verdad, que es lo que dice, cómo nosotros somos. Verdad, ese código genético se llama RNA o DNA, verdad. Y entonces, lo que se hace es que, a ese código genético se le añade otro gen más. Se le añade un gen. Y ese gen es para que el mosquito no pueda… se muera. Punto.

Gris

¿Y eso lo hacen en un laboratorio?

Moderadora

Sí. Sí. Eso lo hacen en un laboratorio. Y entonces, esos mosquitos se sueltan en el ambiente, se casan con las mosquitas hembras del ambiente y, las mosquitas van a parir, van a parir igualito, pero los mosquitos de esa hembra no se van a dar.

Azul

Nacen, pero no…

Gris

Mueren.

Moderadora

No van a llegar a ser adultos. Se van a morir. Y entonces, lo importante es que se ha hecho en otros países. En las Islas Caimán, en Brasil y en Panamá. Pero en Estados Unidos, no hay estudios de eso.

Azul

Que, si lo hacen aquí, nos van a usar de laboratorio.

Moderadora

¿Eso sería una desventaja para usted?

Azul

Bueno. Lo que pasa es que, si está probado y ya hay información de que está probado, no es desventaja. Porque, al contrario, lo que queremos es eliminarlos. Ahora, yo les pregunto, ese mosquito que le ponen ese gen, la probabilidad de vida de ellos, ¿es un 100%?

Moderadora

Esa es otra pregunta. ¿Cuánto viven los mosquitos que se les pone ese…?

Azul

Que, no vaya a ser que los traigan y…

Gris

Sea peor.

Azul

…y en dos días se mueran y las mosquitas queden…

Blanco

Viudas.

Azul

…solteras [risas], se queden viudas.

Moderadora

Eso está bueno. Eso está bueno. Esa es una información que…

Azul

O sea, después que se haga el trabajo que ese mosquito, y nosotros pensando que estamos con un proyecto que nos puede liberar, que en realidad no sea así.

Moderadora

Y resulta que no es.

Azul

Que sea que el mosquito no está… Pero, ya me imagino que tiene que haber pruebas, verdad, que ese mosquito…

Moderadora

Se ha probado en las Islas Caimán, Brasil y Panamá.

Azul

O sea, que sí… lo que quiere decir…

Moderadora

Donde no se ha probado es en los Estados Unidos.

Gris

¿Y han tenido resultado?

Moderadora

Esa es otra pregunta. El resultado, la efectividad que han tenido esos mosquitos…

Azul

Por eso, porque…

Moderadora

…en otros países.

Azul

…eso es bueno saberlo. Ahí es donde uno puede tomar decisiones, por lo menos tener esperanza.

Gris

Claro.

Moderadora

Ok. Muy bien. Pero, ¿han escuchado esta actividad en otro lugar?

Azul

No.

Gris

No.

Rojo

No.

Moderadora

No. La primera vez.

Pregunta 3- ¿Cree que esta actividad reduciría el número de mosquitos en su comunidad? ¿Por qué?

Moderadora

¿Creen ustedes que esta actividad reduciría el número de mosquitos en su comunidad?

Rojo

Sí…

Azul

Yo tendría que ver… Que me den información de la prueba que ya tienen. Ahora, lo que me preocupa de esto es… o, ¿viene la pregunta, verdad, desventajas?

Moderadora

No, no, usted dígame, dígame. ¿Qué es lo que le preocupa?

Azul

Que como en el anterior caso, dice que los mosquitos modificado genéticamente, este, dejan de ser liberados en el área, la población de mosquitos aumentará de nuevo.

Moderadora

Exacto.

Azul

O sea, el día que no se haga, cuando se acaben los mosquitos alterados, o se acaben los mosquitos con la bacteria, volvemos a lo mismo.

Moderadora

Eso sería una desventaja.

Azul

Eso sería una desventaja porque el mosquito es para toda la vida. Y no sé si eso lo van a hacer toda la vida. Yo creo que es mejor eliminarlos y…

Pregunta 3a- ¿Qué beneficios o ventajas tiene esta actividad para usted?

Moderadora

Ok. ¿Le ve usted algún beneficio a esta actividad?

Gris

Yo creo que sí, porque los elimina a todos.

Moderadora

Y entonces, la desventaja sería…

Azul

El beneficio de que los elimina es relativo al objetivo que sea y al tiempo que… porque los puede eliminar, pero si en dos años no lo van a hacer más, si cambian el programa o deja de existir CDC o los Vectores, qué sé yo, y no hay quien bregue con eso, vamos a tener mosquitos otra vez.

Moderadora

Ok. Y entonces, ¿ustedes les ven esas mismas ventajas y desventajas? O, ¿quieren añadir alguna más?

Blanco

Sí, las mismas.

Moderadora

Le ven igual.

Pregunta 3b- ¿Qué desventajas o dificultades le ve a esta actividad? ¿De qué forma se podrían solucionar esas dificultades?

[Se contestó en otra sección]

Pregunta 4- ¿Cuán posible es realizar esta actividad en su comunidad para reducir el número de mosquitos? ¿Por qué?

Moderadora

¿Cuán posible sería realizar esta actividad en su comunidad? ¿Usted ve posible que esto se pueda hacer en sus comunidades?

Azul

Yo creo que sí.

Gris

Sí.

Azul

Me parece que son acti… este tipo de actividad son actividades donde la gente no van a tener acción en esto.

Moderadora

Exacto,

Azul

Eso es, eso tú lo llevas allí, ni se enteran, lo soltaste, y empiezan… hagamos la cuenta, la gente ni cuenta se va a dar de que hay menos mosquitos o que hay menos…

Moderadora

Pero se tiene que orientar a la gente.

Gris

Por eso orientarles…

Moderadora

Se tienen que orientar.

Azul

Sí, pero, la orientación lo que mencionamos ahorita, eso es importante.

Moderadora

De hecho, estos grupos de discusión que estamos haciendo es por si en la eventualidad de que el gobierno tenga el dinero y la disposición para hacerlo, saber qué opina la gente.

Azul

Sí, sí, yo entiendo.

Moderadora

Para hacerlo, verdad.

Azul

Pero, todo esto, como lo mencionamos ahorita, nosotros, en estas cosas que son científicas, no podemos dar mucho detalle, pero yo creo que la publicidad.

Moderadora

Hay que orientar.

Azul

Promoverlo y orientar a las personas. Orientar, que la gente sepa.

Pregunta 5- ¿Apoya usted esta actividad en su comunidad? ¿Por qué? (*Preguntar a cada participante del grupo de discusión*)

Moderadora

Ok. Y entonces. ¿Apoyaría usted esta actividad en su comunidad?

Blanco

Sí.

Moderadora

Si le dijeran que van a soltar estos mosquitos en su comunidad, ¿lo apoyaría?

Blanco

Sí.

Gris

Claro que sí.

Moderadora

¿Lo apoyaría?

Gris

Claro.

Azul

Yo apoyo 100% todo lo que me diga a mí que va a reducir. Sea en mayor o menor grado, los mosquitos yo lo apoyo.

Rojo

[Anotadora presencial tiene en su nota que el participante #6 (Rojo) apoyaría la actividad.]

Pregunta 5a- ¿Piensa que su comunidad apoyaría esta actividad? Sí, No, ¿Por qué?

Moderadora

Y entonces, ¿piensa que su comunidad apoyaría esta actividad?

Blanco

Después que tengan la orientación suficiente, yo creo que sí.

Azul

Yo creo que sí, en todo… yo en la única que yo tengo reserva es en la de los fumigadores profesionales, pero aun así entiendo que…

Moderadora

Ok. Pues ese es mi última… esa es una pregunta que tengo… Ok.

Pregunta 6- ¿Qué otra información necesitaría para entender mejor esta actividad?

Moderadora

Le pregunto…. Ya me dijeron que información que se necesitaría tener, usted hizo una pregunta que cuántos mosquitos se tirarían y si esa actividad es algo que se podría sostener, verdad, se podría mantener a través del tiempo.

Azul

Exacto, eso es lo más importante.

Moderadora

Esa información, y también los resultados en otros países. Verdad que sí.

Azul

Sí. Sí.

Blanco

Exacto.

Moderadora

Esa es la información. ¿Hay alguna otra información que necesitarían?

Gris

No.

Rojo

No.

**Parte 3- Cierre de sesión**

Pregunta 10- ¿En quién de su comunidad confiarían ustedes para hablar sobre estas actividades?

Moderadora

Ok. Y entonces, de todas… ah, espérese, antes de que termine. ¿En quién de su comunidad confiarían ustedes para hablar sobre todos estos métodos?

Blanco

¿En quién?

Moderadora

¿En qué persona de su comunidad, confiarían ustedes para hablar sobre estas actividades? Sobre estos método.

Azul

O sea, tenemos que darte un nombre ahora.

Moderadora

¿Ah?

Azul

¿Nos estas pidiendo un nombre?

Moderadora

No. Me puede decir líderes de comunidad o sencillamente no hay nadie y lo tiene que hacer un profesional certificado.

Azul

Yo pienso más… yo pienso… lo que pasa es que otra característica de PL, es que nosotros somos un barrio, pero somos sectoriales. Aquí la gente de ________, ellos son un mundo aparte, nosotros… Y yo entiendo que cada comunidad tiene sus personas.

Moderadora

¿Tiene sus personas?

Azul

Sí.

Moderadora

Y en la comunidad… no me tiene que decir un nombre, solamente dígame si es un líder de comunidad o así. En su comunidad, ¿Quién sería una persona para llevar esta información? O, ¿piensa que debería ser un profesional?

Blanco

Creo que sí. Un profesional debe ser.

Rojo

Yo creo que sí, creo que eso es mucho mejor.

Blanco

Un profesional.

Moderadora

¿Un profesional? ¿Y en su comunidad también?

Gris

Eso, que le explique bien a la comunidad.

Moderadora

Que le explique bien a la comunidad. ¿Y en su comunidad?

Azul

Puede ser un profesional o un líder comunitario, la cosa es que tenga la información adecuada y que la gente esté dispuesta a escucharlo y que se cree el ambiente para que la persona esté disponible para recibir….

Pregunta 11- ¿Cuál sería la mejor manera de hablar a su comunidad sobre estas actividades?

Moderadora

Ok. ¿Cuál sería la mejor manera de hablar en su comunidad sobre estos métodos? ¿Cuál sería la mejor? Usted dijo que promoción. Usted dijo la televisión. Usted dijo, ¿qué más? Usted dijo *flyers* ahorita.

Azul

Pero si hablamos, por ejemplo, nosotros, aislando la Playa de todo Ponce, yo pienso aquí que el mejor método es ir casa por casa.

Moderadora

Casa por casa.

Gris

Sí.

Azul

Casa por casa y llevarle la información y a la medida que uno pueda explicarles a las personas, hablarles.

Moderadora

Ok, y entonces. Ahora sí les pregunto. De todos estos métodos, cada uno le voy a preguntar individual, ¿cuál fue el más que le gustó? De todos estos. O, los más que les gustó. Puede ser más de uno.

Rojo

Me gustó todos.

Moderadora

¿Todos les gustaron?

Rojo

Sí.

Moderadora

¿Y usted? Hablamos de la reducción de criaderos, hablamos del uso de larvicidas, verdad que la persona lo haga, del uso de larvicidas en *trucks*, hablamos del insecticida residual en las paredes, hablamos de las trampas…

Blanco

La trampa, es la más que…

Moderadora

¿La trampa fue la que más le gusto?

Blanco

Sí, porque es más conveniente y es más mejor para uno trabajarla, es más sencilla.

Moderadora

Ok. ¿Y después? ¿Hay otro que además de la trampa le haya gustado? Ok. ¿Y usted?

Blanco

Déjeme virar el tape para atrás. [risas]

Moderadora

Y usted, ¿cuál fue el que más le gustó?

Rojo

Me gustó ese. [GMO mosquitos]

Moderadora

Ese, el de los mosquitos genéticamente modificados.

Rojo

Seguro, porque no… ¿Cómo se llama? No nos contagian.

Moderadora

No les contagian. Ok. ¿Y usted, cuál le gustó más?

Gris

A mí me gustó éste [GMO mosquitos], me gustó el de la trampa.

Moderadora

Ese y el de la trampa. Ok, y a usted, ¿cuál le gustó más?

Azul

A mí el de la trampa y el de eliminar criaderos, el primero, el primerito. Que es donde la gente se tiene que envolver.

Moderadora

Ok. Había unas preguntas que yo dije que podía contestar…

*****Fin del audio*****
