# Supplementary material for: Acceptability of emergent Aedes aegypti vector control methods in Ponce, Puerto Rico: A qualitative assessment
Source: PLOS Glob Public Health. 2024 Mar 6;4(3):e0002744. doi: 10.1371/journal.pgph.0002744 (PMC10917327; doi:10.1371/journal.pgph.0002744)
Supplement: S2 Appendix — (ZIP) [file pgph.0002744.s002.zip › S2A_Appendix.docx]

**S2 Appendix. Anonymized Transcripts of Group Discussions (Spanish)**

**COPA Grupo de Discusión con Líderes y Residentes sobre Actividades de Control de Vectores en los Clústeres GL, ST y CL**

Fecha y hora- 23 de abril del 2018; 7pm

Lugar- Centro Comunal de GL

Moderador- Carmen Pérez

Anotadores- Sue Ramos y Jessenia

Transcriptor- Sue Ramos

Participante #1- EST (H) - P#1EST

Participante #2- EST (H) - P#2EST

Participante #3- EST (M) – P#3EST

Participante #4- EST (M) - P#4EST

Participante #5- JC (H) - P#5JC

Participante #6- JC (M) - P#6JC

Participante #7- FB (H) - P#7FB

Participante #8- ST (H) - P#8ST

Participante #9- GL (H) - P#9GL

Introducción- Se comenzó leyendo la introducción de la guía de pregunta del grupo de discusión, y se les preguntó a los participantes si tenían preguntas sobre lo leído. Todos afirmaron no tener preguntas. Se pasó a leer el consentimiento informado con los participantes. Todos confirmaron ser mayores de 21 años y todos aceptaron a que se grabara la discusión y participar en el grupo. Luego se leyeron las reglas del grupo y se le asignó número a cada participante.

Parte 1- Conocimiento sobre enfermedades transmitidas por mosquitos

Moderadora

Y vamos a empezar con las preguntas. Ah, ¿Número uno (*P#1EST*) de qué comunidad es?

P#1EST

EST… EST.

Moderadora

¿Y _____… ah….?

P#2EST

EST.

Moderadora

Número dos (*P#2EST*)…

P#2EST

EST.

Moderadora

Ve, que a uno se le sale porque ya conoce la persona. Número tres.

P#3EST

ST.

Moderadora

ST…

P#4EST

EST.

Moderadora

ST…

P#5JC

Pues… JC y JM.

Moderadora

JC…

P#6JC

JC.

Moderadora

JC…

P#7FB

FB.

Moderadora

FB…

P#8ST

ST.

Moderadora

ST… Muy bien.

Pregunta 1- ¿Qué ha escuchado sobre el dengue, Zika y Chikunguña?

Moderadora

Eh… ahora les pregunto. Eh, ¿qué han escuchado ustedes, sobre el dengue, Zika y el Chikunguña? Es decir, ¿cómo se transmiten y cuán peligrosas son?

P#1EST

Una picadura…

P#7FB

Es simple… simplemente ellos hablan de que no haiga pozos de agua en los hogares, que el mosquito se procree. O sea, que eso es lo único que dicen. En la televisión. No dicen constantemente el peligro que suele ser a las personas. Uno lo tiene que averiguar. O sea, que la televisión la información es breve, treinta segundos y ya ahí se le acabó el tiempo y a la persona, y no da. No dan completamente la información.

Moderadora

Muy bien.

P#7FB

Lo que hemos averiguado, estoy seguro que ha sido por boletines. Que indica la información… lo grave que es la picada del mosquito, porque eso crea cuatro enfermedades. A mí me dio una, me dio la del Chikunguña. Y…

Moderadora

Ah ok. ¿Y usted dice que se transmiten…?

P#1EST

Por la picada del mosquito.

Moderadora

Por la picada del mosquito.

P#5JC

El agua es… el agua es clara. No es que sea agua sucia. Sino agua clara…

P#7FB

Pero ellos dicen que también se procrea en el agua sucia. Por… Ve.

Moderadora

Sí. ¿Alguna cosa…? Ajá.

P#6JC

El mosquito se llama *Aedes aegypsi* [*aegypti*].

Moderadora

Exacto.

P#6JC

En estas… boletines que ponen en diferentes sitios, que uno se detiene a leer y esta… las tres enfermedades son bien peligrosas porque todo depende la persona, el receptor, de las enfermedades que padece, cuán frágil o sensible es, pues se puede complicar los eh… síntomas.

Moderadora

Ok.

P#7FB

Sí…

P#6JC

Eh, también otra información importante es, pues, seguir las instrucciones de protegernos, ponernos repelentes, usar mangas largas en los sitios… sobre todo que no estén oscuros. Pero que estén claros y bien ventilados. Porque el mosquito mientras más oscuro, más se queda, más se esconde. Y hay horas que son más propensas para picar, ellos.

Moderadora

¿Alguien quiere añadir alguna otra cosa?

Sue

Permiso, quería decir… traten de decir el numerito que tienen antes de hablar, para nosotros poder saber porque como son muchos…

P#6JC

Ah pues sí. Está bien.

Moderadora

Son ocho, que bueno, me gusta. ¿Alguien quiere decir algo más sobre qué es el Zika, el dengue, y el Chikunguña, cómo se transmiten, cuán peligrosas las consideran? ¿Estamos bien?

P#7FB

Yo iba a decir, que a mí me dio el Chikunguña, y…. me duró menos tiempo, porque yo tenía un té, de esos de productos naturales, llamado Cola de Caballo. Y yo dije, ‘bueno, este té puede funcionar’. En los síntomas, empecé a sentir el jueves, un jueves, luego el viernes me tumbé, me sentí con todos esos síntomas malísimo, destruyendo… como destruyéndome en los huesos, los músculos. Y yo dije, ‘pero si yo tengo un té ahí que podría funcionar, que dice que es para los músculos y coyunturas…’, me doy un palo de ese té. Me lo preparé con incomodidad, no como… con facilidad porque ya me había afectado las coyunturas. Me tomé un té el viernes, otro té el sábado y el domingo estaba listo para jueguito de dóminos.

Moderadora

Ay que bien.

P#7FB

O sea que el té…

Moderadora

Funcionó.

P#7FB

…funcionó.

P#5JC

Funcionó…

P#7FB

Lo vende… las tres A allá cerca… no se si ustedes saben… los almacenes tres A de la FB, pues, ahí los tienen, en una sucursal de medicina natural. No es que le haga promoción, sino que el té a mí me funcionó. Que le funcione a otro, yo no lo sé.

Pregunta 1a- ¿Cómo se transmiten?

[se contestó más arriba]

Pregunta 1b- ¿Cuán peligrosas son?

[se contestó más arriba]

Parte 2- Reacciones a las actividades de control de vectores

Moderadora

Claro. Bueno, entonces, ya habiendo aclarado, verdad, ustedes dándome sus opiniones, de qué es el dengue, Zika y el Chikunguña, ahora vamos a entrar a la parte de reacciones de ustedes a las actividades de control de vectores. Para comenzar les voy a demostrar un vídeo que explica el ciclo de vida del mosquito *Aedes aegypti*. Esta explicación le ayudará a entender mejor cómo funcionan las actividades para el control de mosquitos. ¿Ok? Vamos al vídeo. Dice, el mosquito *Aedes aegypti* macho no pica, que quiere decir que eso que está picando es una…

P#7FB

Hembra.

Moderadora

Hembra. Pero la hembra, pica una persona para obtener su sangre y producir sus huevos. Mira cómo se le va llenando la barriguita. ¿Lo ven? Está picando y mira cómo se le va llenando la barriguita de sangre.

P#6JC

Sí.

Moderadora

El mosquito hembra, pone sus huevos en las paredes de cualquier envase con agua acumulada dentro o fuera del hogar.

P#7FB

Dos o tres días… [lo dice muy bajito, pero se captura en el audio]

Moderadora

Estos huevos pueden durar hasta ocho meses pegados a las paredes del envase. Cuando los huevos se sumergen en el agua nacen las larvas. Eso es una larva naciendo.

P#7FB

[no se entiende el comienzo]… de tres días…

Moderadora

Las larvas viven en el agua donde se alimentan y se convierten en pupa… van a ver ya mismo la pupa, en aproximadamente cinco días. O sea que la larva está en el envase comiendo. El agua no puede ser totalmente limpia porque tiene que tener comida que son hojas… sucio para que la larva coma. Y ahí está naciendo la pupa, ve. Esa es la pupa. De dos a tres días, la pupa se convertirá en los mosquitos adultos. Miren cómo está naciendo de la pupa el mosquito adulto. Y nació. Se toma de siete a diez días desde que un huevo de mosquito se en un mosquito adulto. Por eso es que ustedes han escuchado que semanalmente usted tiene que ir al patio a buscar sus envases con agua y a eliminarlo, para que ese ciclo no se complete.

P#7FB

Yo no le doy tanto tiempo…

Moderadora

¿Ah?

P#7FB

Yo no le doy tanto tiempo, yo lo viro…

Moderadora

Rápido, verdad. Ok. ¿Entendieron el ciclo de vida? Lo vieron, ¿verdad qué impresionante?

P#5JC

Ajúm.

P#6JC

Oh sí.

Moderadora

Como la larva sale. Verdad, del huevo.

P#6JC

Sí.

Moderadora

Sí… Se están desarrollando muchas formas de reducir el número de mosquitos en el ambiente. Algunas acciones pueden ser realizadas por los residentes y las comunidades, y otras pueden ser realizadas por profesionales de control de mosquitos y/o el gobierno. Ahora les mostraré unos dibujos que describen las actividades que se podrían realizar y cómo podrán ayudar. Luego les voy a hacer una serie de preguntas para obtener su opinión sobre cada actividad. Empezamos con la primera, Karla (anotadora).

[se presentan a Karla y Gladys como parte del equipo de CDC]

Actividad #1 – Reducción de fuentes de mosquitos

Moderadora

Ok. Esta es la primera actividad, verdad, la primera acción para reducir los mosquitos en sus comunidades. Se llama reducción de fuentes de mosquitos. La reducción de fuentes es la eliminación de lugares donde los mosquitos ponen sus huevos. El municipio y su comunidad trabajarían juntos para eliminar, vaciar y recoger los envases con agua acumulada que puede hacer criaderos de mosquitos en áreas públicas. Dentro de su hogar y patio, usted cubriría, vaciaría o eliminaría los envases que acumulan agua como los tiestos, latas, gomas y desagües. ¿Qué ustedes ven ahí? En la foto.

P#1EST

Gomas…

Moderadora

Gomas, verdad.

P#2EST

Sacar cosas…

Moderadora

¿Qué hace el hombre con las gomas?

P#2EST

Sacándolas.

P#1EST

Sacándolas.

Moderadora

Sacándolas. ¿Qué más ven?

P#4EST

Envases tapados.

Moderadora

Envases tapados, verdad, que es este… el zafacón.

P#4EST

Vaciando tiestos.

Moderadora

Vaciando tiestos. ¿Quién es? La mujer embarazada, verdad. ¿Y abajo?

P#4EST

Recogiendo.

Moderadora

Recogiendo la basura. Muy bien. Bueno.

Pregunta 2- ¿Es esta una actividad nueva para usted o es algo que ya había escuchado antes? Si la escuchó, ¿dónde la escuchó?

Moderadora

¿Es esta… es esta actividad algo nuevo para ustedes o es algo que ya habían escuchado?

P#1EST

Para mí es nuevo.

Moderadora

¿Para usted es nuevo?

P#1EST

Digo, así, así. En reunión, como estamos ahora.

Moderadora

Ok. Para usted es nueva. Y… los que… ¿alguna otra persona lo había escuchado?

P#2EST

Sí.

P#4EST

Sí, sí.

Moderadora

¿Dónde lo habían escuchado?

P#2EST

Por las noticias. La televisión. Y hojas sueltas que han pasado dentro de la comunidad.

P#7FB

En la televisión. Ahora, que lo apliquen las personas es otro asunto.

Moderadora

Que lo apliquen las personas es otro asunto.

P#2EST

Eso es así.

Moderadora

Verdad que sí. Pues eso mismo es lo que le voy a preguntar ahora.

Pregunta 3- ¿Cree que esta actividad reduciría el número de mosquitos en su comunidad? ¿Por qué?

Moderadora

¿Cree que esta actividad reduciría el número de mosquitos en su comunidad?

P#6JC

Claro que sí.

Moderadora

¿Por qué? ¿Por qué me dice que sí?

P#4EST

Porque estamos contribuyendo a que no se propaguen, a que no crezcan ni se propaguen.

Moderadora

Ok. Otro que me diga que sí.

P#2EST

Sí, eso es así. Ayudar a que…

P#7FB

Pero es si lo hacen…

Moderadora

Ahí vamos ahora.

P#7FB

Si lo hacen las personas.

Moderadora

Exactamente. Ya mismo vamos a llegar ahí.

Pregunta 3a- ¿Qué beneficios o ventajas tiene esta actividad para usted?

Moderadora

¿Qué beneficios o ventajas tiene esta actividad para ustedes?

P#2EST

Aprendemos lo… lo…

P#7FB

Lo que debemos hacer.

P#2EST

… que no sepamos… lo vamos a aprender aquí para llevarlo a la comunidad.

P#6JC

Alentarnos a que todo esto, no solo verlo y contemplarlo, sino llevarlo a cabo, completar en acción.

Moderadora

Ok. Ok. ¿Alguna otra ventaja? ¿O beneficio que tiene?

P#7FB

Sí, aprendemos a que… lo que debemos hacer con ese mosquito.

Moderadora

Ah ok. ¿Y usted?

P#8ST

Si llevamos a cabo esa actividad no nos vamos a enfermar y nos…

Moderadora

Exactamente.

P#5JC

Sí, el eliminar el… donde se producen los mosquitos pues ya tenemos una ventaja.

Moderadora

Claro.

Pregunta 3b- ¿Qué desventajas o dificultades le ve a esta actividad? ¿De qué forma se podrían solucionar esas dificultades?

Moderadora

Y, ¿Cuáles son las desventajas o dificultades que le ve a esta actividad?

P#7FB

¿Yo?

Moderadora

Sí, usted lo dijo.

P#7FB

¿La ventaja?

Moderadora

La desventaja.

P#7FB

Pues, si la gente lo hace. Lo que nosotros estamos discutiendo aquí. Las gomas, voltearlas, eliminarlas, los drones mantenerlos tapados. Que no lo hagan…

Moderadora

¿Pero y si la gente no lo hace?

P#5JC

Na…

P#7FB

Ya veremos…

P#6JC

Si la gente no lo hace, es lo que está ocurriendo ahora mismo. Donde vemos tanta basura en cualquier sitio que usted va. Y no son… basura de toda clase. Porque vemos gomas, hemos visto también, inclusive, material reciclaje [reciclable] que lo están desechando también. Y… así que, es buena idea que la gente recoja. Entonces, no podemos dejar a otro. Yo creo que ac…. Más que palabra, uno hacerlo.

P#4EST

Eso mismo.

P#6JC

Y demostrarle y ser consecuente, no cansarse. Porque muchas veces también son los… mismas personas, en las mismas comunidades, haciendo lo mismo, y los demás, pues, no cooperan.

Moderadora

Ok. Y qué podemos… qué solu… ¿qué forma podríamos tener de solucionar el que la gente no lo haga? ¿Cómo podríamos solucionar eso? Que la gente no coopere.

P#5JC

Ayudar a comunicar lo que vemos…

P#3EST

Regar la voz. [dicen muy bajito]

P#2EST

¿Ah?

P#3EST

Regar la voz.

P#5JC

…los que estamos dispuestos a transmitir. En mi caso, es que, no tenemos… nosotros no queremos los escrínes, o sea, no tenemos protección y pues… nos entra brisa y nos trae lo bueno y lo malo, verdad. Así que si nosotros tratamos de mantener nuestro vecindario limpio y evitar que… pues, tengamos la exposición a que crezcan los mosquitos, pues, vamos a tener una comunidad sana.

Moderadora

Usted quería decir algo.

P#4EST

Sí. Yo pienso que podemos crear grupos de apoyo en las mismas comunidades. Este, yo me imagino que todas las comunidades tienen sus directivas y sus grupos. Que pueden ser portavoces para la misma comunidad, de llevar el mensaje y de crear consciencia a los demás. Es como ella dice, la señora, a veces la gente se cansa, y lo hace por un tiempo y deja de hacerlo, pero entonces, hay que volver otra vez, como decimos, el “feedback” otra vez para que ellos vuelvan a reaccionar de la importancia de mantener todo limpio.

P#6JC

En la comunidad de nosotros, pues llegó un momento en que los camiones de basura aumentaron de tamaño y ya por el portón de entrada del condominio se le hace difícil entrar. Así que lo que hicimos es que, en la entrada del condominio en un espacio, hemos comprado cuatro zafacones grandes… pero con sus tapas, tapas que sellan. Ve. Y entonces, pero para que no se los lleven le hemos tenido que poner una cadena con candado, verdad, porque eso era otra cosa, uno lo ponía y se llevaban la basura con todo, verdad. Así que, hemos hecho eso, los rotulamos con, eh… números y unas… este… iniciales que indican que pertenecen al condominio y se lavan todas las semanas. Esos zafacones cuando recogen la basura los viernes; se lavan, cloro, detergente, se guardan, hasta… nuevamente se sacan, o miércoles, o jueves, porque la basura la recogen viernes, y la basura se echa también en bolsas. Ve, en bolsas ahí adentro, cosa que los señores no tienen que coger eso…

Moderadora

Con sus manos…

P#6JC

…porque están con cadena, pero si con bolsas.

Moderadora

Ok.

Pregunta 4- ¿Cuán posible es realizar esta actividad en su comunidad para reducir el número de mosquitos? ¿Por qué?

Moderadora

Ok. Y entonces, ¿Cuán posible es realizar esta actividad en su comunidad para reducir el número de mosquitos? Y, ¿por qué? ¿Es posible hacerlo?

P#5JC

Sí…

Moderadora

¿Es posible que su comunidad lo haga? Número cuatro (*P#4EST*).

P#4EST

Pues, yo pienso que sí. Porque por lo menos en nuestra comunidad existe una directiva que es bien comprometida con todas las actividades que hay para toda la comunidad y yo pienso que esta actividad sería buena para ellos también. Que ellos contribuirían mucho.

Moderadora

Y, ¿número tres (*P#3EST*)?

P#3EST

Amén. [risas]

P#7FB

Ah, quedó corta de palabra.

P#2EST

Pues, como dice ella, cada uno poner de su parte. Bregar, regar la voz, poner algún documento en cada sector, que la gente, por lo menos lo vea, siempre. Y uno mismo pues, si aquel tiene… ‘mira, tienes eso lleno de basura, o lleno de muchas latas, o gomas’, pues uno, pues decírselo para que las mueva de sitio o las deje en algún otro lado, o vaciar las latas o lo que fuera. Por ejemplo, yo, yo tengo gallinas en mi casa, pero yo les cambio el agua, les echo un pocillo de agua para el día nada más.

Moderadora

Exacto.

P#2EST

O sea, hay días que se me olvida echarles, y voy al otro día a echarles agua, porque no quiero dejar agua acumulada.

Moderadora

Exacto. Y entonces, en su comunidad, ¿sería posible hacer esta actividad?

P#8ST

Sería posible porque… pero más bien… yo creo que sería más factible en lo que se pudiera, el visitar casa por casa. Porque…

Moderadora

¿Con cuál sería el propósito?

P#8ST

Yo creo que sería la… les llegaría la información más directa a las personas, porque pues, como usted dice, pues, este, a veces las personas, o no sacan un poquito de tiempo para esas cosas este… prefieren quedarse viendo televisión y esas cosas y no… pues, estamos viviendo de esa manera. Yo entiendo que sería más y llegaría más la información visitándolos, las personas y yo creo que sería más efectivo.

Pregunta 5- ¿Apoya usted esta actividad en su comunidad? ¿Por qué? (*Preguntar a cada participante del grupo de discusión*)

Moderadora

Ok. Y entonces, ¿apoyarían ustedes esta actividad en su comunidad?

P#2EST

Sí.

Moderadora

¿La apoyaría usted?

P#1EST

Seguro.

Moderadora

¿Y usted?

P#2EST

Sí, yo también.

Moderadora

¿Y usted?

P#3EST

[no se escucha respuesta en el audio, pero asiente con su cabeza. Anotadoras presenciales tienen en sus notas que todos los participantes apoyan la actividad.]

Moderadora

¿Y usted, apoyaría?

P#5JC

Sí la apoyaría.

P#4EST

Sí.

P#6JC

La apoyamos y la estamos haciendo.

P#5JC

La estamos haciendo…

Moderadora

Ah ok.

P#6JC

Pero la estamos haciendo.

Moderadora

¿Y usted la apoyaría?

P#7FB

Yo la apoyaría.

Moderadora

¿Y usted?

P#8ST

Es correcto.

Moderadora

¿Por qué? Alguien que quiera decir, ¿por qué?

P#7FB

Es buena, es buena para la salud.

Moderadora

Ok. Muy bien.

Pregunta 5a- ¿Piensa que su comunidad apoyaría esta actividad? Sí, No, ¿Por qué?

Moderadora

Y ¿cómo piensa que su comunidad apoyaría esta actividad? ¿La apoyaría la comunidad?

P#4EST

Por lo menos la de nosotros sí… [dice muy bajito]

P#7FB

Habría que hacer una buena promoción para que entiendan que es por la salud. Y no una promoción para vender insecticidas y artículos necesarios para combatir el mosquito.

Moderadora

Ok. ¿Y ustedes apoyan… creen que su comunidad apoyaría esa actividad?

P#2EST

Sí.

P#1EST

Sí.

P#2EST

Sí.

P#4EST

Una pregunta, esta charla que ustedes nos están brindando en este momento, ¿sería posible llevarlo a la comunidad?

Moderadora

Lo vamos a hacer eventualmente.

Pregunta 6- ¿Qué otra información necesitaría para entender mejor esta actividad?

Moderadora

¿Qué otra información sería necesaria para entender mejor esta actividad? O, ¿ustedes entienden bien esta actividad? ¿Entendieron bien lo que dice ahí?

P#4EST

Sí.

Pregunta 7- ¿Considerarían realizar ustedes mismos esta actividad?

Moderadora

Ok. Ahora les voy a hacer tres preguntitas más. ¿Considerarían realizar ustedes mismos, ustedes, esta actividad en su comunidad?

P#2EST

Sí.

Moderadora

¿Y en sus casas?

P#4EST

Yo soy la primera que lo hago.

Pregunta 8- ¿Qué les haría difícil realizar esta actividad?

Moderadora

Ok. ¿Qué les haría… qué se les haría difícil para realizar esta actividad en su comunidad? ¿Habría algo que les haría difícil?

P#3EST

No, en casa no creo… [lo dice en volumen muy bajito]

P#2EST

No.

P#5JC

Siempre…

P#2EST

Llevar la información y…

P#7FB

Sí… Llevar un boletín casa por casa.

P#2EST

…. y decirle a la gente tú sabes… convencerlos a que esto es una… es algo que es para todos, no para uno…

P#4EST

Un beneficio…

P#3EST

Es un beneficio para la salud.

P#2EST

…. Sino para todos, un beneficio para todos.

Moderadora

Ok, número tres (*P#3EST*) dice, un beneficio para la salud. Que hay que convencer a la gente que es un beneficio para la salud. ¿Y usted?

P#6JC

Hay que ser insistentes. Ve, y no solamente utilizar un método de transmitir esto, verdad.

Moderadora

Ah….

P#6JC

Esto hay que hablarlo, poner rótulos, ponerle facilidades, no hacerle las cosas difíciles porque entonces… sino ponerle facilidades, ve. Y hay oca… y en ese camino, tener en cuenta que, si hay que modificar algo, se puede. Porque no se puede pensar en una sola cosa, de una manera, de esta forma y más nada. Si no, no; no funciona.

Moderadora

No funciona.

Pregunta 9- ¿Hay algo que podría ayudarles a realizar esta actividad de manera más fácil?

Moderadora

Ok, pues usted me acaba de contestar la próxima pregunta, es, ¿Hay algo que podría ayudarles a realizar esta actividad de manera más fácil? Usted dice que hay que promocionarla de diferentes maneras.

P#5JC

Sí…

Moderadora

No siempre de la misma, porque la gente se cansa, que tiene que ser a través de diferentes canales.

P#6JC

Es correcto.

Moderadora

Es lo que yo entiendo. ¿Usted tiene alguna otra opinión?

P#8ST

No.

Moderadora

¿Alguien tiene alguna otra opinión?

P#2EST

No.

Pregunta 9a- ¿Necesitarían más información?

Moderadora

Ok. Entonces, ¿creen que necesitarían más información?

P#5JC

Por lo menos… este, yo entiendo que se está promoviendo, se está anunciando por los canales, por los… por la radio, por… hay difusión. Y la gente está consciente.

P#7FB

Hay que pedirle ayuda a la alcaldesa.

Moderadora

Sí.

P#5JC

Pero hay que estar… hay que estar sobre ellos porque el problema es que si… si se da la información y pasa un lapso de tiempo prolongado, pues la gente se olvida de las cosas.

Moderadora

Exacto.

Pregunta 9b- ¿Necesitarían más adiestramiento?

Moderadora

Ok. ¿Necesita la… necesitaría la gente más adiestramiento? ¿Algún tipo de adiestramiento para hacer esta actividad?

P#7FB

Yo diría que necesitan mucho adiestramiento y aprender sobre…

Moderadora

¿De qué tipo?

P#7FB

Sobre mantener el área limpia y ordenada para que… adiestrarlos o educarlos, mejor dicho.

Moderadora

Educarlos. ¿Usted quería decir algo?

P#6JC

Una de… que yo he podido observar que puede servir, observar en las comunidades, siempre en las comunidades ciertos comercios cercanos, tiene una farmacia, una repostería, que vamos, que vamos. Por ejemplo, le puedo, verdad, sin anuncios y sin nada, aquí la repostería GL. Eso es continuamente mañana día y noche. Ve. Y ahí ellos son muy cooperadores. Y si se habla con los dueños y se prepara algún anuncio, verdad, algo bien preparado…

P#4EST

Un afiche.

P#6JC

Ellos, estoy segura, que en la entrada… porque es que… a ellos les conviene que la gente que entra ahí, entre saludable, no enferma, a comprar, porque ahí venden alimentos y todo. Así que ese es un buen recurso. En estos sitios donde van grandes cantidades de personas, que entran y salen… ahí ponerlo en la puerta que van a empujar para entrar…

P#7FB

Llega mejor el mensaje…

P#6JC

…y lo tienen leyendo continuamente. Yo creo mucho en la repetición. Las cosas se aprenden por repetición.

Moderadora

Claro.

Actividad #2 – Aplicar larvicidas al agua acumulada

Moderadora

Bueno, les voy a mostrar entonces, otra. Son varias, son bastantitas, pero vamos bien, vamos a tiempo. Seguimos. Aplicar larvicidas al agua acumulada. Los larvicidas son pesticidas que se usan para matar las larvas antes de que se conviertan en mosquitos adultos. Ustedes vieron las larvas, verdad…

P#2EST

Ajúm.

P#5JC

Ajúm.

Moderadora

…en el video. Los larvicidas se pueden aplicar en diferentes maneras, en gránulos, en tabletas o líquido. La aplicación de larvicidas puede reducir la cantidad de mosquitos si se aplica correctamente. Los larvicidas no afectan a las personas, ni a sus mascotas, ni el ambiente, si se siguen las instrucciones en la etiqueta. Los larvicidas no deben usarse en el agua potable para consumo humano o animal. Requiere aplicar la cantidad correcta según las instrucciones de la etiqueta. Requiere re-aplicarse cada cierto tiempo. No alcanza… no alcanza lugares que estén ocultos donde los mosquitos se reproducen. ¿Ok? ¿Hay algo que no entendieron ahí, que me quieran preguntar?

P#2EST

Eh… los líquidos, los venden… los líquidos, ¿dónde se consiguen?

Moderadora

Ah… los líquidos… el larvicida en líquido no creo que esté disponible en las tiendas.

P#2EST

O los granos…

Moderadora

Exacto, los granos sí y no quiero dar el anuncio, pero hay una compañía, verdad, que empieza con letra H y tiene un nombre en inglés… ¿Lo digo verdad? “Home Depot”. “Home Depot” los vende y los vende también otros… otras ferreterías lo cual…. también debe haber en “National”.

P#2EST

¿Y cómo se llaman? ¿Así mismo?

P#4EST

Larvicidas.

Moderadora

Larvicidas.

P#2EST

¿Larvicidas?

Moderadora

Sí. Larvicidas. Está a preparación en granos, que es la del pote… galón. Y está la preparación de tabletas o donitas que también se pueden usar… la de líquido, la vamos a ver en el próximo… en el próximo dibujo.

P#2EST

Ok.

[Participante uno (*P#1EST*) y participante dos (*P#2EST*) hablan entre sí, pero no se entiende en el audio qué se dicen]

P#6JC

¿Lo venderían en estas casas agrícolas?

Moderadora

Sí, yo creo que sí, también.

P#6JC

Porque ahí es que casi siempre yo compro…

Moderadora

Sí, ahí también.

P#2EST

Permiso, porque también por eso yo pregunto, porque también vienen unos granitos que son para las hormigas.

Moderadora

Ah sí. Sí. Sí. Sí. Claro.

P#2EST

Y la gente se puede confundir ahí, entre uno u otro.

P#4EST

Tengo una pregunta, este… que extraño, ¿por qué no alcanza los lugares que están… en los lugares ocultos donde están… donde se reproducen en sí…?

Moderadora

Porque la persona no está consciente que tiene un lugar oculto o un criadero, no le va a echar. Por ejemplo, le voy a dar un ejemplo, eh… hace poco fuimos a una casa, verdad, fuimos a una comunidad a hacer la inspección de los patios. Y entonces, la… una persona, verdad, tenía una casita donde tenia utensilios para deshierbar y ese tipo de cosas. Pues nadie pensó que en esa casita habría un criadero de mosquitos porque está…

P#5JC

Techada.

Moderadora

Techada. Pero ¿qué pasa? La casita tiene goteras, verdad, y para eso nosotros estamos muy bien preparados. Y entramos a la casita, miramos el techo, ella es testigo, miramos el techo, verdad, la persona que estaba haciendo la inspección del patio, miró el techo, y dijo, ‘aquí hay filtración’. Y empezó a buscar. Y entonces, ¿qué pasa? Que habían unas botas de trabajo y cuando voltearon las botas de trabajo… ¿qué pasó Karla?

Karla

Muchas larvas, muchas larvas…

Moderadora

Pero muchas, muchas, muchas, muchas.

Karla

Muchas, muchas, muchas.

Moderadora

Esos son sitios que la persona piensa, ‘pues, está techado. ¿Qué va a pasar? No se va a mojar’. Pero sí se moja. Y entonces, esa es la salvedad que se hace, verdad, esa es la salvedad. Si la persona no sabe que tiene un criadero, pues no le va a echar.

Pregunta 2- ¿Es esta una actividad nueva para usted o es algo que ya había escuchado antes? Si la escuchó, ¿dónde la escuchó?

Moderadora

Ok. Ahora les pregunto yo, ¿es esta una actividad nueva para ustedes o es algo que ya habían escuchado antes?

P#1EST

Par mí es nueva. Para mí.

Moderadora

¿Para usted es nueva?

P#1EST

Sí, o sea, estoy hablando es de la reunión. Porque por la radio y el televisor yo siempre he oído.

Moderadora

Ah, usted lo ha oído.

P#1EST

Sí.

Moderadora

Ah ok. Y me dice que lo escuchó, por la radio.

P#1EST

Eso y en la televisión.

Moderadora

Y en la televisión. Ok.

P#6JC

Para mí es totalmente nueva. Y llegar aquí esta noche y ver lo que está ocurriendo, para mí, ni me imaginaba.

Moderadora

Pues muchas de las actividades que les vamos a presentar son cosas que se hacen a través del mundo, verdad, que quizás en Puerto Rico aún no se hagan. Este, por eso se las estamos mostrando, por si alguna vez el gobierno de Puerto Rico decidiera que se van a hacer o que se pueden hacer aquí, tener su opinión.

P#6JC

Es correcto, está bien.

Moderadora

Ese es el propósito de esta reunión. Que… y pensamos que algunas sí se pueden hacer ya, porque se han hecho como la reducción de fuentes de mosquitos, verdad. Y esta ya se ha hecho en Puerto Rico también que ustedes, ve… que no la sepan, pues… porque empezó esta actividad… el Departamento de Salud utilizaba un larvicida que se llamaba abate. Como es un larvicida químico, necesita que un profesional lo aplique y solamente se aplicaba en los cementerios y sitios, ve, donde el Departamento de Salud o los municipios, pues tienen sus técnicos con licencias, pues ellos podían hacerlo y utilizarlos en sitios públicos. Pero, el abate no lo puede utilizar las personas en Puerto Rico, en otros países del mundo sí la gente lo puede usar, pero en Puerto Rico el abate no porque es un químico. Ahora, este otro eh… larvicida que se llama Bti, este si lo puede usar una persona en su casa y se vende, verdad, como dijimos orita, comercialmente. Pero este se introdujo en Puerto Rico con la epidemia del Zika a través de las mujeres embarazadas. Empezamos a orientar las mujeres embarazadas sobre el uso de este larvicida nuevo.

P#5JC

¿Y la fumigación? Que no la he visto…

Moderadora

La fumigación no la incluimos porque ya es común y tradicional en Puerto Rico…

P#4EST

No se está haciendo. No se está haciendo.

Moderadora

…pero ya mismo le llega otro tipo de fumigación.

P#5JC

Sí, no, es que, por ejemplo, a nosotros no nos llega pues…

P#4EST

No se está haciendo.

P#5JC

…como hay portones de…

P#6JC

De control de acceso.

P#5JC

…de control de acceso, pues, lo que llega es muy poco… yo vi que hubo fumigación, porque después de María, esto quedó todo deshecho y con mucho, verdad, este… contenedor y mucha… vertederos por todos lados.

P#4EST

Clandestino…

Moderadora

Sí.

P#5JC

Este… y entonces, fumigaron, vi que fumigaron, pero no llegaron hasta nuestra casa.

Moderadora

Ok. Déjenme que él hable y entonces les…

P#7FB

El larvicida, ¿es venenoso para las personas?

Moderadora

Este larvicida… bueno, fíjese, las instrucciones dicen que incluso se puede usar para agua potable, pero nosotros no lo recomendamos para agua potable. Las instrucciones dicen que se puede usar, pero nosotros no recomendamos para agua potable ni para personas tomar, ni para animales tomar. Ni para poner… si uno se va a bañar, no. Es para agua que usted guarde como para echarle a las plantas, para bajar los baños, para mapear, ese tipo de agua que uno guarda, ¿no? Que la gente guarda.

P#7FB

Pero… ¿el que hace el larvicida lo recomienda para beber?

Moderadora

Sí porque se ha probado en otros países… lo que pasa es que nosotros no lo recomendamos porque el puertorriqueño específicamente, usted le dice, échele una tapita y el puertorriqueño dice, espérate, para que funcione mejor voy a echar dos.

P#5JC

Voy a echarle… [risas]

P#4EST

Es cierto.

Moderadora

O le voy a echar la mitad. No le voy a echar la tapita completa, le voy a echar la mitad. Y entonces, ya ahí, usted está cambiando las instrucciones del paquete, ve. Por eso hacemos énfasis en las instrucciones de la etiqueta. Pero el puertorriqueño, espérate si una tapita sirve, vamos a echarle tres para que sirva más.

P#7FB

Que ya ahí se vuelve tóxico…

P#5JC

Ahí se vuelve tóxico.

Moderadora

Ahí se puede volver tóxico.

P#6JC

Pues, precisamente usted me respondió a lo que yo iba a decir. Que toda esta información ayuda a nosotros, verdad, eh… a nutrirnos, a enseñarnos, y a ser disciplinados. Porque es la cultura…

P#4EST

Eso es así.

P#6JC

… eso de siempre estar en contra de la corriente. [risas] Si me dicen, no vayas para la izquierda… ah, no, voy para la izquierda, porque es para yo llegar más temprano.

Moderadora

Ajá. Exacto.

P#7FB

Y no llegas…

P#6JC

Este… y entonces, así lo aplicamos a todo. Inclusive, usted ve hasta en los medicamentos que ingerimos, a veces son… arriesgarse a las personas. Ellos automedicamen… o sea, se automedican de una manera…

Moderadora

Exactamente.

P#6JC

Y entonces, después vienen las situaciones.

Moderadora

Las situaciones. Bueno…

P#7FB

No, y a veces vienen medicamentos que… estamos desviándonos un poquito del tema…

Moderadora

Sí. [risas]

P#6JC

Pero es eso.

P#7FB

Pero, a veces vienen medicamentos que te curan una enfermedad, pero te dañan cuatro.

Moderadora

Ajá, te dan otras, verdad que sí.

P#7FB

Como dice la canción de Héctor Lavoe, ‘la cura es peor que la enfermedad…’. [risas]

Moderadora

Exacto.

Pregunta 3- ¿Cree que esta actividad reduciría el número de mosquitos en su comunidad? ¿Por qué?

[no se hizo]

Pregunta 3a- ¿Qué beneficios o ventajas tiene esta actividad para usted?

Moderadora

Ok. Bueno. ¿Qué beneficios o ventajas tiene esta actividad para ustedes? ¿Tiene algún beneficio o ventaja para usted? Número ocho (*P#8ST*).

P#8ST

Sí, claro.

Moderadora

¿Qué…?

P#8ST

Hay beneficio, este… que uno aprende más y puede pasar la información a las demás personas.

Moderadora

Ok. Pero, la actividad como tal, el uso del larvicida como tal.

P#8ST

Ah, sobre el larvicida, pues, claro, yo me imagino pues, que… claro para… usarlo para matar los larvicidas. Y siempre, las personas, pues tienen en las casas los… cómo se dice, los recipientes…

Moderadora

Recipientes. Con agua, ¿verdad? Ok. Alguien tiene… ajá.

P#5JC

En el caso… doy fe, porque mi esposa es bien… eh… puede encontrar todo los fumigadores, puede encontrar de todo y ella lo usa. Y yo le ayudo porque si no, no funciona. [risas] Así que el aprender nos ayuda a mejorar…

Moderadora

Sí, claro. Pero yo estoy pre… cuando yo diga actividad me estoy refiriendo a la…

P#4EST

Larvicida.

Moderadora

…al método como tal. Correcto.

P#6JC

A eso iba…

*****Participante número 9 llega al grupo de discusión*****

P#6JC

A eso iba, el método que usted está usando, me está enriqueciendo a mí en conocimiento.

P#5JC

Exacto.

P#6JC

Así que, para mí, ha sido excelente.

Moderadora

Ok.

P#4EST

A mí me ha creado consciencia para las próximas situaciones que haya de escasez de agua que uno almacena… y por lo menos yo le echo cloro, es lo que le echo…

P#6JC

Sí.

P#4EST

Y mira, uno se puede tener un larvicida y con tiempo se lo echa y ya va a estar más tranquilo.

Pregunta 3b- ¿Qué desventajas o dificultades le ve a esta actividad? ¿De qué forma se podrían solucionar esas dificultades?

Moderadora

Ok. ¿Qué desventajas o dificultades le ve usted al uso de larvicida? ¿Le ve alguna desventaja?

P#7FB

Yo no le veo ninguna, al revés, veo que ayudaría bastante y no sabía que eso lo vendían.

Moderadora

Ok.

P#6JC

Bueno, hay que tener la precaución de tenerlo en un sitio rotulado y apartado, verdad, no se puede… no tener ahí a la mano, porque eso es un veneno prácticamente.

Moderadora

Exactamente. Exactamente. Y, ¿De qué forma se podría solucionar esa desventaja?

P#6JC

Bueno, pues almacenándolo en un sitio seguro bajo llave, en un sitio rotulado, no al alcance donde hay niños o personas, que no… por ejemplo, en nuestro hogar así yo lo hago. Yo tengo un closet más que para eso en el sótano abajo, que tiene hasta llave. Ve. Todo eso está rotulado y aparte.

Pregunta 4- ¿Cuán posible es realizar esta actividad en su comunidad para reducir el número de mosquitos? ¿Por qué?

Moderadora

¿Cuán posible sería realizar esta actividad en su comunidad?

P#5JC

¿Este tipo de…?

Moderadora

El uso de larvicida.

P#5JC

Ah, el uso de larvicida.

Moderadora

¿Cuán posible es realizar el uso de larvicida en su comunidad? ¿Usted cree que la gente lo haría? ¿Usted lo haría?

P#2EST

Yo lo haría.

P#7FB

Yo lo haría también.

Moderadora

¿Usted lo regaría por su patio? ¿Usted cree que su… que en su comunidad se podría hacer? Si se fuera un personal diciendo, ‘mire, este… déjeme entrar a su patio…’ ¿Lo harían?

P#4EST

Pienso que sí.

P#2EST

Sí.

P#7FB

Yo creo que sí. En la comunidad mía son personas que… educadas.

Pregunta 5- ¿Apoya usted esta actividad en su comunidad? ¿Por qué? (*Preguntar a cada participante del grupo de discusión*)

Moderadora

Ok. Entonces, ¿apoyaría usted esta actividad?

P#2EST

Sí.

Moderadora

Piensa que su… a cada uno le tengo que preguntar. ¿Usted lo apoyaría?

P#1EST

Siempre y cuando la persona vaya identificada.

Moderadora

Ah, o sea, que, si va una persona fuera, tiene que identificarse. ¿Pero y si se le da el larvicida a usted? ¿Usted lo aplicaría en su patio?

P#1EST

Seguro que sí.

Moderadora

¿Y usted?

P#2EST

Yo también.

Moderadora

¿Y usted?

P#3EST

Sí.

Moderadora

¿Y usted?

P#4EST

También. Sí.

P#5JC

Sí.

P#6JC

[Asiente con la cabeza; Anotadora presencial tiene en sus notas que todos apoyaron la actividad.]

Moderadora

Sí.

P#7FB

Sí.

P#8ST

[Asiente con la cabeza; Anotadora presencial tiene en sus notas que todos apoyaron la actividad.]

Moderadora

Sí. Y sí. [haciendo referencia a participantes 7 (*P#7FB*) y 8 (*P#8ST*)] Ok. Ahorita cuando yo les… cuando vayamos a la próxima, pues ahí lo incluimos a usted [dirigiéndose a participante 9 (*P#9GL*).

P#9GL

[da su excusa del por qué llegó tarde]

Moderadora

No se preocupe, que le agradecemos que esté aquí. Su esfuerzo se lo agradecemos.

P#9GL

Gracias.

Pregunta 5a- ¿Piensa que su comunidad apoyaría esta actividad? Sí, No, ¿Por qué?

Moderadora

¿Y piensa que su comunidad apoyaría el uso de larvicidas?

P#7FB

Sí.

P#5JC

Sí.

P#2EST

Sí.

P#6JC

Sí.

Pregunta 6- ¿Qué otra información necesitaría para entender mejor esta actividad?

Moderadora

Ok. ¿Qué otra información necesitaría para entender mejor el uso de larvicidas?

P#2EST

Bueno, saberlo explicar para que las personas puedan usarlo como se dijo orita, para que no lo usen de manera…

Moderadora

Incorrecta.

P#2EST

… incorrecta. Y funcione. Y funcione.

Moderadora

Ok.

P#9GL

¿Qué es larvicida [pero lo dice mal]?

P#8ST

Larvicida.

Moderadora

Le voy a leer. Larvicida.

P#8ST

Sí, es como llegaste ahora…

Moderadora

Los larvicidas son insecticidas… ah, el mosquito hembra pone huevos en los envases. Cuando el huevo… cuando… en las paredes del envase, cuando ese envase se llena de agua, esos huevitos se rompen y nacen las larvas que son esos animalitos que usted ve. Los gusanitos. Esas larvas luego de cinco días aproximadamente se convierten en pupas. Y luego a los dos o tres días se convierten en mosquitos voladores o mosquitos adultos. Pues este insecticida mata… este larvicida mata las larvas, cuando el mosquito está en su fase de larva.

P#9GL

¿De reproducción?

Moderadora

Exacto. Y esto se puede aplicar en gránulos, como ve, el señor está echándoselo al pozo séptico. Ve, o a la alcantarilla. O se puede aplicar en forma de donitas, que se usan ya cuando son envases bien grandes, como este…

P#9GL

Con el pozo séptico.

Moderadora

También el pozo séptico, o se puede echar en las fuentes agua, si se pican, se pueden echar en las fuentes de agua. Y también vienen en líquido. Que lo voy a presentar ya mismo. Y entonces, si se aplica correctamente, no afecta a las personas, ni a las mascotas, ni al ambiente. Ahora, si se aplica incorrectamente, verdad, sabe que el puertorriqueño…

P#9GL

Acá viene la duda mía.

Moderadora

Ok.

P#9GL

Como todos sabemos que sí, pues yo también entiendo que sí.

Moderadora

Ok. Y entonces, no se pueden usar en agua potable. Nosotros recomendamos que no se use en agua potable ni para personas, ni para animales. Sino que lo usen si mapea, para el baño. Si tiene un baño que no usa, que esos son los que tienen mosquitos, pues usted se lo echa al agua de inodoro, para que los mosquitos no vayan ahí, o al agua… ¿cómo es que se llama?

P#9GL

Almacenada.

Moderadora

Exacto, cuando usted tiene agua almacenada que no quiere botar, usted la usa para alguna cosa que no sea para beber ni para bañarse, usted se la puede echar.

Pregunta 7- ¿Considerarían realizar ustedes mismos esta actividad?

Moderadora

Entonces, este… les pregunto. ¿Considerarían ustedes… ya les dije verdad, considerarían realizar esta actividad ustedes mismos?

Sue

No, no se preguntó. La siete no se preguntó.

Moderadora

Sí, pero, la contestaron de otra manera. Fue que la contestaron de otra manera, porque yo pregunté si apoyarían…

Pregunta 8- ¿Qué les haría difícil realizar esta actividad?

Moderadora

Y entonces, ¿qué les haría difícil realizar esta…usar los larvicidas? ¿Qué podría ser algo que le haría difícil a ustedes usar el larvicida?

P#4EST

¿Usarlo nosotros o…?

Moderadora

Usarlos ustedes.

P#9GL

¿Cómo conseguirlo?

Moderadora

Cómo conseguirlo sería algo que lo haría difícil.

P#6JC

Este, después de que se me den las instrucciones, que no sean difíciles…

Moderadora

Ah, que las instrucciones sean bien difíciles.

P#6JC

Si las instrucciones son difíciles, pues ahí está el grado de dificultad, pero si no son difíciles, pues uno las puede… por eso es importante leer las instrucciones.

Moderadora

Y entonces, cómo conseguirlo para usted sería…

P#9GL

Si porque este…

P#7FB

El costo. ¿Económico o es caro?

Moderadora

Ah, el costo. El costo sería otra cosa difícil. El número dos (*P#2EST*) dice lo mismo.

P#2EST

Eso es lo que yo iba a decir, exacto. Costo.

P#5JC

Exacto.

Pregunta 9- ¿Hay algo que podría ayudarles a realizar esta actividad de manera más fácil?

Moderadora

Ok, entonces. ¿Hay algo que les ayudaría a usar los larvicidas?

P#6JC

Sí, que en la época cuando se… en esta época, cuando empiezan a llegar tanto los mosquitos y empiezan a caer esos aguaceros, que más o menos en algún momento, en algún charco se aguanta o algo surge y si es corridito, pues, hay que tenerlo, porque es mejor prevenir.

Moderadora

¿Pero usted entiende que necesitaría más información para usar el larvicida?

P#7FB

Una preguntita, los insecticidas tales como las marcas Real Kill y las demás marcas, ¿matan las larvas o…?

Todos

No, no, no.

P#6JC

Eso no.

Moderadora

Mosquito adulto.

P#9GL

Los mata cuando están volando.

Moderadora

Mosquito adulto. No matan las larvas.

P#9GL

Y los matan bien muertos, dice la lata. [risas]

P#7FB

Algunos tú los tienes que bañar…

P#5JC

Yo fumigo, constantemente el área donde resido, porque yo soy bien propenso a que me piquen.

P#9GL

Yo tengo una preocupación con una vecina que está aquí al frente. Perdonando que señale para allá… [risas]

P#7FB

Pero no digas el nombre.

Moderadora

Detrás, detrás de la pared.

P#9GL

Este… la hierba de ella está más alta que la de los pastos. Entonces la vecina mía, que es la que está más… está la calle de ella, y la calle es así, la casa mía. Pero como el pájaro vuelo. Yo le pregunté a ella, que por qué ella no tenía bien cuidado su patio. Ella dijo que porque no tenía chavos. Una casa que es heredá [heredada], es heredera, no paga renta. Entonces, pues me contestó con esa manera. A mí me chocó porque [no se entiende lo que dice] a veinte o treinta pesos que le cobre, por el frente, un muchacho, creo que ahí [no se entiende lo que dice]. Eso, lo que la señora habló casi ahora, eso es un foco de… ayudar a que nuestros ambientes que están limpios, ese nos desayuda.

Moderadora

Claro. Y entonces…

P#9GL

¿Cómo podemos bregar para entrarle a bofetá [bofetada] a los mosquitos de alguien? [risas]

P#7FB

¿En la hierba se puede procrear el mosquito?

P#8ST

Es a ella.

Moderadora

Es a los mosquitos no a ella. [hacen un poco más de chiste]

Pregunta 9a- ¿Necesitarían más información?

[se contestó en otro lado]

Pregunta 9b- ¿Necesitarían más adiestramiento?

Moderadora

¿Creen que para usar el larvicida necesitan algún adiestramiento?

P#6JC

No. Yo no.

Moderadora

¿No?

P#7FB

Debería, debería haber para precaución.

P#9GL

Con lo que pidió la señora… con la orientación escrita [instrucciones]…

P#6JC

Claro.

P#9GL

…sencillas, uno puede entenderlas.

Actividad #3 – Rociar larvicida desde un camión

Moderadora

Ok. Bueno, vamos para el otro. Aquí se unen las dos, es rociar larvicida desde un camión. Ustedes están bien relacionados con la fumigación desde un camión, porque toda la vida lo han visto…

P#7FB

Eso se lo lleva el viento.

Moderadora

…pues en vez de insecticida rociarían larvicida. Se acuerdan que, yo les dije que habían unos gránulos, unas tabletas y uno líquido. Pues esta es la forma en que se usa líquido. Y los larvicidas se pueden aplicar de diferentes maneras. Pero muchos programas de control de mosquitos han encontrado, que aplicar larvicida desde un camión puede ser efectivo para alcanzar los lugares donde se encuentran las larvas. El larvicida se rocía desde un camión sobre edificios, la vegetación, en propiedades, terrenos, etc. Los larvicidas tienen que re-aplicarse regularmente. ¿Por qué? Porque tienen un tiempo de duración. Hay algunos que dicen que duran dos semanas. Otros duran dos meses, otros duran un mes.

Pregunta 2- ¿Es esta una actividad nueva para usted o es algo que ya había escuchado antes? Si la escuchó, ¿dónde la escuchó?

Moderadora

Y les pregunto, ¿habían escuchado el método de rociar insecticidas anteriormente?

P#9GL

¿Así en el camión? Por aquí ha venido, pero pasan como si se les estuviera huyendo un disco, porque van a las millas.

Moderadora

Pero es insecticida, no es larvicida.

P#5JC

No es larvicida…

P#9GL

Ah bueno, desconozco lo que estén echando…

Moderadora

¿No lo habían escuchado verdad?

P#5JC

No.

P#9GL

No.

Moderadora

Porque nunca se ha hecho en Puerto Rico. Se ha rociado insecticida, pero no se ha fumigado nunca con larvicida.

P#9GL

Ah, porque por aquí pasa una guagua, pero es como té helado, cuando usted se mete se ahoga la peseta… [¿?] [risas]

P#8ST

Pasa tan rápido…

P#9GL

Sí y no mata.

P#6JC

De hecho, es la primera vez que yo oí la palabra larvicida. Para mí esa es nueva, ve.

P#9GL

Yo también.

P#6JC

Así que de ahí partimos.

Moderadora

Ok. ¿Nadie la había usado… nadie la había escuchado antes?

Todos

No.

Moderadora

Claro porque…

P#6JC

Ni tenía idea de que eso lo vendían.

Pregunta 3- ¿Cree que esta actividad reduciría el número de mosquitos en su comunidad? ¿Por qué?

Moderadora

Cree que… ¿cree usted que rociar larvicidas, fumigar larvicidas, reduciría el número de mosquitos en su comunidad?

P#7FB

Yo creo que no, porque el viento se lo llevaría.

Moderadora

El viento se lo llevaría.

P#7FB

Se lo llevaría y no caería exactamente en los sitios donde se necesita.

P#5JC

Es que cae… cae en todos lados, el problema es que hay áreas que tú no sabes si tienen… y no todo el mundo va a estar buscando los contenedores, verdad.

P#7FB

Eso es personal, eso lo tiene uno en la casa…

P#5JC

Y eso entra por todos lados y no escoge.

P#7FB

Y cae donde no hay…

P#5JC

Yo entiendo que sí, que ayudaría.

Moderadora

Usted entiende que ayudaría.

P#5JC

Claro.

Moderadora

Él dice que le ve su li… que no ayudaría porque no alcanzaría todos los lugares.

P#7FB

Seguro, donde se necesita más, no va a caer. Porque es rociado a…. [no se entiende la palabra]

P#9GL

¿Ese no se ha usado todavía?

P#5JC

No.

P#6JC

No…

Moderadora

No aquí en Puerto Rico.

P#9GL

Por eso, pues no sab… yo no contestaría, porque si no se ha usado, no sabemos la reacción… verdad, porque el agente Naranja… el agente Naranja los que fueron al ejército, yo no fui, pero, tengo familiares que murieron a consecuencia del agente Naranja, que aquí se hicieron unas pruebas en Puerto Rico, eso lo tiraron y después… al cabo de diez o veinte años es que han dicho que eso estaba mal, y eso mata la gente. No vaya a ser que sea una cosa para los mosquitos…

P#7FB

Y nos haga más daño a nosotros…

P#9GL

No, y le afecte la… porque cuando pase, el que pasa por casa, me ha pasado, se mete dentro de la casa, pero yo siempre el efecto rápido lo… porque uno lo inhala… y entonces, pues, lo pasan tan ligero que se cae, no se queda en el ambiente.

P#7FB

No hace nada, el viento le da….

P#9GL

Mira, aquí vino una vez… aquí vino un avión, yo llevo aquí… yo soy de aquí de GL, yo llevo aquí 55 años. Cuando pasó David y Federico, yo estaba deshierbando el patio, eran como las dos de la tarde. Y pasó un avión que era de Venezuela, el avión, de cuatro motores. Pasó, pero bien bajito, de allá hasta la Playa. Desde yo… lo miré. Se perdió por… bueno, me trepé encima de la casa. Y allá viró y volvió para acá, tirando pa’ todo ese chorro de… no diría de humo, de gas. Eso más nunca lo vi yo. Y a mí me causó tanta sensación ver ese [no se entiende la palabra] sin haberlo anunciado. Y después uno tanta cosa que hay…

Moderadora

¿Qué año sería eso? ¿86?

P#9GL

Fue cuando pasó David y Federico…

Moderadora

¿86? ¿87?

P#6JC

David y Federico fue… como, el 79 para el 80, 81, 82.

P#9GL

Por ahí, por ahí. En un mes corrido.

P#6JC

Sí así mismo fue, en una temporada.

Moderadora

¿Usted quería decir algo?

P#6JC

Siguiendo la línea de, que si fumigan, que si no fumigan cuan efectivo. Primero que tienen que empezar ellos anunciando un programa donde oriente las personas. Y cuando vaya a pasar el camión, ya las personas que tengan en los patios… que ya hayan botado el agua, que hayan reducido las probabilidades. Cosa que una cosa completa la otra. Pero si nadie se entera, y no anuncian y no educan pues es inefectivo.

Moderadora

Ok. ¿Cree que esta actividad reduciría el número de mosquitos?

Pregunta 3a- ¿Qué beneficios o ventajas tiene esta actividad para usted?

Moderadora

¿Qué beneficios o ventajas tiene esta actividad… esta actividad, cuando digo actividad es el … la fumigación con larvicida, tiene algún beneficio para ustedes? Los de este lado que están tan calladitos hace rato.

P#2EST

Yo entiendo que sí.

Moderadora

Entiende que sí. ¿Por qué? ¿Qué beneficio tiene?

P#2EST

Pues porque, aunque no lo hemos probado, pero… si se ha probado en otros países y ha funcionado, pues aquí debe funcionar también.

P#9GL

No, pero eso… usted no sabe que en otros países… ¿ha sido ha aceptado?

Moderadora

Sí.

P#9GL

Ah, por eso, pues… para mí es nuevo y yo no…

Pregunta 3b- ¿Qué desventajas o dificultades le ve a esta actividad? ¿De qué forma se podrían solucionar esas dificultades?

Moderadora

Ok. ¿Qué ventajas o dificultades ustedes le ven a esta actividad?

P#5JC

¿A la fumigación?

Moderadora

Con larvicida.

P#9GL

Pues, falta de información.

Moderadora

Falta de información. Pero como actividad como tal. Yo creo que él [participante 7 (*P#7FB*)] dijo algo, que no necesariamente llegaría a todos los lugares. Él dijo…

P#7FB

Es que, el viento se lo va a llevar…

P#4EST

Pero fíjate, partiendo de lo que él dice, que no llegaría a todos los lugares, yo pienso como la señora. Si hay una coordinación acertada entre el camión y los residentes, pues el camión hace su parte y el residente, pienso yo, el ciudadano en su casa, completa lo que falte. Y habría una coordinación y se haría completo.

P#7FB

Lo mejor sería, permiso, lo mejor sería que si lo podemos comprar. Pues cada cual, en su casa, se encargue de combatir el mosquito. Si se logra conseguir económico…

Moderadora

O sea, que a usted le gustaron más las otras preparaciones, los granos y las tabletas.

P#7FB

Seguro para yo…

Moderadora

Y que cada quien lo haga en su casa.

P#7FB

Seguro. Porque esperar un camión que no sabe cuándo va a pasar…

P#4EST

No, pero si existe la coordinación…

P#9GL

Que esté uno.

P#7FB

Que esté uno y, ‘ah, va a pasar…’ ¿Cuándo? ¿Cuánto hace que no… la alcaldesa no riega el insecticida? ¿Cuánto hace? Por mi comunidad, ¿Cuánto hace? Cuatro, cinco meses.

Moderadora

Ok, y entonces, ustedes… ¿usted quería decir algo? Antes que se me olvide.

P#5JC

Sí, yo entiendo que es algo que complementa las cosas que nosotros podemos hacer. La gente, no todos están aquí, este… porque yo entiendo que se citaron mas personas. Los puertorriqueños no somos muy dados a ni siquiera a venir a preocuparnos para aprender de las cosas que nos pueden perjudicar, cómo ayudarnos, verdad. Pues eso suplementaria… los que no saben, pues mire cojan su mojadita en los patios etc., con esta fumigación.

Moderadora

¿Usted quería decir algo?

P#9GL

Sí, aquí hay un complemento que, ve… a él lo conozco, a él lo conozco, los demás no sé de qué comunidades son; a él lo conozco, de aquí de ST. Pero aquí hay que hacer un censo en GL porque aquí hay mas de 50 casas, más, que están cerradas por años. Que no hay preocupación de patios de casas cerradas que no se sabe que hay dentro de la casa, este… vehículos en las marquesinas, que el vehículo tiene asientos, e inP#9GL también, porque el mosquito se mete por cualquier rotito.

Moderadora

Y entonces, habiendo ese problema de las casas abandonadas, ¿usted le ve un beneficio a esto, o no? ¿A rociar?

P#5JC

Sí.

P#9GL

Bueno, yo le veo beneficio…

Moderadora

Con larvicida.

P#5JC

Sí claro. A eso es a lo que yo me refiero.

P#9GL

Yo le veo beneficio…pero, lo que él dice, lo que él estaba diciendo, que somos ir… yo soy el 9, vamos a poner que habemos 10 o 11 personas aquí…

Moderadora

Nueve… nueve…[risas]

P#9GL

Este…Se pudiera dar dos… una o dos o tres charlas más que haya más gente, más complemento para más intercambio. Porque a lo mejor en la de él, con un balde haciendo la mezcla, y el de al lado se cree que le está haciendo el pitorro. [risas] No, no, vamos a ver la gente. ‘Ah, este está loco, mira lo que está haciendo, o sea…’ Porque el vecino no sabe que está haciendo un beneficio para él también. Y la orientación que sea más amplia.

Moderadora

Más amplia, ok.

Pregunta 4- ¿Cuán posible es realizar esta actividad en su comunidad para reducir el número de mosquitos? ¿Por qué?

Moderadora

¿Cuán posible es realizar esta actividad en su comunidad? O sea, ¿sería posible realizar esta… rociar larvicida en su comunidad?

P#5JC

Después que se…

P#6JC

Yo creo que sí. Y atando un poquito lo anterior con esta. Mire, cada uno, verdad, es diferente, como usted dice, estamos hablando a base de opiniones. Yo desde pequeña me crie en bases militares. Y eso yo, desde los tres o cuatro años lo veía y anunciaban.

P#9GL

Sí. Es más común.

P#6JC

Inclusive cuando iban por las calles se les permitía a las personas, que tenían escrin y todo, abrir las ventanas y las puertas, se acercaba un poquito el camión o se quedaba al frente, cosa de que esa… esa casa. Ellos le llamaban…

P#5JC

Barracas.

P#9GL

Barracas.

Moderadora

Bunkers.

P#6JC

Este… no, a las casas que viven los residentes, ellos le llaman como planteles, algo así, ve. Y entonces, pues nada, y usualmente la calle entraban, tenían un redondel, y eso iban… y cuando la casa quería, con solamente el camión ver que abría las puertas de escrín, él se detenía al frente. Y ahí, eso, se quedaba más rato. Ve. Y yo vi eso y eso era efectivo porque la base donde yo vivía todo alrededor era cañaverales. Ve, así que eso era efectivo.

P#5JC

En el caso nuestro que hay dos controles de acceso, pues tendríamos que saber con anticipación para darles la oportunidad que entre y fumigue. Porque el problema es que, si fumigan acá en GL, allá arriba no va a llegar.

Moderadora

Claro. Entonces, creen ustedes que es posible hacerlo en sus comunidades.}

Todos

[nadie en el audio dijo que no se pudiera]

P#6JC

Sí claro.

P#5JC

Sí.

P#9GL

Sí se puede, se puede.

Pregunta 5- ¿Apoya usted esta actividad en su comunidad? ¿Por qué? (*Preguntar a cada participante del grupo de discusión*)

Moderadora

Ok. Y entonces, ¿apoyarían ustedes, el rociamiento… o la fumigación de larvicidas en su comunidad?

P#5JC

De larvicidas. Sí.

P#6JC

Claro que sí.

P#4EST

Sí.

P#2EST

Sí.

P#1EST

[Participante asiente con la cabeza. Anotadoras tienen en sus notas que todos los participantes apoyan la actividad.]

P#3EST

[Participante asiente con la cabeza. Anotadoras tienen en sus notas que todos los participantes apoyan la actividad.]

P#7FB

[Participante asiente con la cabeza. Anotadoras tienen en sus notas que todos los participantes apoyan la actividad.]

P#9GL

[Participante asiente con la cabeza. Anotadoras tienen en sus notas que todos los participantes apoyan la actividad.]

P#8ST

[Participante asiente con la cabeza. Anotadoras tienen en sus notas que todos los participantes apoyan la actividad.]

Moderadora

Todos lo apoyarían.

P#9GL

Sí.

P#7FB

Si lo hicieran como ella dice, que ellos iban casa por casa, y no forzados así…

P#6JC

Iban despacito.

P#7FB

Ajá… no como aquí, porque… a las millas que va el camión cuando tira el insecticida.

P#1EST

Mira, párate…

Pregunta 5a- ¿Piensa que su comunidad apoyaría esta actividad? Sí, No, ¿Por qué?

Moderadora

Ok. Y entonces. ¿Piensa que su comunidad apoyaría esta actividad?

P#5JC

Sí.

P#2EST

Sí.

P#1EST

Sí.

P#7FB

Sí, yo pienso que sí. Debe apoyarlo, porque es para la salud de las personas.

Pregunta 6- ¿Qué otra información necesitaría para entender mejor esta actividad?

Moderadora

Ok. ¿Qué otra información necesitaría para entender mejor esta actividad? [participante 9 (*P#9GL*) se retira un momento a contestar una llamada telefónica] ¿Qué otra información necesitaría para entender mejor esta actividad? O, ¿está bien la información así?

P#5JC

Ajúm.

P#1EST

Está bien la información.

P#6JC

¿Estamos con lo del camión?

Moderadora

Sí.

P#6JC

Exacto, yo creo que está bien.

Actividad #4 – Fumigación dentro de las casas con insecticida de acción residual

Moderadora

Ok. Pues vamos para la próxima, que ya vamos por la mitad. [hacen chiste de no perderse la novela] Fumigación…. Fumigación dentro de las casas con insecticida de acción residual. La fumigación dentro de las casas con insecticida de acción residual, es un método de control de mosquitos donde un profesional adiestrado trata el interior de su hogar con un insecticida. Consiste en rociar las paredes y otras superficies de una casa con un insecticida que continúa funcionando varios meses. Mata los mosquitos que se posen en superficies que han sido rociadas con el insecticida. Este tipo de fumigación se ha usado en muchos países del mundo incluyendo Puerto Rico durante la epidemia del Zika y Estado Unidos. Puede Ser efectivo en reducir la cantidad de mosquitos si se aplica en una gran cantidad de casas en un área. O sea que si son poquitas casas en un área…

P#4EST

No se nota…

Moderadora

…tiene que ser en muchas casas en un área. Después de aplicarlo, puede que haya olor por unas horas, pero es poco probable que cause daño a las personas cuando se hace correctamente. Requiere del permiso y la disponibilidad del residente para entrar a la casa a fumigar. El uso repetido, a través del tiempo, puede hacer que los mosquitos puedan ser resistentes a los insecticidas.

P#1EST

Se convierten inmune…

P#4EST

Mira eso…

P#6JC

Sí, eso es así.

P#7FB

Ese animal es un monstruo…

Moderadora

Esta fumigación debe repetirse para mantener baja las poblaciones de mosquitos. Orita alguien estaba diciendo que tenia que echarle muuucho insecticida. Mucho Raid verdad, a los mosquitos, para que se murieran.

P#5JC

Sí.

P#6JC

Sí.

Moderadora

Eso es porque los mosquitos están causando resistencia porque se ha usado tanto…

P#6JC

Claro. Como las bacterias.

Pregunta 2- ¿Es esta una actividad nueva para usted o es algo que ya había escuchado antes? Si la escuchó, ¿dónde la escuchó?

Moderadora

Ok. ¿Han escuchado sobre el uso de este insecticida de acción residual? ¿Habían escuchado de eso anteriormente?

P#5JC

Sí.

P#4EST

Cuando va el fumigador…

Moderadora

Ella no. Ella dice que no. La dos [realmente la 3 (*P#3EST*)] no.

P#9GL

Y yo tampoco.

P#4EST

Eso es cuando va el fumigador profesional a la casa, ¿no?

Moderadora

Pues, sí y no. Es… se parece, pero el exterminador, solamente rocía por el piso, si usted lo ve, por las rendijas del piso. Pero esto es paredes de arriba abajo, paredes y todo. Ve, que lo dice ahí, dice, consiste en rociar las paredes y otras superficies de una casa con un insecticida que continúa funcionando por varios meses. Ve aquí… ¿qué usted ve aquí? Está fumigando como el exterminador, verdad. El piso y la superficie. ¿Qué es esto?

P#9GL

Un “hamper”.

Moderadora

Un “hamper”. ¿Y esto?

P#4EST

La nevera.

P#9GL

Una nevera.

P#7FB

Nevera.

Moderadora

Es un librero.

P#6JC

Una biblioteca…Un librero ajá.

Moderadora

¿Y aquí qué hace? ¿Dónde está fumigando?

P#6JC

En la puerta.

P#2EST

Las paredes…

P#9GL

Una puerta.

P#6JC

En la pared.

Moderadora

En la pared de la puerta. ¿Y aquí?

P#6JC

Detrás del sofá.

Moderadora

Detrás del sofá. O sea, que se fumiga en todos lados. Todas las paredes, en todo, todo, piso, todo.

P#7FB

¿Y hay diferencia entre ese y el insecticida?

Moderadora

Bueno…

P#4EST

Pregunto. Si esa es una fumigación que, como dice la literatura, tiene que ser un profesional adiestrado, cómo nosotros en nuestras casas, si no es un exterminador, ¿cómo podemos conseguir eso?

Moderadora

No lo puede hacer. No lo puede hacer. Tiene que venir un… si… si…. Digamos, si usted decide que quiere ese tipo de fumigación y es una alternativa que el gobierno de Puerto Rico está ofreciendo, como pasó para la epidemia del Zika, pues entonces, usted pide el servicio, y van a su casa y se lo hacen. Por ejemplo, durante la epidemia del Zika, las mujeres embarazadas se les ofreció ese servicio. Algunas dijeron que sí, otras dijeron que no. Pues las que dijeron sí, se les fumigó su casa de esa manera.

P#7FB

¿Y tiene algún…?

Moderadora

¿Perdón?

P#7FB

¿Tiene algún costo?

Moderadora

En aquel momento no tenía costo.

P#7FB

¿Y ahora…?

Moderadora

Pero siempre hubo un aprovechado, verdad. Pero en aquel momento no tenía costo.

P#7FB

¿Y ahora? No sabes…

P#6JC

Depende...

Moderadora

Y ahora, todo depende de cómo se vaya a aplicar.

P#9GL

Si se empalman las paredes y un niño, un bebé, que gatea, lo toca y ve que el nene está metiéndose los dedos, ¿le puede hacer daño?

Moderadora

Bueno, yo entiendo que cuando se rocía dentro de la casa hay que salirse de la casa por varias horas. Ahora… a lo que…

P#1EST

Penetra.

Moderadora

Penetra. Sí.

P#9GL

Porque se da el caso que uno está… yo estoy en casa ahora, y me fumigan. Pero si salgo y al ratito viene la hija mía con las nietas, porque ya la hija mía dejó de… ya está grande. Pero los nietos míos tienen ya… tengo bisnietos que están…

Moderadora

Ajá, chiquitos.

P#9GL

Entonces, como no saben si eso se hizo a las diez de la mañana que vayan a entrar y los nenes se pegan y al ratito están…

P#7FB

Les hace daño.

Moderadora

Eso sería una dificultad para usted, eso sería una desventaja. Que usted no sabe si cuando se fumigue, los nenes peguen a las paredes las… manitas y eso, y se la metan a la boca, si les haría daño. Eso sería una dificultad.

P#9GL

Eso sería una preocupación para mí.

Moderadora

¿Y cómo se podría solucionar esa dificultad?

P#9GL

Porque ahorita yo iba a decir…

P#7FB

Una llamadita por teléfono.

P#9GL

… cuando nos está enseñando el que es primero, el que fumiga por el piso, yo tengo uno que me fumiga a mí la casa.

Moderadora

Un exterminador.

P#9GL

Ese viene detrás de las cucarachas. Ese no mata mosquitos.

Moderadora

Claro.

P#9GL

Y mata comején. Mata los terrestres…

P#7FB

Los rastreros.

P#9GL

Pero el volador no lo mata.

Moderadora

Ok. ¿Y usted quería decir algo?

P#7FB

Este… se me olvidó la pregunta.

Moderadora

Se le olvidó. [risas]

Pregunta 3- ¿Cree que esta actividad reduciría el número de mosquitos en su comunidad? ¿Por qué?

[no se hizo la pregunta]

Pregunta 3a- ¿Qué beneficios o ventajas tiene esta actividad para usted?

Moderadora

¿Qué beneficios o ventajas ustedes le ven a esto?

P#5JC

Bueno, todo lo que sea fumigar para mí es importante. Sí.

Moderadora

O sea, que le ve ventaja.

P#5JC

Yo le veo ventaja sí.

Moderadora

¿Por qué?

P#5JC

Pues porque, es que mantener el área con protección… es que siempre… yo he tenido la experiencia que donde he vivido han sido áreas que se han hecho residentes, entonces, se llenan de sabandijas de los mismos…. Mire, hace tres días atrás como cuatro días, bajé a la marquesina a buscar algo…

P#6JC

El jueves. El jueves.

P#5JC

El jueves, me encontré un cien pies que yo no había visto…

P#6JC

Enorme.

P#5JC

Enorme. Era enorme. Menos mal que tenía una escoba conmigo, y lo partí por la mitad, pero lo liquidé y después se lo enseñé a mi esposa. Y eso que está fumigada la marquesina porque ella es bien, bien… ella fumiga por todas las mesas, todo, ésta es un “Oliver Exterminating”.

P#6JC

También yo he notado, verdad. Eh, nosotros estamos en casa y hay mosquitos y el mismo me dice, ‘¿pero a ti no te están picando los mosquitos? Porque a mí me tienen asediado’. Algo tiene que ver también a veces, con el olor el humor…

P#4EST

La sangre.

P#6JC

Yo digo, ‘como tú eres diabético será que a ellos les gusta porque es…’. Algo pasa…

P#5JC

Tengo picada dulce.

P#6JC

Y es cierto. Yo estoy segura…

Moderadora

Bueno, orita yo les explico eso. Orita yo les explico eso.

P#9GL

Yo me embarro… Yo me unto “Vicks” de aquí para abajo. [risas] Yo me unto “Vicks” y no me pican. Y las orejas que yo me arropo… [más risas] Sí….

Moderadora

Ustedes por acá querían decir algo. No. ¿Ustedes por acá? Él quería decir algo.

P#2EST

No que sería… para mí sería beneficiosos porque… tendríamos que probar… pero yo entiendo que sí que debe funcionar.

Pregunta 3b- ¿Qué desventajas o dificultades le ve a esta actividad? ¿De qué forma se podrían solucionar esas dificultades?

Moderadora

Ok. Y entonces, ¿qué desventajas o dificultades, además de la que él dijo de los nietos, usted le ve? Porque usted ahorita dijo que… el número uno (*P#1EST*), cuando yo le hablé de las paredes y eso, usted hacía con la cabeza que no.

P#1EST

Pues, eso es como si estuviera rociándola completa.

Moderadora

Y, ¿por qué para usted eso es una desventaja?

P#1EST

Porque yo no sé, y si… por ejemplo, como él dice, uno mismo por ser adulto, le pega las manos cualquier cosa…

Moderadora

¿Le… eso le da inseguridad?

P#1EST

Sí, un poquito ahí.

Moderadora

Ok.

P#1EST

Porque ahora mismo, que no viene al caso, yo vengo de Estados Unidos y nosotros comprábamos unas bombas, pero teníamos que estar afuera cuatro horas para poder entrar a la casa. Nosotros la cerrábamos…

P#7FB

Eso es efectivo, y cucarachas y …

P#1EST

…y nos íbamos. Cuando entrábamos todavía quedaba el olorcito ese.

Moderadora

El olor, exacto. Y ahí dice que puede quedar olor, verdad. Sí. O sea, que eso le da a usted preocupación. Y entonces… ajá, dígame.

P#4EST

La única desventaja que yo le veo es que, como dice que tiene que ser por un profesional adiestrado, nosotros no tenemos el acceso para hacerlo. Y cómo conseguimos ese profesional adiestrado y con ellos… que sea costo efectivo.

Moderadora

Ah, que tenga un costo, verdad, que valga la pena. Exacto.

P#7FB

Entonces, nosotros no podemos…

P#4EST

No. Aplicarlo.

P#7FB

…rociar con ese insecticida. No lo podemos usar nosotros.

Moderadora

Tienen que tener una licencia para hacerlo. Y entonces, esa sería… ¿y cómo se podría solucionar esa desventaja?

P#1EST

Usándolos primero.

Moderadora

Usándolos primeros…

P#8ST

¿Quiere decir que eso no lo venden? O sea, sino… me imagino que…

Moderadora

No. Tiene que ser un profesional licenciado para eso.

P#8ST

Exacto. O sea, que no puede ir cualquier persona a comprarlo.

Moderadora

No.

P#6JC

Tiene que ser controlado.

Moderadora

No. Tiene que ser controlado.

Pregunta 4- ¿Cuán posible es realizar esta actividad en su comunidad para reducir el número de mosquitos? ¿Por qué?

Moderadora

Ok. ¿Es posible realizar esta actividad en su comunidad? Si el gobierno les dijera a ustedes, ‘mira tenemos…’, como pasó con… durante la epidemia del Zika. ‘Mira, tenemos unos fondos para rociar las casas…’, verdad, que el costo no fuera un impedimento, tampoco el exterminador, digamos el…

P#5JC

Profesional.

P#4EST

Sí…

Moderadora

…el fumigador. ¿Es posible realizar esta actividad en su comunidad?

P#4EST

Yo diría que me apunten a mí primero. [risas]

Moderadora

Que la apunten primero.

P#4EST

Vayan a casa.

P#6JC

Eso sería una novedad, antes se hacían esos servicios a la comunidad, se anunciaban. Y sería retomar otra vez esa buena costumbre.

P#9GL

¿Esto es auspicio del gobierno?

Moderadora

Esta conversación no. Nosotros estamos, nosotros somos… este programa, lo está haciendo la…. La universidad… ¿cómo le llaman?

Sue

Escuela de Medicina de Ponce.

Moderadora

La Escuela de Medicina de Ponce, junto con el… la unidad de control de vectores, que es de control de mosquitos, en San Juan, y el CDC. Nosotros queremos… que esto es la… orientación que ustedes me están pidiendo para la comunidad, luego de estos grupos vamos a hacer unas actividades en su comunidad para explicarles todo el proyecto. Verdad, queremos estar aquí por un periodo… no es por una semana ni por dos, es… si nos permiten estar en sus comunidades, la idea es estar aquí años. Verdad.

P#6JC

Claro…

Moderadora

Dando actividades de control de mosquitos, pero queremos que la comunidad participe también. Por eso estamos citando, invitando a los líderes y a los residentes para que se unan a este esfuerzo, verdad que sí. Y por eso les estamos explicando esto. Nosotros que… en nuestra… nuestra misión es educarlos a ustedes sobre todo lo que se está haciendo a nivel del mundo. Todo lo que se está haciendo a nivel del mundo. Cosa de que ustedes sepan todo. Si el gobierno decidiera, tuviera el dinero, y decidiera hacerlo, qué ustedes opinan de todas estas, cuál sería las mejores para ustedes en sus comunidades. Por eso es que estamos aquí esta noche.

P#7FB

Si le llega el mensaje al gobierno…

P#9GL

Pues usted me dio… usted me mandó… usted fue la que habló conmigo, ¿verdad?

Moderadora

No, pero una… mi compañera que se fue, Gladys.

P#9GL

Está bien, pues me dejó cinco [flyers de invitación] y de cinco, el que vine fui yo. Porque los vecinos…

Moderadora

A pues fue Jessenia el que lo llamó. Espérate.

Pregunta 5- ¿Apoya usted esta actividad en su comunidad? ¿Por qué? (*Preguntar a cada participante del grupo de discusión*)

Moderadora

Entonces, ¿apoyarían ustedes esta actividad en su comunidad? [no se pregunta a cada persona, sino que se contesta en grupo]

P#5JC

Sí.

P#6JC

Correcto. Sí.

P#9GL

Sí.

P#2EST

Sí.

P#1EST

[Participante asiente con la cabeza. Anotadores tienen en sus notas que todos los participantes apoyan la actividad.]

P#8ST

[Participante asiente con la cabeza. Anotadores tienen en sus notas que todos los participantes apoyan la actividad.]

P#4EST

[Participante asiente con la cabeza. Anotadores tienen en sus notas que todos los participantes apoyan la actividad.]

P#3EST

[Participante asiente con la cabeza. Anotadores tienen en sus notas que todos los participantes apoyan la actividad.]

P#7FB

[Participante asiente con la cabeza. Anotadores tienen en sus notas que todos los participantes apoyan la actividad.]

Pregunta 5a- ¿Piensa que su comunidad apoyaría esta actividad? Sí, No, ¿Por qué?

Moderadora

¿Piensa que su comunidad lo apoyaría?

P#6JC

Sí.

P#2EST

Claro.

P#6JC

Eso es así.

P#5JC

Sí.

Pregunta 6- ¿Qué otra información necesitaría para entender mejor esta actividad?

Moderadora

¿Qué información… qué otra información necesitarían para entender mejor esta actividad?

P#6JC

Pues la información ilustrativa, es buena.

P#8ST

Del producto, claro.

Moderadora

Del producto. Ok. Del producto que se use. Ok. Muy bien. Muy bien. ¿Alguna otra información que necesiten?

P#4EST

Que nos informen quiénes serían esos profesionales que están disponibles para hacerlo.

P#6JC

Claro…

Moderadora

Quiénes son esos profesionales. Credenciales.

P#7FB

Y el costo. Y el costo. Si es alto o bajito.

P#5JC

Y los días. Porque es que, donde yo vivo todo el mundo sale…

P#6JC

Trabaja, trabaja.

P#7FB

¿Esa es la base, verdad?

P#5JC

…nosotros… trabajan. O sea que, tendrían que ser días donde pudieran estar todos, que esos son sábados y domingos básicamente…

P#6JC

Habría que preguntar. Habría que preguntar.

Actividad #5 – Trampa AGO para mosquitos

Moderadora

Ok. Vamos para la próxima. Las trampas AGOS. A lo mejor ustedes las han oído nombrar. La trampa para mosquitos AGO, ya ha sido usada en Puerto Rico para reducir el número de mosquitos. La trampa atrae y captura los mosquitos hembras *Aedes aegypti* que buscan envases para poner sus huevos. La trampa consiste de una paila negra de cinco galones, una cámara de captura… una cámara de captura y un escrin. La paila esta llena hasta la mitad con agua y heno para atraer a los mosquitos hembras, verdad, a las hembras que son las que pican. Dentro de la cámara de captura hay un papel con pega especial que atrapa a los mosquitos cuando entran a poner sus huevos. Esta cámara atrapa las hembras. Cuando la hembra entra no puede bajar porque esta el escrin y ¿qué hace?, que se pega. Y queda pegada ahí hasta que se muere. La trampa contiene material orgánico que es esto, así que puede oler. No se requiere un entrenamiento especializado…

P#4EST

Está bueno eso…

Moderadora

...para armar la trampa. La trampa es a “custom made”. La trampa requiere mantenimiento cada dos meses, para que no se convierta en un criadero de mosquitos.

P#6JC

Imagínese.

Moderadora

Y la trampa reduce la cantidad de mosquitos si se mantiene adecuadamente y se usa en ocho de cada diez hogares de la comunidad.

P#6JC

Ah eso es bueno.

Moderadora

Y le añado yo. Hay que poner tres trampas en cada casa. Dos en el patio y una al frente.

P#7FB

Esa es buena.

P#1EST

¿Y los rotitos esos?

Moderadora

Esos rotitos es para cuando llueve… llueve, ¿qué va a pasar? El agua se va a subir si no tuviera estos rotitos y va a alcanzar y entonces sí la mosquita va a poder… se van a crear las larvitas. Y entonces, estos rotitos lo que hacen es que cuando llega a este nivel por ahí se va el exceso de agua. Cosa de que nunca los huevitos puedan tocar el agua y no se exploten y nazcan las larvitas.

P#6JC

Esa trampa… ¿es una trampa?

Moderadora

Es una trampa.

P#6JC

Ahí está la trampa.

P#9GL

Y una pregunta que voy a hacer… que me gustó eso, y que me vino la idea. Yo tengo dos vecinos, que los invité, que tienen…ahora mismo tienen piscinas plásticas grande, llena de agua en las casas. ¿Qué beneficioso…?

Moderadora

¿Qué beneficios qué?

P#9GL

¿Qué beneficios pueden tener esas piscinas, llenas de agua, que se han bañado en el fin de semana los usuarios con agua ya sucia de cuerpo de uno, agua y orín porque el que se está orinando no va a salir para ir al baño para volverse a meter a la piscina verdad? Yo lo que… ¿a qué quiero llegar con esto? Que el agua se contamina, al contaminarse cae… porque yo tengo el vecino de atrás y el de al lado… yo creo que ellos ya sabían eso, para mí las piscinas son todas plásticas, las lavan… el agua se vuelve jabón en las paredes cuando está estancada.

Moderadora

Pero eso no son trampas de mosquitos. Esos son criaderos de mosquitos.

P#9GL

No, no, no, no…

P#8ST

Una pregunta, ¿dónde se consigue esa trampa? [risas]

Moderadora

Esa trampa nosotros la diseñamos en CDC. Es algo que el entomólogo… el Dr. Barrera creó con su equipo de trabajo en nuestro centro. Esa trampa este…

P#7FB

¿Es casera?

Moderadora

Se puede hacer, porque usted ve que se puede hacer. El problema de esa trampa es que necesita una pega especial. No puede ser cualquier pega. No puede ser la pega que los ratones se pegan porque, ¿qué pasa?, con el sol se derrite y ya no sirve. No puede ser pega “crazy glue” ni nada de eso porque se pone dura y no se pegan los mosquitos. Es una pega especial que solamente…. CDC le vendió la patente, o sea, le dio la patente a una compañía, y esa compañía es la única que las fabrica. Pero no las… usa para Puerto Rico… o sea, no… aun todavía no se venden en Puerto Rico estamos en eso… Y…

P#7FB

Se va a hacer millonario si viene para acá. [risas]

Moderadora

Aun no las usan en Puerto Rico… no las venden en Puerto Rico, estamos en ese trámite, se puede… se venden por internet pero no hacen “shipping” a Puerto Rico. Ok. Pero acuérdese tiene que usarse, para que sean efectivas, en ocho de diez casas.

P#6JC

Y en cada casa tres.

Moderadora

Y en cada casa tres.

P#9GL

Nadie va a encajar con eso.

Moderadora

Bueno habían… guárdemela ahí.

Pregunta 2- ¿Es esta una actividad nueva para usted o es algo que ya había escuchado antes? Si la escuchó, ¿dónde la escuchó?

Moderadora

¿Había ustedes escuchado de esto anteriormente?

P#9GL

No.

P#6JC

Yo no.

Moderadora

¿No? ¿Ninguno? Oye y eso se hizo un proyecto de 30, 000 casa en Caguas.

P#4EST

Wow…

P#9GL

Ah, porque es Caguas.

Moderadora

Y esa gente estaban locas con esas trampas.

Pregunta 3- ¿Cree que esta actividad reduciría el número de mosquitos en su comunidad? ¿Por qué?

Moderadora

Ok. ¿Cree que esta actividad reduciría el número de mosquitos en su comunidad?

P#8ST

Sí…

P#5JC

Ajúm.

P#6JC

Claro. Y que han probado que sí.

Pregunta 3a- ¿Qué beneficios o ventajas tiene esta actividad para usted?

Moderadora

Ok. ¿Y qué beneficios o ventajas le ve a esta trampa? ¿Qué beneficios le ve?

P#7FB

Si es económica, es un buen beneficio.

Moderadora

Si es económica, sería un beneficio. ¿Por qué? ¿Por qué el beneficio?

P#9GL

Pues porque no va a enfermar la gente el mosquito que se muera ahí.

Moderadora

Ok.

P#6JC

No es algo eléctrico, ve, que es algo manual. Está en contacto con la naturaleza, se nutre de algo de la naturaleza, agua y heno. Así que tiene muchas ventajas.

P#9GL

Y en Caguas, ¿compraron ese sistema?

Moderadora

El gobierno las dio. El gobierno las dio.

P#7FB

El alcalde…

Moderadora

Si se hace un proyecto aquí en Cagu… en Ponce, y la gente en su comunidad quiere, es probable, no sabemos, puede ser probable que pase como en Caguas que el gobierno las de.

P#9GL

Eso va a ser un milagro.

Moderadora

Por el tiempo que tome el pro… bueno, pero las dieron para Caguas.

Pregunta 3b- ¿Qué desventajas o dificultades le ve a esta actividad? ¿De qué forma se podrían solucionar esas dificultades?

Moderadora

Este, usted me dijo… usted puso como un ‘pero’ orita. ¿Qué es la desventaja o la dificultad que usted le ve? Que yo le dije, aguántemelo ahí.

P#9GL

Pues vamos… vamos a poner que yo pueda comprar tres, porque el mínimo, son tres. En la periferia mía, las casa son alquiladas. Vive gente, pero son alquiladas. Entonces, este… la mayor parte están cerradas. Mas tengo dos que tienen piscinas. Piscinas grandes, donde pueden meterse 15 a 20 personas adultas y disfrutan el cuatro de julio ahí mejor que todo el mundo. Pero, yo encuentro como que la comunidad no vaya a responder que lo cojan de diversión, y que mis vecinos no vayan a ser mis vecinos.

Moderadora

Entonces, usted piensa que no todo el mundo en la comunidad la va a querer tener. Esa es la desventaja que ve usted.

P#9GL

Yo lo invité y él conoce del área, él vive aquí y vine yo y vine tarde. La gente es apática a ver lo que es constructivo.

Moderadora

Ajá. Ok que usted piensa que no todo el mundo lo va a querer tener. O sea…

P#5JC

Exacto. Y el mantenimiento, es cuestión de que…

P#2EST

Orientando… [dice en volumen bajito]

P#5JC

…o sea, nadie va a estar pendiente a cambiar…

Moderadora

Usted cree que, aunque sea dos… cada dos meses, nadie va a estar pendiente a cambiar.

P#4EST

Al cambio.

Moderadora

Ok. Al cambio. ¿Y usted qué quería decir?

P#2EST

Yo entiendo que si se les orienta a las personas y se les da una explicación de cómo funciona, yo entiendo que la gente la van a querer. Porque en realidad es algo más fácil y más rápido, porque funcionaría más rápido que ustedes breguen con eso y echarle los cositos [larvicidas] acá al agua yo entiendo que se controlaría bastante.

P#4EST

¿Y el mantenimiento, se lo tiene que dar el usuario en la casa? ¿Y orientan a uno cómo hacerlo?

Moderadora

Sí.

P#4EST

Ok.

P#7FB

¿Y es eficiente al 100%?

P#6JC

Yo comparto con él. Todo en la vida, todo en la vida, cuando uno compra algo, tiene que aprenderlo a usar y le tiene que dar un mantenimiento. Que compré una aspiradora. No va a estar toda la vida usando hasta que no… tiene que cambiarle el filtro, tiene que limpiarla, si es de agua se cambia. Es integrar que es algo más que uno le da mantenimiento, porque eso tiene que ver con la salud de uno.

Pregunta 4- ¿Cuán posible es realizar esta actividad en su comunidad para reducir el número de mosquitos? ¿Por qué?

Moderadora

Ok. Y entonces, ¿usted cree que es posible que el uso de las trampas se pueda realizar en su comunidad?

P#2EST

Sí. Yo entiendo que sí.

P#5JC

Yo lo veo ahí…

Moderadora

¿Por qué?

P#5JC

Vuelvo y le digo es que somos vagos. [risas]

Moderadora

Somos vagos.

P#5JC

Sí, es verdad. ¿Qué trabajo nos cuesta cada dos meses? Yo lo haría, pero el resto…

Moderadora

O sea, que ya hay dos cosas que serían una desventaja, es que todas las casa las tuviera y que la gente le diera mantenimiento. ¿Cómo podría… como se podría solucionar esos dos impedimentos?

P#5JC

Bueno que… si hay… habría una compañía que las trajera y las pusiera cada dos meses…

P#4EST

Que le dieran seguimiento. Exacto.

P#5JC

Que le dieran seguimiento.

P#7FB

Pero así sube el costo…

P#5JC

Porque es que darle el seguimiento uno es el problema.

Moderadora

¿Cómo dijo?

P#7FB

Eso sería un costo adicional a la trampa.

Moderadora

Exactamente.

P#7FB

El mantenimiento que se le dé a la trampa…

P#9GL

Miss, una pregunta que yo le iba a hacer. Con todo respeto que usted se merece.

P#7FB

Es buena idea, lo de hacerlo uno.

P#9GL

¿Usted es de aquí de Ponce?

Moderadora

No, yo soy de Carolina.

P#9GL

Buen pueblo. Popular. [risas]

P#7FB

Con calma que popular y pnp están empatados, son igual de amigos.

[siguen con chistes políticos]

P#9GL

Aquí al frente, donde termina el garaje de gasolina, esta la carretera y está la escuela. Detrás de esa escuela el rio que baja por ahí… hicieron unos “deliveries”, que son unos niveles de cemento para que el agua baje. Pues al lado de allá de la carretera yendo para Mayagüez, está el lago grande de allá, pero acá hay tremendo lago, aquí al detrás del piso de casa. Donde vive aquí el amigo, es donde termina la carretera de esquina donde está la tienda, allá hay otro estanque más de agua. Colinda con la cantera y con ustedes. Esos son charcos que antes no existían, charcos, semi lagos. Eso es criadero… ahí meten caballos, meten vacas, que eso dan garrapatas y criaderos de muchos insectos. O sea que…

Moderadora

¿Tiran basura?

P#9GL

No creo porque la verja es… no creo, porque la verja es alta. Pero la basura la tira la gente después de las once de la noche que está todo el mundo durmiendo.

Moderadora

Ok. Orita hablamos de eso. Le explico orita.

Pregunta 5- ¿Apoya usted esta actividad en su comunidad? ¿Por qué? (*Preguntar a cada participante del grupo de discusión*)

Moderadora

¿Apoyarían ustedes, esta actividad en su comunidad? [no se pregunta a cada persona, sino que se contesta en grupo]

P#7FB

Yo sí.

P#5JC

Sí.

P#1EST

[Participante asiente con la cabeza. Anotadora tiene en sus notas que todos los participantes apoyan la actividad.]

P#2EST

[Participante asiente con la cabeza. Anotadora tienen en sus notas que todos los participantes apoyan la actividad.]

P#3EST

[Participante asiente con la cabeza. Anotadora tienen en sus notas que todos los participantes apoyan la actividad.]

P#4EST

[Participante asiente con la cabeza. Anotadora tienen en sus notas que todos los participantes apoyan la actividad.]

P#6JC

[Participante asiente con la cabeza. Anotadora tienen en sus notas que todos los participantes apoyan la actividad.]

P#9GL

[Participante asiente con la cabeza. Anotadora tienen en sus notas que todos los participantes apoyan la actividad.]

P#8ST

[Participante asiente con la cabeza. Anotadora tienen en sus notas que todos los participantes apoyan la actividad.]

Pregunta 5a- ¿Piensa que su comunidad apoyaría esta actividad? Sí, No, ¿Por qué?

Moderadora

Creen… ¿Piensan que su comunidad apoyaría esta actividad?

P#2EST

Sí.

P#7FB

Sí.

P#6JC

Seguro que sí.

P#4EST

Si se les orienta bien…

P#7FB

Habría que orientarlos a ellos.

Moderadora

Orientarlos a ellos.

P#9GL

Sí…

Pregunta 6- ¿Qué otra información necesitaría para entender mejor esta actividad?

Moderadora

¿Qué información necesitarían tener para hacer esta realidad?

P#9GL

Pues la poquita que uno se lleva de aquí ahora.

Moderadora

Ok.

Pregunta 7- ¿Considerarían realizar ustedes mismos esta actividad?

Moderadora

¿Considerarían ustedes mismos realizar esta actividad? O sea, ¿ustedes mismos, pondrían las trampas y les darían el mantenimiento?

P#7FB

Yo sí, yo le daría mantenimiento.

Moderadora

¿Sí?

P#2EST

Sí.

P#6JC

Correcto.

P#7FB

¿Dónde está?

Moderadora

Ya usted se llevó el plano. [risas]

P#7FB

Tiene una por ahí que me obsequie.

Pregunta 8- ¿Qué les haría difícil realizar esta actividad?

Moderadora

¿Qué le haría…? ¿Qué se les haría…? ¿Qué se le haría a usted difícil, para realizar esta actividad? ¿Qué sería un impedimento para ustedes para realizar…?

P#5JC

Conseguir la pega.

Moderadora

Conseguir la pega. Ave María, estaba atento, estaba escuchando. Ese es el proyecto, porque el heno lo puede conseguir donde quiera…

P#7FB

Ese es el problema.

P#6JC

Exacto. Poner la pega.

P#7FB

¿La pega es la que atrae la hembra?

Moderadora

No, el heno. El agua con heno. La pega lo que hace…

P#6JC

Cuando la atrapa.

P#9GL

El que se queda pegado allí, ahí se quedó.

P#4EST

La atrapa.

Moderadora

La mosquita… lo bueno de esta trampa es que atrapa a las mosquitas que ya picaron, que son las que transmiten virus porque se lo pega… picó… él está enfermo, la mosquita no está enferma, pero él tiene el virus. La mosquita lo pica a él y le chupa el virus y cuando lo pica a usted se lo transmite a usted.

P#5JC

Exacto…

Moderadora

Pues esta trampa es buena porque…

P#5JC

Recoge…

Moderadora

Recoge, mata, captura a las que ya picaron que tienen virus. Se quedan ahí pegadas. Y ni ella ni sus hijos vuelven a dar…

P#9GL

O sea, que el plástico ese es el macho de la hembra que entra ahí.

Pregunta 9- ¿Hay algo que podría ayudarles a realizar esta actividad de manera más fácil?

[No se hizo la pregunta]

Pregunta 9a- ¿Necesitarían más información?

[Se contestó en otro lado]

Pregunta 9b- ¿Necesitarían más adiestramiento?

Moderadora

Ok. Este… ¿Necesitarían adiestrarse para hacer esta… hacer el mantenimiento…?

P#6JC

Claro que sí.

Moderadora

Y poner la trampa.

P#7FB

Hacerla…

Moderadora

Armar la trampa. ¿Sí necesitarían? ¿Qué informa… qué otra información necesitarían para hacer esta tram… esta actividad?

P#6JC

Dónde se consiguen los materiales.

P#4EST

Tiene que ser negra…

Moderadora

Dónde se consiguen los materiales.

P#6JC

Exacto.

[parece haber una conversación sobre los materiales de la trampa y cómo hacerla entre P#4EST y P#8ST o P#1EST, pero no se escuchan lo suficiente como para entenderlos]

P#9GL

¿Qué alto tiene… qué alto tiene eso?

P#7FB

Eso es como una paila, ¿no?

P#6JC

Sí, eso es una paila de 5 galones.

[muchos hablando a la vez, y no se entiende lo que dicen]

P#9GL

O sea, que con dos pailas… con dos pailas se resuelve eso.

Moderadora

[no se entiende lo que dice el moderador al principio] no, esto es otra cosa.

P#6JC

No tres…. Ahora mismo venden pailas de diferentes…

P#9GL

No, no, me refiero donde está el agua.

Moderadora

Sí, esto es una paila, esto es una paila completa…

P#8ST

Sí, lo que pasa es que la ponen así…

Moderadora

Esta es la tapa. Esta es la tapa de la paila, que se le hace un hueco aquí, y se hace una cámara de captura.

Actividad #6 – Mosquitos macho y hembra con Wolbachia

Moderadora

Ok. Vamos para el próximo que nos quedan tres, tres. A ley de nada, vamos. Ok. Ahora vamos a los mosquitos estériles. Ahora vamos a hablar de los mosquitos con Wolbachia. Wolbachia es una bacteria que vive en muchos insectos incluyendo algunas especies de mosquitos que pican a las personas. Pero la Wolbachia no se encuentra casi nunca en los mosquitos *Aedes aegypti* que son los que transmiten el dengue, el Zika y el Chikunguña en Puerto Rico. La Wolbachia se introduce a los mosquitos *Aedes aegypti* en el laboratorio. Al presente, los estudios muestran que el uso de mosquitos con Wolbachia…

P#4EST

Ah… yo sé. Un mosquito que estaban hablando…

Moderadora

…es seguro para las personas, los animales y el ambiente. Los científicos creen que cuando nacen mosquitos con Wolbachia pueden ser menos capaces de transmitir enfermedades a las personas. Pásale. Los mosquitos con Wolbachia funcionan de dos maneras diferentes. Vamos a hablar de la primera. En la primera se liberan mosquitos *Aedes aegypti* machos y hembras con Wolbachia. Cuando el mosquito hembra con Wolbachia se reproduce, o sea, que se une, a un mosquito macho con o sin Wolbachia la bacteria se pasa a través de la hembra a sus crías, de generación en generación. ¿Lo ven? El macho con Wolbachia, la hembra con Wolbachia, van a tener mosquitos con Wolbachia. Pero si este macho con Wolbachia o esta hembra con Wolbachia se parean con un macho y hembra… se unen con un macho y hembra que no tienen Wolbachia, como quiera esos mosquitos van a nacer con Wolbachia.

P#6JC

Porque es dominante.

Moderadora

Y entonces, después de liberarlos varias veces la población de mosquitos, espérate… aquí voy. Con el tiempo la cantidad de mosquitos con Wolbachia aumenta y reemplaza, reemplaza, a los mosquitos del ambiente que no tienen la bacteria. Después de liberarlos varias veces la población de mosquitos con Wolbachia se mantendrá sin tener que liberar mas de estos mosquitos. Los mosquitos con Wolbachia son menos capaces de transmitir enfermedades. Aun habrá mosquitos en la comunidad, y ¿qué quiere decir eso? Si hay mosquitos en la comunidad, te van a picar. Claro, hay menos probabilidad de que te transmitan enfermedades. Eh… aun habrá mosquitos en la comunidad ya que la intención de este método no es reducir el número de mosquitos sino reducir el riesgo de epidemias. Sin embargo, no se reducirá la cantidad de mosquitos como habíamos dicho. Esta actividad se ha usado en otros países como P#4EST y P#6JC. Actualmente no hay reglas definidas para el uso de mosquitos macho y hembras con Wolbachia en los Estados Unidos. Aunque, ha habido noticias de que se van… que se están ya utilizando, de que se van a utilizar en la Florida. Ok entendieron el diagrama, verdad, el dibujo.

P#6JC

Claro que sí.

P#7FB

Sí.

Pregunta 2- ¿Es esta una actividad nueva para usted o es algo que ya había escuchado antes? Si la escuchó, ¿dónde la escuchó?

Moderadora

Ok. Les pregunto, ¿habían escuchado de esta actividad anteriormente?

P#6JC

No.

P#1EST

No.

P#4EST

Yo sí.

Moderadora

¿Sí? ¿Dónde?

P#4EST

No puedo dar con certeza, pero sí había escuchado, que se lo había comentado a ella, que se mataban entre ellos mismos como quien dice.

Moderadora

Ok. Ok. No sabe dónde lo escuchó, pero lo escuchó. Ok.

Pregunta 3- ¿Cree que esta actividad reduciría el número de mosquitos en su comunidad? ¿Por qué?

Moderadora

Y entonces, ¿cree que esta actividad… los mosquitos con Wolbachia, macho y hembra, los dos, reducirían el número de mosquitos en su comunidad?

P#8ST

Con lo que usted me da…

P#4EST

Bueno, yo entiendo que no, porque el mosquito va a seguir picando.

Moderadora

Exacto.

P#6JC

Exacto, yo creo que no, que va a seguir…. Pero, el riesgo de las personas contaminarse, mientras más crece esa población, pues ya la otra población ha bajado y el riesgo de que las personas adquieran enfermedades pues ya es menor.

Moderadora

Es menor. Exacto.

P#4EST

Eso es, si es así…

P#7FB

¿Qué es la Wolbachia?

Moderadora

La Wolbachia es una bacteria que existe.

P#6JC

Un animalito chiquito, microscópico…

Moderadora

En es… es una bacteria que existe en el ambiente. Y la tienen muchos insectos. Incluso la tienen mosquitos que nos pican… porque déjeme decirles, *Aedes aegypti* es un mosquito, el que transmite el *Aedes*… el dengue, el Zika y el Chikunguña. ¿Pero sabe cuántos mosquitos hay en Puerto Rico identificados? 44 especies de mosquitos diferente.

P#9GL

Va a ser bien difícil extinguirlos. [risas]

Moderadora

Pero nosotros lo que queremos extinguir son los que transmiten…

P#6JC

Las epidemias.

P#7FB

Las enfermedades.

Moderadora

Las epidemias. Porque déjeme decirles, el *Aedes aegypti*, además de estas tres, también transmite fiebre amarilla.

P#6JC

Ah sí.

Moderadora

Ahora mismo hay un brote en el P#6JC de fiebre amarilla. ¿Se acuerda? Que las teníamos hace dos siglos atrás, en los 1800 aquí había fiebre amarilla.

P#6JC

Claro que sí.

P#9GL

Sí, pero allá también es la falta de alimentación.

Moderadora

Bueno…

P#6JC

Aquello era… de eso moría la gente.

P#9GL

Porque el mosquito, el mosquito mundialmente está en todos lados.

P#7FB

Él se ha vuelto…

P#9GL

[no se entiende el comienzo] la señora…

Moderadora

Acuérdese que el mosquito…

P#9GL

Que ella joven, ella siendo joven veía que en el ejercito hacían ese tipo de… ¿Por qué no lo siguieron haciendo [no se entiende la palabra] enfermos?

Moderadora

Porque los mosquitos, acuérdese que los mosquitos transmiten el virus, verdad. Porque pican una persona infectada con el virus. Claro, si la persona tiene un sistema inmunológico que no es tan bueno, pues la enfermedad le va a dar peor y ahí viene lo que usted dice, si la persona no está bien alimentada, no tiene un buen sistema inmunológico, y le va a dar peor la enfermedad. Pero una persona bien alimentada, también se puede infectar con el virus.

P#9GL

Sí, sí, sí… eso nos coge.

P#6JC

Pero resiste, aguanta los síntomas y sale adelante. Otros que han quedado…

Moderadora

Sí. ¿Usted?

P#4EST

Tengo este… una duda. ¿Qué probabilidades hay o estudio científico habrá de que la Wolbachia esa no se le pegue al ser humano, o no le afecte o nos vaya a contaminar con otra cosa?

Moderadora

O sea, que usted…que usted tendría… usted necesitaría más información sobre la Wolbachia.

P#9GL

Sí…

P#4EST

Sí, porque…

P#9GL

Indiscutiblemente.

P#4EST

… ¿qué nos garantiza que no nos de otra cosa?

Moderadora

Ok. Esa sería una desventaja para usted. Una dificultad.

P#4EST

Sí…

Moderadora

Que usted tendría que saber si la Wolbachia se le puede pegar a la persona y le puede causar daño. Ok. Muy bien.

Pregunta 3a- ¿Qué beneficios o ventajas tiene esta actividad para usted?

Moderadora

¿Qué beneficios usted le ve a esta actividad? ¿Qué ventajas le ve a esta actividad?

P#1EST

Lo que aprendemos… aprendemos.

Moderadora

No, no. A la actividad esta no. Al uso de mosquitos hembra y machos con Wolbachia. ¿Usted le ve alguna ventaja, algún beneficio?

P#8ST

Reduce el riesgo de la epidemia.

Moderadora

Reduce el riesgo de la epidemia.

P#2EST

Se ha comprobado que reduce el riesgo.

P#6JC

Claro.

P#2EST

Y ahora mismo que nosotros tenemos… porque hay mosquitos en todos lados como uno dice, ¿y se ha reducido la… el dengue, el Zika, y todo eso? ¿Sería a cambio de eso?

Moderadora

Lo que pasa es que los estudios que se han hecho como dice ahí, verdad, pues son en áreas limitadas. Habría que ver como funcionaría en un área extendida.

P#2EST

Por eso, pero ahora mismo está controlado la cuestión del Zika, dengue. Ya no se oye tanto como antes que todo el mundo tenía Zika, tenía el dengue, tenía el desto…

Moderadora

Ah, que usted… ah ok.

P#7FB

Una pregunta. La Wolbachia, ¿está en Puerto Rico el estudio o han hecho los…?

Moderadora

Wolbachia lo hay en… es una bacteria de la naturaleza, está en la naturaleza. La hay en Puerto Rico y la hay en todo el mundo.

P#7FB

¿Y es… se le inyecta al mosquito o el mosquito…?

Moderadora

Sí. Sí. Mire como funciona esto, le voy a explicar, porque esta pregunta me la han hecho en otros grupos. Los mosquitos ponen sus huevos, esos huevos se conservan, verdad en una lámina en un papel. En un papel de germinación. Si alguien sabe aquí qué es un papel de germinación, pues las mosquitas ponen sus huevos ahí, verdad. Y entonces, en el laboratorio esos huevitos se sumergen en el agua y salen las larvas. Y las larvas pues crecen y demás. Y entonces, cuando están hechas mosquitos pues se les pone la bacteria, ve. Se le pone la bacteria y se libera, de esa forma es que funciona.

P#6JC

Y a través del microscopio, uno las ve y sabe lo que es macho, cuál es hembra, las empareja y todo eso…

Moderadora

Claro. Los machos tienen el bigote más largo, las antenitas, tienen un bigote bien frondoso.

P#6JC

Sí, exacto.

Moderadora

Las hembras tienen un bigote más… menos frondoso. No tienen tantos pelitos. Y entonces… acuérdense que los machos no pican, y que las hembras sí.

P#6JC

Es correcto.

Pregunta 3b- ¿Qué desventajas o dificultades le ve a esta actividad? ¿De qué forma se podrían solucionar esas dificultades?

[No se hizo la pregunta]

Pregunta 4- ¿Cuán posible es realizar esta actividad en su comunidad para reducir el número de mosquitos? ¿Por qué?

Moderadora

Bueno, sigo por aquí. ¿Usted cree que es posible que esta actividad se pueda realizar en su comunidad? Aquella dice que sí. ¿Por qué?

P#4EST

Bueno, pero entonces tendrían que… ¿te refieres a liberar mosquitos con Wolbachia en la comunidad?

Moderadora

¿Usted cree que es posible?

P#4EST

Bueno tendrían que consultarles a las personas primero…

Moderadora

Exacto, que usted cree que…

P#4EST

… ver si ellos están de acuerdo…

Moderadora

Exacto. ¿Usted cree que la comunidad…?

P#4EST

…de ser posible, sería.

Moderadora

¿…lo apoyaría?

P#4EST

Si son bien orientados, sí.

P#6JC

Es correcto.

Moderadora

¿Y usted qué dice?

P#1EST

También.

Moderadora

Que si son bien orientados sí.

P#1EST

Exacto. Seguro que sí.

P#5JC

Si no tienen que hacer mucha cosa, sí.

Moderadora

Si no se tiene que hacer mucha cosa, ¿a qué usted se refiere con mucha cosa?

P#5JC

O sea, si la actividad… no necesitan hacer actividad ellos, la comunidad…

Moderadora

Ah, ok…

P#5JC

…me refiero…

P#4EST

La vagancia.

P#5JC

…sabes, ellos te aceptan todo, la vagancia es…

P#4EST

Exacto.

Moderadora

Esto no lo puede hacer una persona. Esto lo tiene que hacer el gobierno.

P#5JC

Exacto.

P#4EST

Exacto.

P#6JC

Eso.

Moderadora

Si los ciudadanos dicen que sí, que quieren hacer esto, pues el gobierno lo puede hacer. Sin autorización del gobierno no se puede hacer. Sin autorización, o sea sin… el…por eso se piden las opiniones de los ciudadanos, ve. Para poder hacerlo. Usted quería decir algo y después voy con usted. Dígame.

P#7FB

La Wolbachia, ¿es dañina a las personas, animales? ¿Podría llegar a ser dañina a las personas y animales?

Moderadora

Que yo sepa… no le puedo dar eso. Yo le puedo buscar información y hacérsela llegar. Pero yo entiendo que si es una bacteria que está en el ambiente… no, porque hemos vivido con ella. Hemos vivido con ella. Pero vale la pregunta y se le podría traer información. De hecho, esa sería una información que las personas necesitarían para poder apoyar esa… esta actividad, verdad. El uso de los mosquitos… Usted quería decir algo.

P#6JC

A eso yo voy, lo que usted acaba de explicarle a él. Que después que a la comunidad se le explique, se le ilustre y se le comparta estos conocimientos, porque ahora mismo yo estoy aprendiendo muchísimo, pues ellos también, pues claro que lo ven como algo beneficioso.

P#5JC

Exacto.

P#6JC

Para la salud y el ambiente.

P#7FB

¿Pero sabe a quién no va a ser beneficioso? A los que venden insecticida y esas compañías… [risas]

Moderadora

Ah bueno, pero ellos que breguen. [más risas]

P#7FB

A ellos no les va a beneficiar nada.

Moderadora

Ellos que breguen.

P#6JC

En la comunidad no tenemos ninguno de esos, así que…

Moderadora

Ellos que breguen. Y entonces… ok y entonces, ustedes piensan que sería posible hacer esa actividad en la comunidad siempre y cuando se orienten a las personas.

P#5JC

Claro.

Pregunta 5- ¿Apoya usted esta actividad en su comunidad? ¿Por qué? (*Preguntar a cada participante del grupo de discusión*)

Moderadora

Ok, ¿apoyaría usted esta actividad en su comunidad?

P#7FB

Yo sí.

P#4EST

Sí.

P#5JC

Sí.

Moderadora

¿Todos?

P#6JC

Yo sí.

P#9GL

Es que… vuelvo y digo…

Moderadora

No veo nada por aquí. Está en blanco.

P#9GL

Pues yo lo que digo es que…

P#8ST

Sí, yo lo apoyo, sí.

P#9GL

…necesitamos que la comunidad…

Moderadora

¿Sin reservas o con reservas?

P#8ST

Bueno.

Moderadora

¿Por qué? ¿Qué reservas?

P#8ST

Habría que… no, no, porque habría que tener más información.

Moderadora

Más información. ¿De qué?

P#8ST

Sobre la…

Moderadora

¿La bacteria?

P#8ST

Exacto.

P#9GL

…Para que la gente, pueda... viendo esto, porque uno le lleva eso a ellos, y no le van a creer a uno.

P#4EST

Viéndolo como ellos lo están viendo, yo tendría mis reservas en cuanto al gobierno.

Moderadora

¿Por qué?

P#4EST

Con el gobierno que estuviese de turno, porque no estoy hablando del de ahora, o el que vendrá o el que habrá. La comunidad, el pueblo, tendría que confiar, para delegar en ellos esa tarea. Porque y si en vez de Wolbachia le echan otra cosa y nos matan a nosotros. ¿Me entiende?

Moderadora

¿Y cómo se puede solucionar eso?

P#4EST

Bueno, ahí le toca al gobierno hacer su parte de que el pueblo…

Moderadora

¿Qué parte?

P#4EST

De ganarse la confianza y la credibilidad del pueblo para que se den.

P#7FB

Sí, como hacen para las elecciones… [risas]

P#4EST

Para que puedan implementar eso. Que no cambien nada, que sea eso mismo.

Moderadora

Que sea exactamente Wolbachia, que no sea otra cosa.

P#4EST

Es cuestión de credibilidad.

Moderadora

Ok.

Pregunta 5a- ¿Piensa que su comunidad apoyaría esta actividad? Sí, No, ¿Por qué?

Moderadora

¿Piensa usted que la comunidad apoyaría esta… el uso de mosquitos macho y hembras con Wolbachia?

P#5JC

Sí.

P#6JC

Claro.

P#4EST

Si son bien orientados, sí.

P#7FB

Si se le hacemos una promoción de que ese…

P#4EST

Exacto.

P#7FB

… Wolbaria [Wolbachia] va a funcionar para eliminar… no, de hecho, indirectamente, las enfermedades, yo creo que sí.

Pregunta 6- ¿Qué otra información necesitaría para entender mejor esta actividad?

[Se contestó en otro lado]

Actividad #7 – Mosquito macho con Wolbachia

Moderadora

Ok. Vamos para… ya nos quedan dos nada más. Próxima. Esto es, machos solamente con Wolbachia. Acuérdense, la primera era machos y hembras. Yo les dije que la Wolbachia funcionaba de dos maneras, la primera era machos y hembras, ahora es solamente machos. En la segunda forma de usar esta actividad, solo se liberan mosquitos macho con Wolbachia. Se liberan mosquitos macho al ambiente, que no pican ni transmiten enfermedades. Acuérdense lo que les había dicho, los mosquitos macho no pican, pican las hembras porque necesitan sangre para producir sus huevos. Pero como los machos no ponen huevos, no pican, verdad. Así es que, no pican los machos, ni transmiten enfermedades, porque no pican. Entonces, los mosquitos macho con Wolbachia se unen con las hembras sin Wolbachia que hay en el ambiente. Y entonces, las hembras sin Wolbachia ponen sus huevos, pero estos no nacen, ve. El macho con Wolbachia se libera en el ambiente, se tira, y se casa con las mosquitas del ambiente que no tienen Wolbachia y ponen los huevos, pero los huevos no nacen. Y entonces, los mosquitos con Wolbachia deben liberarse continuamente, en grandes cantidades para mantener baja las poblaciones de mosquitos. Una vez que los mosquitos con Wolbachia dejan de ser liberados en una área, la población de mosquitos aumentará de nuevo. O sea, que siempre hay que estar tirando los mosquitos macho con Wolbachia para que esas poblaciones de mosquitos se mantengan bajas. El día que se deje de hacer…

P#2EST

Vuelven de nuevo.

Moderadora

Los mosquitos macho con Wolbachia se han utilizado en estudios en California, en los Cayos de la Florida. Y han sido probados en Miami, Florida.

Pregunta 2- ¿Es esta una actividad nueva para usted o es algo que ya había escuchado antes? Si la escuchó, ¿dónde la escuchó?

Moderadora

¿Habían escuchado esto antes?

P#6JC

No.

P#5JC

No.

P#2EST

No.

Moderadora

¿Nadie había escuchado?

P#4EST

Esa es la que yo decía que los mataba.

Moderadora

Esa es la que usted decía que había escuchado, verdad.

P#4EST

Que los mataba.

Pregunta 3- ¿Cree que esta actividad reduciría el número de mosquitos en su comunidad? ¿Por qué?

Moderadora

¿Cree que esta actividad reduciría el número de mosquitos en su comunidad? Número uno (*P#1EST*).

P#1EST

Sí.

Moderadora

¿Sí lo cree? ¿Por qué?

P#1EST

Pues porque, como usted dice, si funcionó en Miami, Florida, en Puerto Rico tiene que funcionar también. [risas]

Moderadora

Si funcionó allá, tiene que funcionar aquí.

P#1EST

Tiene que funcionar acá también.

Pregunta 3a- ¿Qué beneficios o ventajas tiene esta actividad para usted?

Moderadora

Ok. ¿Qué beneficios o ventajas ustedes le ven a esta actividad?

P#5JC

Pues, eliminar los mosquitos, esa es la prerrogativa de esto. Que es lo que queremos.

Moderadora

¿Eliminar los mosquitos?

P#6JC

Sí. Y otra… y una observación que yo hago es que prácticamente es una forma de quedar estéril las…

P#4EST

Las hembras.

P#6JC

…las hembras. Porque, y si ponen huevo que no germina pues queda estéril.

Moderadora

Exactamente.

P#5JC

Es la mejor forma de eliminar…

P#4EST

Y el beneficio que yo le veo es que mientras se esté usando la bacteria, va a funcionar. Porque no van a producir.

Moderadora

Exacto.

P#4EST

Lo malo es si las dejan de inyectar.

P#7FB

Pero, ¿y los mosquitos que no tienen la Wolbachia y procrea?

Moderadora

Hasta el día que se case con uno que tenga Wolbachia.

P#7FB

Por eso…

Moderadora

El día que se case con uno que tenga Wolbachia…

P#6JC

Ahí queda.

Moderadora

…los huevos no nacen. Los pare, pero no nacen.

P#4EST

No. No. Eso está bueno.

P#7FB

Eso estaría bien.

Moderadora

¿Qué les parece esa? Comparándola con la otra. Comparándola con la otra de Wolbachia de machos y hembras.

P#7FB

Mejor es que no nazcan.

P#6JC

Sí.

Moderadora

Mejor es que no nazcan, ¿verdad?

P#6JC

Correcto. Sí.

P#4EST

¿No se pueden aplicar de las dos formas?

P#6JC

Sí…

Moderadora

¿Cómo?

P#4EST

Pues en unos ponerle al macho y la hembra y en otros así que sean los macho nada más.

P#7FB

Es un insecto que no hace falta…

P#4EST

O sea, aplicar, como yo digo, las dos estrategias. Por decirlo así. Si se podría… si se acaba más rápido.

Moderadora

Voy a preguntarlo, porque esa no me la sé. Pero… no voy a decir… lo voy a decir al final…

P#4EST

Es la combinación de estrategias.

Moderadora

…lo voy a decir al final. ¿Alguien más tiene una opinión sobre esta?

Pregunta 3b- ¿Qué desventajas o dificultades le ve a esta actividad? ¿De qué forma se podrían solucionar esas dificultades?

Moderadora

¿Qué desventajas le ven? ¿Le ve alguna desventaja número ocho (*P#8ST*)? ¿No? Y la… y el número nueve (*P#9GL*), ¿le ve alguna desventaja?

P#9GL

No entiendo… estoy confundido porque el que pone los huevos, no lo puede producir, no sabe uno si es que el otro lo puede producir y sigue él caminando… O sea, no, no…

Moderadora

El macho no puede poner huevos. Es la hembra.

P#9GL

Es la hembra, está bien.

Moderadora

Si el macho se casa con una hembra…

P#9GL

Pero nosotros somos los que no sabemos quién es el macho y la hembra. [risas]

P#4EST

No, pero, el laboratorio sabe, que son los que lo van a inyectar.

Moderadora

El laboratorio sabe.

P#4EST

Yo la única desventaja que veo es si se… como dice allí, si se dejan de ser liberados va a volver otra vez la población de los mosquitos…

Moderadora

¿Y cómo se solucionaría eso?

P#4EST

Bueno, es un poco difícil, pero habría que tener siempre la seguridad, la certeza, de que alguien lo esté haciendo para que no se propaguen.

Moderadora

Ok.

P#6JC

Tiene que haber un programa de una continuidad.

P#4EST

Exacto.

P#6JC

Para que esos mosquitos sean liberados en grandes cantidades. Esa… esa es la dificultad. Si ese paso no se hace pues el programa no es efec… exitoso.

P#7FB

Sí. Eso es verdad lo que dice ella. Porque como el mosquito se reproduce tan rápido, lo de la enfermedad, pues si combaten por un lado y no mantienen la continuidad, va a seguir la enfermedad.

P#9GL

¿El mosquito en dos semanas ya es mosquito? Verdad, en dos semanas más o menos.

Moderadora

¿Ah?

P#7FB

En siete días.

P#9GL

El mosquito se enP#9GL en dos semanas y ya está.

Moderadora

En una.

P#9GL

En una. Vea…

Moderadora

En una. Ok…

P#9GL

Está uno… perdón, está uno matando uno, pero son como los chinos. Uno mata un chino, pero aparecen diez por allá.

Moderadora

Son como los güimos. [risas]

Pregunta 4- ¿Cuán posible es realizar esta actividad en su comunidad para reducir el número de mosquitos? ¿Por qué?

Moderadora

Ok. ¿Es posible que esta actividad se pueda realizar en su comunidad?

P#5JC

Sí.

P#6JC

Sí.

Pregunta 5- ¿Apoya usted esta actividad en su comunidad? ¿Por qué? (*Preguntar a cada participante del grupo de discusión*)

Moderadora

¿Ustedes apoyarían el uso de esto…?

P#4EST

Yo apoyo. Yo apoyo.

P#5JC

Sí.

Moderadora

¿Todos apoyan?

P#8ST

Claro, exacto.

Moderadora

Usted apoya.

P#1EST

[parece contestar sí con la cabeza, no se escucha una negación en el audio]*

Moderadora

Lo apoya.

P#2EST

[parece contestar sí con la cabeza, no se escucha una negación en el audio]*

Moderadora

Apoya.

P#3EST

[parece contestar sí con la cabeza, no se escucha una negación en el audio]*

Moderadora

Apoya…

P#9GL

Se puede.

P#7FB

El mosquito por poco me mata así que hay que hay que apoyarlo.

Moderadora

¿Apoya?

P#9GL

[parece contestar sí con la cabeza, no se escucha una negación en el audio]*

Moderadora

¿Apoya?

P#8ST

[parece contestar sí con la cabeza, no se escucha una negación en el audio]*

*** Anotadora presencial tiene en sus notas que todos contestaron que sí.**

Pregunta 5a- ¿Piensa que su comunidad apoyaría esta actividad? Sí, No, ¿Por qué?

Moderadora

¿Ustedes creen que en su comunidad apoyarían esta actividad?

P#5JC

Sí.

P#2EST

Sí.

P#6JC

Yo también.

Pregunta 6- ¿Qué otra información necesitaría para entender mejor esta actividad?

Moderadora

¿Qué información necesitarían tener para entender mejor esta actividad?

P#9GL

Para apoyar la actividad, perdón, hay que usar el sistema de… de la cosa esta, ¿de la paila?

Todos

No.

Moderadora

No. No. Esta es otra. Esta otra. [risas] No lo mezcle con la paila.

P#8ST

Este mosquito se ha pasado picando desde que llegó… Está perdido, desde que llegó… [más risas]

Moderadora

La número seis (*P#6JC*) quería decir algo.

P#6JC

Pues, claro, para llevar esta novedad porque llevar… a la comunidad este método, es una novedad, así que necesitamos suficientes… ilustrar y explicar este método para convencerlos.

P#9GL

Exactamente.

P#7FB

A cada… llevarles un buen boletín, una buena información a cada persona de que podemos eliminar el mosquito.

Moderadora

Ok.

P#7FB

De varias maneras.

Actividad #8 – Mosquitos modificados genéticamente

Moderadora

Ok. Y entonces, pasamos a la última. Llegamos a la última. [aplauden y hacen chiste de que aún quedan mosquitos] Otro más, el último. Mosquitos modificados genéticamente. Se llevan mosquitos macho para que se unan con mosquitos hembras del ambiente. Los mosquitos macho modificados genéticamente se reproducen con las hembras del ambiente y pasan un gen a sus crías que impide que las larvas y las pupas se desarrollen normalmente.

P#6JC

Eso es bueno también.

Moderadora

Así, estas mueren antes de convertirse en mosquitos adultos. Se pasa el macho que le pusieron un gen, para que cuando se una con la hembra, los que nacen, nacen con ese gen defectuoso y no llegan a ser mosquitos adultos.

P#4EST

Se mueren…

P#6JC

Natimuertos.

Moderadora

Se quedan en el camino.

P#6JC

Natimuertos.

Moderadora

De… se pueden morir de mosquitos, de larva o de pupa.

P#6JC

Exacto, natimuerto.

Moderadora

Eso es, natimuertos, así mismo. Los mosquitos deben liberarse varias veces a la semana a lo largo del tiempo para mantener bajas las poblaciones de mosquitos.

P#7FB

Lo mismo que decía… [supongo que se refiere a los mosquitos macho con Wolbachia]

Moderadora

Lo mosquitos macho generados no pican ni transmiten enfermedades.

P#6JC

Ah, qué bueno.

Moderadora

Estos mosquitos se deben liberar varias veces a la semana a lo largo del tiempo y en grandes cantidades para mantener bajas las poblaciones de mosquitos *Aedes aegypti* solamente. Una vez los mosquitos modificados genéticamente *Aedes aegypti*, dejan de ser liberados en un área, la población de mosquitos aumentará de nuevo.

P#6JC

Claro.

Moderadora

Los mosquitos modificados genéticamente han sido evaluados en diferentes países incluyendo las Islas Caimán, P#6JC y Panamá. Al presente no se han hecho estudios de los mosquitos modificados genéticamente en los Estados Unidos.

Pregunta 2- ¿Es esta una actividad nueva para usted o es algo que ya había escuchado antes? Si la escuchó, ¿dónde la escuchó?

Moderadora

¿Habían escuchado de eso anteriormente?

P#5JC

No.

Moderadora

¿No?

P#7FB

Este, ¿Cuánto de vida tiene el mosquito, hembra o macho?

P#9GL

¿Cuánto es la vida de…?

Moderadora

En la vida… en el ambiente normal, pues hasta que uno le dé un bofetón… [risas]

P#9GL

Eso iba a decir yo.

Moderadora

O le eche el “spray” y lo mate. Porque si le hecha el Raid y no lo pisa vuelve y sale volando a los quince minutos.

P#6JC

Se recompone. Ellos como que se activan.

P#7FB

Pero, el tiempo de vida natural, así…

Moderadora

Entre semanas… pues semanas y días. En el ambiente natural pueden… del ambiente urbano pueden durar poco, pero en el ambiente natural de… pueden durar más.

P#6JC

En el campo…

P#9GL

Pero mira, esos países que tú estás mencionando, son países que tienen selva, extensa. Y por eso es que ponen, la Isla Caimán, P#6JC y Panamá, por ahí para abajo, son bosque, selva.

P#7FB

Lo que pasa es que el mosquito, los insectos, se han convertido en un negocio. Para los insecticidas, no los van a combatir así tan fácil.

Moderadora

Bueno, hasta donde yo sé es una…

P#9GL

Eso es una fábrica de Real Kill…

Moderadora

Hasta donde yo sé….

P#7FB

No, yo no soy, los grandes millonarios.

Moderadora

Escúchenme. Hasta donde yo sé, es el… es en comunidades. En comunidades en P#6JC. No es en la selva que se han probado, es en comunidades.

P#6JC

En un área.

Moderadora

En un área urbana.

P#9GL

Cuando yo muchacho, cuando yo me crie por Baldorioty, Montañez, San Antonio… todo eso era caña. Cuando venía el corte de caña, el azúcar. Lo que ella vio en la casa de ella, el…

Moderadora

El cien pies.

P#9GL

El ciempiés. Dicen los capataces y los que iban a la casa de mi papa, a la tienda. Es de calor, de sitios este… entonces, el mosquito se cría en la caña con la cuestión de la azúcar. Claro como sacaban la caña e iba a la casa. Pues se introducía entonces a las comunidades. Pero el mosquito y el ciempiés son de…

P#6JC

Y arañas peludas también.

P#7FB

De la humedad…

P#9GL

Mira, y yo por mi casa…

Moderadora

Bueno, lo que pasa es que son, perdone que lo interrumpa, lo que pasa es que son, insectos que son del Caribe. De temperaturas calientes. Así que no hay que sembrarlos ni nada, ellos solitos ahí porque eso es su ambiente.

P#9GL

Pero en mi casa aquí, usted sabe lo que es… el alacrán.

Moderadora

Sí, claro.

P#9GL

Pues yo en mi casa conseguí, cogí un alacrán. En la próxima reunión voy a traerlo.

Moderadora

Ay no…

P#9GL

Mire… [cuenta la historia de cómo atrapó el alacrán, nada relevante a lo que se está hablando]

Moderadora

Está preservado.

Pregunta 3- ¿Cree que esta actividad reduciría el número de mosquitos en su comunidad? ¿Por qué?

Moderadora

Ok. Les pregunto, porque ya nos quedan poquitas preguntas. ¿Cree usted que esta actividad puede reducir el número de mosquitos en su comunidad?

P#5JC

Sí.

P#9GL

Bueno sí.

P#6JC

Sí.

Moderadora

Sí.

Pregunta 3a- ¿Qué beneficios o ventajas tiene esta actividad para usted?

Moderadora

¿Qué beneficios o ventajas tiene esta actividad para ustedes?

P#9GL

Bueno, que… para mí, yo, mi opinión, yo he aprendido cosas que no había…

Moderadora

No, no, no. La actividad no… Acuérdese, los mosquitos genéticamente modificados. [a la vez, P#8ST le explica lo mismo a P#9GL]

P#6JC

Qué hacen en la comunidad.

Moderadora

Vamos a tener que quitar la palabra actividad.

P#8ST

Sí, es que confunde.

Moderadora

El método. Porque confunde.

P#6JC

Eso mismo.

Pregunta 3b- ¿Qué desventajas o dificultades le ve a esta actividad? ¿De qué forma se podrían solucionar esas dificultades?

[Se pregunta en otro lado]

Pregunta 4- ¿Cuán posible es realizar esta actividad en su comunidad para reducir el número de mosquitos? ¿Por qué?

Moderadora

Usar método. Que… este método, ¿usted cree que se pueda hacer en su comunidad?

P#5JC

Mira, y ese método, ¿es muy sencillo o es…?

P#4EST

Eso es en laboratorio…

Moderadora

Eso tiene que hacerse en laboratorio y lo tiene que hacer el gobierno. O sea, no puede hacerlo una persona independiente.

P#6JC

No. Exacto. El… se puede hacer siempre y cuando uno tenga el respaldo del… como usted dice, del gobierno, de los laboratorios, porque de otra manera, el método no se puede hacer si no hay esa… esas poblaciones de mosquitos que continuamente estén accesibles para mantener controlado el ambiente.

Moderadora

Exacto. Estos son métodos que solamente el gobierno los puede hacer.

P#6JC

Claro.

Moderadora

Verdad.

P#7FB

Si lo autoriza el gobierno…

Moderadora

Solamente, el gobierno los puede hacer. No los puede hacer una persona individual, porque una persona individual no puede comprar esos mosquitos. No puede hacerlo. Ni los puede tirar al ambiente sin unas de Estados Unidos, de la FDA, de la EPA. Verdad, no se pueden hacer.

P#7FB

Recuerden que los insecticidas los venden las grandes empresas.

Moderadora

Esto depende de que Estados Unidos reglamente…. Esto depende de que Estados Unidos establezca unas reglas para uso, sea por estudio primero, y luego para uso de las comunidades. Y así por eso lo pones ahí. Estamos siendo bien honestos. Esto todavía no se ha probado en los Estados Unidos. En el anterior dijimos, el de los mosquitos macho y hembras, dijimos que no hay reglas todavía en Estados Unidos para hacer eso, en donde lo único que hay es reglas establecidas es para los machos solos con Wolbachia. Por eso se han hecho en California, en Miami…

P#7FB

Los insecticidas son de las grandes empresas. Ellos son los que aguantan. Eso no va a venir tan fácil.

Moderadora

Ok. ¿Qué desventajas ustedes le ven a este método?

P#5JC

¿Desventajas?

Moderadora

¿Ustedes le ven alguna desventaja a este método?

P#7FB

Algo beneficioso para nosotros.

P#5JC

No.

P#6JC

Sí. ¿Desventaja? Sí, pues…

Moderadora

¿Cuál?

P#6JC

Si no hay… si no hay, el… las poblaciones disponibles, ¿cómo se va a hacer el programa? Si no la hay.

Moderadora

Exacto. ¿Y cómo se solucionaría eso?

P#6JC

Eh, precisamente es el gobierno el que se tiene que comprometer.

Moderadora

Ok. ¿Es posible realizar esta actividad en su comunidad? ¿Ustedes creen que sería posible?

P#9GL

¿El qué?

Moderadora

Hacer esta actividad en su comunidad.

P#5JC

Sí.

P#7FB

La promoción, sí.

P#9GL

Puede que sí.

P#7FB

Conseguir los mosquitos y eso, está distante, porque hay que tener autorización de Estados Unidos y autorización de este gobierno, y se van a oponer las grandes fábricas de insecticidas. Se van a oponer, porque eso es un negocio que les deja dinero. El mosquito es un negocio para los comerciantes que venden insecticidas. Por eso es que va a ser bien difícil que el gobierno lo apruebe. Es así. Son bases.

P#9GL

Yo estoy de acuerdo con lo que él dice, porque aquí hay diez personas. De aquí de GL… el compai es de GL pero vive en ST, éste vive en ST. ¿De dónde tú eres?

P#7FB

Cerca de ST…

P#9GL

Ve. Imagínese. Ustedes son de JC. Pues… entonces…

P#6JC

Frente a GL.

P#9GL

¿Frente aquí? Lo que quiero decir es que, son muy pocos los que han venido aquí, para entonces uno hablarle de las diferentes… capítulos.

P#7FB

Pero es que hay que empezar desde abajo no podemos empezar desde arriba, porque nos vamos a reventar.

P#2EST

Para eso son las charlas que se están dando, para llegar a acuerdo, para que todo el mundo esté de acuerdo, para que entonces, pueda ser aprobado.

Moderadora

Exactamente.

Pregunta 5- ¿Apoya usted esta actividad en su comunidad? ¿Por qué? (*Preguntar a cada participante del grupo de discusión*)

Moderadora

¿Apoyaría usted esta actividad en su comunidad?

P#9GL

Sí.

P#6JC

Sí.

Moderadora

¿La apoyaría? [Participantes asienten con la cabeza. Anotadoras tienen en sus notas que todos los participantes apoyan la actividad.]

P#8ST

Sí, claro.

Pregunta 5a- ¿Piensa que su comunidad apoyaría esta actividad? Sí, No, ¿Por qué?

Moderadora

¿Y cree usted que su comunidad apoyaría el uso de esta actividad?

P#2EST

Sí.

P#7FB

Sí.

P#6JC

Sí.

P#5JC

Sí.

Pregunta 6- ¿Qué otra información necesitaría para entender mejor esta actividad?

Moderadora

¿Qué información necesitarían para entender mejor esta… este método?

P#2EST

Pues, lo que se dio aquí ahora mismo.

P#6JC

Información ilustrativa, e información… este, didáctica también.

Moderadora

Usted tenía algo que decir, que lo dejé ahí en suspenso, porque…

P#7FB

Sí la información debe llegar casa por casa.

Moderadora

Casa por casa.

P#7FB

No solamente… una reunión.

Moderadora

Un boletín.

P#7FB

Un boletín.

Moderadora

Ok. Ahora, yo les hago una pregunta. Ustedes están diciendo aquí que, si le gente no viene, usted está diciendo que la gente no viene, que mira que poquitos somos, la gente no viene. ¿Qué ustedes podrían hacer para que la gente viniera?

P#9GL

Esta es la segunda llamada, porque en la primera no vino nadie. Esta es la segunda, llegamos diez. Y cuidado, porque estamos en GL.

Moderadora

Porque vamos a hacer una actividad para el grupo de comunidades de GL.

P#9GL

Sí…

Moderadora

Que incluye todas estas comunidades. Y vamos a explicar el proyecto. ¿Qué ustedes van a hacer para traer gente aquí?

P#9GL

Pues yo le sugerí a la que habló conmigo, en la iglesia… se pueden poner…

P#5JC

Para eliminar los mosquitos.

P#6JC

Claro.

Moderadora

Hay todas… ¿hay actividad pautada para este lugar o ya fue? [alguien de CDC contesta, pero no se entiende] ¿Para la próxima semana habría que bregar? Ok. Y entonces, había una… ese fue el último método. De todos estos métodos, ¿cuál es el más que a usted le gusta? Número uno (*P#1EST*).

P#1EST

Las trampitas.

Moderadora

Las trampas. ¿Y a usted?

P#2EST

Las trampas y el…

Moderadora

Las trampas, ¿y qué?

P#2EST

El método ese de los mosquitos. Los tres que pasaron último.

Moderadora

¿Los últimos tres le gustaron?

P#2EST

Y echarle a…

Moderadora

Larvicida. O sea, que a usted le gustó casi todo.

P#2EST

Casi todo.

Moderadora

¿Y a usted, específicamente, el más pero el más que le gustó?

P#3EST

Estos tres últimos.

P#7FB

Eso no va… [dice muy bajito]

Moderadora

Estos tres últimos. ¿Y esos fueron los más que le gustaron a usted?

P#2EST

Aján. Sí.

Moderadora

¿Y a usted? ¿Qué fue el más que le gustó?

P#4EST

El último también.

Moderadora

¿El último o los tres últimos?

P#4EST

Los tres últimos.

Moderadora

¿Y hay alguno de esos tres últimos en específico que le guste más?

P#4EST

Esto como que es más o menos…

P#6JC

Son casi lo mismo…

Moderadora

Ok. ¿Y a usted?

P#5JC

Lo más que me gusta es este.

Moderadora

El genéticamente modificado.

P#5JC

El genéticamente modificado.

Moderadora

¿Y usted?

P#6JC

A mí me gustaron esos métodos, pero creo que podría ser más factible, más práctico, el del camión y la trampa.

Moderadora

Camión y trampa.

P#4EST

Eso sí, son más reales….

Moderadora

El larvicida de camión y trampa. ¿Y a usted? ¿Cuál le gustó más?

P#7FB

El larvicida de… ¿semilla es?

Moderadora

De gránulos.

P#7FB

De gránulos. Porque según usted, pues yo lo puedo conseguir más fácil que los demás.

Moderadora

Ok. Porque se consigue ya comercialmente.

P#7FB

Claro, lo puedo comprar yo, lo puede comprar uno de nosotros. No como el de la trampa que todavía le falta… faltaría conseguir la pega. No como los genéticamente que no está aprobado por Estados Unidos.

P#6JC

Sí, eso tardaría…

P#7FB

Hembra o macho. Eso todavía está en “stop”.

Moderadora

Ok. ¿Y usted? ¿Cuál es el que más le gusta?

P#9GL

El de… con la señora, el de las trampas y…

P#6JC

El camión.

P#9GL

Y el camión. El camión me es más efectivo.

Moderadora

De larvicida.

P#9GL

Porque se va a beneficiar el que no vino aquí. Y va a decir, ‘¿ese ruido, y eso?’ Pues eso es para matar los mosquitos que tú no quieres… en tu casa. [risas]

Moderadora

Ok. ¿Y usted? ¿Cuál más… cuál fue el que más le gusto?

P#8ST

El larvicida. Y el de la trampa.

Moderadora

También, larvicida y trampa. ¿Esos fueron los que más les gustó? Había unas preguntas que ellos hicieron, que yo dije que iba a contestar…

Sue

Faltan dos preguntas Moderadora.

Moderadora

¿Cuáles dos?

Sue

La diez y la once.

Moderadora

Ah, me faltaron dos preguntas caramba.

**Parte 3- Cierre de sesión**

Pregunta 10- ¿En quién de su comunidad confiarían ustedes para hablar sobre estas actividades?

Moderadora

Ok. Para todos estos métodos. Para todos estos métodos, ¿en quién de su comunidad confiarían ustedes para hablar sobre estas actividades? Para hablar sobre estos métodos. ¿En quién confiarían? Para que diga a usted el mensaje y le explique estos métodos.

P#9GL

Pero es que si uno viene aquí para que se empapen de los cinco métodos que hay aquí…

Moderadora

No, son los nueves.

P#9GL

Pues nueve. Este… uno lo capta todo para explicárselo a la persona…

Moderadora

Sí, pero ¿en quién de la comunidad? Un médico, el párroco, el pastor, un científico…

P#7FB

Un presidente de barrio.

Moderadora

Un presidente de barrio.

P#1EST

A _________ (nombre), el presidente de la…

P#6JC

Y un científico. Para mi… Yo…

Moderadora

Un científico para usted. Para usted un líder de la comunidad.

P#2EST

El líder de la comunidad y que la información llegue… no como hoy, que me llevaron el papel hoy, para hoy mismo.

Moderadora

Ah…

P#2EST

Porque no tenemos … si es de hoy para hoy, no tenemos el tiempo de buscar la gente a que vengan aquí.

P#4EST

Eso es verdad.

Moderadora

¿Y para usted?

P#4EST

También, lo mismo. El de comunidad.

Moderadora

El líder de comunidad. Ok.

Pregunta 11- ¿Cuál sería la mejor manera de hablar a su comunidad sobre estas actividades?

Moderadora

Y entonces, ¿Cuál sería la mejor manera de hablar a su comunidad sobre estas actividades? Usted mencionó boletines. Ya lo habían dicho. Él mencionó boletines. Él mencionó más actividades con… de comunidad. ¿Qué más? Ella mencionó muchas.

P#6JC

Pues yo estoy… exacto.

Moderadora

Ella mencionó muchas al principio.

P#9GL

De lo que él dice aquí de boletín. Y a la gente… en el buzón. Es más fácil…

P#7FB

Lo van a leer.

Moderadora

¿Y la gente lo leería? La gente no quiere leer nada.

P#9GL

Pues, ese es el detalle también…

P#5JC

Se le deja así.

Moderadora

¿Se le deja así?

P#9GL

Es que los buzones se les llenan de tanta cuenta, que ya no les cabe ese papel. [risas]

P#6JC

Y vuelvo y le digo, es tener… disponible en estos sitios donde hay gente que va, no una vez… yo salgo a veces a la repostería, hay personas que van dos y tres veces al día. Se le acabó algo… ay espérate. Ahí es que paran.

P#9GL

Mira, yo le sugerí a la que me llamo a casa, que me llevo los papeles a casa… yo le dije que en el parque hay un boletín *board*, que todas las actividades que se van a hacer en el parque la gente la leen.

P#6JC

Ve, hay que buscar todos los métodos.

P#9GL

En la panadería que se le pide permiso a _______(nombre), que es para el beneficio de ellos también. Lo ven allí.

P#6JC

Claro.

P#9GL

Aquí en la iglesia, en el quiosco.

P#6JC

También.

P#9GL

Todos los de la comunidad… en la iglesia la gente que va a comer relleno como yo, hago como ellos, como relleno y leo lo que hay.

P#6JC

En la casita ________(nombre).

Moderadora

Ella dijo que se tenían que usar muchos métodos a la misma vez.

P#6JC

Es correcto.

Moderadora

Así es que, esta que está aquí, me está ajorando porque vamos para San Juan. Bueno, muchas gracias por haber estado aquí. Me alegra que hayan venido, han sido un grupo excelente, han participado y han dado sus opiniones.

*****Fin del audio*****
