# Supplementary material for: Acceptability of emergent Aedes aegypti vector control methods in Ponce, Puerto Rico: A qualitative assessment
Source: PLOS Glob Public Health. 2024 Mar 6;4(3):e0002744. doi: 10.1371/journal.pgph.0002744 (PMC10917327; doi:10.1371/journal.pgph.0002744)
Supplement: S2 Appendix — (ZIP) [file pgph.0002744.s002.zip › S2B_ Appendix.docx]

**S2 Appendix. Anonymized Transcripts of Group Discussions (Spanish)**

**COPA Grupo de Discusión con Líderes y Residentes sobre Actividades de Control de Vectores en los Clústeres PA, MA y JD**

Fecha y hora- 24 de abril del 2018; 7pm

Lugar- Centro Comunal de LD

Moderadora - Coral Rosado

Anotadores- Mariely y José

Transcriptor- Sue Ramos

Participante #1- LD (H) - P#1LD

Participante #2- LD (H) – P#2LD

Participante #3- TI (M) – P#3TI

Participante #4- EG (H) – P#4EG

Introducción- Se comenzó leyendo el consentimiento informado con los participantes, y se les preguntó a los participantes si tenían preguntas sobre lo leído. Nadie tuvo preguntas. Todos aceptaron a que se grabara la discusión y participar en el grupo. Luego se les leyó la introducción de la guía de preguntas y se leyeron las reglas del grupo y se le asignó número a cada participante

Parte 1- Conocimiento sobre enfermedades transmitidas por mosquitos

Moderadora

Por favor, para propósitos de saber las comunidades representadas, les voy a pedir que digan su número y la comunidad que representa. Si puede comenzar usted.

P#1LD

Sí. Mi nombre es [participante dice su nombre] y…

Moderadora

No tiene que decir su nombre. Sólo su número y… sí.

P#1LD

Mi número… ah, es que yo no los conozco y es la primera que me ven… me ven en la calle o algo, pues…

P#2LD

Te voy a conocer por número.

P#1LD

Después me ven en la calle y me van a decir, ‘número uno (*P#1LD*)’. [risas]

Moderadora

Número uno, sí.

P#1LD

Pues, resido hace 46 años aquí en la Segunda LD. Estuve a cargo del centro comunal por 27 años. Y aquí estamos…

Moderadora

Ok. De LD. ¿Y usted?

P#2LD

Soy aquí de LD. Soy encargado ahora de centro. Soy el sucesor del número uno (*P#1LD*). Por eso somos el uno y el dos. [risas] Y llevo aquí viviendo casi toda mi vida.

Moderadora

Ah. Bien, que bien, ah pues conocen bien su comunidad. ¡Qué bueno! ¿Y por acá?

P#3TI

Represento a urbanización TI. Eh ya hemos estado pasando por esta experiencia y me ha gustado. Y gracias por la invitación.

Moderadora

Qué bueno. Ay gracias a usted por estar aquí. ¿Y él es su hijo?

P#3TI

Él es mi hijo.

P#4EG

Soy el administrador de EG.

Moderadora

Ok. Chévere.

Pregunta 1- ¿Qué ha escuchado sobre el dengue, Zika y Chikunguña?

Moderadora

Bueno, pues para ir comenzando… sé que se están integrando otros, pero van a hacer un grupo, verdad, allá, para estar, eh… equitativos, cinco y cinco. Eh… voy a hacerles unas preguntitas generales. ¿Qué ha escuchado sobre el dengue, Zika y el Chikunguña? ¿Qué ha escuchado?

P#2LD

Eso es a través de un mosquito.

Moderadora

Ok. Número dos (*P#2LD*) …

P#2LD

Eso lo transmite un mosquito, el *Aedes aegypti*.

Moderadora

Eso lo transmite un mosquito, el *Aedes aegypti*.

P#2LD

[no se entiende el audio]… el mismo mosquito. Y los síntomas son casi parecidos, las enfermedades.

Moderadora

Sí, sí, parecido. ¿Algo más que hay…?

P#2LD

Y el mosquito se cría en agua estancada.

Moderadora

Ok. Sí, sí, así mismo es. ¿Algo adicional que hayan escuchado?

P#1LD

Bueno, el Zika, tengo entendido, es una enfermedad, el Chikunguña también, que es transmitida a través del mosquito *Aedes aegypti*. Una variedad, pues, del mosquito. Así que como dijo el compañero, este… en la manera que se cría el mosquito es a través de aguas estancadas, sea en gomas, en áreas… en las residencias que muchas veces, las personas no se percatan que ese es el centro de crianza de esos mosquitos.

Moderadora

Aján, sí. Cierto, cierto. Y ahí vamos en eso también de que no se percatan.

Pregunta 1a- ¿Cómo se transmiten?

[se contestó más arriba]

Pregunta 1b- ¿Cuán peligrosas son?

Moderadora

¿Y cuán peligrosas son estas enfermedades? Que ustedes sepan.

P#2LD

Dele número tres (*P#3TI*).

P#3TI

Son enfermedades que pueden ser devastadoras en la salud de algunas personas que tengan… eh, que estén comprometidas con otras condiciones de salud. Eh, tenemos, pues, también, por ejemplo, pues, las embarazadas. Que, eh, ahora mismo, este, este, pues hay niños naciendo que son de la cepa, víctimas de lo que fue el Zika. Y pues este… que son niñitos que les tenemos que prestar mucha atención, por el cien número de condiciones que traen a consecuencia de esto.

Moderadora

Sí, cierto, cierto. ¿Alguna otra… ajá… opinión? Sí.

P#4EG

Eh, son enfermedades que por desconocimiento y falta de información la gente no… la confunden con un tipo de influenza sin mayor… o catarro, sin mayores consecuencias y cuando se percatan pues… como dice la compañera es bien devastadora, que inclusive puede afectar, en el caso de… personas con… en el proceso de tener hijos. Verdad. Este… traer unas consecuencias mayores.

Moderadora

Sí.

P#2LD

Esto puede llegar hasta la muerte. En nuestra…ha habido muchas muertes a causa de estas enfermedades en Puerto Rico.

Moderadora

Sí. ¿Cómo cuáles en específico, de las tres… o las tres?

P#2LD

Las tres en conjunto más… [no se entiende lo que dice en el audio].

P#4EG

Eh, ok. Una de las… una de las consciencias que debemos tener es, en el tipo de ambiente que nosotros vivimos. Que nosotros vivimos en un área subtropical, que tiende a provocar, si no tenemos la atención y la fumigación adecuada, a que se produzcan, además de estas enfermedades, otras más que no vamos a entrar en esos detalles, pero por eso tenemos que ser responsables de nosotros transmitirle a nuestro vecindario cuál es a mejor manera para evitar que estas enfermedades nos ataquen.

Moderadora

Claro. Sí, sí, sí. Muy bueno, muy bueno. A eso vamos más adelante, para estrategias, ¿no? De cómo llevar a cabo el mensaje…

Parte 2- Reacciones a las actividades de control de vectores

Moderadora

Y bueno, como esta es la introducción para comenzar, yo les voy a mostrar un video sobre entonces… el mosquito y el ciclo de vida del *Aedes aegypti*. Esta explicación les va a ayudar a entender mejores cosas sobre otras actividades que vamos a hablar en el grupo, sob… y para el control de mosquitos. Voy a comenzar. Aquí tienen el mosquito *Aedes aegypti*. Y el mosquito *Aedes aegypti* macho no pica. ¿Ok? Es la hembra. La hembra pica una persona para obtener su sangre y así producir sus huevos. Está llenando su abdomen de sangre al picar. Y entonces luego verdad, que sus huevos están listo los va a poner en las paredes de cualquier envase con agua acumulada, como habían dicho, dentro o fuera del hogar. Y estos huevos pueden durar hasta ocho meses, en lugares secos, pegados a las paredes de un envase. Cuando los huevos se sumergen en agua, sale la larva. Y las larvas viven en el agua donde se alimentan y se convierten en pupas, que vamos a ver más adelante, en alrededor de cinco días. Ellas se van a alimentar de materia orgánica en el agua. [no se entiende lo que dice] Y aquí pueden ver cuando se convierte en pupas. Y de dos a tres días, estas pupas… de dos a tres días se van a convertir en los mosquitos adultos. Y se toma de siete a diez días desde que comienza en un huevo de mosquito, verdad, y se convierte en un mosquito adulto. Alrededor de una semana, de siete a diez días, para volver al ciclo de vida nuevamente. Bueno. Eh… ahora, pues les voy a explicar que se están desarrollando muchas formas de reducir el número de mosquitos en el ambiente. Y algunas acciones pueden ser realizadas por los residentes y las comunidades, y otras pueden ser realizadas por profesionales de control de mosquitos o el gobierno. Les voy a mostrar unos dibujos que describen las actividades que se podrían realizar y cómo podrían ayudar. Y luego de esos dibujos y explicaciones voy a hacerles unas preguntas para obtener sus opiniones de esa actividad.

Actividad #1 – Reducción de fuentes de mosquitos

Moderadora

Ok. Pues vamos a la primera ilustración. Se llama reducción de fuentes de mosquitos. Vamos a ver si ustedes han escuchado sobre esta actividad y ver como siguen. La reducción de fuentes es la eliminación de lugares donde los mosquitos ponen sus huevos. El municipio y su comunidad trabajarían juntos para eliminar, vaciar y recoger los envases con agua acumulada que puede hacer criaderos de mosquitos en áreas públicas. Dentro de su hogar y patio, usted cubriría, vaciaría o eliminaría los envases que acumulan agua como los tiestos, las latas, gomas y desagües.

Pregunta 2- ¿Es esta una actividad nueva para usted o es algo que ya había escuchado antes? Si la escuchó, ¿dónde la escuchó?

Moderadora

¿Esta actividad es nueva para usted o es algo que ya había escuchado antes?

P#1LD

Anteriormente había escuchado sobre… sí.

Moderadora

Ok. ¿Y dónde la había escuchado?

P#1LD

La he escuchado en la radio, la televisión, y aquí mismo en… en una vez que estuvimos el año pasado, que yo estuve presente, aquí había escuchado. [el participante se refiere al grupo focal que se invalidó de Pastillo, recuerdo que él estuvo presente] Y yo lo he leído también en panfletos. He ido a diferentes oficinas de médicos y eso y a veces tienen un panfleto.

Moderadora

Ok. No es nueva para usted. Ok. ¿Y para ustedes?

P#2LD

Tampoco para mí es nueva.

Moderadora

No es nueva. Ya la había escuchado.

P#2LD

La había escuchado, también estuve aquí el año pasado en una de… entrevista que me hicieron también.

Moderadora

Que bueno. Sí. Ok. ¿Y usted?

P#3TI

Sí, ya se ha convertido como una rutina en mi vida. Porque todos…todas las semanas yo verifico el patio, sacamos todo, verdad, verifico que no haya nada, el agua de los perros todos los días la cambio, le lavo el envase.

Moderadora

Oh que bien.

P#3TI

Para que no cree nada de eso. Y se ha convertido ya en parte… incluso en la comunidad, pues, con él y con mi otro hijo, limpiamos las áreas verdes del centro comunal, la placita. Y los vecinos que tengan el patio pelú, él también va para… y es una manera de prevención.

Moderadora

¿Y por acá? ¿Lo había escuchado?

P#4EG

Sí, ya… Sí, lo había escuchado. A través de los medios y en panfletos.

Pregunta 3- ¿Cree que esta actividad reduciría el número de mosquitos en su comunidad? ¿Por qué?

Moderadora

Ok. ¿Cree que esta actividad…? ¿Creen ustedes que reduciría el número de mosquitos en su comunidad?

P#4EG

Yo entiendo que sí.

Moderadora

¿Sí? Número cuatro (*P#4EG*), sí. ¿Por qué reduciría el número…?

P#4EG

Bueno, yo entiendo que al quitar los focos o el medio donde puedan ellos desarrollarse, este… les estas qui… le estas eliminando la fuente principal para que ellos se multipliquen. Sin embargo, probablemente haya otras medidas, que necesitan ayuda en aquellos lugares donde no todo el mundo participa, en… esos… porque entiendo, que esos son unos datos elementales. Esa es la forma más elemental de tú evitar que… propagar el mosquito.

Moderadora

Sí. Sí. Eso es así. ¿Alguien más creen que reduciría la cantidad de mosquitos en su comunidad?

P#1LD

Bueno, sí, yo creo que sí. Lo único que el gobierno debe ayudar más a las comunidades a desarrollar esos programas. Porque hace años que yo no veo la máquina de fumigar por ahí.

P#2LD

Pero yo no estoy de acuerdo con la fumigación.

Moderadora

Que no está de acuerdo con la fumigación. Ok.

P#2LD

La fumigación… este, mata mosquitos, pero mata otros insectos que nos hacen falta, como la abeja. O sea, que eso es un arma de doble filo. Que hay que ver dónde se fumiga. Y también afecta, pues a la gente de la comunidad que están asmáticos. Hay gente que… con enfermedades. Y el gobierno no avisa cuándo va a fumigar. O sea, si ellos avisaran, ‘mira, tal día vamos a fumigar a tal hora’, pues uno se preparaba. Pero ellos no avisan, ellos fumigan y por ahí… a repartir suerte.

Pregunta 3a- ¿Qué beneficios o ventajas tiene esta actividad para usted?

Moderadora

Lo están discutiendo entre líneas, la próxima pregunta, sobre esta actividad, les quiero preguntar, ¿Qué beneficios o ventajas tiene esta actividad para ustedes? ¿O qué beneficios y ventajas le ven a esta actividad?

P#4EG

¿Esta actividad? Yo entiendo que es la actividad fundamental para evitar la propagación del mosquito. Entiendo que si nosotros comprometemos a nuestro vecindario a que se cumpla y veremos… por eso no vamos a necesitar del gobierno, y entiendo que no debemos necesitar de la asperjación, ni de la fumigación. ¿Por qué? Porque, pues, como dice el compañero, eso puede conllevar la muerte de otros… y quizás la contaminación de uno mismo. Pero entiendo, que esa es la parte más esencial.

Moderadora

Ok. Ok.

Pregunta 3b- ¿Qué desventajas o dificultades le ve a esta actividad? ¿De qué forma se podrían P#4EGucionar esas dificultades?

Moderadora

Y ahora lo contrario, ¿Qué desventajas o dificultades le ven a esta actividad?

P#4EG

Yo no le veo ninguna desventaja. Yo lo que entiendo es que… la gente, nosotros, somos vagos, muchas veces esperamos, y que el gobierno nos lo acuerde. Entiendo, nosotros debemos crear una agenda para nosotros comenzar a comunicarlo, este… y creo que esta es una manera de tener una herramienta para nosotros ilustrarnos del asunto, verdad, de este tema. De manera tal, que lo podamos comunicar y entonces, crear unos grupos en nuestra comunidad para que le den seguimiento a esas tareas. Como en el caso mío, quizás diferente al caso de ellos, yo no vivo en la comunidad. O sea, yo voy y trabajo… lo que sí, probablemente, lo que tengo que hacer es… exacto, citarlo y darle seguimiento para que se comuniquen entre sus vecinos.

Moderadora

Ok. Que bien. Que bien. ¿Y alguna otra desventaja o dificultad que vean de esta actividad? Ok. Sí, porque la otra pregunta era, ¿de qué formas se podrían P#4EGucionar esas dificultades? Pero usted ya dio la recomendación de una agenda para entonces darle seguimiento a los residentes.

P#4EG

Quizás con una pregunta vamos a arreglar… y esto no es todo el año, en qué época básicamente nosotros debemos prestar atención para nosotros comenzar con ese protocolo, de manera tal que podamos activar el vecindario en tomar las medidas de reducción de fuentes.

Moderadora

Aján. Sí, sí. Chévere.

Pregunta 4- ¿Cuán posible es realizar esta actividad en su comunidad para reducir el número de mosquitos? ¿Por qué?

Moderadora

Ok. La próxima pregunta sería, ¿Cuán posible es realizar esta actividad en su comunidad para reducir el número de mosquitos? ¿Cuán posible o viable ustedes lo ven? ¿Qué dice…?

P#2LD

P#4EGamente hay que comunicarlo a la comunidad.

Moderadora

Ok. Que lo ve posible.

P#2LD

Es posible.

Moderadora

Ok. Ok. ¿Y por qué es posible? ¿Qué lo facilita?

P#2LD

Es que todos estamos en el mismo barco. Todos cuidamos por nuestra salud. Tú sabes, y todos estamos conscientes que esas enfermedades pueden… sabes, son dañinas. Y evitando el mosquito es que evitamos que nos enfermemos nosotros. O sea que, la gente tiene que ser consciente, eliminando el agua estancada y eliminando también este, el… como se dice, elimina la enfermedad.

Moderadora

Ok. Ok. ¿Y usted lo ve también posible aquí en LD? ¿O piensa distinto?

P#1LD

Sí como número dos (*P#2LD*), yo lo veo posible. Lo que pasa es que esta comunidad segunda LD se ha convertido en una comunidad de personas de la tercera edad mayormente.

Moderadora

Sí, claro.

P#1LD

Y… como el día de hoy, yo cité muchas personas, pero me dijeron que no salen de noche, tienen miedo a salir de noche. Y así mismito es, uno invita a una actividad, y pues las personas mayores… no que no estén interesados, es que no quieren porque tienen temor a salir y hacer otras…. Pero… se puede, se puede intentar con… Porque hay muchos jóvenes. Tenemos una escuela aquí al lado que se puede hablar con la directora y ellos nos pueden ayudar. Por ejemplo, nosotros podemos hacer unos panfletos y entre ellos pueden repartirlo a la comunidad y darle orientación personalmente, y visitar casa por casa. Viendo si hay, este…

P#2LD

El agua estancada.

Moderadora

Criaderos.

P#1LD

Sí, cubierto, las fuentes, las gomas y todo eso, en los patios de las casas. Sí, y así pues, podemos evitar… ajá…

Moderadora

Ok, sí. Esos criaderos con esa estrategia. ¿Y usted, ve posible esta actividad en su comunidad?

P#3TI

Sí. Yo la veo posible. Y más cuando tenemos cuerpos de aguas cercanos a nuestras viviendas. Por ejemplo, nosotros tenemos como una quebrada que pasa, este… por una de las calles, yo vivo en la calle cuatro y eso desagua por los puentecitos que uno conecta con la cantera. Pues entonces, esos son focos. Por ende, nosotros tenemos que llevar el comunicado a las personas de que estén pendientes. Y por lo menos, en TI la gente se aplica, verdad, mantenemos áreas limpias y velar por que no se tenga agua estancada… yo creo que tenemos un buen mensaje.

Moderadora

Ok. ¡Qué bueno!

Pregunta 5- ¿Apoya usted esta actividad en su comunidad? ¿Por qué? (*Preguntar a cada participante del grupo de discusión*)

Moderadora

Y… E individualmente, ¿apoya usted esta actividad en su comunidad? Si me pueden decir su número y su contestación. ¿Usted apoyaría esta actividad en su comunidad?

P#1LD

La apoyo (la actividad) rotundamente.

Moderadora

Lo apoya.

P#1LD

Es una actividad que es para el beneficio de todo el mundo.

Moderadora

¿Y por acá número dos (*P#2LD*)? ¿Apoyaría esta actividad?

P#2LD

100%.

Moderadora

100%. Ok. ¿Y número tres (*P#3TI*) apoyaría esta actividad en su comunidad?

P#3TI

100%.

Moderadora

Ok. ¿Y número cuatro (*P#4EG*)?

P#4EG

Sí, la apoyo.

Moderadora

Ok. Ok. Y ahora les pregunto por qué. ¿Por qué la apoyarían?

P#1LD

Pues como contesté anteriormente.

Moderadora

Sí, sí, sí.

P#1LD

Es para el beneficio de todos nosotros.

Moderadora

Sí, brevemente. Ajá, para beneficio de todos.

P#2LD

[no se entiende el comienzo]… toda la comunidad está envuelta.

Moderadora

Ok.

P#3TI

Este… como dijo el compañero aquí, pues, para beneficio de la comunidad entera. No P#4EGamente, verdad, pues, para los niños, ancianos, para todo el mundo, porque cualquiera podemos ser víctima.

Moderadora

Ok. Ok. ¿Y usted, por qué la apoyaría?

P#4EG

Sí, yo lo apoyo porque es beneficioso para todo el mundo. Lo que pasa es que, probablemente, nosotros desconocemos qué recursos no tenemos… tenemos nosotros a la disposición para nosotros proponer eso. Un ejemplo de esto sería, crear un censo de los residentes que están en nuestra comunidad. Y de esa manera, cuando nosotros creamos un censo, determinamos cuántos son los componentes de la familia. Como hacen aquí. El número tal tiene tantas personas, tantos adultos, tantos jóvenes… la experiencia que yo tengo es que la mayor cooperación nosotros la vamos a tener de los de la tercera edad y de los jóvenes, adolescentes, jóvenes adultos. Porque ya cuando las personas están en sus trabajos, pues están más concentrados en la crianza y en la forma de cómo hacerlo. Y mi… otras cosas que yo he participado, verdad… ese censo ha sido genial. ¿Por qué? Porque eso lo que va a reforzar es, qué sectores van a necesitar más ayuda y más participación de los otros miembros de la comunidad, para que ayuden a esas tareas, para que se puedan conformar o estructurar la manera en que nos vamos a ayudar. Por lo menos la experiencia que yo he tenido cuando hago un censo, que lo nivelo entre las familias… cuántos son niños, jóvenes, jóvenes adultos, tú ves, personas de 20 a 40 años, si están casado, si se han casado, ya los adultos retirados que estén enfermos que no estén enfermos, de esa manera tú vas a establecer cuál va a ser tu objetivo y cómo tú vas a poder ayudar a esa comunidad para que tenga éxito el programa.

Moderadora

Ok. Que usted lo apoyaría siempre y cuando, se pueda hacer ese censo…

P#4EG

Que hagamos algo estructurado. Exacto.

Moderadora

Aján. Y organizado para distribuir las tareas…

P#4EG

Exacto.

Moderadora

Ok. Bien.

Pregunta 5a- ¿Piensa que su comunidad apoyaría esta actividad? Sí, No, ¿Por qué?

Moderadora

Eh… ahora, ¿piensa que su comunidad apoyaría esta actividad? Pensando en sus vecinos, los residentes. ¿La apoyarían…? El número dos (*P#2LD*) me dice que sí. Que lo apoyarían.

P#1LD

Sí, porque eso es para el beneficio de todos.

Moderadora

Ok. Y, ¿la misma contestación? [dirigido al número dos (*P#2LD*)] Que es para beneficio…

P#2LD

Sí.

Moderadora

…Ok. De todos.

P#3TI

De la misma manera, es para beneficio de todos.

Moderadora

Que lo apoyarían por eso. ¿Y allá?

P#3TI

Y en lo que…

P#4EG

Ok, me estás hablando la comunidad, no yo.

Moderadora

Exacto. Ahora su comunidad la que…

P#4EG

Yo entiendo, que, si no se les explica, no la van a apoyar. Yo entiendo. Pero por eso es importante, primero estructurarlo, establecer los censos y cómo lo vamos a hacer para entonces, involucrarlos.

Moderadora

Ok. Chévere, chévere.

Pregunta 6- ¿Qué otra información necesitaría para entender mejor esta actividad?

Moderadora

Ahora, ¿Qué otra información necesitaría para entender mejor esta actividad? O, ¿con esta información es suficiente? O, ¿se necesita algo adicional con lo que le expliqué? Ustedes me dirán. ¿Necesitan algo adicional? Vayan pensando…

P#4EG

Bueno, entrenarnos bien.

Moderadora

Entrenarnos bien.

P#4EG

Nosotros entrenarnos bien de cuál es el uso, poder dominar el tema de forma tal que cuando nosotros lo comuniquemos seamos efectivos.

Moderadora

Ok. Ok. Que quizás, a parte de esta información, tener algo más vasto, más completo.

P#4EG

Exacto. Sí, porque muchas veces traen otras preguntas y no estamos preparados para poderlas contestar.

Moderadora

Ok. ¿Se le ocurre alguna información en específico?

P#4EG

No. No, quizás, esto mismo que estamos haciendo de forma tal de que se entrenen los grupos para que a su vez puedan entrenar a otras personas.

Moderadora

Ok. Chévere. Eso lo hacemos nosotros. Así que, se puede organizar algún adiestramiento para las comunidades.

Pregunta 7- ¿Considerarían realizar ustedes mismos esta actividad?

Moderadora

Ahora, individualmente. ¿Considerarían realizar ustedes mismos esta actividad? ¿Ustedes considerarían realizar la reducción de fuentes de mosquitos en su hogar? Honestamente.

P#1LD

Honestamente, yo, yo… yo puedo.

Moderadora

Lo consideraría…

P#1LD

Siempre y cuando mi salud me lo permita y el tiempo que tenga disponible. Porque yo no dependo de mí P#4EGo. Ahora mismo, este pues… este… mi familia, son personas, ya mayores de 92, 94 años, con Alzheimer y hay que dedicarles mucho tiempo a ellos. O sea, que no puedo estar todo el tiempo…

Moderadora

Ok. Que lo consideraría siempre y cuando su salud y el tiempo que tiene disponible, lo permita. Está muy bien. ¿Y acá?

P#2LD

Lo hago, lo practico.

Moderadora

Lo practica ya. Ok.

P#2LD

Sí, en casa no hay agua estancada. Yo, agua que veo, la elimino.

Moderadora

Ok. Ok. Que bien. ¿Y allá?

P#3TI

Sí, de igual manera.

Moderadora

Ya usted me había dicho que sí.

P#3TI

Sí, sí se está haciendo y yo lo estoy practicando.

Moderadora

¡Qué bueno! ¿Y usted, lo consideraría?

P#4EG

Sí. Yo… no, de hecho, yo lo practico. Al igual que hace el compañero, el número dos (*P#2LD*), pues yo trato de mantenerlo todo el año, de forma tal que una vez yo identifico que puede ser una fuente de crianza de mosquitos, lo elimino. De hecho, hay unas preguntas, que después más adelante, porque hay ciertas matas que puede provocar esto. Y tengo entendido que mientras uno le esté cambiando el agua, echándole agua constantemente evita que se propaguen.

Moderadora

Sí. Y limpiarlas… podemos hablar ahorita, sí de eso.

P#4EG

Ok.

Moderadora

Porque se ha hablado también de las bromelias. Por ejemplo…

P#2LD

Eso, sobre todo las bromelias.

Moderadora

Sí. Pero exacto, limpiarlas con agua, así un poquito de presión que saque lo que hay adentro del huequito… sí, y vamos a hablar sobre otras estrategias que ayuda también a controlar los mosquitos ahí.

P#4EG

Ok. Perfecto.

Pregunta 8- ¿Qué les haría difícil realizar esta actividad?

Moderadora

Ahora. Ya me mencionaron a cuidar a familiares que tienen su salud comprometida también, se les haría difícil para realizar esta actividad. Usted número uno (*P#1LD*). ¿Hay algo más que se les haga difícil a algunos de ustedes, para realizar esta actividad? ¿Algo adicional? Ok. Bueno.

Pregunta 9- ¿Hay algo que podría ayudarles a realizar esta actividad de manera más fácil?

Moderadora

¿Hay algo que podría ayudarles a realizar esta actividad de manera más fácil? Por ejemplo, que usted menciona, quizás, el tiempo. ¿Qué le ayudaría a realizar esta actividad de forma más fácil?

P#1LD

Yo, semanalmente, deshierbo el área de… frente a mi casa y la parte atrás a la avenida y a los vecinos también. O sea, ahora mismo, el vecino mío está afuera de Puerto Rico, y yo me encargo de deshierbarle la parte de atrás y al frente. Y así, pues, este, ayudo.

Moderadora

Ok. Ayuda. Que, aunque sea un tiempito, una vez a la semana, sí trata de sacar…

Pregunta 9a- ¿Necesitarían más información?

Moderadora

Ahora, ya me habían mencionado, que más información y adiestramiento los puede ayudar también para realizar esta actividad.

P#3TI

Aján.

Pregunta 9b- ¿Necesitarían más adiestramiento?

[se contestó en otra pregunta]

Actividad #2 – Aplicar larvicidas al agua acumulada

Moderadora

Pues vamos entonces a la próxima actividad. Se llama, verdad, aplicar larvicidas al agua acumulada. Voy a leer la explicación. Los larvicidas son pesticidas que se usan para matar las larvas antes de que se conviertan en mosquitos adultos. Y los larvicidas se pueden aplicar de diferentes maneras, en gránulos, tabletas o líquido. La aplicación de larvicidas puede reducir la cantidad de mosquitos si se aplica correctamente. Los larvicidas no afectan a las personas, ni a sus mascotas, ni el ambiente, si se siguen las instrucciones en la etiqueta. Los larvicidas no deben usarse en el agua potable para consumo humano o animal. Requiere aplicar la cantidad correcta según las instrucciones en la etiqueta. También, requiere reaplicarse cada cierto tiempo. Eso sí, no alcanza lugares que estén ocultos donde los mosquitos se reproducen.

Pregunta 2- ¿Es esta una actividad nueva para usted o es algo que ya había escuchado antes? Si la escuchó, ¿dónde la escuchó?

Moderadora

Eh… ¿esta una actividad la habían escuchado anteriormente? ¿No? ¿Alguien sí la había escuchado anteriormente? ¿Sí? Número tres (*P#3TI*) …

P#3TI

Sí, la había escuchado. Incluso, alguien me regaló unos… como unas donitas, que eran los larvicidas. Para echarlo para prevención, por si acaso salían. Y pues yo digo, ‘pues, en casa no tengo…’, pues se lo regalé a alguien que sí que tenía más potencial y que podía utilizarlo más.

Moderadora

Ok. Ok. Bueno. Seguimos… eh… los demás no lo habían escuchado.

Pregunta 3- ¿Cree que esta actividad reduciría el número de mosquitos en su comunidad? ¿Por qué?

Moderadora

Y cree que esta… ¿Creen que esta actividad reduciría el número de mosquitos en su comunidad? Ustedes me dicen.

P#4EG

Pues no sé.

Moderadora

No saben. Ok. Ok. ¿Y por acá?

P#2LD

Sí, porque es un veneno para los mosquitos.

Moderadora

¿Es qué?

P#2LD

Es un veneno para los mosquitos. O sea, que los va a eliminar *anyways*. El larvicida.

Moderadora

Ok. Que sí lo reduciría la cantidad.

P#2LD

Sí, seguro.

Moderadora

Y acá, ¿por qué no sabe si reduciría la cantidad?

P#4EG

No porque no… es la primera vez que lo escucho y desconozco cuales son los…

P#1LD

El producto.

P#4EG

El componente. El producto.

Moderadora

Ok. Ok. Y entonces, acá ya me dijo el por qué.

Pregunta 3a- ¿Qué beneficios o ventajas tiene esta actividad para usted?

Moderadora

Ahora, de lo que ustedes ven presentado. ¿Qué beneficios o ventajas tiene esta actividad para usted de lo que les expliqué? ¿Qué beneficio o ventaja? Ya que usted también lo había escuchado.

P#3TI

Pues había… continuar con la prevención, verdad. Y si tenemos herramientas para mantener esa prevención pues, estaríamos victoriosos de no tener mosquitos. En nuestras comunidades y nuestro hogar.

Moderadora

Ok. ¿Usted ve alguna ventaja?

P#2LD

Igual que la número tres (*P#3TI*). Es prevención. Todo es prevención.

Moderadora

Ok, la prevención… y para el control de mosquitos. Ok.

Pregunta 3b- ¿Qué desventajas o dificultades le ve a esta actividad? ¿De qué forma se podrían P#4EGucionar esas dificultades?

Moderadora

¿Ven alguna desventaja o dificultad ahora mismo? Con esta…

P#4EG

No puedo evaluarlo.

Moderadora

Ok. ¿Qué pregunta… por ejemplo, necesita… qué información adicional necesitaría para entenderlo mejor?

P#4EG

Sí, los componentes del larvicida.

Moderadora

Componentes del larvicida. Ok.

P#4EG

Sí.

Moderadora

Brevemente…

P#4EG

Dónde se obtiene.

Moderadora

Ok. Brevemente le voy a explicar rapidito. El larvicida hay uno de… Hay diferentes tipos. Que pueden venir, como explica, en gránulos, tabletas, o líquido, y hay uno que se llama *Bti* que venden, por ejemplo, en *Home Depot*. Y este es orgánico. Es una bacteria, que lo que hace es que, cuando la larva se come esa tableta o gránulo, piensa que está llena y entonces pues, va a morir. Eh… porque cree que está llena y no se alimenta. Así que, muere. Y eso es lo que hace el larvicida como tal, el *Bti* en específico. Y ese es el que se está… por lo menos CDC y también diferentes organizaciones para el control de mosquito, pues, llevando a los residentes…

P#3TI

¿Tiene un químico? ¿No es un químico que va a hacer daño a nada, no?

Moderadora

En este caso, el *Bti*, no.

P#3TI

Ok.

Moderadora

Y ese el que recomienda el CDC. Ahora, eh… no se pone… eh… no se debe usar, verdad, en agua potable para consumo humano o animal, aunque no hace daño, pero ¿por qué? Porque… Se recomienda porque a veces no leemos las instrucciones y si echamos de más pues puede que sí, quizás, ocasionar alguna reacción, no sabemos. Y entonces, y… de hecho, hay que seguir la etiqueta según la cantidad de agua, es la cantidad de larvicida que se echa. Esto lo usan para las bromelias que le estaba diciendo ahorita. Le echan unos pocos de gránulos, y entonces mata las larvas de... Lo utilizan para eso.

P#3TI

Ya que hablas de las bromelias. Es que estaba hablando con mis hermanas el domingo. Y estábamos hablando de las bromelias y entiendo que, verdad, según la orientación que se está dando… personas que bregan con plantas y con lo que es huertos caseros y todo esto. Que la bromelia crea su propia larva que no deja que otra cosa venga a su medio ambiente. Que ella tiene su propia defensa y que eso no pasa. Que eso es como un tabú, que las bromelias aguantan agua y que viene la larva.

Moderadora

Ok. De hecho, los coquíes se comen las larvas que se crían en las bromelias. Sí, que ese ecosistema entonces, pues esa sería la respuesta más indicada, crea esa propia defensa para que no se sigan propagando el mosquito.

P#3TI

Ok. Hay que dejar los coquíes.

Moderadora

Exacto. Necesitamos muchos coquíes, verdad, para que entonces se coman todas las larvas.

P#1LD

Para aclarar a número tres (*P#3TI*), este… aquí en este mismo centro comunal, nos visitó Douglas Candelario y uno de los temas que él tocó precisamente fue ese de las bromelias. Que es un mito de que las bromelias acumulan el agua y de ahí se cría la larva del mosquito. Y el dice que no porque ella produce esa…

Moderadora

Ese medio ambiente.

P#1LD

Sí. Y no deja que el mosquito se reproduzca.

Moderadora

Sí. Sí. Porque son muy pocos los que van a emerger de la bromelia. Pero, para aquellas personas verdad, que aun así…

P#4EG

Pero, yo he escuchado que, si no mantiene el agua fresca de la bromelia, pues, con el tiempo lo que hace es que la larva del mosquito entonces se empieza a criar. No sé qué verdad sea. Lo que sí es que hay que mantenerla con el agua fresca… ayuda al ecosistema. Y estoy hablando una cosa… repitiendo algo que verdaderamente desconozco.

Moderadora

No claro, claro. Es que también hay otros… eh… no P#4EGamente hay *Aedes aegypti* que pone, verdad, sus huevitos en diferentes lugares. Si no, hay otros tipos de mosquitos. Y entonces, en ocasiones hay larvas de diferentes tipos de mosquitos en la bromelia y como que cada uno, pues, va a tratar de sobrevivir, y a lo mejor también, verdad, de los adiestramientos que yo he estado, pues hay poco espacio para mucha larva. Así que no siempre van a emerger muchos mosquitos de las bromelias, por esa competencia entre diferentes mosquitos…

P#4EG

Ok. Ok. Que ellos mismos crean su propia lucha. Y se destruyen a ellos mismos.

Moderadora

Sí. Así que… Pero para aquellas personas que aun así se preocupan porque tienen muchas bromelias, etc., pues eso es una de las alternativas también. El larvicida. Ahora vamos a continuar. Eh… no sé si yo hice ya, las desventajas o dificultades que ustedes le veían a esta actividad. ¿Sí? Ok.

Pregunta 4- ¿Cuán posible es realizar esta actividad en su comunidad para reducir el número de mosquitos? ¿Por qué?

Moderadora

¿Cuán posible es realizar esta actividad en su comunidad? El de aplicar larvicidas al agua acumulada. ¿Cuán posible ustedes lo ven? En sus comunidades…

P#2LD

Se puede hacer.

Moderadora

¿Lo ve posible?

P#2LD

Lo veo posible.

Moderadora

Ok. ¿Y por qué?

P#2LD

Como dije ahorita, es para prevención. Porque ellos no van a estar en la negativa de hacerlo si es para el bien de ellos.

Moderadora

Ok. Ok. Que esa percepción de que es para el beneficio de ellos lo haría posible. ¿Y por acá? ¿Alguna otra opinión?

P#4EG

Sí, yo entiendo que es una herramienta adicional a la fundamental que es mantener el área limpia, verdad, libre de lugares que se pueda… almacenar agua. Con esto pues hay una serie de… es una alternativa o una herramienta adicionales para la prevención.

Moderadora

Sí. El detalle verdad, como pueden ver aquí en la lámina. Alcantarillas, pozos sépticos, verdad, esos lugares donde va a ser difícil cambiar el agua, pueden echarle larvicidas entonces mataría las larvas.

Pregunta 5- ¿Apoya usted esta actividad en su comunidad? ¿Por qué? (*Preguntar a cada participante del grupo de discusión*)

Moderadora

Ok. Pues continuamos. ¿Apoya usted esta actividad en su comunidad?

P#1LD

100% lo apoyo. Porque es para beneficio de uno mismo, y la comunidad.

Moderadora

Ok. ¿Y por acá? ¿Lo apoya?

P#2LD

100%

Moderadora

¿100%? ¿Por qué lo apoya?

P#2LD

Por lo mismo es por el bien de la comunidad. O sea que, uno ve ninguna aversión por no hacerlo. Es para nosotros, es por nosotros.

P#3TI

100% lo apoyo. Y pues para beneficio de toda la comunidad y de mí misma familia.

P#4EG

Sí la apoyo.

Moderadora

La apoya, ¿por qué la apoya?

P#4EG

Sí la apoyo, porque este… es una… como expliqué, es una herramienta adicional que tú le vas a proveer a la comunidad para evitar la propagación del mosquito.

Pregunta 5a- ¿Piensa que su comunidad apoyaría esta actividad? Sí, No, ¿Por qué?

Moderadora

Ahora. ¿Piensan que su comunidad apoyaría esta actividad? ¿La comunidad como tal? ¿Sí? ¿Por qué?

P#3TI

Yo entiendo que sí, porque es para el propio beneficio para continuar la prevención. Que pues… eh… estemos en mejor estado de salud, este, dejándose llevar por la prevención.

Moderadora

Ok. ¿Y ustedes piensan que su comunidad esté en…?

P#1LD

Sí. Yo creo que la comunidad la apoya 100%. Porque todo es para beneficio de nosotros.

Moderadora

Ok. ¿Y acá piensa que su comunidad la apoyaría?

P#4EG

Sí, como había explicado anteriormente, yo entiendo que es cuestión de adiestrarlos, informales, verdad, comunicarles el beneficio que tiene.

Moderadora

Ok.

Pregunta 6- ¿Qué otra información necesitaría para entender mejor esta actividad?

Moderadora

Ahora, me habían hablado de que necesitaban información sobre de qué se componía el larvicida. ¿Qué otra información necesitarían para entender mejor esta actividad? Además de lo que está compuesto el larvicida. ¿Alguna otra información?

P#4EG

Sí, los efectos que pueda tener.

Moderadora

Efectos que pueda tener. Ok.

Pregunta 7- ¿Considerarían realizar ustedes mismos esta actividad?

Moderadora

¿Considerarían realizar ustedes mismos esta actividad? ¿Sí, número dos (*P#2LD*)?

P#2LD

Sí.

Moderadora

Ok. ¿Y alguien más consideraría realizar esta actividad?

P#3TI

Sí.

Moderadora

Ok. Número tres (*P#3TI*) y cuatro (*P#4EG*).

P#1LD

Yo también, sí.

Moderadora

Número uno (*P#1LD*).

Pregunta 8- ¿Qué les haría difícil realizar esta actividad?

Moderadora

Pregunta ocho. ¿Qué les haría difícil realizar de esta actividad? Algo que vean difícil de realizar. Hasta ahora nada.

P#4EG

Este… yo entiendo, como expliqué ahorita, nosotros dominar o estar claro de los efectos secundarios y los efectos que pueda tener tanto positivos como negativos, el larvicida, para nosotros entonces, recomendarlo a la comunidad. Porque a la medida que uno no… desconozca, quizás no tenga éxito.

Moderadora

Ok. Ok. Chévere.

Pregunta 9- ¿Hay algo que podría ayudarles a realizar esta actividad de manera más fácil?

Moderadora

Y, ¿Algo más que le podría ayudar para realizarlo de manera más fácil? Además de información, adiestramiento. ¿No? Ok.

Pregunta 9a- ¿Necesitarían más información?

[se contestó en otro lado]

Pregunta 9b- ¿Necesitarían más adiestramiento?

[se contestó en otro lado]

Actividad #3 – Rociar larvicida desde un camión

Moderadora

Pues vamos a la próxima. Hablando de larvicidas, que les dije que podrían ser en gránulo o líquidos, pues cuando es de forma líquida se puede rociar larvicida desde un camión. Esto no es insecticida, esto sería rociar larvicida. Los larvicidas se pueden aplicar de diferentes maneras. Pero muchos programas de control de mosquitos han encontrado, que aplicar larvicida desde un camión puede ser efectivo para alcanzar otros lugares donde se encuentran las larvas. El larvicida se rocía desde un camión sobre edificios, y en la vegetación, y en propiedades, terrenos, etc. Los larvicidas tienen que reaplicarse regularmente.

Pregunta 2- ¿Es esta una actividad nueva para usted o es algo que ya había escuchado antes? Si la escuchó, ¿dónde la escuchó?

Moderadora

¿Habían escuchado sobre esta actividad?

P#1LD

Sí, anteriormente la había escuchado. Lo que pasa es que tengo una pequeña duda.

Moderadora

Ajá. Sí. Claro.

P#1LD

Los químicos del larvicida, nosotros lo confundimos con los químicos para…

Moderadora

¿De insecticidas? Para fumigar.

P#1LD

…de herbicida a las plantas. O sea que, no sabemos distinguir si este es más perjudicial o el otro es más perjudicial para la salud de la comunidad.

Moderadora

Ok, necesitaría más información sobre si es perjudicial para la vegetación.

P#1LD

Y necesitamos esta información para transmitirla a la comunidad, porque yo puedo ir y decirles a las personas, ‘esta semana voy a fumigar o voy a repartir larvicida’, y hay personas que me van a decir, ‘no a mi casa no entres, no quiero eso en mi casa’, porque desconocen. Este… los…

Moderadora

Los detalles.

P#1LD

Los detalles, sí. Si nos adiestran bien y nos informan bien, pues esa información nosotros la transmitimos a…

Moderadora

La transmiten. Ok. Le pregunto un momentito, porque quería saber dónde había escuchado de esta actividad. De rociar larvicida desde un camión.

P#1LD

¿De rociar… la actividad?

Moderadora

Ajá. Larvicida.

P#1LD

Sí, eso sale mucho en la televisión y en los periódicos y aquí en la comunidad, años atrás, eso cada vez venían a fumigar. De hecho, mucha gente se… no se si fue el año pasado o el anterior, llegó hasta la televisión, de que no se… había mucho mosquito y los alcaldes no habían hecho nada por fumigar y la comunidad pues se [no se entiende la palabra]. Y creo que surgió efecto, porque cuando una comunidad se levanta y hace fuerza, pues se triunfa.

Moderadora

Número dos (*P#2LD*).

P#2LD

Una pregunta, una duda que tengo. Cuando el municipio fumiga. ¿Usa herbicida o insecticida? Digo, ¿larvicida o insecticida?

Moderadora

Hasta ahora es insecticida. Esto es una actividad nueva que estamos preguntando…

P#2LD

Ok. Es la cosa…

Moderadora

…por eso les decía… larvicida.

P#2LD

O sea, que esto es nuevo ahora. Verdad. O sea, lo que el municipio pasaba por ahí era insecticida.

Moderadora

Insecticida.

P#2LD

Entonces, mi pregunta es, ¿esto tiene olor? ¿Algún olor fuerte? O algo o ¿es sin olor?

Moderadora

Bueno en ese caso no le sabría decir porque ni lo he experimentado yo en persona, y esa información tampoco la tengo ahora mismo, del olor que puede causar.

P#2LD

Si no es insecticida, que no causara daño al ambiente, P#4EGamente va a atacar al mosquito…

Moderadora

Ajá, a la larva.

P#2LD

… pues, 100%... ajá, la larva, pues 100% lo apoyaría. Pero hay que ver el olor. El olor que no vaya a afectar a las personas con asma, a las personas que tienen enfermedades respiratorias y a qué horas se iría a fumigar.

Moderadora

Exacto.

P#2LD

El municipio tiene que dar la hora. Y pasar por… ‘mira, a tal hora, tal día, vamos a fumigar, estén preparados’, no hacerlo como hacen ahora que a cualquier hora aparece el *truck* por ahí, fumigan y uno aguantando todo eso que…

Moderadora

Ok. Ok. Que necesitarían saber la hora y efectos del olor.

P#2LD

Y los efectos del olor. Ya sabemos que no causa daño a los demás insectos. O sea, que eso está bien. Lo demás el olor.

Moderadora

¿Y usted…? Ah, por acá no la habían escuchado, me habían dicho.

P#4EG

No.

Moderadora

Ok. Está bien.

Pregunta 3- ¿Cree que esta actividad reduciría el número de mosquitos en su comunidad? ¿Por qué?

Moderadora

Ahora, ¿Creen que esta actividad reduciría el número de mosquitos en sus comunidades?

P#2LD

100%.

P#3TI

100% Sí.

Moderadora

¿Y usted? ¿Sí? Número dos (*P#2LD*) y tres (*P#3TI*). ¿Y usted cree que la reduciría?

P#4EG

Pues realmente, como no sé lo que es un larvicida, no tengo para evaluar. Y déjame hacerte una aclaración, ya que los compañeros lo estaban hablando. Hace un año, año y medio a tras cuando estaba el problema del Zika, entiendo que el gobierno y el CDC informaron bien del producto que se iba a utilizar. Eso creó una serie de crisis y comienzan, supuestamente, personas que son duchos en la materia a traer una información inadecuada. Yo presumo que es inadecuada porque, en mi opinión, si bien es cierto que tú vas a educar, tú debes tener, como estaba explicando anteriormente, todos los elementos y adiestramientos adecuados para tú poder hacer una recomendación. Entiendo que aquello se desvirtuó. Para mi sorpresa, no bien aquí no se aceptó, en Miami tuvieron unos problemas con el mosquito que estaba haciendo el Zika y en Miami lo asperjaron. Verdaderamente, desconozco qué resultados tuvo aquello, a la larga en el medio ambiente y los individuos, porque yo sé que tomar mucha agua, mucha agua no es beneficioso para los riñones y crea una seria de problemas. Por lo tanto, yo entiendo que todo se puede hacer, siempre y cuando esté en las manos y la información adecuadas. Y aquello creó una crisis y que por eso no se pudo traer insecticida, en un momento dado… o larvicida, no sé lo que era…

Moderadora

Aquí en lo próximo insecticida.

P#4EG

Exacto. Poderlo usar, y beneficiarnos nosotros… pues, porque no se informó adecuadamente. Entiendo que se encerró la información y no se… dio males para que pasaran otras cosas.

Moderadora

Claro, tiempo adecuado y educar muy bien. Pero, ok, usted no sabría si reduce el mosquito en su comunidad porque no tiene la información completa. Le faltaría más estudios sobre los efectos si reduce o no. Ahora, ustedes sí creen que reduciría el número de mosquitos en sus comunidades, ¿y por qué creen que reduciría el mosquito? La cantidad de mosquitos.

P#2LD

Sí.

Moderadora

Que lo reduciría. ¿Y por qué creen que lo reduciría? El rociar larvicidas.

P#2LD

Bueno, fue creado para eliminar a las larvas, ¿no?

Moderadora

Las larvas, para eliminar las larvas.

P#2LD

Si se elimina la larva, se elimina el mosquito.

Moderadora

Ok. Elimina el mosquito.

P#4EG

No, y yo entiendo que, con el sistema de rociar, podría ser más efectivo porque va a llegar a lugares que con los sistemas manual, porque cuando hablamos, pues, probablemente hablamos de la casa, pero no hablamos de las otras áreas que nosotros no tenemos acceso.

Moderadora

Claro. Ok.

Pregunta 3a- ¿Qué beneficios o ventajas tiene esta actividad para usted?

Moderadora

Mas o menos me habló también, que eso sería quizás una ventaja, verdad.

P#4EG

Sí.

Moderadora

De que entonces, el rociar llega a otros lugares. ¿Alguna otra ventaja que vean de rociar larvicidas desde un camión?

P#3TI

Hay áreas en las comunidades que nosotros no tenemos accesibilidad. Verdad, entonces, este, pues eso sería una manera de impactarlo.

Moderadora

Ok. Chévere.

Pregunta 3b- ¿Qué desventajas o dificultades le ve a esta actividad? ¿De qué forma se podrían P#4EGucionar esas dificultades?

Moderadora

Bueno, pues, ¿qué desventajas o dificultades ustedes le ven a esta actividad? Si no saben no hay problema.

P#2LD

Bueno, lo único que he querido evitar, el olor.

Moderadora

El olor, ahí le ve…

P#2LD

Enfermedades respiratorias. Esa es la única desventaja que le veo.

P#3TI

Lo mismo. Si tuviera algún olor porque cuando… hace unos añitos atrás se hacia la asperjación, verdad con el insecticida, yo tenía que coger a mí hijo, ’mira, escóndete en el cuarto y no salgas en buen rato’, porque era… es asmático y le servía… ya tú sabes, era darle veneno a él.

P#1LD

La única dificultad que yo puedo ver… la fumigación es si lo hacen como lo están haciendo actualmente. Que ellos pasan por al frente de la casa y piden… pero el foco de los mosquitos, o la larva, no están al frente de la casa, eso mayormente es la parte posterior de la casa, que es dónde nosotros almacenamos todas las cosas. Tiramos las gomas, tiramos las neveras, tiramos todo y ahí es donde está el foco. Yo creo que debe ser una fumigación que esté bien eficiente, o sea que… que no sea por camión… como en los tiempos de antes que venía un señor que se vestía de Kaki, iba casa por casa, se metía a la… por todo el patio de la casa y a vuelta redonda… y fumigaba. Eso es una… bien, bien eficaz.

Moderadora

Ok. Vamos a hablar algo… a lo mejor de lo que usted está hablando ahora, ya mismito. Sí. Eh… P#4EGo quería resaltar que una forma que se podía re… P#4EGucionar una de las dificultades, me dijeron que un horario establecido para entonces, la persona saber si van a rociar con larvicida y pues, hacer los arreglos, no.

P#3TI

La comunicación. Porque cuando iban a fumigar, se comunicaban, ‘tal día van a fumigar estas comunidades’, ‘tal día, estas otras comunidades’, y ya uno, verdad, sin los medios…

Moderadora

Esa comunicación, ¿cómo era?

P#3TI

Este… mira, por lo menos, el municipio de Ponce, el periódico La Perla es muy buena comunicación, lo tiraban. Y hasta cuando empezó Noticias Ponce, y todas estas comunicaciones a través de los celulares, sí… y uno sabía…

Moderadora

Enviaban un texto.

P#3TI

Sí, se enviaban mensajes a la comunidad…

Moderadora

Ok. Eso sería efectivo. Ok.

Pregunta 4- ¿Cuán posible es realizar esta actividad en su comunidad para reducir el número de mosquitos? ¿Por qué?

Moderadora

Bueno, pues entonces… eh… ¿Cuán posible sería realizar esta actividad en sus comunidades? Para reducir el número de mosquitos.

P#2LD

Bueno…

Moderadora

¿Sería posible? ¿Por qué? Si es posible, y si no me dicen por qué no.

P#2LD

Es lo mismo… la misma contestación…

Moderadora

De las anteriores. [risas]

P#2LD

Es la misma. Para cosas de la comunidad ellos no van a decir que no. Ok. Ok. Si tiene algo diferente que decir, me interrumpen. Ok.

Pregunta 5- ¿Apoya usted esta actividad en su comunidad? ¿Por qué? (*Preguntar a cada participante del grupo de discusión*)

Moderadora

¿Apoyarían ustedes esta actividad en su comunidad? Número uno (*P#1LD*). ¿Lo apoyaría? Y, ¿por qué?

P#1LD

Apoyo 100%. Sí y como siempre, es para beneficio de nosotros, para la salud de nosotros.

Moderadora

Ok. ¿Y número dos (*P#2LD*)? ¿Lo apoyaría?

P#2LD

100%.

Moderadora

Ok. ¿Y número tres (*P#3TI*)?

P#3TI

Lo apoyaría, pues porque, vamos a preservar la salud de toda la comunidad.

Moderadora

Ok. ¿Y número cuatro (*P#4EG*)? ¿Lo apoyaría su comunidad?

P#4EG

Sí, sí, siempre y cuando… como…. Regreso al principio. Siempre que orienten bien a uno y uno sepa cuáles son los efectos secundarios.

Moderadora

Ok.

Pregunta 5a- ¿Piensa que su comunidad apoyaría esta actividad? Sí, No, ¿Por qué?

Moderadora

Y, ¿Piensa que sus comunidades lo apoyarían?

P#3TI

Entendemos que….

P#4EG

Igualmente.

Moderadora

Igualmente. Y, la respuesta para el beneficio de todos… Si tienen otra respuesta me dicen, porque si no, voy a brincar, verdad esa parte.

Pregunta 6- ¿Qué otra información necesitaría para entender mejor esta actividad?

Moderadora

Bueno, ¿Qué otra información necesitaría para entender mejor…? Ya me dijeron, el olor. ¿Algo más? Los efectos… ¿Algo más que le venga a la mente, otra información?

Actividad #4 – Fumigación dentro de las casas con insecticida de acción residual

Moderadora

Ok. Bueno. Pues vamos a la próxima. Rapidito, verdad. La fumigación dentro de las casas con insecticida de acción residual. Les leo cómo sirve. La fumigación dentro de las casas con insecticida de acción residual es un método de control de mosquitos donde un profesional adiestrado trata el interior de su hogar con un insecticida. Ahora sí es insecticida. Consiste en rociar las paredes y otras superficies de una casa con un insecticida que continúa funcionando varios meses. Mata los mosquitos que se posen en superficies que han sido rociadas con el insecticida. Y este tipo de fumigación se ha usado en muchos países en el mundo incluyendo Puerto Rico y Estado Unidos. Puede ser efectivo en reducir la cantidad de mosquitos si se aplica a una gran cantidad de casas en un área. Después de aplicarlo, puede que haya olor por unas horas, pero es poco probable que cause daño a las personas cuando se hace correctamente. Requiere del permiso y la disponibilidad del residente para entrar a la casa a fumigar. El uso repetido, a través del tiempo, puede hacer que los mosquitos sean resistentes a los insecticidas. Y esta fumigación debe repetirse para mantener baja las poblaciones de mosquitos.

Pregunta 2- ¿Es esta una actividad nueva para usted o es algo que ya había escuchado antes? Si la escuchó, ¿dónde la escuchó?

Moderadora

Ahora, ¿Han escuchado sobre esta actividad?

P#3TI

Anteriormente…

Moderadora

Anteriormente, número tres (*P#3TI*) la ha escuchado, ¿cuatro?

P#4EG

Estás hablando de insecticida.

Moderadora

Sí de insecticida de acción residual, que es que tiene larga duración. Cuando fumigan con este insecticida.

P#4EG

Pues, eso es… Eso es un producto comercial, ¿verdad?

Moderadora

Sí. Sí.

P#4EG

Los… los exterminadores son los que ha… efectúan eso, ¿no?

Moderadora

Sí. Profesionales adiestrados… eh…

P#4EG

Sin embargo, nosotros nos concentramos más en hormigas, cucarachas, este… ratones…

Moderadora

Sí. Que en mosquitos.

P#4EG

No tanto en mosquitos porque de los que yo he contratado no me hablan de mosquitos.

Moderadora

Claro, claro. Sí. Pues, hay, los hay. [risas] Pero se concentran más, como usted dicen, en otros insectos. Y, ¿dónde lo habían escuchado, esta actividad? ¿Porque lo han hecho en sus hogares? Además…. para otros insectos.

P#1LD

Sí. Por años en casa, cada dos meses, va el fumigador a fumigar dentro de la casa y por fuera de la casa. Y él usa un químico que no tiene olor. Sí, no tiene olor.

Moderadora

Ok. Ese es distinto.

P#1LD

No tiene olor. Y yo se lo recomiendo, no sé si ustedes lo han usado o algo, pero es muy bueno.

Moderadora

Ok. Hay que preguntar el nombre.

P#1LD

Sí, no tiene olor. Y en forma jocosa… de chiste, el suegro mío pues cuando van a fumigar, dice, ‘eso lo que viene es a tirar una agüita por ahí’. [risas] Que van a tirar una agüita por ahí porque eso no mata nada, no huele a nada. Y al otro día cuando va a la marquesina encuentra diez o doce cucarachas muertas.

Moderadora

Sí. Sí. Ok.

P#1LD

Sí, porque ese producto lo que hace es… va al nervio de la cucaracha y la mata.

P#4EG

Lo tumba…

Pregunta 3- ¿Cree que esta actividad reduciría el número de mosquitos en su comunidad? ¿Por qué?

Moderadora

Bueno, y propiamente ahora de mosquitos. ¿Creen que esta actividad reduciría el número de mosquitos en sus comunidades? Si se hiciera en sus comunidades. ¿Usted cree que sí, a lo mejor, el número cuatro (*P#4EG*)?

P#4EG

Sí, porque mientras… si todos lo hacen, pues sí, lo podría reducir. Pero siempre va a haber unas áreas descubiertas.

Moderadora

Ok. Siempre va a haber unas áreas descubiertas que puede que no…

P#4EG

Yo entiendo que la combinación de eventos es lo que sería más efectivo.

Moderadora

Ok. Combinación de diferentes… esta, con otra actividad.

P#4EG

Exacto.

Moderadora

Diferentes maneras. Y por acá, usted dice que…

P#3TI

De igual manera. Pues, porque como te dije ahorita, cuando va el camión asperjando, pues ese impacta áreas que se quedan descubiertas porque pues, no todo el mundo va a ir a fumigar a esa área, que allí no vive nadie, que es un área recreativa. Pues, si hay una asperjación de… bien sea de un… además de la larva, un larvicida o un insecticida, pues impactaría más.

Moderadora

Ok. Bueno, ¿y acá creen que reduciría el número de mosquitos? Esta actividad…

P#2LD

Sí.

P#1LD

Sí.

Moderadora

¿Por qué creen? ¿Por lo mismo que ya dijeron?

P#2LD

Por lo mismo.

Pregunta 3a- ¿Qué beneficios o ventajas tiene esta actividad para usted?

Moderadora

Ok. ¿Ven algún beneficio o ventaja? Ya me explicaron que, entonces, en combinación impactaría los lugares dentro de los hogares.

Pregunta 3b- ¿Qué desventajas o dificultades le ve a esta actividad? ¿De qué forma se podrían P#4EGucionar esas dificultades?

Moderadora

Y, ¿qué desventajas o dificultades le ven a esta actividad?

P#4EG

Es que yo entiendo que hay que usarlo en todo el vecindario. Practicarlo. Porque es…. la única cuestión de fumigar, que tú fumigas en tu casa, pero el vecino no lo hace y vienen los insectos.

Moderadora

La desventaja es que, si no lo hace el vecino, o todos pues no va a funcionar.

P#4EG

Exacto. Bueno, quizás funcione, pero no sea tan efectivo.

Moderadora

Ok, no sea tan efectivo. ¿De qué forma usted cree que se podría P#4EGucionar esa dificultad? Si hay alguna manera.

P#4EG

No sé. Con la ayuda de… a través de…de un municipio o algo que se vaya impactando por área.

Pregunta 4- ¿Cuán posible es realizar esta actividad en su comunidad para reducir el número de mosquitos? ¿Por qué?

Moderadora

Ok. Bueno, pues la próxima es, ¿Cuán posible ven que es realizar esta actividad en sus comunidades para reducir el número de mosquitos? ¿Cuán posible ven esta actividad que se pueda realizar en sus comunidades? Es posible, sí, no, y por qué.

P#3TI

Yo entiendo que a través de… para impactarlo… hojas comunicativas, como un *flyer* donde se le lee como unos puntos clave, de ha… *checkmark*, qué tú haces para prevención…y que va…cada persona vaya marcando y se haga un autoexamen de cómo yo estoy manteniendo mis áreas para la prevención. Y que de esa manera cada persona concientice.

Moderadora

Ok. Ok. Sí. Esa manera ve viable que…

P#4EG

Perfecto.

Moderadora

Ok. Chévere.

P#4EG

Perfecto.

Pregunta 5- ¿Apoya usted esta actividad en su comunidad? ¿Por qué? (*Preguntar a cada participante del grupo de discusión*)

Moderadora

Bueno. ¿Apoyan ustedes esta actividad en su comunidad? Voy a empezar por aquí para que… [risas] ¿Número cuatro (*P#4EG*) la apoyaría?

P#4EG

Sí, sí, la apoyo.

Moderadora

Sí. ¿Por qué?

P#4EG

Bueno, Sí, como dice la número tres (*P#3TI*) pues es cuestión de la manera en que uno lo haga. Que ellos se convenzan de que, sí que es efectivo, no es suficiente que yo crea, es que el lo va a permitir en su casa tiene que estar convencido también.

Moderadora

Sí. Muy importante. ¿Apoya usted esta actividad?

P#3TI

Claro que sí.

Moderadora

Sí. ¿Por qué la apoyaría?

P#3TI

Pues porque…

Moderadora

Porque ahorita me dijo del asma y ahí también habla un poco del olor que…

P#3TI

Este… teniendo en consideración las necesidades de algunas personas que la comunicación efectiva, preparándolo para cuando esto vaya a surgir, la comunicación, que las personas que tengan que tomar sus propias medidas, la tomen.

Moderadora

Ok. Tomando las medidas preventivas necesaria.

P#3TI

Sí, para si se afectara por el…

Moderadora

Ok. ¿Y usted?

P#2LD

Sí.

Moderadora

Sí, número dos (*P#2LD*) lo apoya. ¿Por qué?

P#2LD

Porque sí, porque estamos eliminando el foco de la enfermedad, que es el mosquito.

Moderadora

Sí. Sí.

P#2LD

Y pues tomar las medidas preventivas [no se entiende lo que dice].

Moderadora

Ok. ¿Y número uno (*P#1LD*), lo apoya?

P#1LD

Sí lo apoyo…

Moderadora

¿O no lo apoya?

P#1LD

Con ninguna razón, porque eso es un beneficio para nosotros y para la comunidad, y para todo el mundo. Todo lo que se haga para beneficio de algo, es bienvenido.

Pregunta 5a- ¿Piensa que su comunidad apoyaría esta actividad? Sí, No, ¿Por qué?

Moderadora

Y, ¿Piensan que sus comunidades apoyarían, al igual que ustedes, la actividad? O, ¿piensan que no? ¿Qué sí, que lo apoyarían?

P#4EG

Sí.

P#3TI

Yo entiendo que sí.

Moderadora

Ok. ¿Y por las mismas razones o alguna otra razón?

P#3TI

Misma razón.

P#4EG

…por mismas razones.

Pregunta 6- ¿Qué otra información necesitaría para entender mejor esta actividad?

Moderadora

Ok. ¿Qué otra información necesitan para entender mejor esta actividad? Ya me habían dicho ahorita discutiendo… pero algo adiciona. ¿Alguna duda que tengan? Si les surge una pregunta, me avisan para anotarlo y tenerlo en la grabación.

Actividad #5 – Trampa AGO para mosquitos

Moderadora

Pero vamos a otra actividad que se llama, trampas AGO para mosquitos. ¿La han visto? Voy a preguntarles desde el principio. ¿Han visto esta trampa? ¿Sí? Número dos (*P#2LD*) la ha visto, los otros no la han visto… Número uno (*P#1LD*), ¿la ha visto?

P#1LD

Sí, yo la vi… en un [no se entiende] fue que la vi.

Moderadora

Que sí la ha visto.

P#1LD

Sí, me parece que sí.

Moderadora

Ok, ok. Vamos a ver si es esta entonces me dicen… La trampa para mosquitos AGO, ya ha sido usada en Puerto Rico para reducir el número de mosquitos. La trampa atrae y captura los mosquitos hembra *Aedes aegypti* que buscan envases para poner sus huevos. La trampa consiste en una paila negra de cinco galones, y una cámara de captura… la pueden ver aquí [no se entiende lo que dice] y un escrín o tela metálica. La paila está llena hasta la mitad con agua y heno para atraer a los mosquitos que pueden [no se entiende] en el agua. Y entonces, esa…Dentro de la cámara de captura hay un papel con pega especial que atrapa a los mosquitos… entonces, esa pega especial atrapa los mosquitos cuando entran a poner lo huevos. La trampa contiene material orgánico, que huele. Y no se requiere un entrenamiento especializado para armar la trampa. Sí requiere mantenimiento cada dos meses, para que no se convierta en criadero de mosquitos. Y la trampa reduce la cantidad de mosquitos si se mantiene adecuadamente y se usa en ocho de cada diez hogares de la comunidad. O sea, el 80% de la comunidad, entonces así es efectivo para reducir la cantidad de mosquitos.

Pregunta 2- ¿Es esta una actividad nueva para usted o es algo que ya había escuchado antes? Si la escuchó, ¿dónde la escuchó?

Moderadora

Entonces, sí había escuchado número dos (*P#2LD*). ¿Dónde la había escuchado?

P#2LD

Aquí mismo.

Moderadora

Aquí mismo, ok.

P#2LD

Una conferencia que hubo, dos o tres semanas atrás.

Moderadora

Ah que bien.

P#2LD

Y las trajeron.

Moderadora

Ah sí. Sí. Que bien. ¿Y usted la había escuchado?

P#1LD

Yo la había escuchado anteriormente y por televisión en un reportaje que… no sé si fue… Candelario o uno de ellos, que presentó esa misma trampa, presentó cómo se construía y todo para que la gente viera…. Hacerla.

Moderadora

Esto es producto de Puerto Rico para que sepan [risas]. Y ustedes… tres (*P#3TI*) y cuatro (*P#4EG*) no lo habían escuchado.

P#4EG

No. Yo no lo había escuchado.

Moderadora

Ok.

Pregunta 3- ¿Cree que esta actividad reduciría el número de mosquitos en su comunidad? ¿Por qué?

Moderadora

¿Creen que esta actividad reduciría el número de mosquitos en su comunidad?

P#1LD

Sí.

Moderadora

¿Me dijo que sí?

P#1LD

Sí.

Moderadora

¿Y por qué en específico creen que lo reduciría?

P#2LD

Una trampa para cazarlos.

Moderadora

Una trampa para cazarlos [risas]. Ok. Y acá, ¿alguna otra opinión?

P#4EG

Sí, una herramienta adicional.

P#3TI

Es una herramienta.

Moderadora

Una herramienta adicional…

P#4EG

Una herramienta más para eliminarlos.

Moderadora

Y una herramienta adicional para eliminarlos.

Pregunta 3a- ¿Qué beneficios o ventajas tiene esta actividad para usted?

Moderadora

Ok. ¿Qué beneficios o ventajas tiene esta actividad para ustedes? ¿Qué beneficios le ve? Que sea la trampa… Ajá.

P#3TI

Le veo la efectividad que vas a poder ver el resultado. ‘¿Cuántos he atrapado? Mira, todos estos me estaban rodeando…’ Y te va a concientizar y que no tiene químicos…

P#2LD

Nada.

P#3TI

…no tiene cosas de olor que no te vayan a dañar la salud y el ambiente.

Moderadora

Sí. Usualmente ven esa ventaja otras personas.

Pregunta 3b- ¿Qué desventajas o dificultades le ve a esta actividad? ¿De qué forma se podrían P#4EGucionar esas dificultades?

Moderadora

¿Alguna desventaja o dificultad? O alguna ventaja que vean. De ambas… desventajas o ventajas… Ok

Pregunta 4- ¿Cuán posible es realizar esta actividad en su comunidad para reducir el número de mosquitos? ¿Por qué?

Moderadora

Bueno, pues entonces, ¿cuán posible es realizar esta actividad en su comunidad?

P#2LD

Esa está un poco difícil.

Moderadora

Esa está un poco difícil. ¿Por qué?

P#2LD

Sí porque ellos tienen que entrar a poner la trampa y tienen que ir a buscarla también. Y con los tiempos que estamos viviendo ahora mismo… tú sabes, muchas personas se van a… tú sabes, se van a oponer. Porque tienen miedo a que lo asalten, o tienen miedo… pues, si van identificados, y se comunican anteriormente, puede ser, pero si van así, ‘mira, voy a entrar a tu casa…’. No van a… ni del portón van a pasar.

Moderadora

Ok. Por el miedo, que otra persona o profesional venga a darle mantenimiento y no le den acceso a sus hogares, a los patios… ok.

P#2LD

Pues porque el tiempo que estamos viviendo… tú sabes, hay asaltos, tú sabes, cualquiera se viste como él y… tú sabes, eso es este… el miedo de la comunidad. Esa está un poco difícil.

Moderadora

Ahora, si fuera el mismo residente que aprenda a darle mantenimiento, ¿lo creen viable, creen que es posible que se realice?

P#3TI

Si Es así, sí.

P#1LD

Sí….

P#3TI

Yo entiendo que cada uno que tenga su trampa y tenga el conocimiento y adquiera una destreza, le puede dar el mantenimiento.

Moderadora

Ok. ¿Alguna otra opinión?

P#4EG

Bueno el único inconveniente, partiendo de la premisa de lo que dice el número dos (*P#2LD*), que es que necesitan mucho para que tenga un efecto en la comunidad. Que, al ser tanto, vas a tener que comprometer mucha gente. ¿Por qué? Porque debe ser continuo. En el caso de asperjación pues simplemente, tú en un momento dado, haces y… pero no vas a interrumpir el diario vivir de la persona.

Moderadora

Ok. Sí. Entiendo el punto, sí. Eso sería una dificultad…

P#4EG

Sí, una dificultad.

Moderadora

…de esa actividad. ¿De qué forma usted cree que se podría P#4EGucionar esa dificultad?

P#4EG

Yo creo que ni regalándoselo la gente lo… [risas]. No, no. Es que estoy de acuerdo con lo que dice él. Conociendo la idiosincrasia, verdad, de nuestras comunidades. Mucho compromiso.

P#2LD

Está bien difícil…

Moderadora

Muy importante. Sí, sí… esa…

P#4EG

Hay cosas más sencillas y no…

Moderadora

Número dos (*P#2LD*) y número cuatro (*P#4EG*), pues no la ven viable. Que no sea posible de realizar.

Pregunta 5- ¿Apoya usted esta actividad en su comunidad? ¿Por qué? (*Preguntar a cada participante del grupo de discusión*)

Moderadora

Ahora, ustedes individualmente, ¿Apoyarían esta actividad en su comunidad? Número uno (*P#1LD*).

P#1LD

Sí, la apoyaría.

Moderadora

Sí. La apoyaría. Ok. ¿Por qué? Además de que es el beneficio para todos, ¿alguna otra razón de por qué la apoyaría?

P#1LD

No porque estamos eliminando el foco de…

Moderadora

Eliminando el foco.

P#1LD

Sí y como dice allí, que se usa un término de dos meses, tú sabes, que es un tiempo razonable que uno no tiene que estar constantemente…

Moderadora

Limpiando.

P#1LD

Sí, limpiando y eso. Usted la pone ahí… cuando vengan los dos meses, pues, este… vuelve y…

Moderadora

Pues entonces, lo apoyaría si otras personas le iban a dar el mantenimiento, no usted.

P#1LD

No. Yo mismos la…

Moderadora

Ah, usted también lo podría hacer.

P#1LD

…yo le puedo dar el mantenimiento.

Moderadora

Está bien.

P#1LD

Ahora, lo que estoy notando es que todo lo que se está hablando somos nosotros los que estamos haciendo…

Moderadora

Bueno, hay tres actividades que son los residentes. Ya la de fumigar… perdón, rociar larvicida, es el gobierno, verdad, porque ustedes no podrían ir con el camión… Y vamos a hablar de otras que no son los residentes también. Ya mismito…

P#1LD

Porque es que hay mucho envejeciente. Eh… están encamados, mucho envejeciente en sillón de ruedas. Y usted le va con una trampa de estas, ellos no van a ir al patio a ponerla. Imposible. O sea, que hay que tener un personal que se dedique a esa [no se entiende la palabra final].

Moderadora

Ok. Que entonces, si fuera el residente, tiene que haber un personal para P#4EGucionar esa dificultad.

P#1LD

Correcto.

Moderadora

Ok. Ahora, ¿usted apoyaría esa actividad? ¿Sí?

P#2LD

Yo sí lo apoyaría. Sí porque…

Moderadora

¿Usted la puede cambiar?

P#2LD

Sí, sí. Pues, este… estamos eliminando un foco de la enfermedad. Pero… yo la apoyo, ahora, que la comunidad lo apoye, son otros veinte pesos. O sea, porque yo sé, porque estoy aquí, yo sé de lo que están hablando, pero la comunidad… tú vas a una casa, ellos no estuvieron aquí y va a tener que explicarles a ellos lo que tú me explicaste a mí.

Moderadora

Ok. Que hasta que ellos no sepan, no sabrían…

P#2LD

No te van a dejar entrar. Y como te digo por el tiempo que estamos viviendo, yo creo que tú vas a tocar las puertas y no te van a abrir.

Moderadora

Ok. Entonces, si fuera individual que se cambia y se mantiene la trampa, ¿lo ve que ellos lo apoyarían? O como número cuatro (*P#4EG*) … dice que no… no lo apoyarían. Ok, si fueran ellos mismos, ok. Acá número tres (*P#3TI*), ¿lo apoyaría usted?

P#3TI

Yo lo apoyaría, claro que sí y… pues, teniendo en consideración los atenuantes que afectan ahora mismo en la comunidad, porque la gente ya no se siente segura, ya no se siente segura, porque dice, este… ‘espérate, en esta comunidad hay este proyecto les están trabajando déjame aprovecharme…’ porque pues… este… mira, por ejemplo, cortando los árboles que cayeron del huracán, que todavía están estorbando en los patios. Este… recientemente un familiar de una compañera mía de trabajo, entré este señor con una señora, ‘te vamos a cortar el árbol, nos vas a dar tanto, dame los chavos…’, se fueron, se llevaron los chavos….

Moderadora

Que era para aprovecharse…

P#3TI

O sea que, la desconfianza puede hacer que parte de las comunidades sean una… característica que impida…

P#4EG

Un inconveniente.

P#3TI

Un inconveniente, para que… que impidan que la labor se haga. La desconfianza por lo que estamos viviendo.

Moderadora

Ok. Y, ¿ve alguna forma de solucionar ese problema o…? ¿Ni aún con identificación?

P#3TI

Es que ahora, hoy en día la gente hace la trampa. [risas]

P#1LD

La gente copia los uniformes.

P#2LD

Sí. Tú sabes, que… esa yo la veo…

P#3TI

Copian uniformes… difícil, difícil.

P#2LD

Es por la desconfianza.

P#3TI

Crean los *badge* de identificación falsa. O sea, ya estamos…

P#2LD

Se visten del FBI, imagínate.

Moderadora

Ok.

P#3TI

Estamos viviendo en unos momentos…

Moderadora

O ir acompañados de líderes comunitarios.

P#3TI

Ahí, sí.

Moderadora

Ahí sí. Ir acompañados…

P#3TI

Gente conocida en la comunidad.

Moderadora

Ok, eso, verdad, sería una P#4EGución…

P#4EG

Si se hace un esfuerzo mayor en términos de tener más personal adiestrado.

P#3TI

Empoderar a más gente.

Moderadora

Ok. Empoderar a más gente. ¿Y usted apoyaría esta actividad? Usted me dijo que la comunidad, cree no, pero ¿usted, lo apoyaría?

P#4EG

Sí. Sí. Yo no tengo problema de hacerlo en mi casa. Tremendo, ¿dónde está? [risas]

Moderadora

Las tenemos, las tenemos.

P#3TI

¿Ajá? [en voz de emoción]

Moderadora

Sí, sí. Es parte de por qué también estamos haciendo estos grupos para ver cuáles entienden, verdad, estas actividades, incluyendo la de trampas.

P#3TI

Pues tenemos que hacer ese proyecto y nosotros ponerlo en órbita para que puedan funcionar. [risas]

P#4EG

…salió bueno.

Moderadora

Pero esta semana que usted va a ir a la otra reunión…

P#3TI

Mañana.

Moderadora

…van a estar hablando sobre eso.

P#3TI

Mañana.

Moderadora

Sí. Sí.

[el hijo del número tres (*P#3TI*) dice que no es mañana, sino el jueves]

P#3TI

No perdón, el jueves.

Pregunta 6- ¿Qué otra información necesitaría para entender mejor esta actividad?

Moderadora

Bueno, entonces, ya me hablaron de sus comunidades. ¿Qué otra información necesitaría para entender mejor esta actividad? ¿Qué otra información? O, ¿se entiende bien?

[Nadie dice nada]

Pregunta 7- ¿Considerarían realizar ustedes mismos esta actividad?

Moderadora

Ya me hablaron de que ustedes sí considerarían realizar ustedes mismos esta actividad. Número tres (*P#3TI*) también me había dicho que usted también podría realizar esta actividad y número cuatro (*P#4EG*), usted también. Ok.

Pregunta 8- ¿Qué les haría difícil realizar esta actividad?

Moderadora

¿Hay algo que se les haría difícil realizar de esto? De darle mantenimiento a la trampa…. ¿Que conseguir el heno no se les haría fácil, que es una paquita? Ok.

P#3TI

De lo mismo que se deshierba en la comunidad. [risas]

Moderadora

Pero ese es un heno en específico, o sea, el que se compra, no.

P#3TI

El que se compra, ok.

Moderadora

Sí, sí, porque ese es el olor que va a atraer a la hembra. Para poner el huevo.

P#3TI

Por lo menos en casa de mi papá hay caballos y compran heno. [risas]

Moderadora

Ok. Que bien. Que bien.

Pregunta 9- ¿Hay algo que podría ayudarles a realizar esta actividad de manera más fácil?

Moderadora

Bueno, pues… no hay… algo adicional que necesiten, verdad. Más información… Adiestramiento sí necesitarían. Entiendo yo.

P#1LD

Sí…

P#3TI

Sí una demostración, claro.

Moderadora

O sea, cómo monta, cómo se desmonta.

Pregunta 9a- ¿Necesitarían más información?

[Se contestó en otro lado]

Pregunta 9b- ¿Necesitarían más adiestramiento?

[Se contestó en otro lado]

Actividad #6 – Mosquitos macho y hembra con Wolbachia

Moderadora

Bueno, pues vamos rapidito a las últimas que nos quedan, voy a leerles. Mosquitos con Wolbachia. Wolbachia es una bacteria que vive en muchos insectos incluyendo algunas especies de mosquitos que pican a las personas. Pero la Wolbachia no se encuentra en los mosquitos *Aedes aegypti* que transmiten el dengue, Zika y Chikunguña en Puerto Rico. Wolbachia es una bacteria que vive en muchos insectos, no se encuentra en mosquitos *Aedes aegypti.* La Wolbachia se introduce a los mosquitos *Aedes aegypti* en el laboratorio. Al presente, los estudios muestran que el uso de mosquitos infectados con Wolbachia es seguro para las personas, los animales y el ambiente. Los científicos creen que cuando nacen mosquitos con Wolbachia pueden ser menos capaces de transmitir enfermedades a las personas. ¿Preguntas hasta aquí? Vamos a continuar leyendo la información. Una bacteria natural que vive en muchos insectos, no se encuentra en los mosquitos *Aedes aegypti*. Se les introduce a los mosquitos *Aedes aegypti* en un laboratorio. Y cuando nacen mosquitos con Wolbachia pueden ser menos capaces de transmitir enfermedades a las personas. Hay dos maneras, en la que funcionan los mosquitos con Wolbachia. En la primera se liberan mosquitos *Aedes aegypti* machos y hembras con Wolbachia. Cuando el mosquito hembra con Wolbachia se reproduce con un mosquito macho con o sin Wolbachia la bacteria se pasa a través de la hembra a sus crías, de generación en generación. El mosquito hembra se reproduce con un mosquito macho con o sin Wolbachia y como la hembra tiene la Wolbachia va a pasar a sus crías… Con el tiempo la cantidad de mosquitos con Wolbachia aumenta y reemplaza, reemplaza, a los mosquitos del ambiente sin la bacteria. Después de liberarlos varias veces, porque estos mosquitos se liberan, la población de mosquitos con Wolbachia se mantendrá sin tener que liberar más de estos mosquitos. Los mosquitos con Wolbachia son menos capaces de transmitir enfermedades. Aun habrá mosquitos en la comunidad ya que la intención de este método no es reducir el número de mosquitos sino reducir el riesgo de epidemias. Sin embargo, no se reducirá las picadas por mosquitos. Esta actividad se ha usado en otros países como Colombia y Brasil. Actualmente no hay reglas definidas para el uso de mosquitos macho y hembras con Wolbachia en los Estados Unidos.

Pregunta 2- ¿Es esta una actividad nueva para usted o es algo que ya había escuchado antes? Si la escuchó, ¿dónde la escuchó?

Moderadora

¿Habían escuchado sobre los mosquitos con Wolbachia?

P#1LD

Yo oí un reportaje por televisión en un canal extranjero. Sí.

Moderadora

Televisión. Ok. Ok. Que bien. ¿No se acuerda de qué lugar era? El lugar extranjero…

P#1LD

Este… no, el lugar no recuerdo bien. Pero es un programa que dan por televisión, que no P#4EGamente habla… habla de todas las enfermedades que… o sea, que existen.

Moderadora

Ok. Ok. ¿Y por acá, no lo habían escuchado? Ok. Tampoco.

Pregunta 3- ¿Cree que esta actividad reduciría el número de mosquitos en su comunidad? ¿Por qué?

Moderadora

¿Creen que esta actividad reduciría el número de mosquitos en su comunidad? Dice que no, exacto, están atendiendo. Muy bien.

P#2LD

Ahí dice que no, sí.

Moderadora

¿Qué reduciría entonces?

P#2LD

Las enfermedades. Supuestamente las enfermedades entre comilla. Porque eso hay que cogerlo con pinzas.

P#3TI

Eso es como un proyecto piloto o un proyecto investigativo, a ver cuál es el resultado. ¿Cuántos países además de los que mencionas ahí…?

P#2LD

Eso hay que cogerlo con pinzas. Porque eso trae efectos secundarios.

P#3TI

Exacto…

Moderadora

Sí. Realmente esto está en Australia… Están haciendo este proyecto ahora en diferentes países.

Pregunta 3a- ¿Qué beneficios o ventajas tiene esta actividad para usted?

Moderadora

Bueno, entonces. ¿Qué beneficios o ventajas tiene esta actividad para usted? Para ustedes.

P#2LD

Yo el único beneficio… no hay.

Moderadora

No le ven ventaja o beneficio. Número dos (*P#2LD*), número tres (*P#3TI*).

P#2LD

No veo nada.

P#3TI

Pues mira, ahí dice, este… pues que van a haber mosquitos, te van a picar, o sea pues, no hay balance.

P#4EG

Sí hay balance. Hay balance porque evita la transmisión. Lo que yo entiendo es que está muy reciente porque no hay nada efectivo…

Moderadora

Ok. Está muy reciente.

P#2LD

O sea, no sabemos si va… si transmite o no transmite todavía porque eso es un proyecto piloto. Hay que esperar a los resultados, o sea, que yo no confío en eso. Y menos si es de laboratorio.

Moderadora

Ok. Pero sí han visto que no puede transmitir el virus ni del dengue, Zika y ni Chikunguña.

P#2LD

Eso es lo que dicen ellos.

Moderadora

Al… cuando pican… ok. Que usted está desconfiando de… no cree en eso.

P#2LD

No creo.

Moderadora

Ok.

P#1LD

Yo como lo vi por televisión, pues creo en el proyecto. Como todo, cuando se empieza se va mejorando. Yo creo que, con los años, ese proyecto…

P#3TI

Puede ser más efectivo los resultados.

P#1LD

…va… sí, va a ser más efectivo porque va a ir progresando nuevos estudios, nuevas cosas.

P#4EG

Nos van a cobrar por los mosquitos que pongan en el ambiente. [risas]

P#2LD

Pero mira dónde lo prueban ellos, en los países pobres. O sea, no lo prueban en Estados Unidos. Dónde prueban ellos todos estos virus, en los países más pobres del mundo, Colombia, Brasil, África. O sea, si pasa algo, son países pobres, allá ellos. Ahora, ¿por qué no lo prueban en Estados Unidos acá? O lo prueban en Canadá, donde hay chavos.

P#1LD

En Puerto Rico lo prueban…

Moderadora

Ok. Bueno. Ok. Está bien, está bien. Pues entonces, una forma de P#4EGucionar esto es que tenga información de que en Estados Unidos lo están haciendo.

P#2LD

O sea, es que siempre los virus, siempre los virus los han probado en países pobres, nunca he visto un virus… ¿dónde empezó el SIDA? En África. ¿Dónde empezó el Zika? En Brasil, supuestamente, o sea, países pobres. ¿Por qué nunca se trata de Estados Unidos, donde empieza una enfermedad? Canadá. Siempre empieza en países pobres.

Moderadora

Podemos hablar de eso ahorita, cuando se termine… de hablar un poquito de esa historia, de esa historia.

P#1LD

El agente Naranja fue aquí probado en Puerto Rico.

P#2LD

Sí.

Moderadora

Bueno, ¿alguna forma que ven de P#4EGucionar esa dificultad, esa credibilidad? Verdad… o esperar. Ustedes dirían esperar el tiempo.

P#4EG

Dar tiempo y ver resultados.

Moderadora

Esperar el tiempo…ver el resultado. Ok.

Pregunta 3b- ¿Qué desventajas o dificultades le ve a esta actividad? ¿De qué forma se podrían P#4EGucionar esas dificultades?

[Se contestó en otra sección]

Pregunta 4- ¿Cuán posible es realizar esta actividad en su comunidad para reducir el número de mosquitos? ¿Por qué?

Moderadora

¿Cuán posible es realizar esta actividad en sus comunidades?

P#2LD

No creo.

P#4EG

Como yo, no la van a querer.

Moderadora

No la van a querer.

P#4EG

No.

Moderadora

¿Y usted número uno (*P#1LD*)? [risas]

P#1LD

Tengo duda…

Moderadora

Tiene duda.

P#1LD

Porque es que no sabemos…

P#2LD

Es que eso es algo desconocido, no lo van a querer. Eso es desconocido, tú sabes, que no.

Moderadora

Ok. Pero número uno (*P#1LD*) aunque lo vio por televisión, no ve que a su comunidad es factible realizarlo.

P#1LD

Sí.

Pregunta 5- ¿Apoya usted esta actividad en su comunidad? ¿Por qué? (*Preguntar a cada participante del grupo de discusión*)

Moderadora

Ok, individualmente, ¿apoya esta actividad en su comunidad, número uno (*P#1LD*)? Individualmente.

P#1LD

Individual… para mí sí, porque es algo bueno.

Moderadora

Porque es algo bueno. Ok.

P#1LD

Es algo bueno. O sea, este… los resultados que… los experimentos que han dado han sido positivo, pues entonces, este… las enfermedades van a bajar.

Moderadora

Ok. ¿Y acá?

P#2LD

No.

Moderadora

No. Individualmente, no. ¿Y por qué? Porque ya lo mencionó.

P#2LD

Sí, porque eso es traer un plan piloto y nosotros no somos un mono de laboratorio para que nos cojan de… como especies para probar eso, de conejillos de India.

Moderadora

Ok. Sí. ¿Y el número tres (*P#3TI*), cómo usted lo ve? ¿Lo apoyaría o no lo apoyaría?

P#3TI

No lo apoyaría porque, hay que ver cuáles son los alcances de ese piloto.

Moderadora

Ok. Ver los alcances. ¿Y número cuatro (*P#4EG*)?

P#4EG

Sí, sí. No lo apoyaría por falta de conocimiento.

Moderadora

Ok. No lo apoyaría por falta de conocimiento.

P#4EG

Y efectos.

Pregunta 5a- ¿Piensa que su comunidad apoyaría esta actividad? Sí, No, ¿Por qué?

Moderadora

Ya sí dijeron que en sus comunidades piensan que no lo apoyarían.

Pregunta 6- ¿Qué otra información necesitaría para entender mejor esta actividad?

Moderadora

¿Y, qué otra información necesitaría para entender mejor esta actividad entonces? Además de…

P#2LD

Toda.

Moderadora

¿Cuánto?

P#2LD

Toda, el resultado, todos.

P#4EG

Resultados.

Moderadora

Todos los resultados de todos esos otros proyectos que tienen en diferentes países…

P#2LD

Pruebas.

Moderadora

Pruebas.

P#2LD

Todo.

Actividad #7 – Mosquito macho con Wolbachia

Moderadora

Ok. Chévere. Pues miren, vamos a la próxima que también es la segunda manera de mosquitos con Wolbachia. En la segunda forma P#4EGo se liberan mosquitos macho con Wolbachia. Se liberan mosquitos macho al ambiente, que no pican ni transmiten enfermedades. Miren la Wolbachia, y se unen con hembras sin Wolbachia que hay en el ambiente. Y las hembras sin Wolbachia ponen sus huevos, se fecundan, pero sus huevos no se van a reproducir. Y entonces, los mosquitos con Wolbachia deben liberarse continuamente, en grandes cantidades para mantener baja las poblaciones de mosquitos. Una vez que los mosquitos con Wolbachia dejan de ser liberados la población de mosquitos aumentará de nuevo. Los mosquitos macho con Wolbachia se han utilizado en estudios en estudios en California, eso sí, los machos, en California, Estados Unidos y en los Cayos de la Florida. Y han sido aprobados para evaluación en Miami, Florida.

Pregunta 2- ¿Es esta una actividad nueva para usted o es algo que ya había escuchado antes? Si la escuchó, ¿dónde la escuchó?

Moderadora

¿Han escuchado esta actividad de mosquitos macho? El número uno (), no sé si se refería a la otra o a esta también.

P#1LD

No sé, porque fue que estaba mirando ese programa y me interesó y lo vi pero no sabía si era macho o hembra…

Moderadora

O hembra… ajá, sí, sí.

P#1LD

…el programa no definía. Estaban haciendo ese reportaje.

Moderadora

Ok. Chévere. Y los demás no lo habían escuchado.

P#2LD

No.

Pregunta 3- ¿Cree que esta actividad reduciría el número de mosquitos en su comunidad? ¿Por qué?

Moderadora

Bueno, ¿creen que esta actividad reduciría el número de mosquitos en su comunidad?

P#2LD

No.

Moderadora

Número dos (*P#2LD*) dice que no, y número cuatro (*P#4EG*) …

P#4EG

No. No sé.

Moderadora

…no sabría. ¿Número tres (*P#3TI*)?

P#3TI

No. No sabría.

Moderadora

No sabría. Y número uno (*P#1LD*), ¿cree que lo reduciría?

P#1LD

Bueno si…

P#2LD

Si ahí dice que … [no se entiende lo que dice]

Moderadora

Ay, es que no le escuché, perdón.

P#2LD

Ahí dice que la población de mosquitos aumentará de nuevo.

Moderadora

Aumentaría si se dejan de ser librados.

P#3TI

Si se dejan de…

Moderadora

Ajá, porque mientras se liberan y se juntan con la hembra, pues cuando ponen huevos, la hembra, no van a nacer. Entonces, sí se van a mantener bajas las poblaciones, porque los huevitos no van a nacer.

P#1LD

No se desarrollan. Claro, entonces bajan.

Moderadora

Si se dejan de liberar, sí aumentarían.

Pregunta 3a- ¿Qué beneficios o ventajas tiene esta actividad para usted?

Moderadora

¿Y qué ventaja le ven a esta actividad?

P#2LD

Eso no… esos estudios, y con bacterias, menos.

P#3TI

Porque ahí dice que los huevillos no se van a fecundar, no van a nacer. Ahí bajaría la cantidad de mosquitos. Pero si se deja de hacer el proyecto…

Moderadora

Claro.

P#3TI

…este… continuaría la procreación de mosquitos, que sí serían dañinos y perjudiciales a la salud. Habría que ser consistente. Y esa es la atenuante que encontramos en muchos de estos proyectos, que no hay la consistencia, para una total prevención.

Moderadora

Aján. Sí. Si ven alguna otra ventaja o dificultad.

P#1LD

Este… yo creo que es ventajoso. Porque según yo estaba viendo en ese programa, recuerdo, que actualmente están haciendo pruebas o hicieron pruebas con las abejas. Las abejas ellos le dicen, robóticas, algo así, tiene un nombre… y esa abeja, ellos la sueltan… con… ¿cómo se llama?

Moderadora

¿Control?

P#1LD

Con control remoto, y van a las flores y la van polinizando las flores. Sí. Y yo decía, ‘¿cómo va a ser?’. Y efectivamente, y en ese programa lo presentaron. Y yo digo, ‘hasta dónde ha llegado la ciencia’, que ya… la escasez de abejas que hay, ellos, ya están trabajando en eso, para que no suceda que nos quedemos sin alimento en la tierra. Porque el alimento es la abeja quien lo… y ya están trabajando en la abeja esa que son como robots que van a la flor y…

Moderadora

Qué bueno. Entonces, usted ve que este adelanto científico lo ve ventajoso…

P#1LD

Sí.

Moderadora

…para entonces, reducir la cantidad de mosquitos.

P#1LD

Sí. Yo creo que sí, porque si lo están haciendo con abejas, que lo hagan con mosquitos no quiere decir nada.

Moderadora

Ok. De esta manera la ve beneficiosa.

Pregunta 3b- ¿Qué desventajas o dificultades le ve a esta actividad? ¿De qué forma se podrían P#4EGucionar esas dificultades?

[se contestó en otra sección]

Pregunta 4- ¿Cuán posible es realizar esta actividad en su comunidad para reducir el número de mosquitos? ¿Por qué?

Moderadora

Individualmente… o, ¿cuán posible ven realizar esta actividad… machos, aquello era hembras y machos con Wolbachia, esta de machos P#4EGamente, la ven viable, es posible realizarla?

P#4EG

No creo.

Moderadora

Cuatro (*P#4EG*).

P#4EG

Primera vez que lo escucho y me falta información.

P#3TI

Más información.

Moderadora

Más información. Ok.

P#2LD

No.

Moderadora

No. Usted, no la ve posible…

P#2LD

No la veo… todo lo que venga de laboratorio, trae consecuencias.

Moderadora

Todo lo que sea de laboratorio, trae consecuencias.

P#2LD

Eso es… eso se ha probado. Todo virus que salga de un laboratorio siempre… a la larga o a la… siempre va a traer consecuencias, entonces, como hubo el fallo, entonces es que vienen a la… ‘a Dios, espérate, si tratamos con esto y esto no era así’.

Moderadora

Sí. Sí.

P#2LD

O sea, que yo no confío en los laboratorios. Todo lo de laboratorio siempre ha traído sus consecuencias, eso no falla.

Moderadora

Ok. Ok. Está bien. Y número…

P#1LD

Yo confío en la ciencia y cuando hacen experimentos unos fracasan, otros no fracasan, pero los que no fracasan [los que sí fracasan], pues ellos no lo van a tirar al mercado. Al igual que la medicina. Las medicinas… algunas funcionan, otras no funcionan y esas que no funcionan se eliminan, seguimos con las que son efectivas.

Pregunta 5- ¿Apoya usted esta actividad en su comunidad? ¿Por qué? (*Preguntar a cada participante del grupo de discusión*)

Moderadora

Ok. Yo entiendo que ya número dos (*P#2LD*) me dijo su contestación de que, si apoya esta actividad, y sé que la respuesta es no, con todo lo que me ha explicado. Ahora, número uno (*P#1LD*), ¿usted la apoyaría? Porque ahí… ahí, no sé cuál es su respuesta. ¿La apoyaría esta actividad de machos… de mosquitos macho con Wolbachia?

P#1LD

Sí porque elimina la procreación de… la larva… de la hembra…

Moderadora

Ah bueno, ahí el huevito. El huevito se eliminaría.

P#1LD

El huevito, sí. Se eliminaría. Si se elimina pues ya…

Moderadora

Ok. Número tres (*P#3TI*), ¿usted apoyaría esta actividad? En su comunidad. Mosquito macho nada más con Wolbachia.

P#3TI

Tengo mis dudas.

Moderadora

Sus dudas. Muy bien.

P#3TI

Sí, tengo mis dudas en ambas.

Moderadora

Ok. Y, entonces, ¿por qué…?

P#3TI

Pues porque necesitaría como que más información…

Moderadora

Más información que ya lo había mencionado.

P#3TI

Sí, porque si son unas investigaciones, pues hay que ver los pros y los contras de esa investigación, y cuál va a ser de verdad, la efectividad, y como te dije ahorita, si no va a haber una consistencia después de eso, vamos a estar trabajando para la prevención, pero si no se es consistente, vamos a volver a lo mismo. A tener muchos mosquitos.

Moderadora

Ok. Quizás algo más simple, de una tablita con los pros y los contra de esta estrategia y la otra. Ok.

P#3TI

Exactamente.

Moderadora

¿Y usted apoyaría esta actividad en su comunidad? No. Porque falta información.

P#4EG

Desconocimiento.

Moderadora

Por el desconocimiento. Ok.

Pregunta 5a- ¿Piensa que su comunidad apoyaría esta actividad? Sí, No, ¿Por qué?

Moderadora

Eh… ahora, ¿sus comunidades apoyarían esta actividad? Faltaría información, es lo que veo en general.

P#3TI

Sí.

Pregunta 6- ¿Qué otra información necesitaría para entender mejor esta actividad?

Moderadora

Ok. Información sobre los efectos, los pros y los contras, ¿algo adicional? ¿Qué otra información? ¿En qué laboratorio se hizo? [risas]

P#3TI

Sí, los resultados de esos… de esos *research*. Sí es bien importante… y cuán efectivo y si vamos a gráficas, la duración de la efectividad.

Moderadora

La duración de la efectividad, chévere.

Actividad #8 – Mosquitos modificados genéticamente

Moderadora

Bueno pues vamos a la última actividad. Mosquitos modificados genéticamente. Se liberan mosquitos macho, otra vez machos únicamente, para que se unan con mosquitos hembras del ambiente. Los mosquitos macho modificados genéticamente se reproducen con las hembras del ambiente y pasan un gen a su cría, en este caso un gen a las crías, y ese gen impide que las larvas y las pupas se desarrollen normalmente. Así, estas mueren antes de convertirse en mosquitos adultos. Los mosquitos deben liberarse varias veces a la semana a lo largo del tiempo para mantener bajas las poblaciones de mosquitos. Los mosquitos modificados genéticamente no pican ni transmiten enfermedades. Estos mosquitos se deben liberar varias veces a la semana y en grandes cantidades para mantener bajas las poblaciones de mosquitos *Aedes aegypti* P#4EGamente. Una vez los mosquitos modificados genéticamente, dejan de ser liberados en un área, la población de mosquitos aumentará de nuevo. Los mosquitos modificados genéticamente han sido evaluados en diferentes países incluyendo las Islas Caimán, Brasil y Panamá. Al presente no se han hecho estudios de los mosquitos modificados genéticamente en los Estados Unidos.

Pregunta 2- ¿Es esta una actividad nueva para usted o es algo que ya había escuchado antes? Si la escuchó, ¿dónde la escuchó?

Moderadora

¿Habían escuchado de los mosquitos modificados genéticamente?

P#1LD

Ahí no, no.

Moderadora

Ahí no.

P#3TI

No. Ahí sí que no.

P#1LD

Ahí me cogiste.

Pregunta 3- ¿Cree que esta actividad reduciría el número de mosquitos en su comunidad? ¿Por qué?

Moderadora

Bueno, ¿creen que esta actividad reduciría el número de mosquitos en su comunidad?

P#2LD

No.

Moderadora

Número dos (*P#2LD*) dice que no, número tres (*P#3TI*) …

P#3TI

No porque mira volvemos a lo mismo, dice que, si dejas de llevarlo, dejas de asperjarlo para que se procreen… volvemos a lo mismo, o sea, volvemos a que tiene que haber consistencia en estos proyectos.

Moderadora

Ok. ¿Alguna otra opinión?

P#4EG

Yo no estoy de acuerdo.

Moderadora

No está de acuerdo.

P#4EG

No estoy de acuerdo con nada modificado genéticamente.

Moderadora

Ok. Ok. Está muy bien.

Pregunta 3a- ¿Qué beneficios o ventajas tiene esta actividad para usted?

Moderadora

Bueno, ¿Ven algún beneficio de esta actividad? ¿Alguna ventaja? ¿No? Ok. ¿Iba a decir algo?

P#1LD

No, no.

Moderadora

Ok. Que no lo vi.

P#1LD

Porque desconocemos…

Pregunta 3b- ¿Qué desventajas o dificultades le ve a esta actividad? ¿De qué forma se podrían P#4EGucionar esas dificultades?

Moderadora

Ok. ¿Qué desventajas, o dificultades…? Ya usted mencionó que se volvería a lo mismo si se dejan de liberar. Y, ¿se podría P#4EGucionar eso de alguna manera?

P#3TI

Vuelvo y te digo, si no hay consistencia que se haga… seguimos en lo mismo. Hay que ver.

Moderadora

Consistencia, ok. Y ¿qué otras desventajas le ven? Que usted dice que no… algo modificado…

P#4EG

El principio no… es cuestión de principio.

Moderadora

El principio. Sí. Sí. Que chévere. ¿Otra desventaja?

Pregunta 4- ¿Cuán posible es realizar esta actividad en su comunidad para reducir el número de mosquitos? ¿Por qué?

Moderadora

Ok. Bueno, ¿es posible realizar esta actividad en su comunidad? Número dos (*P#2LD*) dice que no, número tres (*P#3TI*), no. Y número cuatro (*P#4EG*) … No. Y número uno (*P#1LD*), tampoco.

P#3TI

No.

P#1LD

Desconocemos…

Moderadora

Individual… y en sus comunidades piensan que lo... ¿no?

P#2LD

Tampoco.

P#3TI

Tampoco.

P#4EG

Ahí desconozco.

Moderadora

Ok. Cuatro (*P#4EG*) desconoce.

P#1LD

Hay que ver para creer.

Moderadora

Ok. Hay que verlo para creer, usted diría. Bueno, vamos a brincar…

P#4EG

Créelo, la modificación genética…

Moderadora

Sí. Sí.

Pregunta 5- ¿Apoya usted esta actividad en su comunidad? ¿Por qué? (*Preguntar a cada participante del grupo de discusión*)

[se contestó en otra sección]

Pregunta 5a- ¿Piensa que su comunidad apoyaría esta actividad? Sí, No, ¿Por qué?

[se contestó en otra sección]

Pregunta 6- ¿Qué otra información necesitaría para entender mejor esta actividad?

Moderadora

¿Qué información adicional necesitarían? ¿Alguna otra?

P#3TI

Lo que pasa es que se mutan y por eso hay tantas cosas diferentes saliendo. [risas]

P#2LD

Todos los estudios que se han hecho.

P#1LD

Los resultados de todos los estudios.

Moderadora

Los resultados. Y todos los estudios, diría número dos (*P#2LD*).

P#3TI

Es como la influenza, que se sigue mutando, y sigue mutando, y sigue mutando. Y siguen creando, nuevas cepas.

P#2LD

Sí porque eso muta, eso es mutante.

P#4EG

No y los animales…

Moderadora

Es que están hablando los dos y quiero escuchar.

P#3TI

Ay perdón.

Moderadora

No, no. Es que quiero escucharlos. ¿Qué dice…?

P#2LD

Que son mutantes.

Moderadora

Ah, que son mutantes.

P#2LD

Eso muta. O sea, que cualquier cosa puede pasar, por eso yo no creo en el laboratorio, porque ellos pueden mutar. Son cosas que pueden pasar.

Moderadora

Ok. Que podrían hacer daño, es lo que estoy entendiendo.

P#2LD

Exactamente. Ellos están trabajando con una cosa y después te va a salir por el otro lado.

Moderadora

Ok. Ok. Entiendo. Y número tres (*P#3TI*) iba a decir algo.

P#3TI

Pues yo estaba comentando con número cuatro (*P#4EG*), que las mutaciones son las consecuencias ahora mismo de tantas cosas, porque, todos los años sale una cepa nueva de influenza. ¿Y de qué es consecuencia? De las mutaciones, de las mezclas, las investigaciones… que nos siguen creando efectos negativos en nuestra salud.

Moderadora

Ok. Está bien.

**Parte 3- Cierre de sesión**

Pregunta 10- ¿En quién de su comunidad confiarían ustedes para hablar sobre estas actividades?

Moderadora

Entonces, bueno, ya ahí terminan las preguntas, pero les voy a hacer dos preguntitas finales. O tres, mejor dicho. ¿En quién de su comunidad, pensando ustedes en sus comunidades, confiarían ustedes para que hablen sobre todas estas actividades? Si van a hacer, por ejemplo, un adiestramiento y… piensan en alguien de sus comunidades, en quienes confiarían. Sí.

P#2LD

¿De las comunidades de nosotros?

Moderadora

Sí. Sí.

P#2LD

Yo me atrevo… a mí mismo…

Moderadora

Usted mismo, verdad. Ok. Chévere. ¿Y por acá? Si me… ¿Se viene alguien a la mente?

P#1LD

Sí, porque es la misma comunidad de él.

Moderadora

Es la misma comunidad de…. Bueno, ok. Pues lo dejamos en número dos (*P#2LD*). [risas]

P#2LD

Uno (*P#1LD*) y dos (*P#2LD*) es lo mismo.

P#3TI

Yo entiendo que, en mi comunidad, yo me atrevería y tengo una parte integrante de la directiva de la comunidad que es una enfermera epidemióloga…

Moderadora

Ay que bueno.

P#3TI

…que podría cooperar conmigo.

Moderadora

Ok. Chévere. ¿Y por acá, usted?

P#4EG

Pues yo creo que puede haber varios candidatos. Es cuestión de comunicarlo. Y con un comunicado general van a reaccionar. Y ahí voy a poder determinar quiénes son los recursos.

Pregunta 11- ¿Cuál sería la mejor manera de hablar a su comunidad sobre estas actividades?

Moderadora

Ok. Chévere. ¿Cuál sería la mejor manera de hablar a su comunidad sobre estas actividades?

P#1LD

Por convocatorias como está la gente no viene.

Moderadora

No vienen a estas convocatorias.

P#1LD

No vienen.

Moderadora

Esta no sería una, en LD.

P#1LD

Una alternativa, voy a hacer, este… o llevándole panfletos entregándosela a las manos, ver que ellos lo lean y después uno va a conversar con ellos a ver si se han reunido o escrito en el panfleto y si están de acuerdo…

Moderadora

Ok. Escrito, de forma escrita.

P#1LD

Pero hay que impactarlos a ellos en su mismo hogar. Porque traerlos acá, imposible.

P#2LD

Yo creo que se pueden traer aquí.

Moderadora

¿Sí? Usted cree.

P#2LD

Pero hay que disfrazarlo.

Moderadora

¿Cómo lo disfrazaríamos?

P#2LD

Con una feria de salud.

Moderadora

Una feria de salud.

P#3TI

Sí…

P#2LD

Es lo mismo, estamos hablando desde cierto punto, le traemos una feria de salud, le cogemos la presión, le cogemos… entonces, le ponemos un par de sillas y les damos una conferencia de los mosquitos al mismo tiempo. O sea, hay que disfrazarlo para traerlos. Como tirar una bola de dos montes…

Moderadora

Ok. Chévere.

P#3TI

Yo pienso de igual manera que el número dos (*P#2LD*), con una buena feria con una buena comunicación, y varios atractivos…

P#2LD

Seguro.

P#3TI

… a parte de la feria de salud, ‘pues mira, va a haber esto, va a haber lo otro’, verdad. Y ahí pues, lo impactas. Y de esa forma esos que fueron ahí van a ser portavoces, ‘mira, y por qué vieron allí, funcionó…’

Moderadora

Ok. Buena… y usted, ¿qué piensa?

P#4EG

Sí. Yo estoy de acuerdo con la tres (*P#3TI*), de hecho, yo he participado en una y son bien exitoso. Siempre y cuando la comuniques con tiempo, y puedas organizarla bien, este… pues, han sido bien exitosas.

Moderadora

Ok. Que bien, su comunidad también….

P#4EG

La gente queda bien satisfecha.

Moderadora

¡Qué bueno! Y… ah… En general, ¿cuál de todas las actividades presentadas les gustó más?

P#1LD

No entendí bien la…

Moderadora

Que las vieron todas.

P#4EG

Varias.

Moderadora

¿Varias?

P#4EG

Sí, sí. La uno, la dos y la tres. Que eran las que yo conocía.

Moderadora

Uno, dos y tres, larvicida, y reducción de fuentes.

P#2LD

Las que no me gustaron fueron las últimas tres.

Moderadora

Las últimas tres no le gustó. Ok. Ok. Está muy bien.

P#4EG

Bueno, no nos han gustado por desconocimiento y otra por principio.

Moderadora

Sí. Sí. Y eso lo entendemos, porque ha sucedido en otros grupos también. Bueno pues voy a ir apagando aquí porque ya hasta aquí…

*****Fin del audio*****
