# Supplementary material for: Acceptability of emergent Aedes aegypti vector control methods in Ponce, Puerto Rico: A qualitative assessment
Source: PLOS Glob Public Health. 2024 Mar 6;4(3):e0002744. doi: 10.1371/journal.pgph.0002744 (PMC10917327; doi:10.1371/journal.pgph.0002744)
Supplement: S3 Appendix — Ponce, Puerto Rico, 2020–2021. (DOCX) [file pgph.0002744.s003.docx]

**S3 Appendix. Community Outreach Activities conducted as part of the COPA (Communities Organized to Prevent Arboviruses) project. Ponce, Puerto Rico, 2020-2021**

| **Activity** | **Year 2020** | **Year 2021** |
| --- | --- | --- |
| Educational booths | 30 | 22 |
| Educational talks | 54 | 18 |
| Community visits | 7,535 | 1,527 |
| Workshop or training | 8 | 7 |
| Distribution of educational material | 16,771 | 11,829 |
| Hours of loudspeakers | 160 | 80 |
| Posters | 190 | 4 |
